# Sequential Change of Wound Calculated by Image Analysis Using a Color Patch Method during a Secondary Intention Healing

**DOI:** 10.1371/journal.pone.0163092

**Published:** 2016-09-20

**Authors:** Sejung Yang, Junhee Park, Hanuel Lee, Soohyun Kim, Byung-Uk Lee, Kee-Yang Chung, Byungho Oh

**Affiliations:** 1 Institute of Art and Science Convergence, Ewha Womans University, Seoul, Republic of Korea; 2 Department of Electronics Engineering, Ewha Womans University, Seoul, Republic of Korea; 3 Department of Dermatology and Cutaneous Biology Research Institute, Yonsei University College of Medicine, Seoul, Republic of Korea; 4 Department of Dermatology, Keimyung University, College of Medicine, Daegu, Republic of Korea; IDI, Istituto Dermopatico dell'Immacolata, ITALY

## Abstract

**Background:**

Photographs of skin wounds have the most important information during the secondary intention healing (SIH). However, there is no standard method for handling those images and analyzing them efficiently and conveniently.

**Objective:**

To investigate the sequential changes of SIH depending on the body sites using a color patch method

**Methods:**

We performed retrospective reviews of 30 patients (11 facial and 19 non-facial areas) who underwent SIH for the restoration of skin defects and captured sequential photographs with a color patch which is specially designed for automatically calculating defect and scar sizes.

**Results:**

Using a novel image analysis method with a color patch, skin defects were calculated more accurately (range of error rate: -3.39% ~ + 3.05%). All patients had smaller scar size than the original defect size after SIH treatment (rates of decrease: 18.8% ~ 86.1%), and facial area showed significantly higher decrease rate compared with the non-facial area such as scalp and extremities (67.05 ± 12.48 vs. 53.29 ± 18.11, *P* < 0.05). From the result of estimating the date corresponding to the half of the final decrement, all of the facial area showed improvements within two weeks (8.45 ± 3.91), and non-facial area needed 14.33 ± 9.78 days.

**Conclusion:**

From the results of sequential changes of skin defects, SIH can be recommended as an alternative treatment method for restoration with more careful dressing for initial two weeks.

## Introduction

Secondary intention healing (SIH) is a simple method of wound management that provide excellent cosmetic results [1]. Although there are some drawbacks such as a prolonged healing period, a need for regular and frequent dressing changes, and the risk of infection, it can reduce the size of scarring more than that of skin defects which heal through the natural wound contraction and epithelialization [2,3]. There are many reports showing the acceptable functional and cosmetic outcomes of SIH especially when applied to fingertip amputation [4], concave areas of the face [5], and skin defects following wide excision of melanoma on the foot [6]. In addition, the results of SIH are greatly improving with the newly developed methods such as negative pressure wound therapy (NPWT) and growth factors, combined with existing various dressing materials for the optimal moisturization [7–11].

Nevertheless, dermatologic surgeons cannot easily select this method for restoring skin defects due to difficulty in predicting final results which vary depending on the location, size, depth, concomitant diseases, and dressing materials. Therefore, for the appropriate application of SIH method, it is necessary to establish a system of calculating the total required time for the completion of wound healing and predicting the final cosmetic results. In that respect, photographs of skin defects will provide more accurate information than “length (L) × width (W)” measurement [12]. However, there is no standard method of how to take those images and analyze them effectively and conveniently. In addition, in predicting and preventing the occurrence of scarring, it is also important to detect accurately the sequential changes of SIH, because abnormal wound healing process also acts on final scars [13,14].

For the convenient extraction of information from the images of wounds and scars, the authors designed the color patch method and applied it to measure the sequential changes of the size and color of wounds during the secondary healing process. Herein, we evaluate the error rate of this method for measuring skin defects, and compare the therapeutic results of SIH between facial and non-facial area.

## Materials and Methods

### Color Patch Method for Automatically Calculating Defect sizes

**Fig 1:**
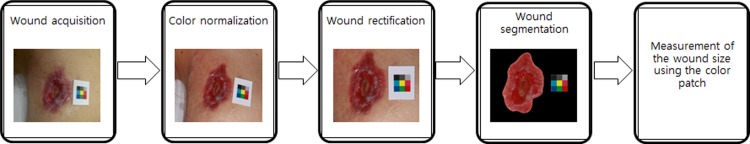
Block diagram for measurement of wound sizes. To measure the size of wound precisely, pre-processing steps are needed before segmentation.

The color patch method is designed for effectively measuring defect sizes by comparing them to the true area of the color patch. The designed color patch is provided in Fig 1. The size of each color segment is 0.7 cm in width and 0.7 cm in height, and the gross area of color patches is 2.1x2.1 cm^2^. On the basis of this absolute size, the wound sizes were estimated reflecting the amount of variations in size among color patches appearing in the pictures. The patch should be placed in the same plane as the wound as much as possible for the delicate measurement. The variations in color change depending on the photographing conditions were adjusted based on the color of patches. The areas which show different color and texture compared to those around normal skin areas are defined as skin wounds and scarring. For this segmentation of the wounds, boundary detection was performed using the gradient vector flow (GVF) snake algorithm [15]. This algorithm is an effective segmentation tool attracting active contour towards object boundaries from a relatively large distance. GVF method was selected for the wound segmentation in that this method converges to object cavities and performs well for a closed curve area such as wounds and scars. A block diagram for wound measurement is shown in Fig 1. After acquiring a wound image, color normalization is performed to reduce the influence of color difference between devices. Next, image rectification is processed to measure the size precisely. Finally, the wound is segmented and the wound size is calculated.

**Fig 2:**
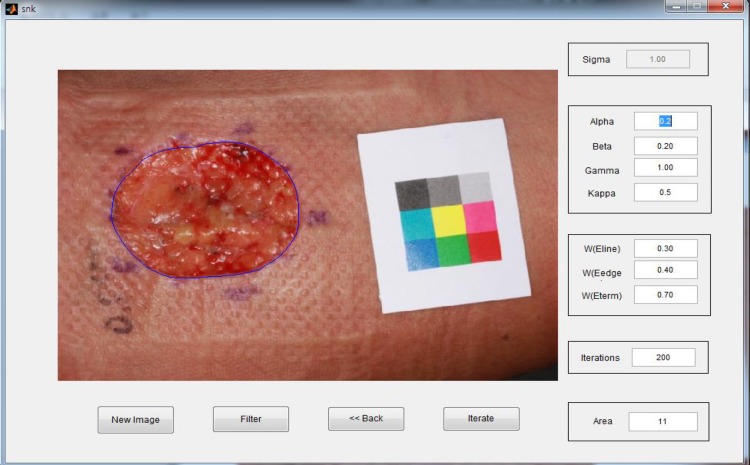
Wound image analysis using a color patch method. The area which shows different color and texture compared to around normal skin area was automatically defined as a skin wound using the devised software tool.

All calculating procedures were automatically performed using the devised software tool (Fig 2). Since the initial point is designed to set up automatically for GVF method by means of ‘K-means clustering algorithm’ [16], there is no need of user intervention. Exhaustive search was performed to find the fixed values of parameters, alpha, beta, gamma and kappa as shown in Fig 2. The values of alpha, beta, gamma and kappa are 0.2, 0.2, 1.0 and 0.5, respectively.

**Fig 3:**
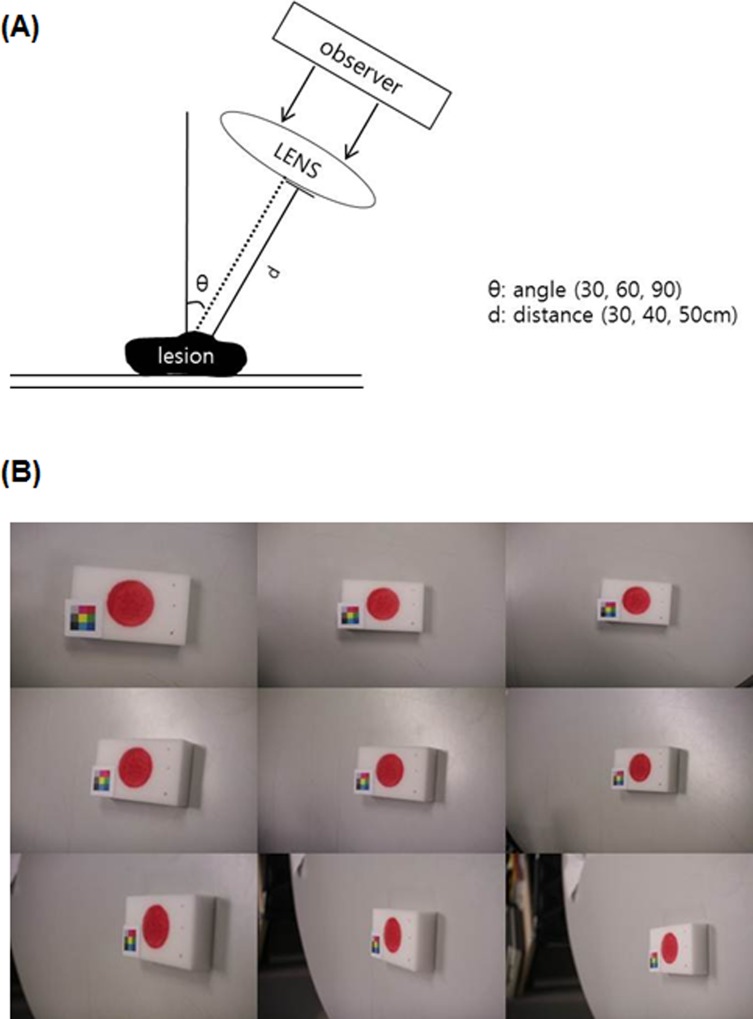
Experimental model for calculating the error rate of color patch method. (A) The capturing system (B) Images captured from 30, 40, and 50 cm distances and at 30, 60, and 90 angles.

Before applying this method to analyze the sequential changes of SIH, we calculated the error rate using a model of skin defect to evaluate this method. In this model, the diameter of defect was 2 cm and the total area was 12.57 cm^2^. Images were captured from 30, 40, and 50 cm distances and at 30, 60, and 90 angles and compared to the true value of the defect area (Fig 3).

### Patients Who Have Skin Defects

We performed a retrospective review of 30 patients (12 males and 18 females) who underwent SIH for the restoration of skin defect from January 2013 to February 2015 at the Severance Hospital in Yonsei University Health System, Seoul, Korea. When the patient visited the hospital for the dressing, the wound size was calculated by the color patch method. We performed a review of each patient’s medical record, including age, sex, location, date and methods of treatment, the presence of complications, and patient’s complaint and satisfaction. This study protocol was approved by the Institutional Review Board of Yonsei University, Severance Hospital (IRB No. 4-2013-0511) and was conducted according to the Declaration of Helsinki Principles. Patient records/information was anonymized and de-identified prior to analysis.

### Dressing Methods for the Secondary Intention Healing

With respect to the methods of dressing for SIH, if there was a heavy exudate from wounds, we performed cold wet dressing using hyperosmotic fluid such as liquid alum, and then attached transparent silicone to minimize the damage to skin and lastly applied the gauze. If there was a light to moderate exudate, foam dressing was applied and exchanged, every 2 or 3 days [11]. Oral antibiotics were only administered when there were definite infection signs such as erythema, swelling, and tenderness. At the end of treatment, we evaluated patients’ satisfaction with this treatment on a 4-point grading scale (0 = unsatisfied, 1 = slightly satisfied, 2 = satisfied, 3 = very satisfied).

### Photograph Analysis

A total of 201 photograph images from 30 patients were collected. We calculated the sequential changes in the size of skin wounds over time by the color patch method. The degree of decrease in wound was calculated based on the initial skin defect size at the beginning of dressing and the final scar size after the follow up period. In addition, to evaluate the velocity of wound healing, we estimated the date corresponding to the half of the final decrement, which was resulted from the calculation by the color patch method.

### Evaluation of the Clinical Outcomes

For the evaluation of the clinical outcomes, patients’ final photographs were evaluated by two individual dermatologists using visual analog scale (VAS) scores in a blinded manner. The VAS is a 10-cm linear scale in which the left of the scale (score of 0) reflects the worst outcome and the right of the scale (score of 10) reflects the best outcome. In addition, Patients’ final scars were evaluated using the Vancouver Burn Scar Assessment Scale (VBSAS), which includes pigmentation (0 = normal, 1 = hyperpigmented, 2 = hypopigmented), vascularity (0 = normal, 1 = pink, 2 = red, 3 = purple), pliability (0 = normal, 1 = supple, 2 = yielding, 3 = firm, 4 = contracture) and height (0 = flat, 1 = 0–2 mm, 2 = 2–5 mm, 3 = >5 mm). The score for each parameter was assessed separately, and then all four scores were summed.

### Statistical Analysis

Discrete variables were described with counts and percentages. For continuous variables, the median (range) or mean ± standard deviation were calculated, as appropriate for the distribution of the data. Group differences between facial and non-facial area in age, follow up periods, initial defect size, final scar size, estimated duration for 50% improvement, VAS score, VBSAS score and patients’ satisfaction were evaluated using nonparametric Mann-Whitney U-test. Difference between two groups in degree of decrease was evaluated using independent samples *t*-tests. Linear regression analysis was performed to evaluate the association between the degree of decrease and initial defect size and estimated duration for 50% improvement. All statistical analyses were performed with SPSS (version 19.0, SPSS, Inc., Chicago, IL), with the level of significance set at *P* < 0.05.

## Results

The mean age of the 30 patients who received SIH for the restoration of skin defects was 53.8 ± 18.9 years (range: 14–81 years). 11 patients had skin defects on the face, and 19 patients had them on the non-facial area such as scalp, shoulder, upper and lower extremities. The average period of follow up was 93.9 ± 59.2 days and during this period, there was no patient who had any side effects such as secondary infection or severe pain.

The color patch method was very useful for detecting the sequential changes of SIH accurately and conveniently. The range of error rate of our color patch method from the experimental model was from -3.39% to +3.05% (Table 1). The picture taken from 30cm distance and 90 degree angle had the lowest difference (-0.05%) from the original size of defect, and that from 50cm distance and 60 degree angle had the highest difference (-3.39%).

**Table 1:** Results of area measurement and error rate from the experimental model of color patch method (d: distance (cm), *θ*: angle (degree)).

	d: 30 *θ*: 90	d: 30 *θ*: 60	d: 30 *θ*: 30	d: 40 *θ*: 90	d: 40 *θ*: 60	d: 40 *θ*: 30	d: 50 *θ*: 90	d: 50 *θ*: 60	d: 50 *θ*: 30
measured area (cm^2^)	12.56	12.84	12.83	12.81	12.23	12.82	12.81	13.10	12.95
error rate (%)	-0.05	2.18	2.10	1.94	-2.68	2.02	1.94	-3.39	3.05

**Fig 4:**
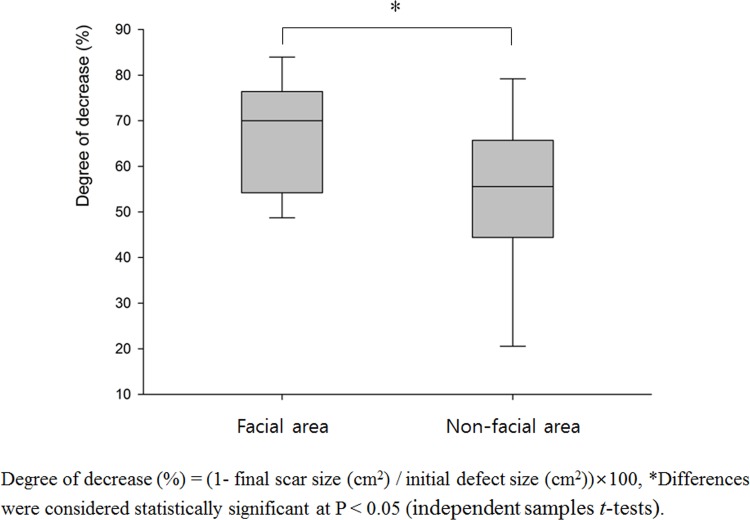
Degree of decrease after final scarring in the facial and non-facial skin defects.

**Fig 5:**
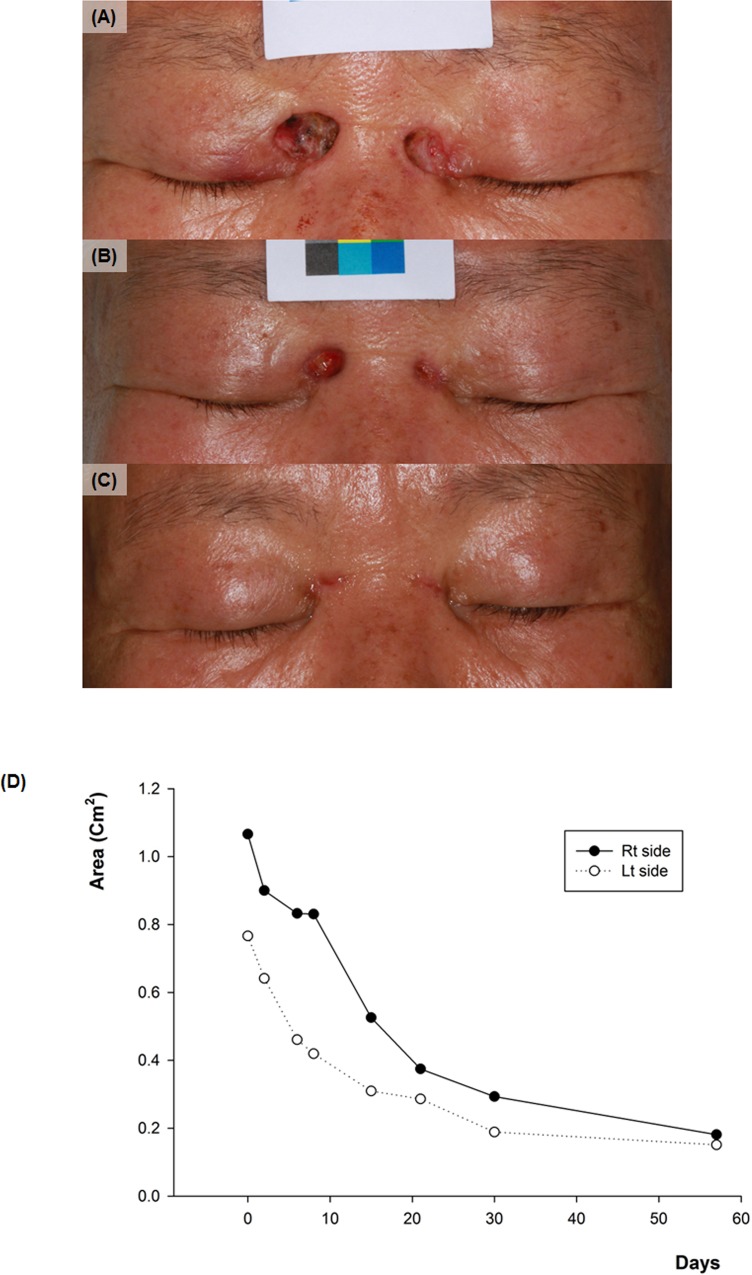
**Sequential changes of skin defect on the medial canthus area** (A) Initial image of case no.20 (B) 15 days after (C) 57 days after. (D) The graph of change in size of the skin defect on each side with time.

**Fig 6:**
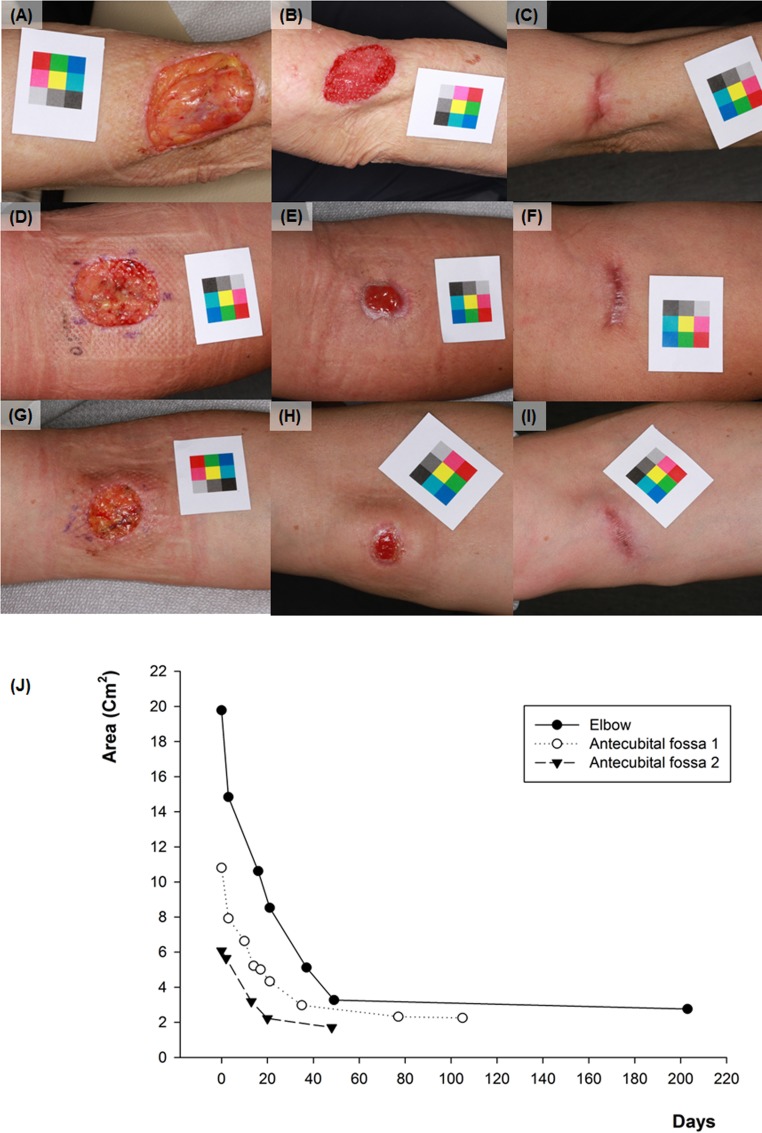
Sequential changes of skin defect on the elbow and antecubital fossa. (A) Initial image of case no.7 (elbow) (B) 21 days after (C) 203 days after (D) initial image of case no.8 (antecubital fossa) (E) 21 days after (F) 105 days after (G) initial image of case no.9 (antecubital fossa) (H) 20days after (I) 48days after. (J) The graph of change in size of the skin defect on each site with time.

Using this method, we calculated that all patients had smaller scar size than the original defect size after SIH treatment (rates of decrease: 18.8% ~ 86.1%). After the final follow up, facial area showed significantly higher decrease rate (67.05 ± 12.48%) compared with the non-facial area (53.29 ± 18.11%) (Fig 4). Among the facial areas, thin and concave areas such as medial canthus showed impressive results (Fig 5). Among the non-facial areas, antecubital areas showed high decrease rate (71.9 ~ 86.0%), and interestingly, the direction of final scars was corresponded to the relaxed skin tension line (Fig 6). On the other hand, the lowest degree of decrease was appeared on the shoulder (18.8%), which has not only a relatively thick thickness but also greater mobility than other sites.

**Fig 7:**
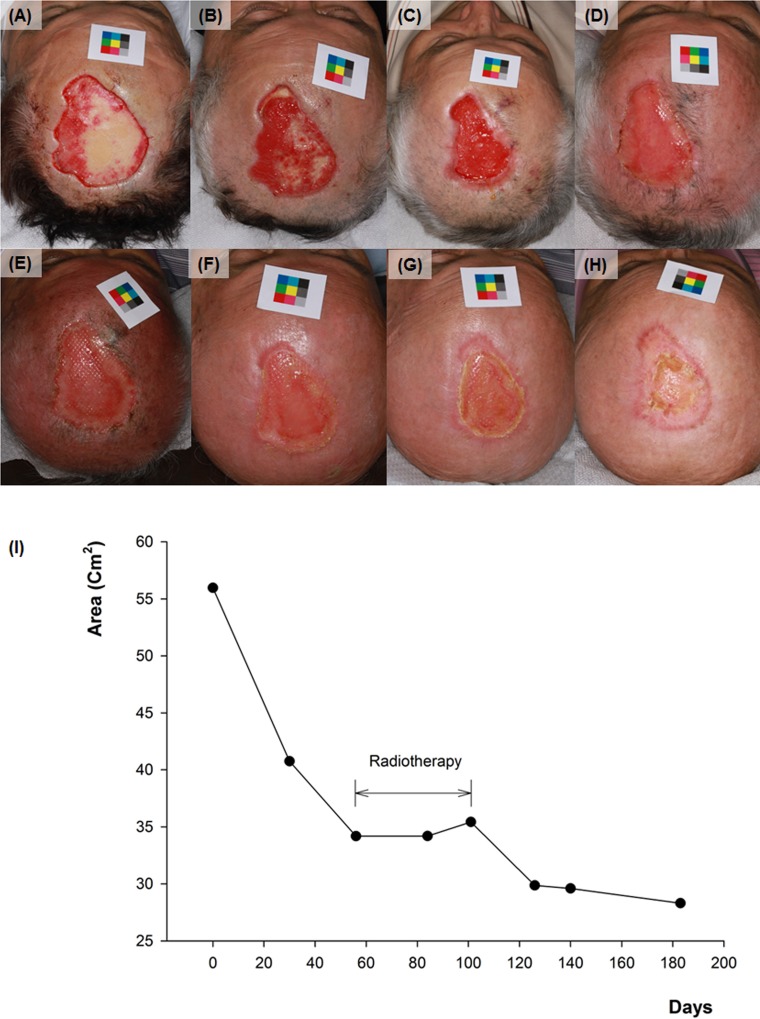
Sequential changes of skin defect on the scalp. (A) initial image of case no.1 (B) 30 days after (C) 56 days after (D) 84 days after (E) 101 days after (F) 126 days after (G) 140 days after (H) 183 days after. (I) The graph of change in size of the skin defect with time.

From the result of estimating the date corresponding to the half of the final decrement, all of the facial area showed improvements within two weeks (8.45 ± 3.91), and non-facial area needed 14.33 ± 9.78 days (Table 2). It took longer time to reach this improvement if there was a vascular disorder such as livedoid vasculopathy, or a need of radiotherapy after surgery (Fig 7). Although there were significant differences in age, follow up periods, initial defect size, final scar size, estimated duration for 50% improvement between facial and non-facial area group, there was no correlation between the decrease rate and each factors (linear regression analysis).

**Table 2:** Characteristics of patients in the facial area group and the non-facial area group.

	Facial area (N = 11)			Non-facial area (N = 19)			
	Average	SD	Range	Average	SD	Range	P-value*
Age	63.64	12.80	42–81	48.11	19.86	14–76	<0.05
Sex							N/A
Male (n)	5			7			
Female (n)	6			12			
Follow up periods (days)	69.82	47.30	30–162	107.84	61.94	34–239	<0.05
Initial defect size (cm^2^)	1.91	1.26	0.21–4.21	10.46	16.85	0.35–57.25	<0.01
Final scar size (cm^2^)	0.59	0.50	0.11–1.60	5.54	6.36	0.14–45.48	<0.01
Estimated duration for 50% improvement (days)	8.45	3.91	1.0–13.0	14.33	9.78	1–46	<0.05
VAS score	6.14	2.11	3.0–8.5	5.24	1.69	3.0–8.0	0.171
VBSAS score	4.91	1.92	2.0–8.0	5.95	1.81	3.0–10.0	0.150
Patients’ satisfaction	1.91	0.83	1.0–3.0	1.79	0.86	0–3	0.800

SD, standard deviation; N/A, not applied; Estimated duration for 50% improvement, the date corresponding to the half of the final decrement.

*: Nonparametric Mann–Whitney U-te.

Average patients’ satisfaction, VAS and VBSAS score were 1.83 ± 0.83, 5.57 ± 1.87, 5.57 ± 1.89, respectively, and there were no significant differences between facial and non-facial area group (Table 2).

## Discussion

Human skin has its own healing ability to repair skin damage or to restore the skin defect by itself without any suture procedure. Although it requires a lot of time to complete it, SIH is the process using this healing ability to protect our body from the external environment and adapt to given environmental conditions [17]. If SIH was applied to ancient people who lived by hunting and gathering, natural healing would be impeded or their lives would be threatened, because there were frequent chances of infection by foreign body, bacteria, fungus and etc. intruding into their body through the skin defect. However, it is rarely of any relevance to modern people who live in the city. In addition, various dressing materials and advanced products for protecting skin and improving the healing ability have been developed. Therefore, SIH can be recommended as an alternative treatment method for the skin defect. In addition, after first performing SIH with the consent of patients, the need for scar revision to be assessed at a later time can be evaluated.

In particular, the fact that skin defects occurred in early prenatal phase can be healed by tissue regeneration without scarring has recently emerged [18]. According to this discovery, the characteristics of tissue regeneration during this period, mechanical stress and inflammatory response are less as well as growth factor and abundant stem cells are provided. Therefore, this can be used to drastically reduce the incidence of scarring after treatment of SIH [19]. On the other hand, scarring is an inevitable result of the repair procedure with suture technique, because it is impossible to eliminate mechanical stress completely.

The barrier to select SIH methods for restoring the skin defect is its difficulty to predict the process and results due to many variables such as the location and depth of wound, the occurrence of complications like infections, patient’s age, compliance and genetic factors. Even more substantial is that there is no standard method for physicians or patients to estimate accurately and evaluate easily patients’ wound during the treatment.

In this study, in order to solve these problems, changes in wound defect and scarring in relation to the time were measured by attaching a simple color patch. From our experimental model, error rate of this method was from -3.39% to + 3.05%, which was minimized by taking picture closely with 90 degree angle. On the other hand, conventional “L × W” measurement was reported that it overestimates the true area of a circle by 27%, and it is double the true area of a triangle. It also has limitations such as subjective interpretation and interobserver variability [12,20–22]. Another advantage of this method is that using color calibration, a consistent value for area is measured regardless of lighting conditions and types of cameras, and it is unnecessary to make an effort to take pictures from the same distance.

A total of 30 patients enrolled in this retrospective study were successfully treated by SIH without any side effects, and their final scar size was decreased more than the first one from the result of calculating by the color patch method. Facial area especially on the thin and concave area showed significantly higher decrease rate compared with the non-facial area. This result correspond well with those of the earlier study, which reported that wounds in concave areas of the face healed by secondary intention had a high chance of healing with an excellent cosmetic outcome, especially if these wounds were small, superficial, and located near the medial canthus and medial cheek [5]. Another interesting finding was that the final scar shape on antecubital fossa was corresponded to RSTL. This result accounts for that mechanical stress depending on the movement influences on the process of SIH. Also, this may be used as a data when explaining to patients about the final scar before the treatment initiation. Actually, if patients with skin defect on this area undergo primary closure, they usually experience pain due to tension when moving, and they are likely to be at a higher risk for scar widening. Even if skin graft was conducted on this area, cosmetic results can be unacceptable if the skin texture of graft is different from the wound site and another scarring of donor site occurs.

From the results of the evaluation of wound healing velocity, all of the facial area showed improvements reaching to the half of the final decrement within two weeks (8.45 ± 3.91), and non-facial area needed 14.33 ± 9.78 days. Although it is necessary to consider the various factors related with wound healing in large samples, this result represents the importance of dressing for the initial two weeks.

The present study was limited by its retrospective design and by small sample size in verifying the statistical significance about many factors associated with wound healing. Regardless, our results of SIH treatments showed that it was acceptable for the restoration of skin defects not only on the facial area but also on the non-facial area especially such as antecubital area, with more careful dressing for initial two weeks. Image analysis using the color patch was very useful for calculating changes in the wound size as time elapsed. Further controlled trials in large sample size will be necessary to make a prediction system of final scars after SIH treatments.

## Supporting Information

S1 File Wound image data set for patient 1. (ZIP)

S2 File Wound image data set for patient 2. (ZIP)

S3 File Wound image data set for patient 3. (ZIP)

S4 File Wound image data set for patient 4. (ZIP)

S5 File Wound image data set for patient 5. (ZIP)

S6 File Wound image data set for patient 6. (ZIP)

S7 File Wound image data set for patient 7. (ZIP)

S8 File Wound image data set for patient 8. (ZIP)

S9 File Wound image data set for patient 9. (ZIP)

S10 File Wound image data set for patient 10. (ZIP)

S11 File Wound image data set for patient 11. (ZIP)

S12 File Wound image data set for patient 12. (ZIP)

S13 File Wound image data set for patient 13. (ZIP)

S14 File Wound image data set for patient 14. (ZIP)

S15 File Wound image data set for patient 15. (ZIP)

S16 File Wound image data set for patient 16. (ZIP)

S17 File Wound image data set for patient 17. (ZIP)

S18 File Wound image data set for patient 18. (ZIP)

S19 File Wound image data set for patient 19. (ZIP)

S20 File Wound image data set for patient 20. (ZIP)

S21 File Wound image data set for patient 21. (ZIP)

S22 File Wound image data set for patient 22. (ZIP)

S23 File Wound image data set for patient 23. (ZIP)

S24 File Wound image data set for patient 24. (ZIP)

S25 File Wound image data set for patient 25. (ZIP)

S26 File Wound image data set for patient 26. (ZIP)

S27 File Wound image data set for patient 27. (ZIP)

S28 File Wound image data set for patient 28. (ZIP)

S29 File Wound image data set for patient 29. (ZIP)

S30 File Wound image data set for patient 30. (ZIP)
