# Supplementary figures and images for: Sequential Change of Wound Calculated by Image Analysis Using a Color Patch Method during a Secondary Intention Healing (part 1 of 3)
Source: PLoS One. 2016 Sep 20;11(9):e0163092. doi: 10.1371/journal.pone.0163092 (PMC5029888; doi:10.1371/journal.pone.0163092)

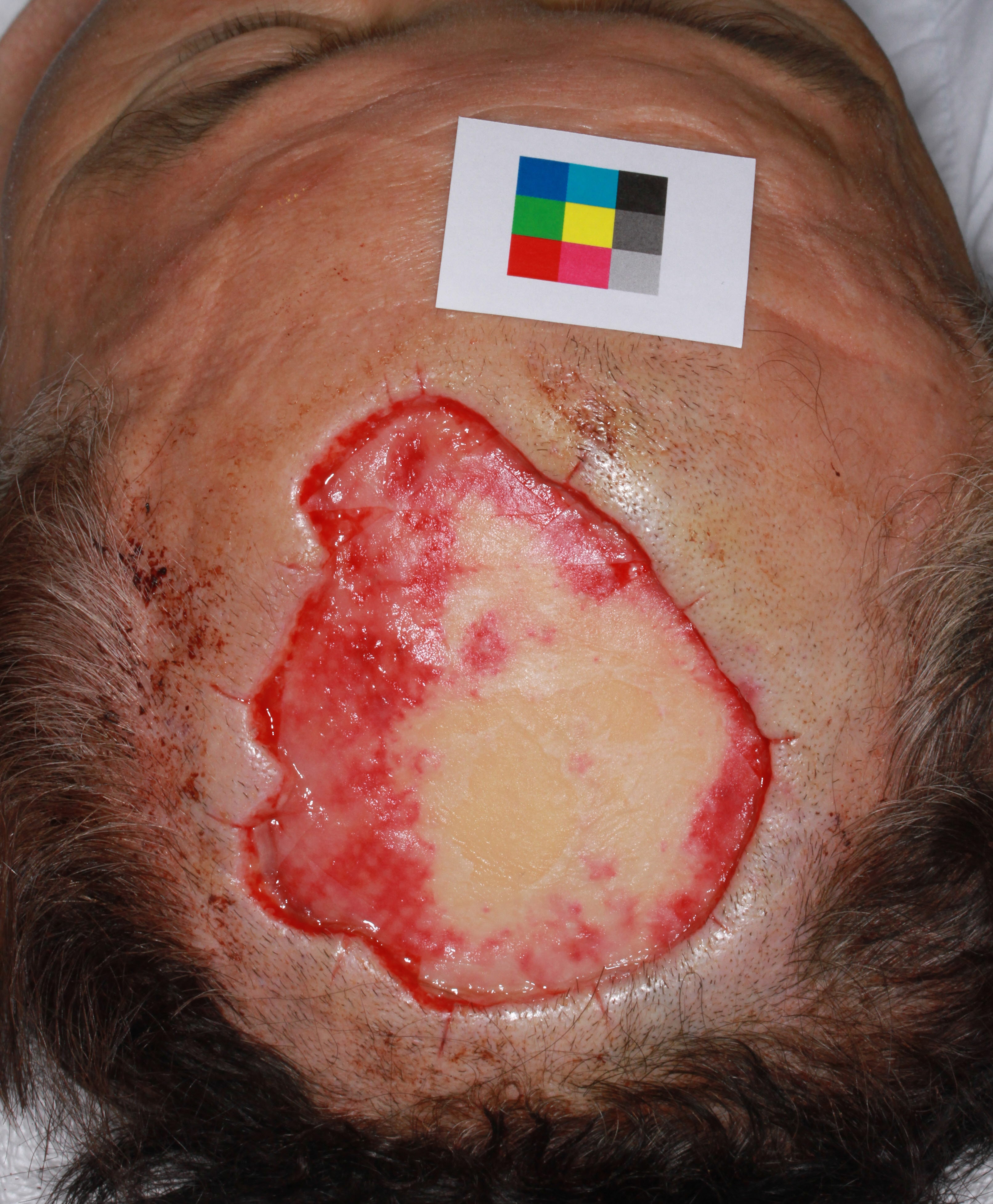

Supplement: S1 File — (ZIP) [file pone.0163092.s001.zip › 0430.jpg]

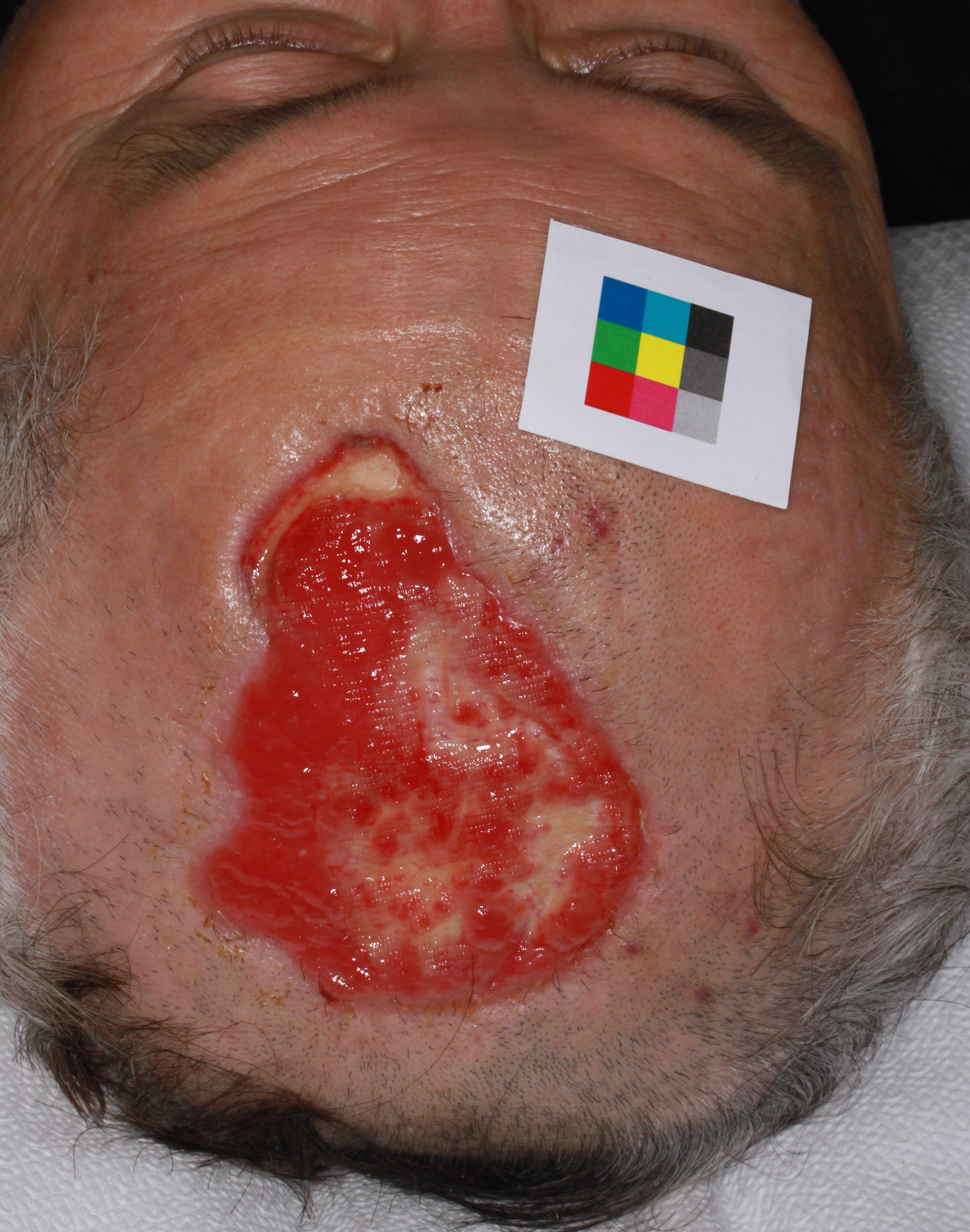

Supplement: S1 File — (ZIP) [file pone.0163092.s001.zip › 0530.jpg]

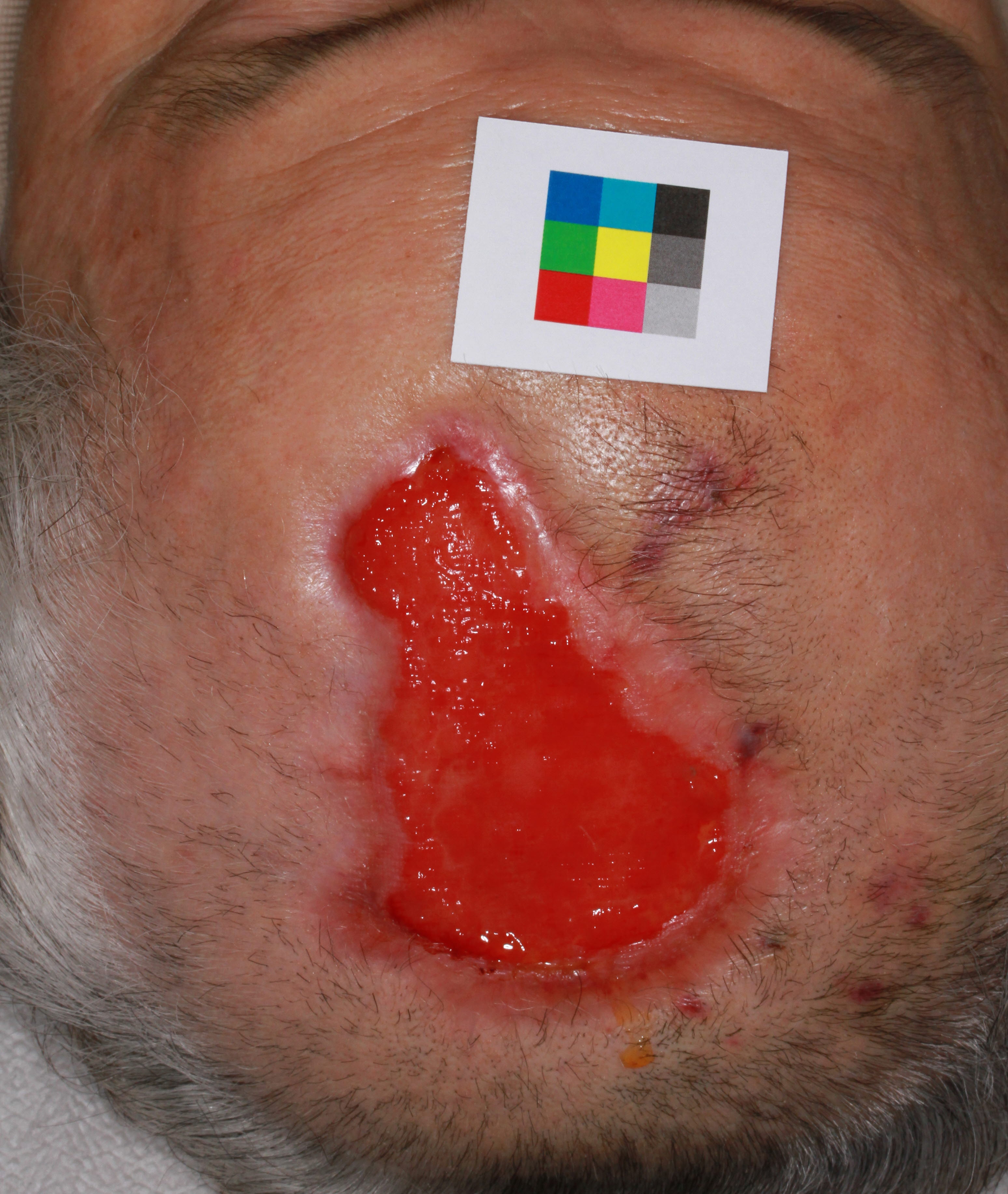

Supplement: S1 File — (ZIP) [file pone.0163092.s001.zip › 0625.jpg]

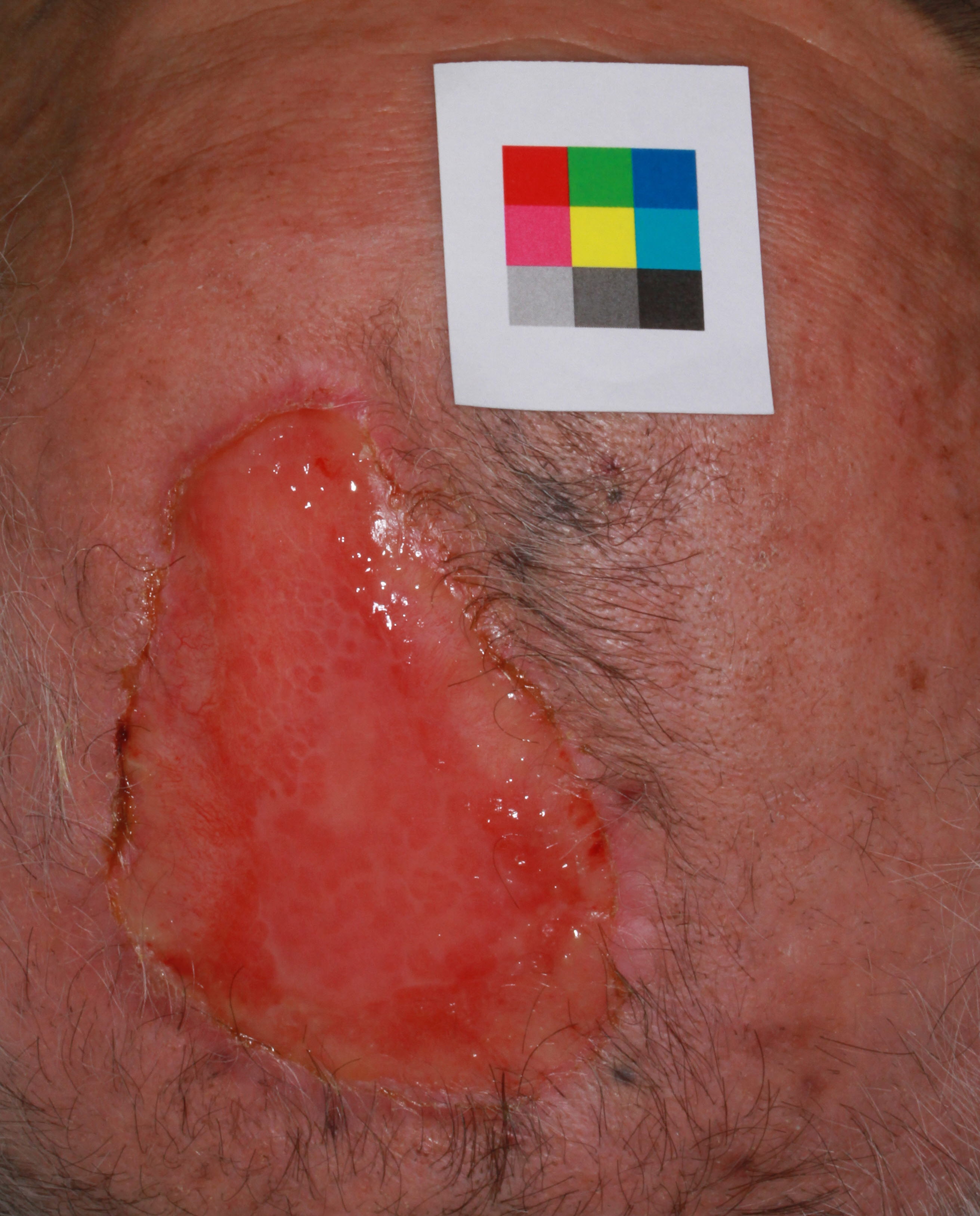

Supplement: S1 File — (ZIP) [file pone.0163092.s001.zip › 0723.jpg]

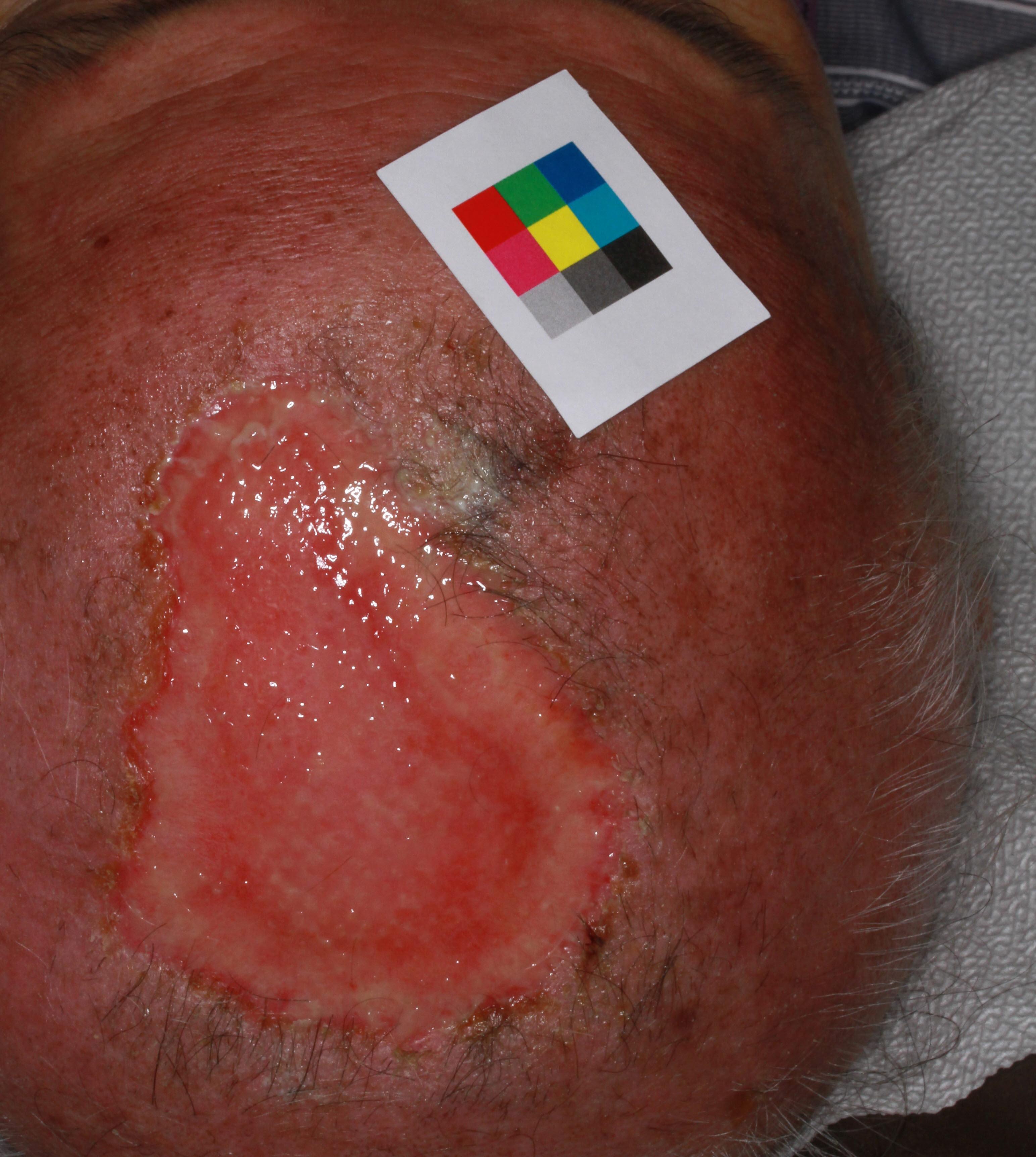

Supplement: S1 File — (ZIP) [file pone.0163092.s001.zip › 0809.jpg]

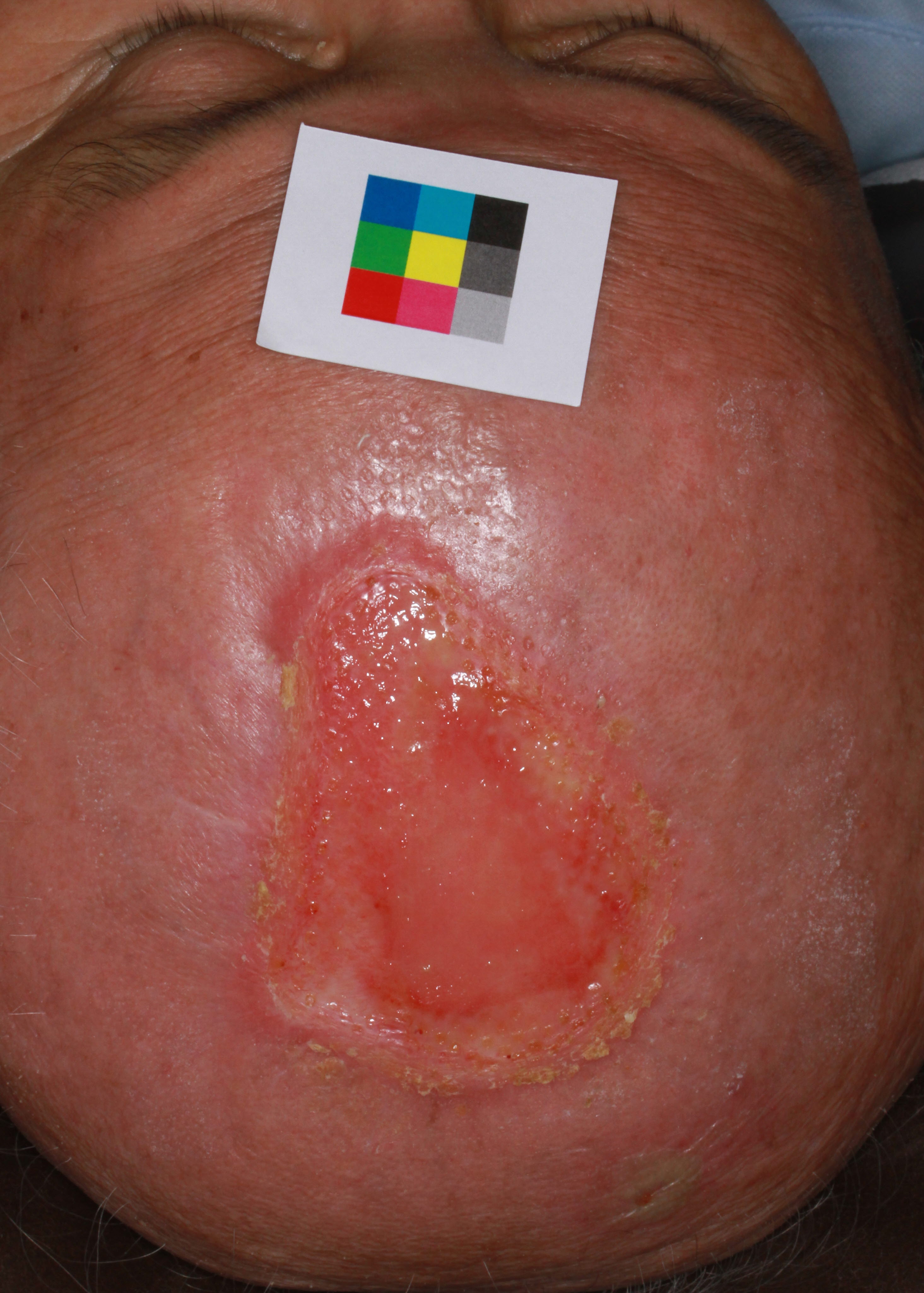

Supplement: S1 File — (ZIP) [file pone.0163092.s001.zip › 0903.jpg]

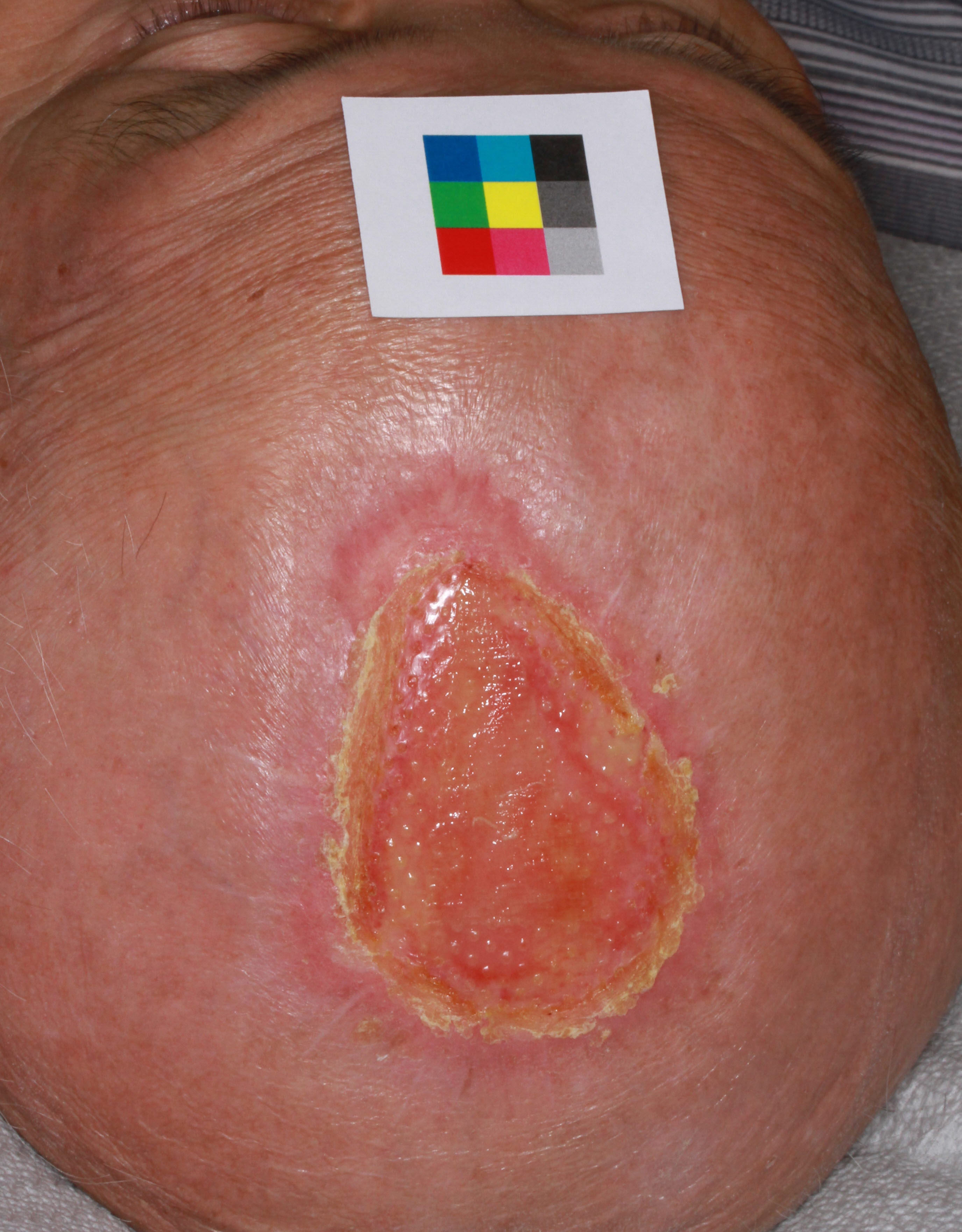

Supplement: S1 File — (ZIP) [file pone.0163092.s001.zip › 0917.jpg]

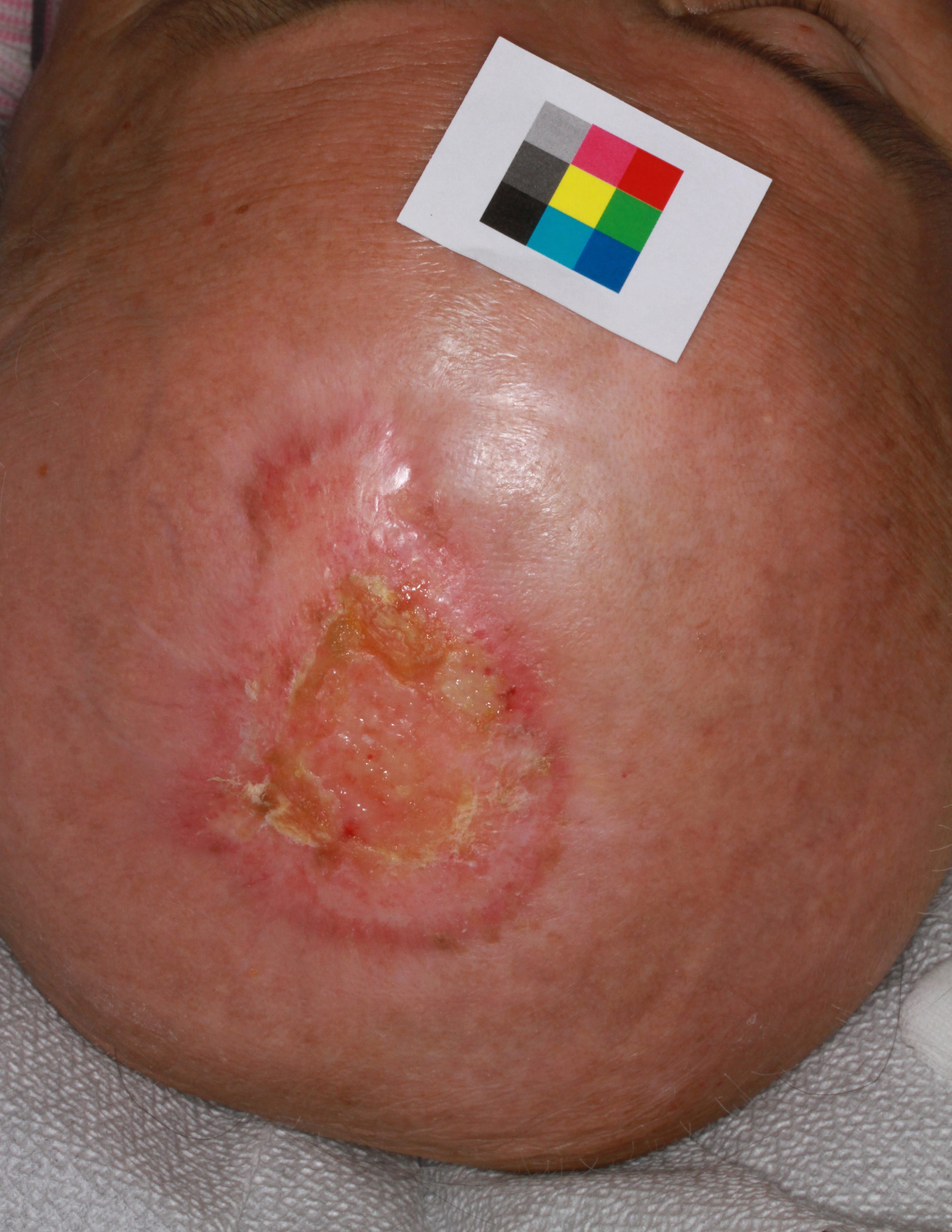

Supplement: S1 File — (ZIP) [file pone.0163092.s001.zip › 1030.jpg]

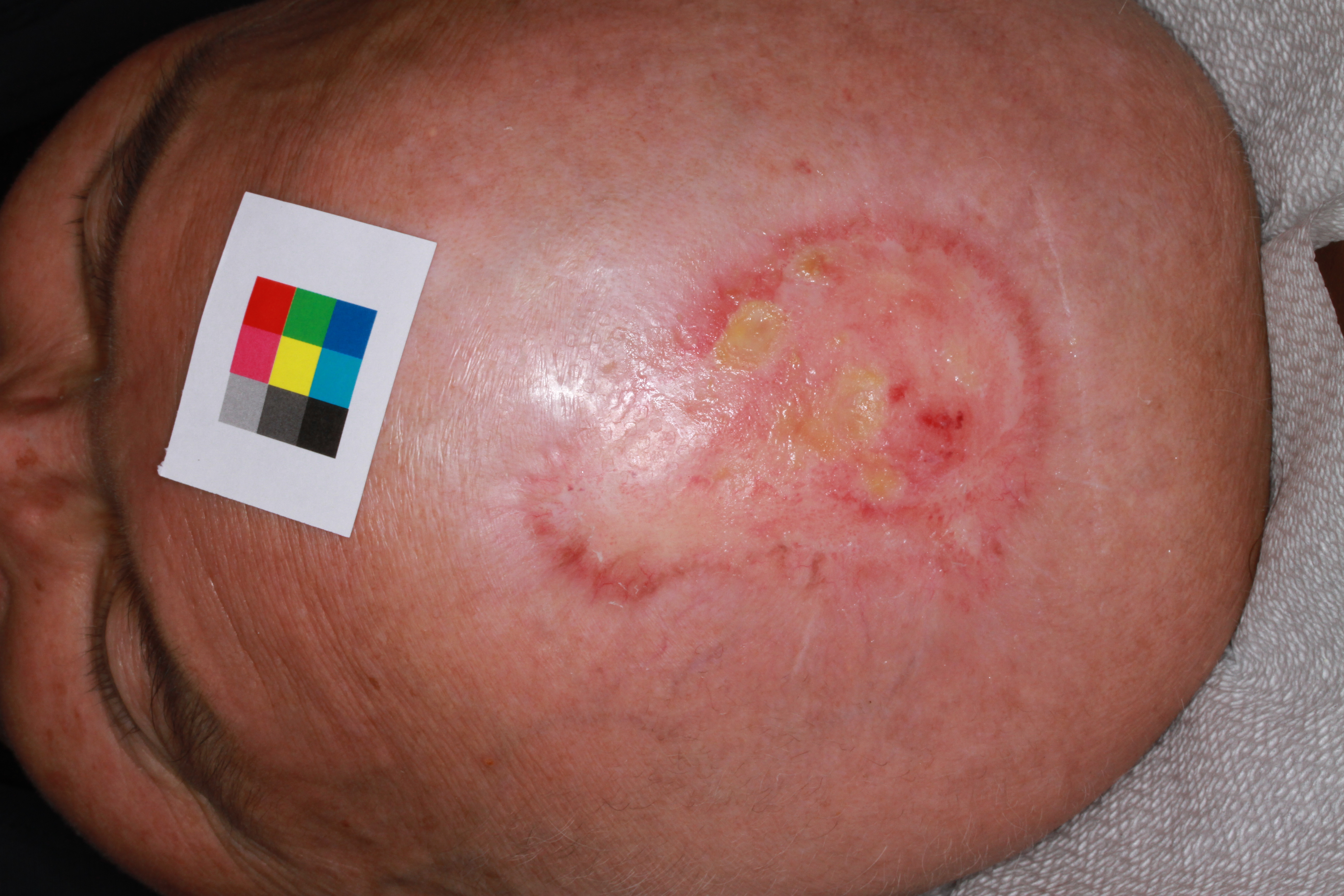

Supplement: S1 File — (ZIP) [file pone.0163092.s001.zip › 1127.JPG]

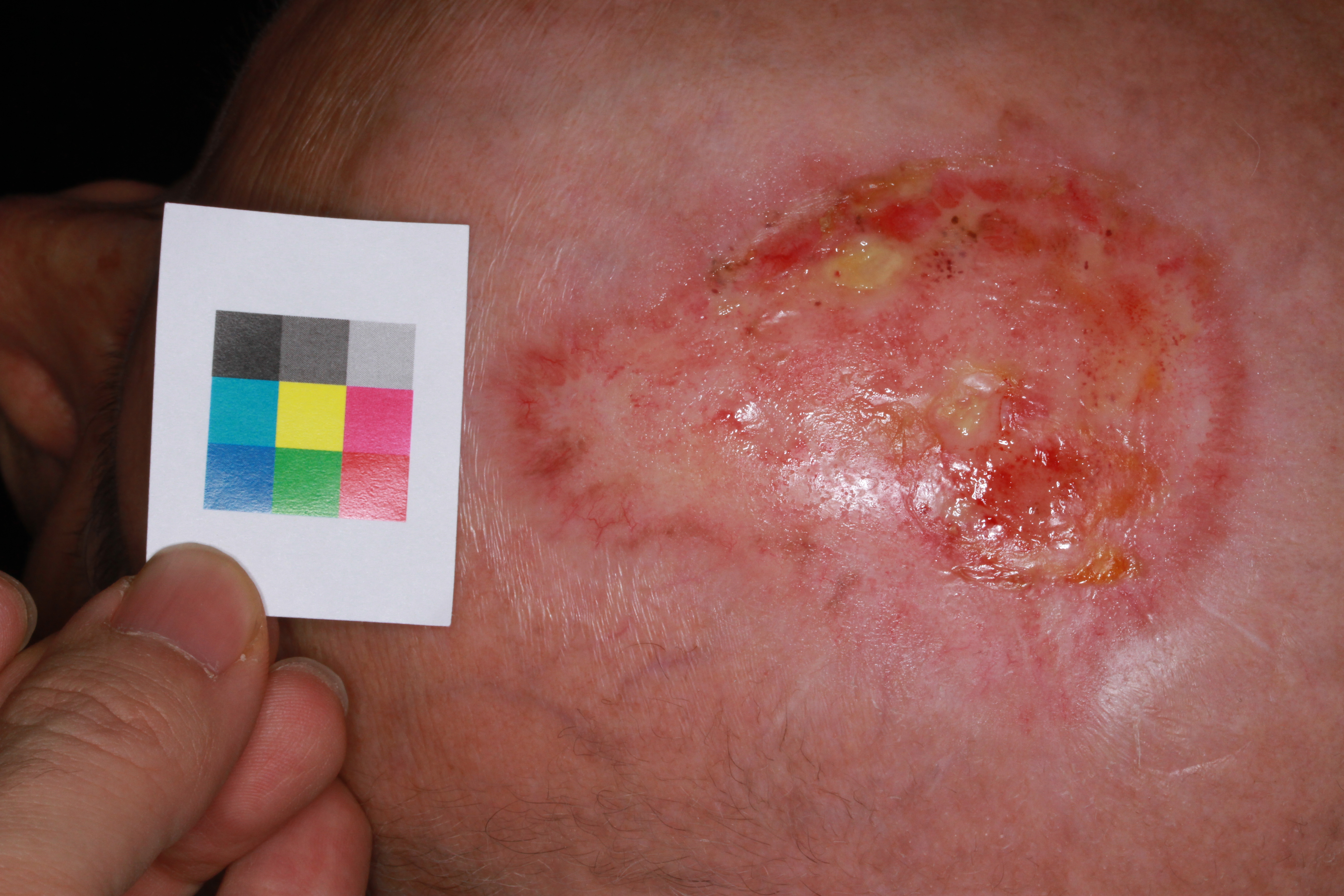

Supplement: S1 File — (ZIP) [file pone.0163092.s001.zip › 1218.jpg]

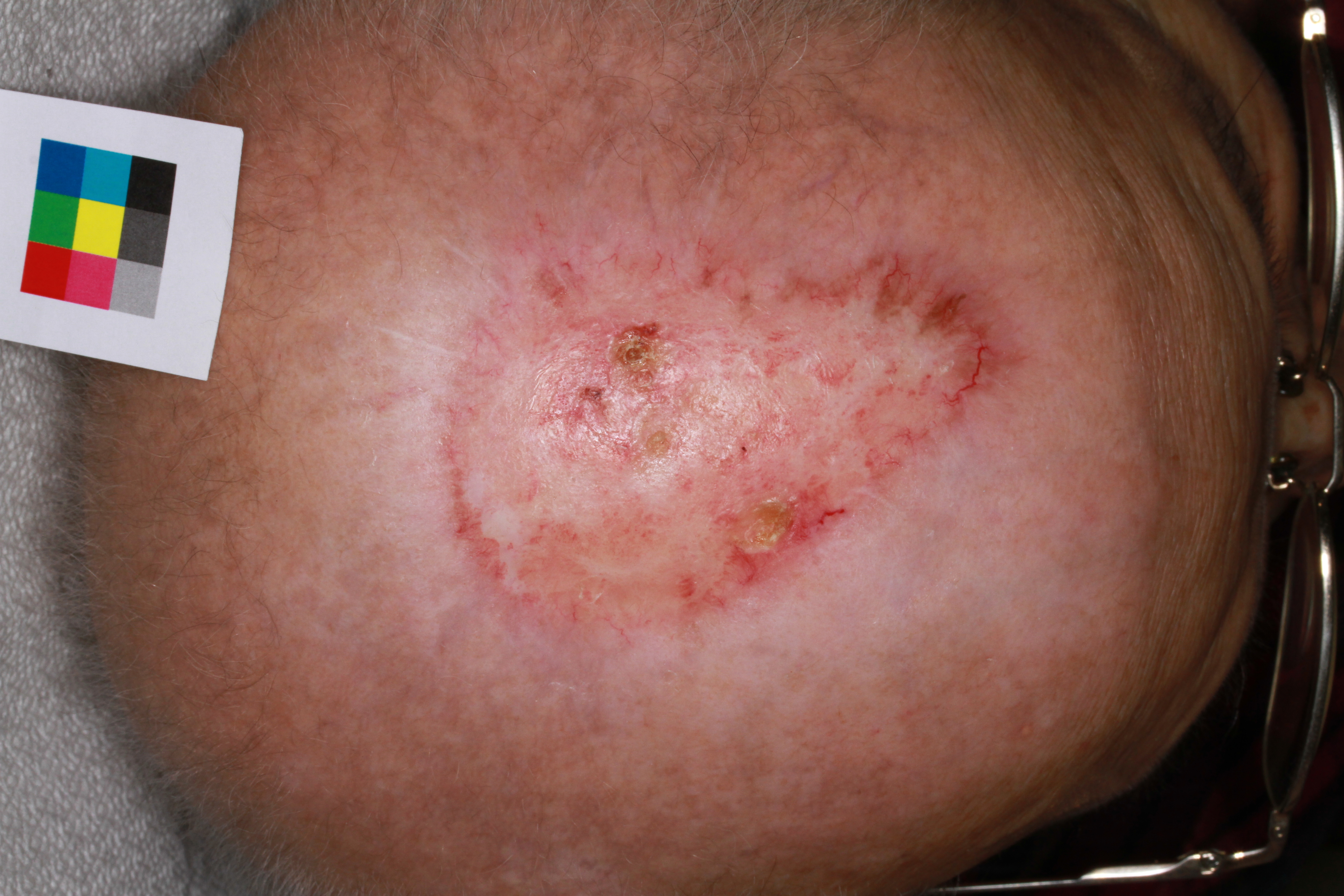

Supplement: S1 File — (ZIP) [file pone.0163092.s001.zip › 40225.JPG]

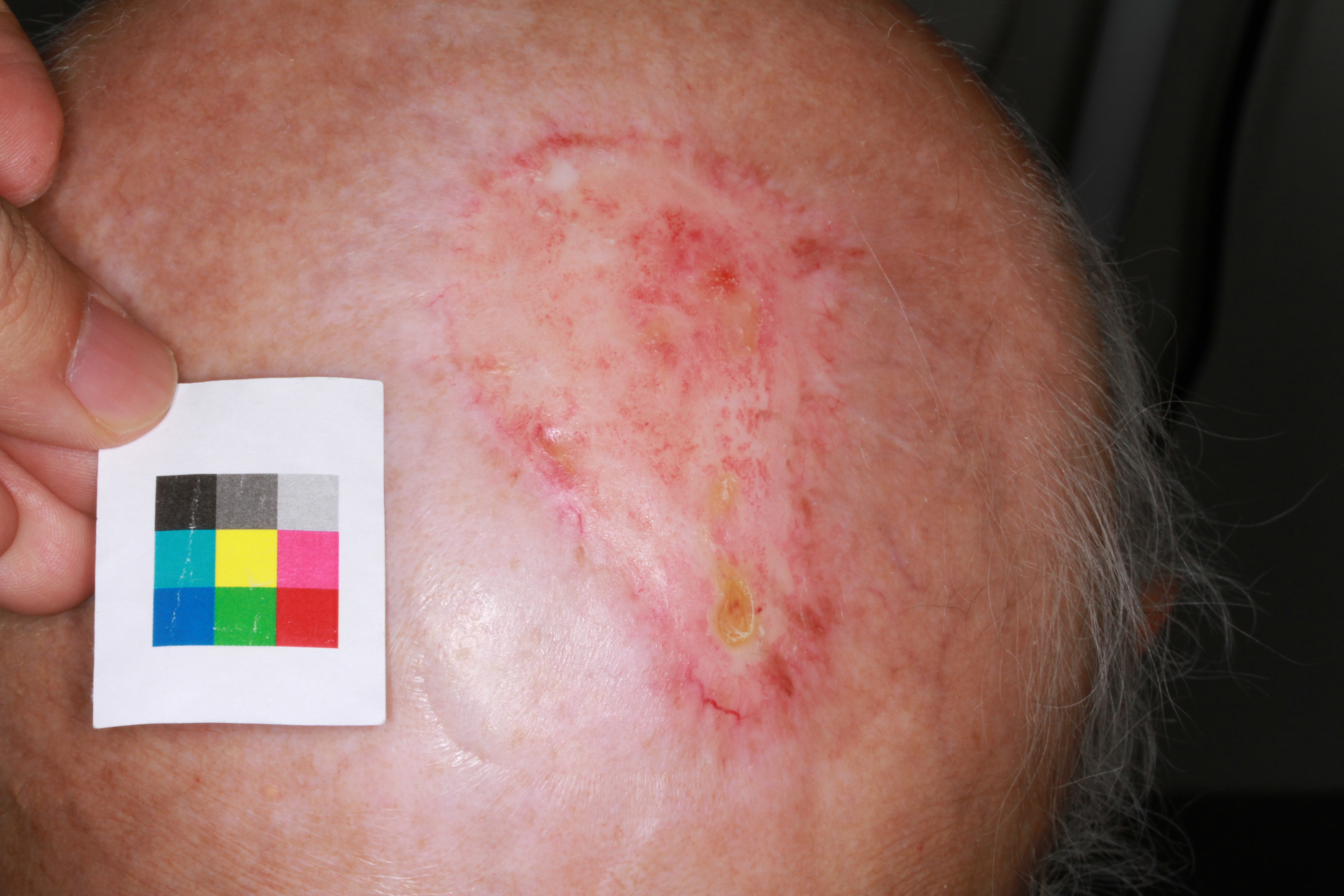

Supplement: S1 File — (ZIP) [file pone.0163092.s001.zip › 40521.JPG]

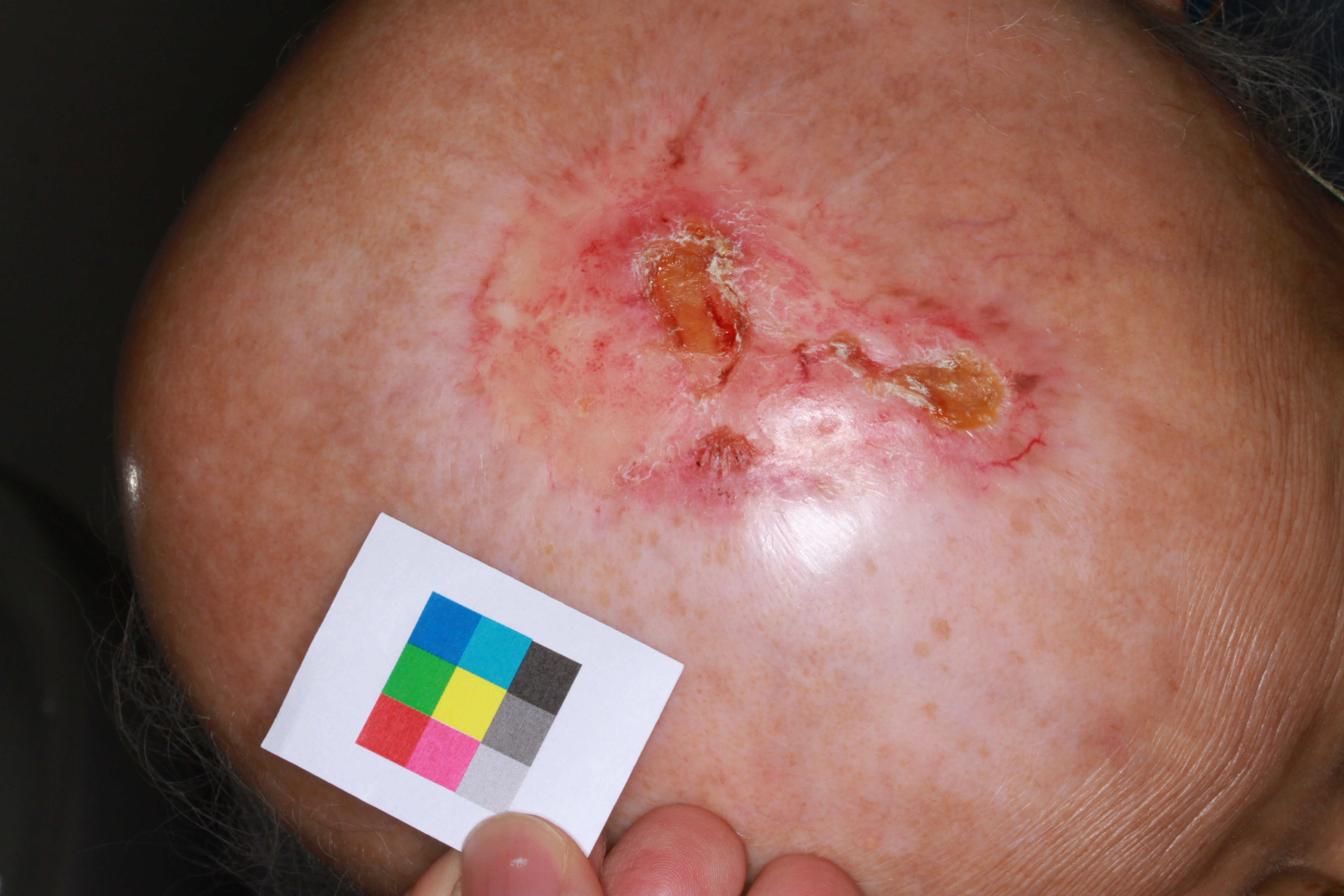

Supplement: S1 File — (ZIP) [file pone.0163092.s001.zip › 40820.JPG]

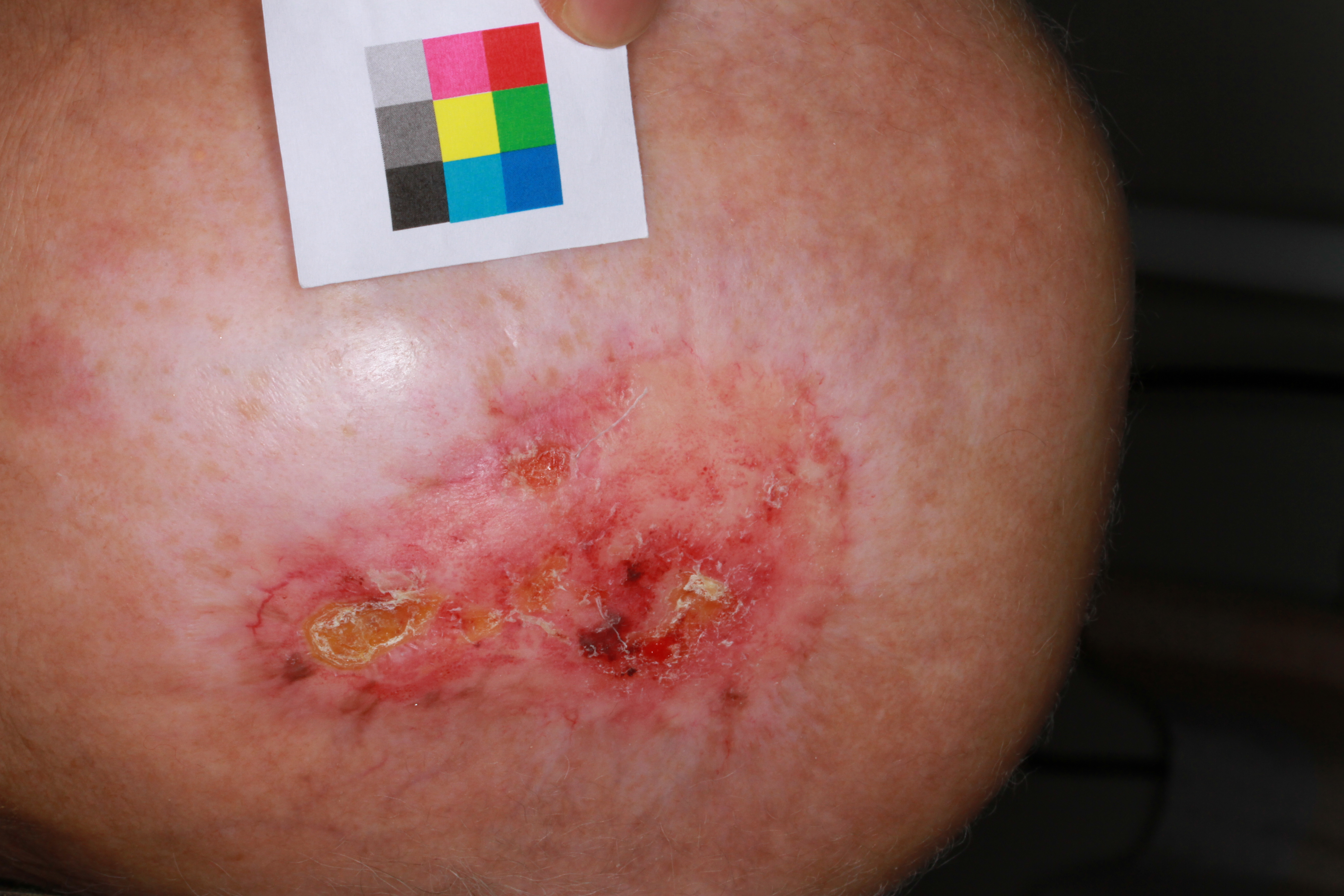

Supplement: S1 File — (ZIP) [file pone.0163092.s001.zip › 41112-1.JPG]

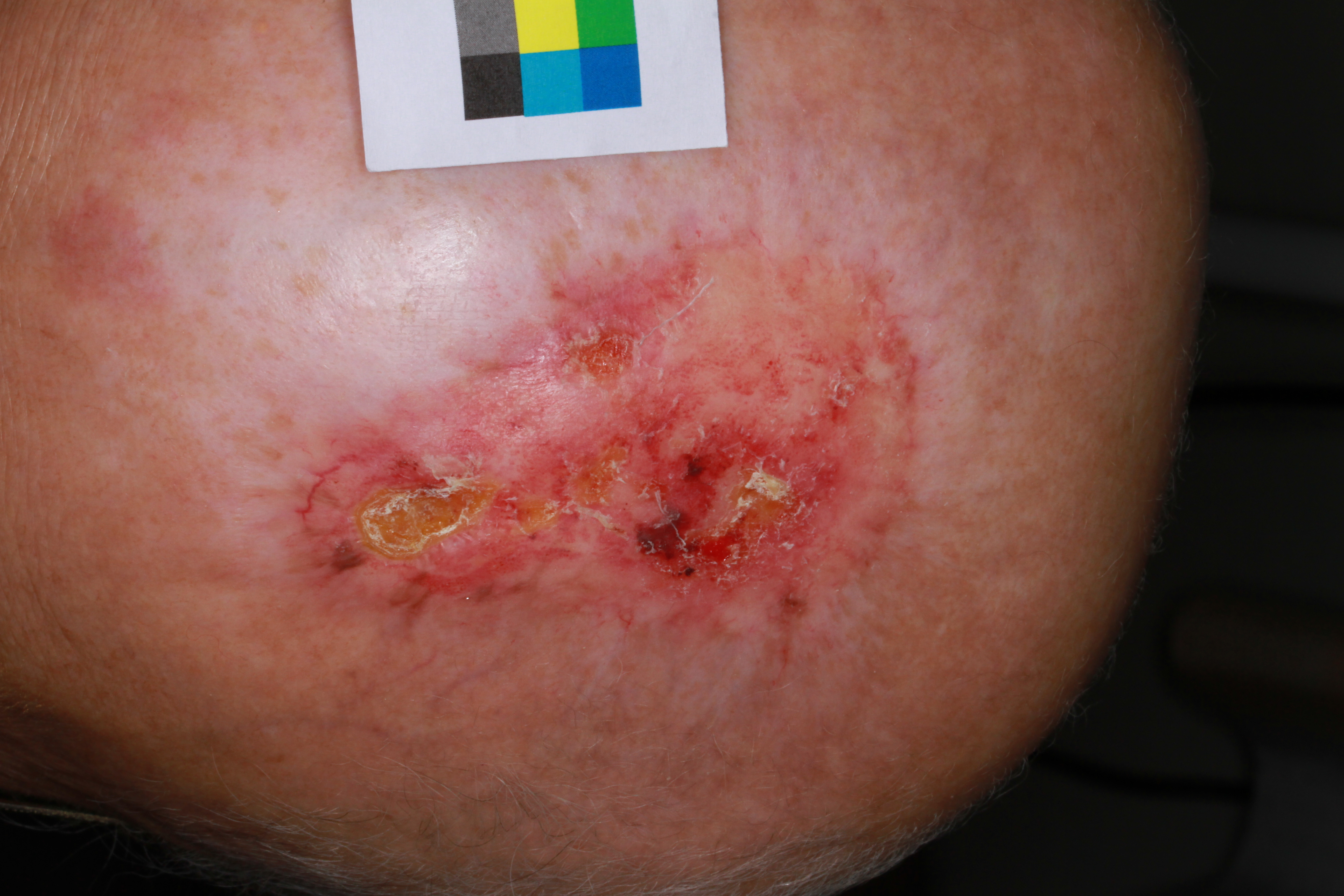

Supplement: S1 File — (ZIP) [file pone.0163092.s001.zip › 41112.JPG]

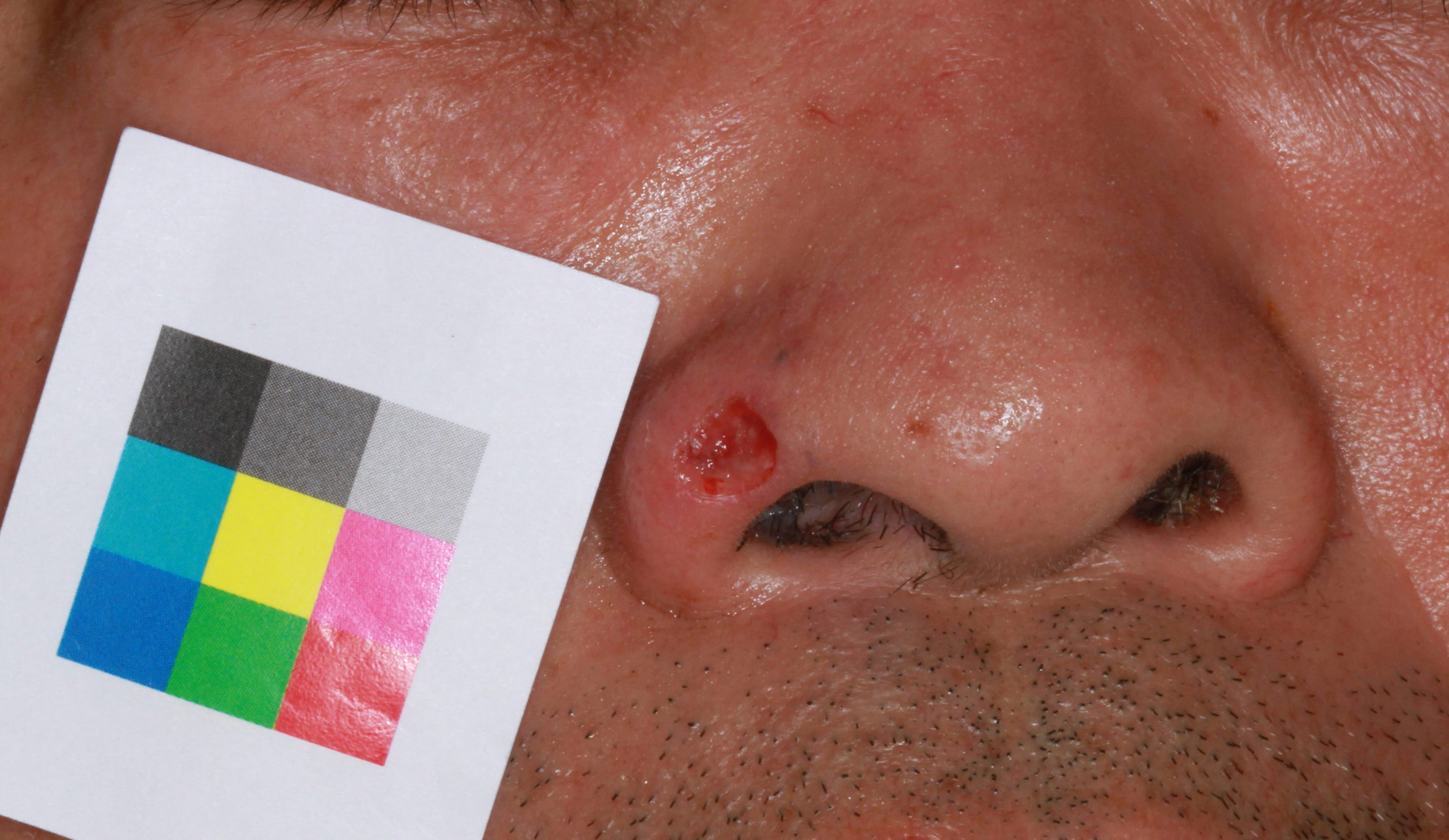

Supplement: S2 File — (ZIP) [file pone.0163092.s002.zip › 1120.jpg]

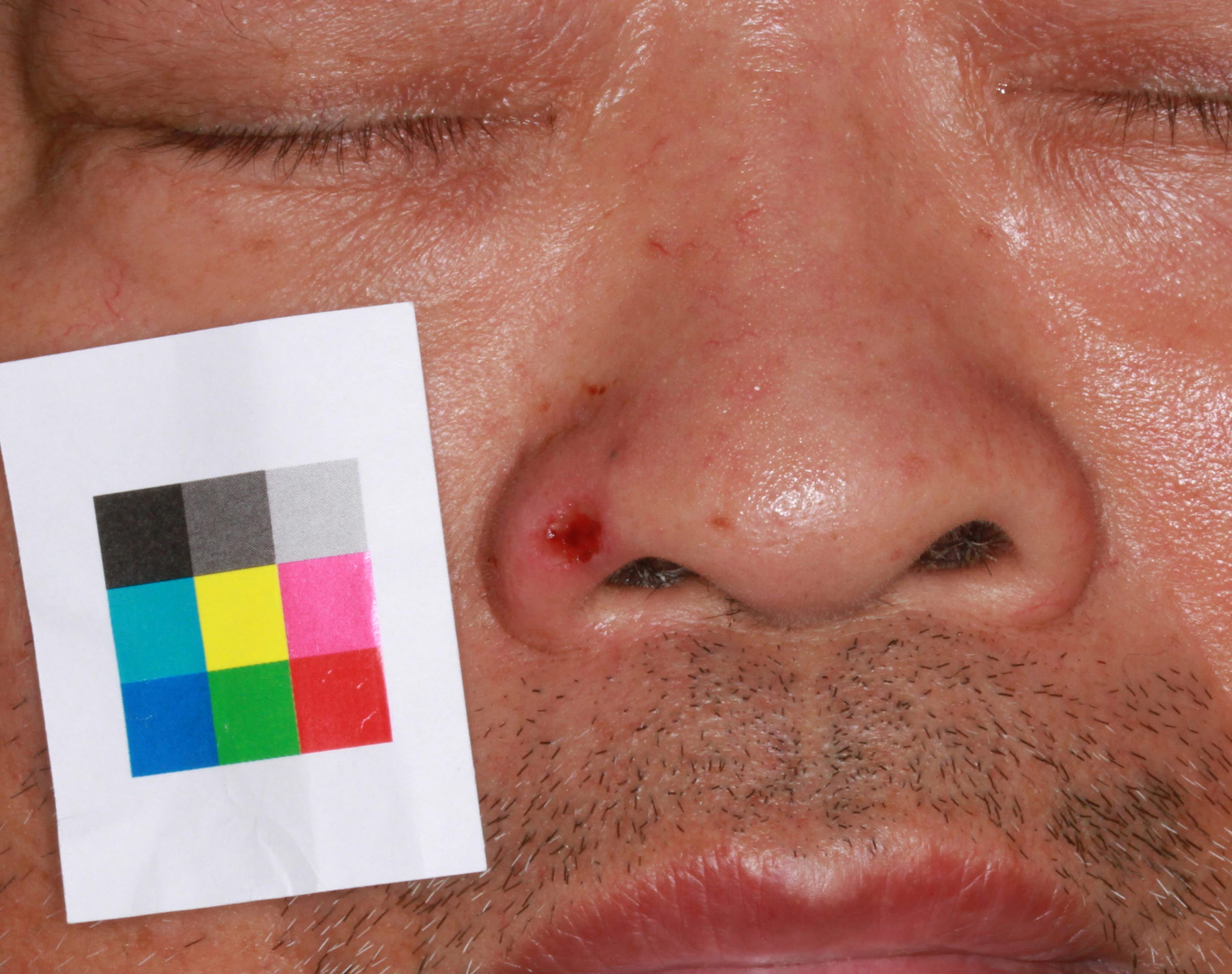

Supplement: S2 File — (ZIP) [file pone.0163092.s002.zip › 1122.jpg]

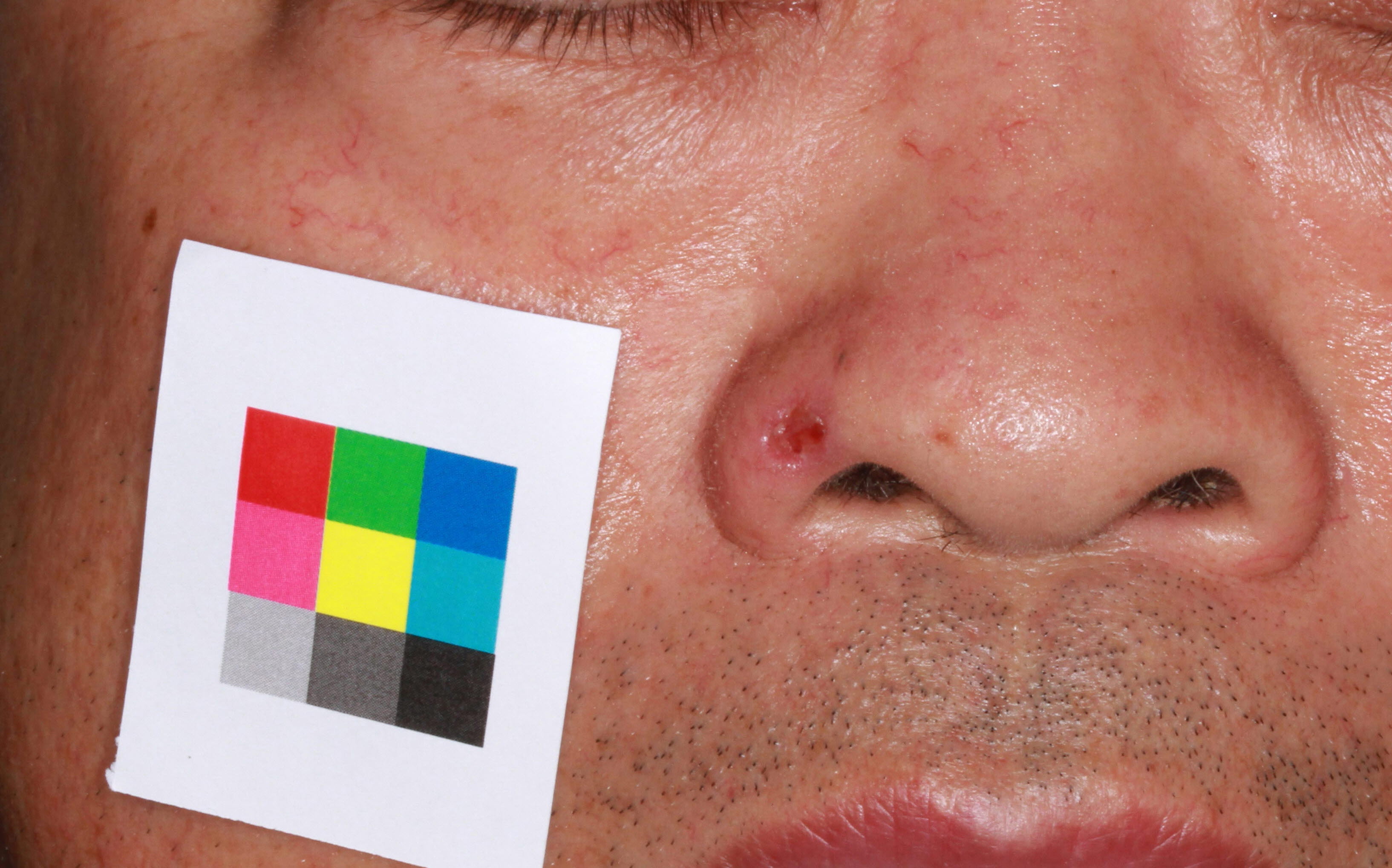

Supplement: S2 File — (ZIP) [file pone.0163092.s002.zip › 1127.jpg]

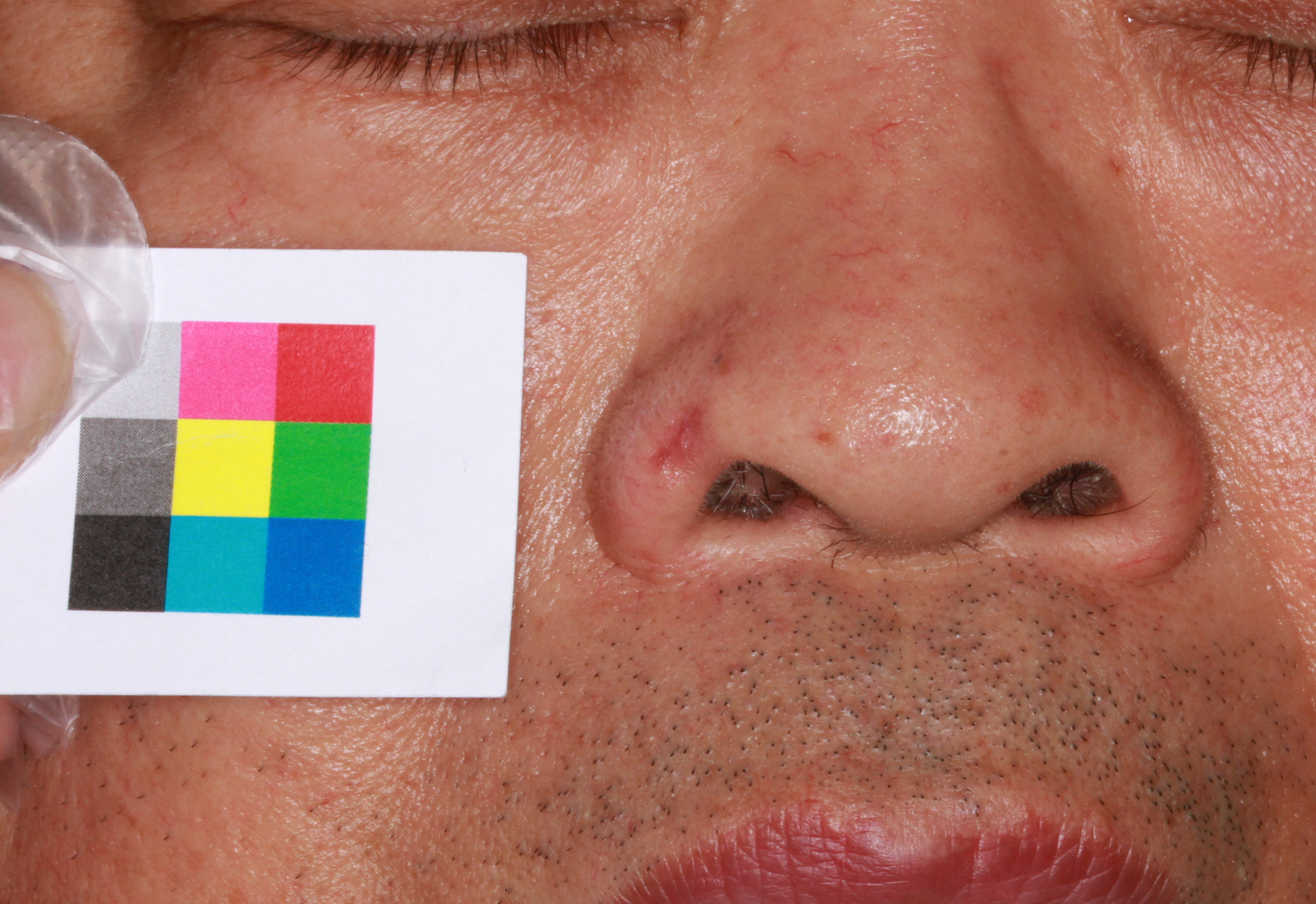

Supplement: S2 File — (ZIP) [file pone.0163092.s002.zip › 1226.jpg]

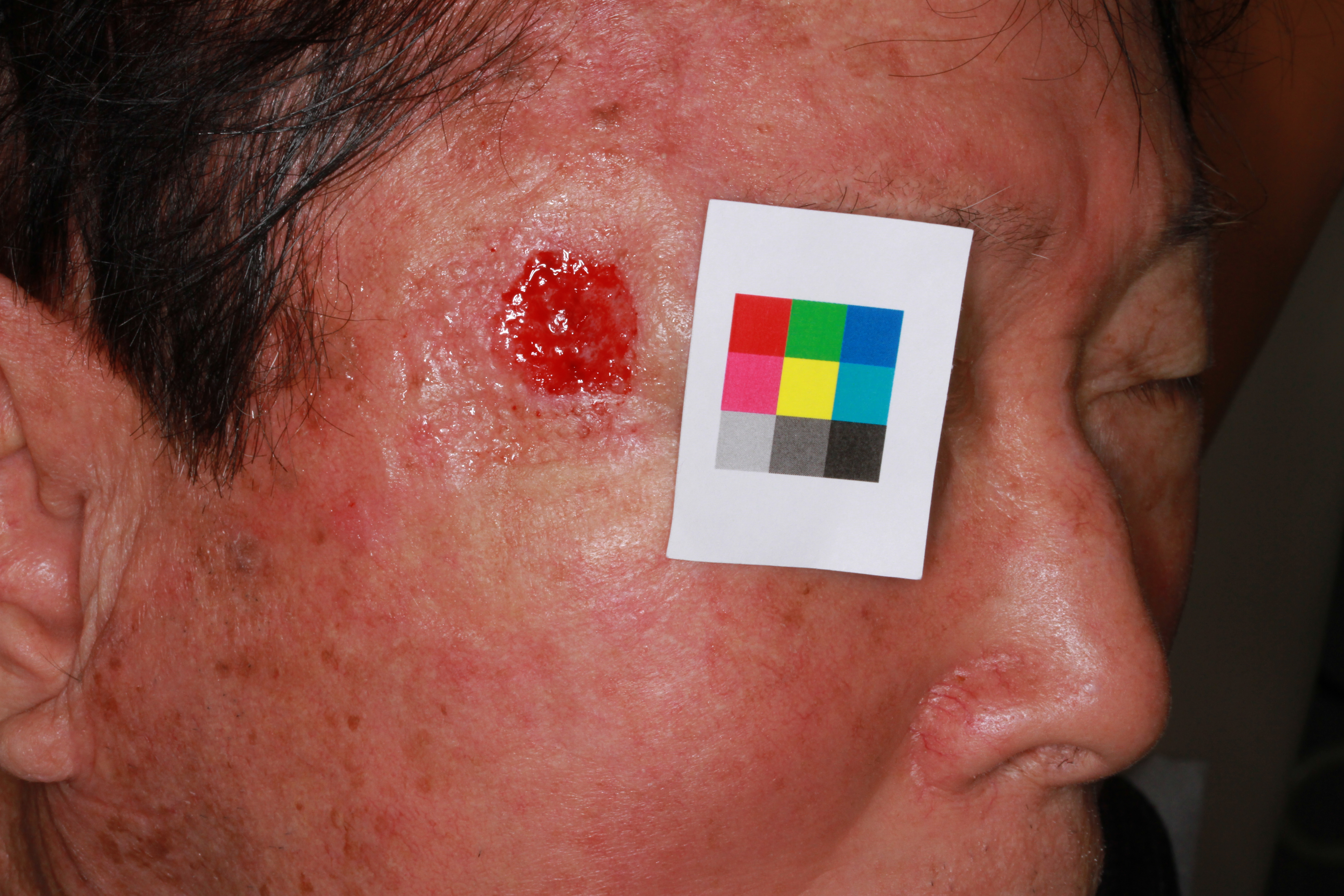

Supplement: S3 File — (ZIP) [file pone.0163092.s003.zip › 31113.JPG]

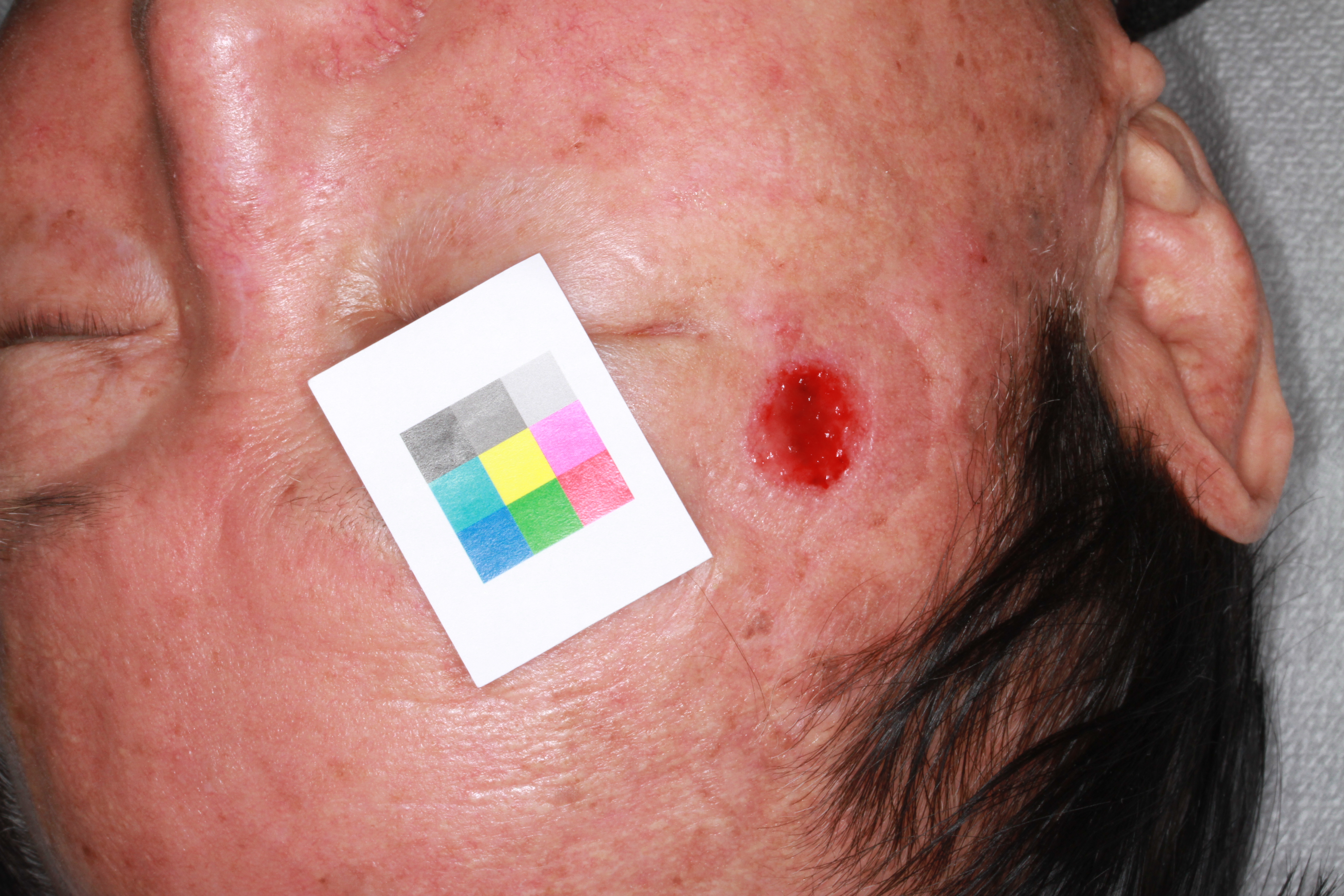

Supplement: S3 File — (ZIP) [file pone.0163092.s003.zip › 31115.JPG]

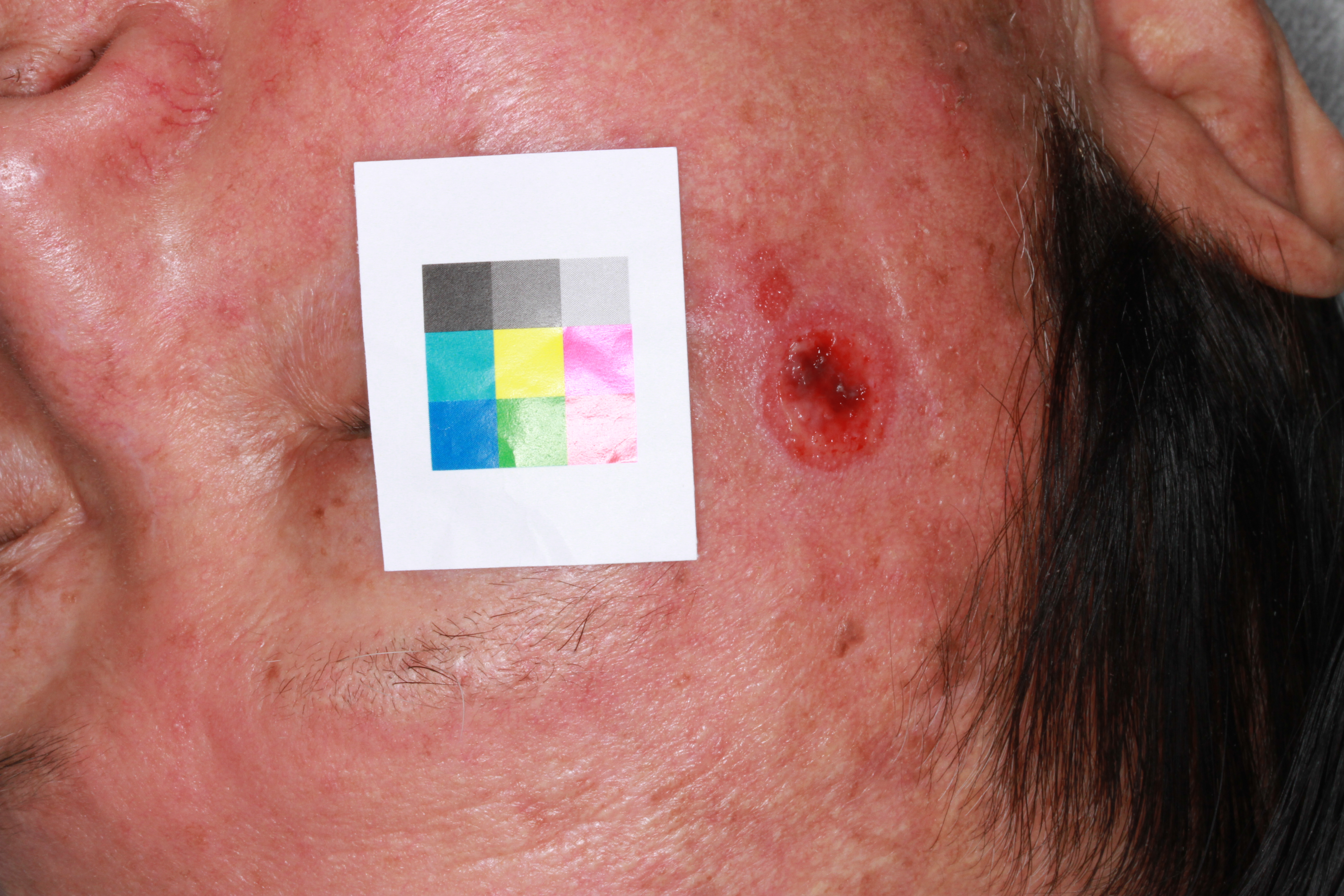

Supplement: S3 File — (ZIP) [file pone.0163092.s003.zip › 31119.JPG]

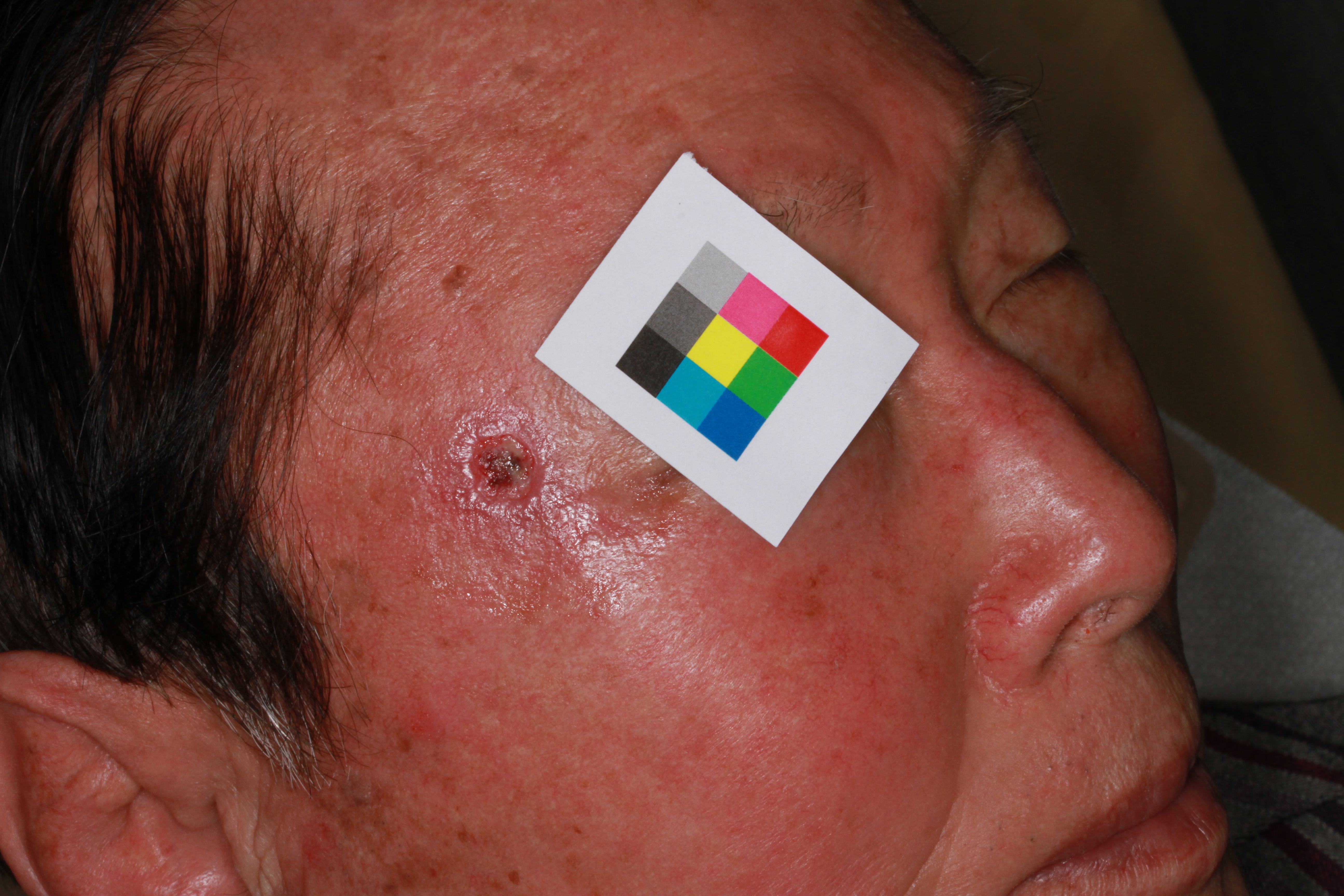

Supplement: S3 File — (ZIP) [file pone.0163092.s003.zip › 31127.JPG]

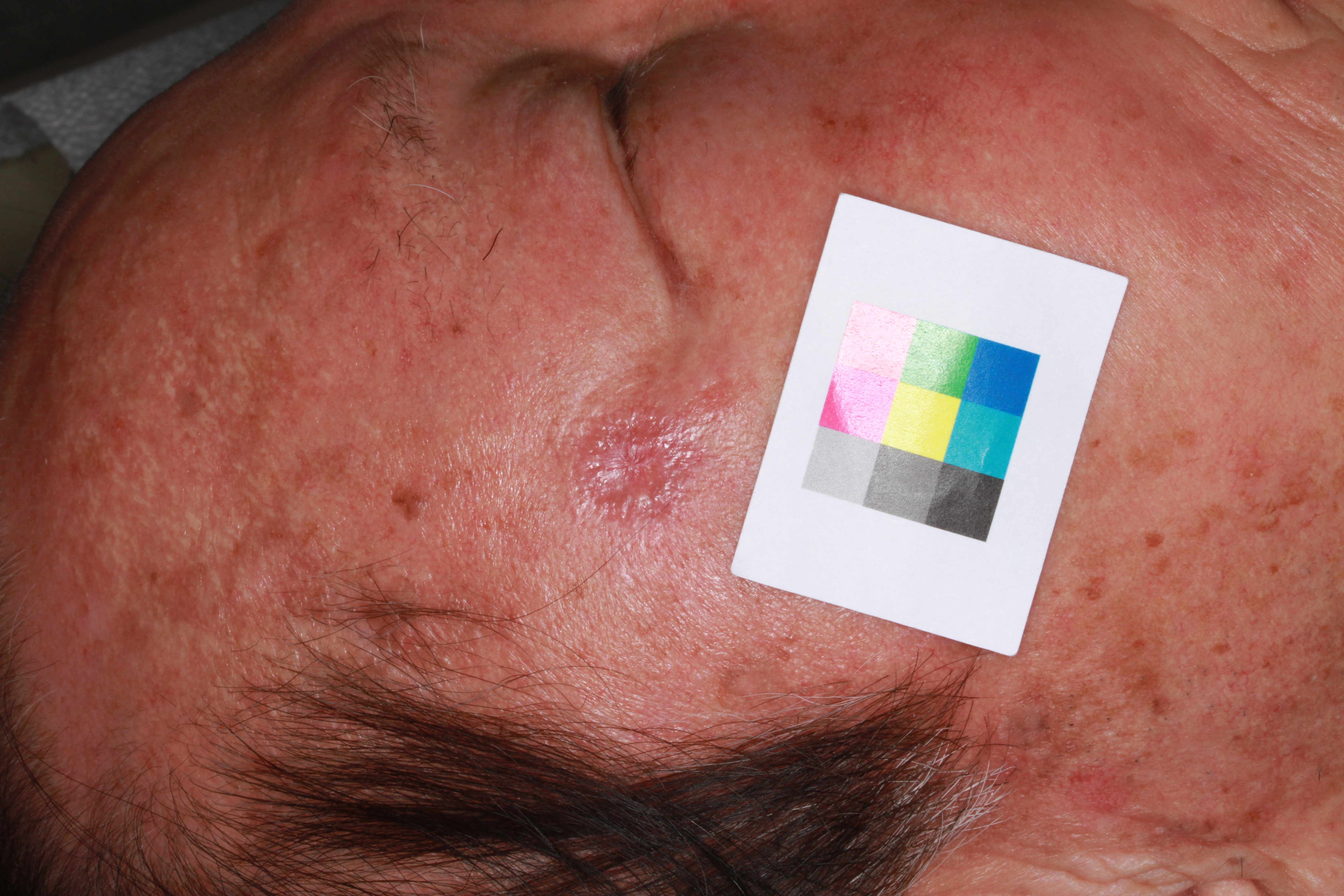

Supplement: S3 File — (ZIP) [file pone.0163092.s003.zip › 31210.JPG]

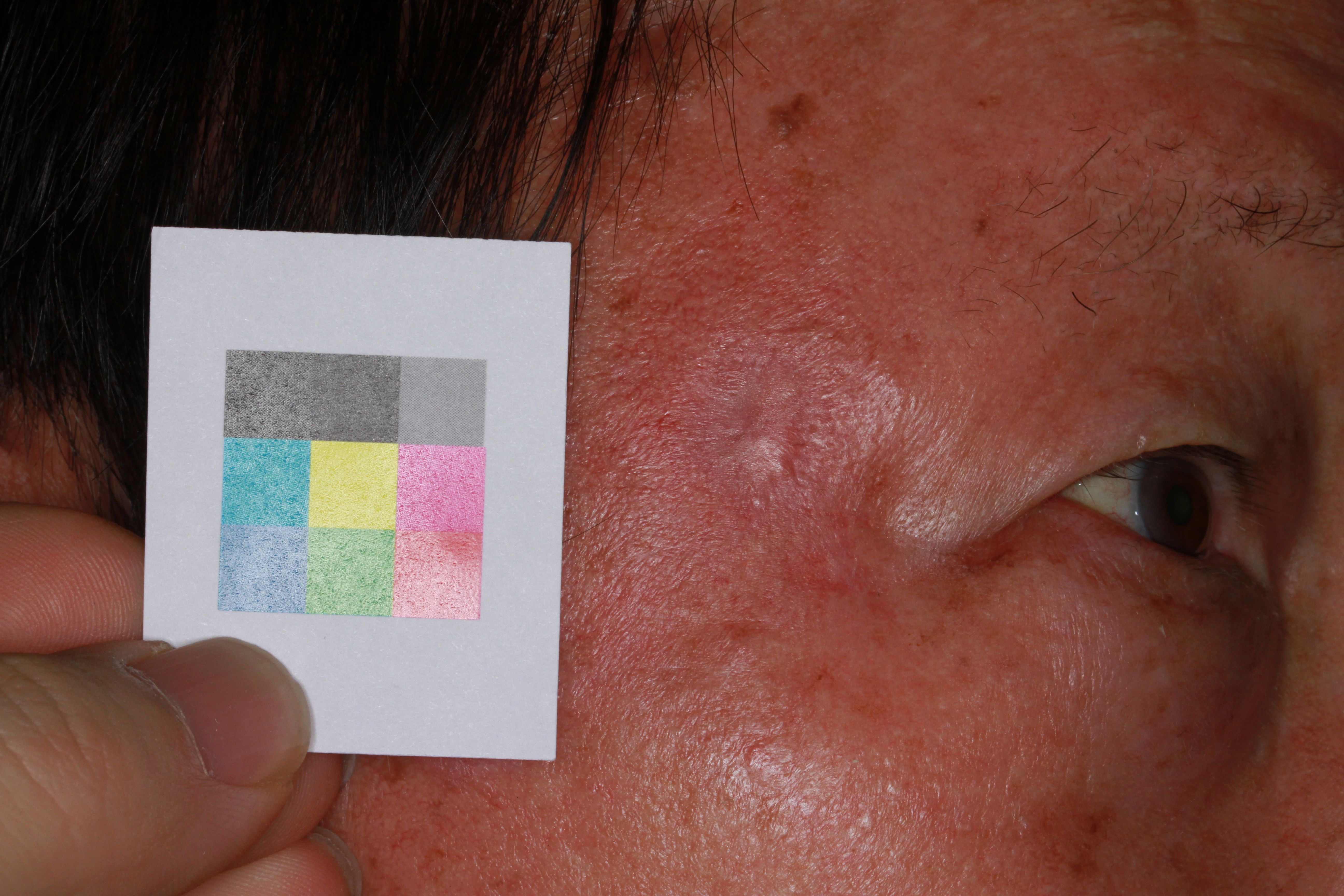

Supplement: S3 File — (ZIP) [file pone.0163092.s003.zip › 40108.JPG]

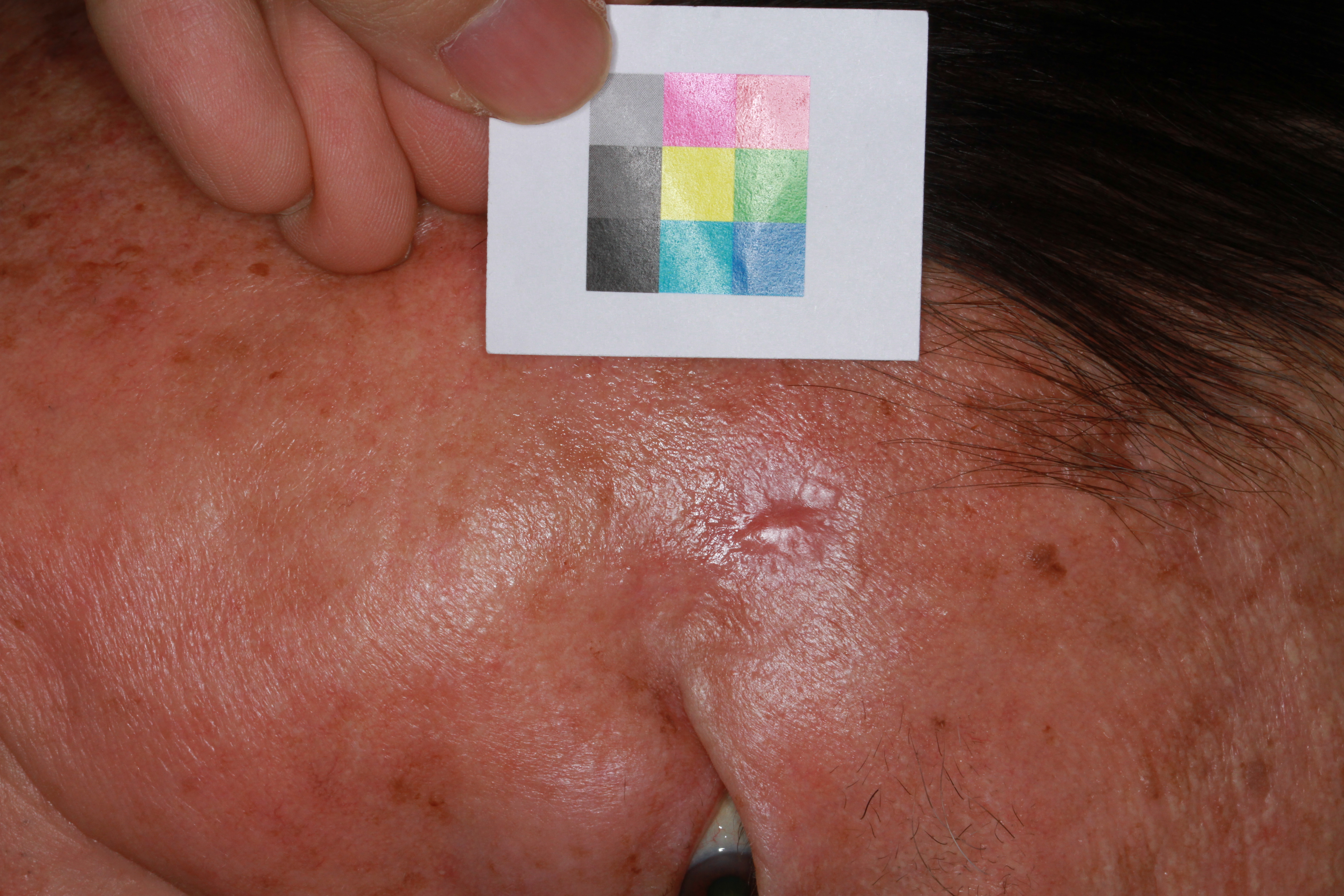

Supplement: S3 File — (ZIP) [file pone.0163092.s003.zip › 40207.JPG]

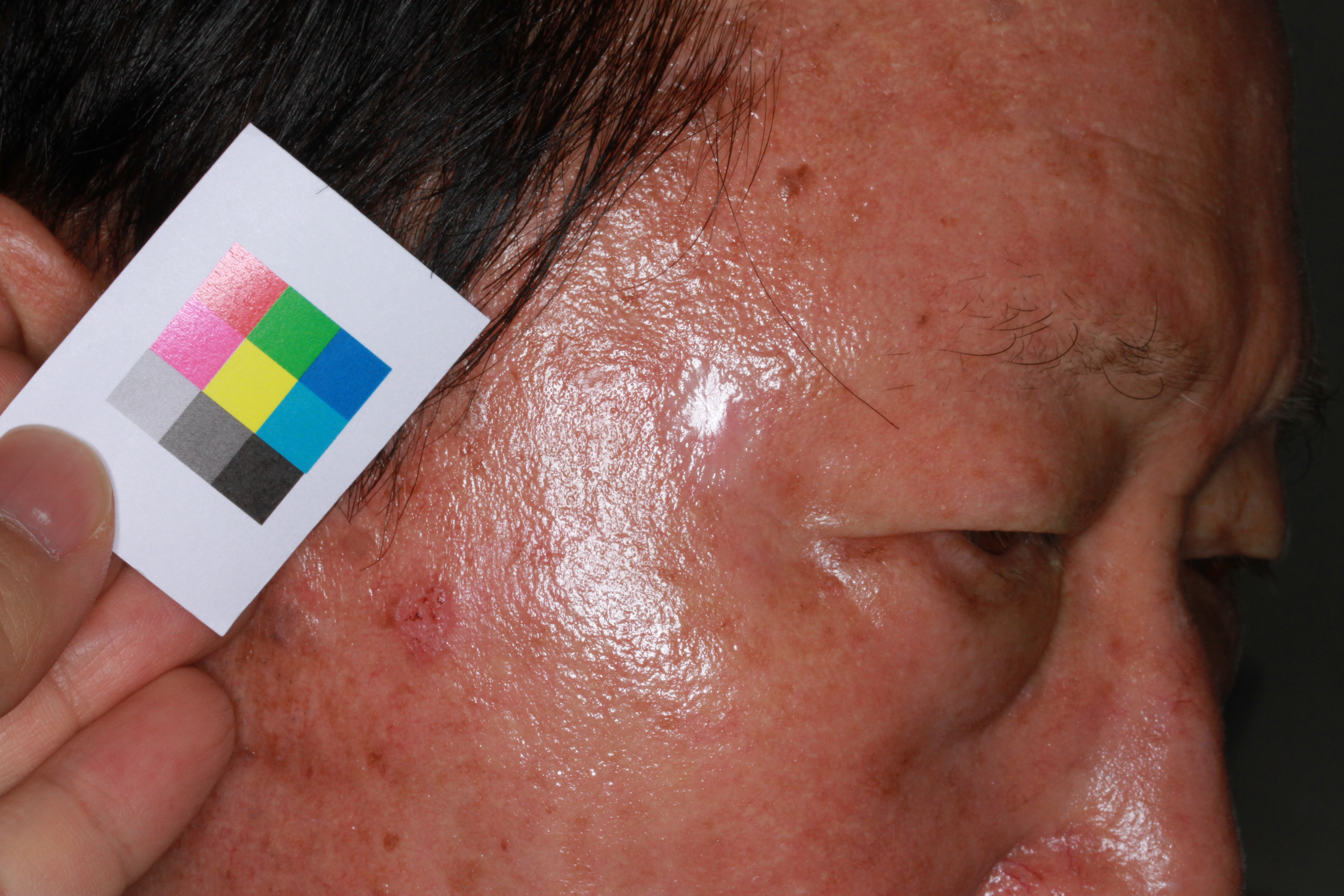

Supplement: S3 File — (ZIP) [file pone.0163092.s003.zip › 40711.JPG]

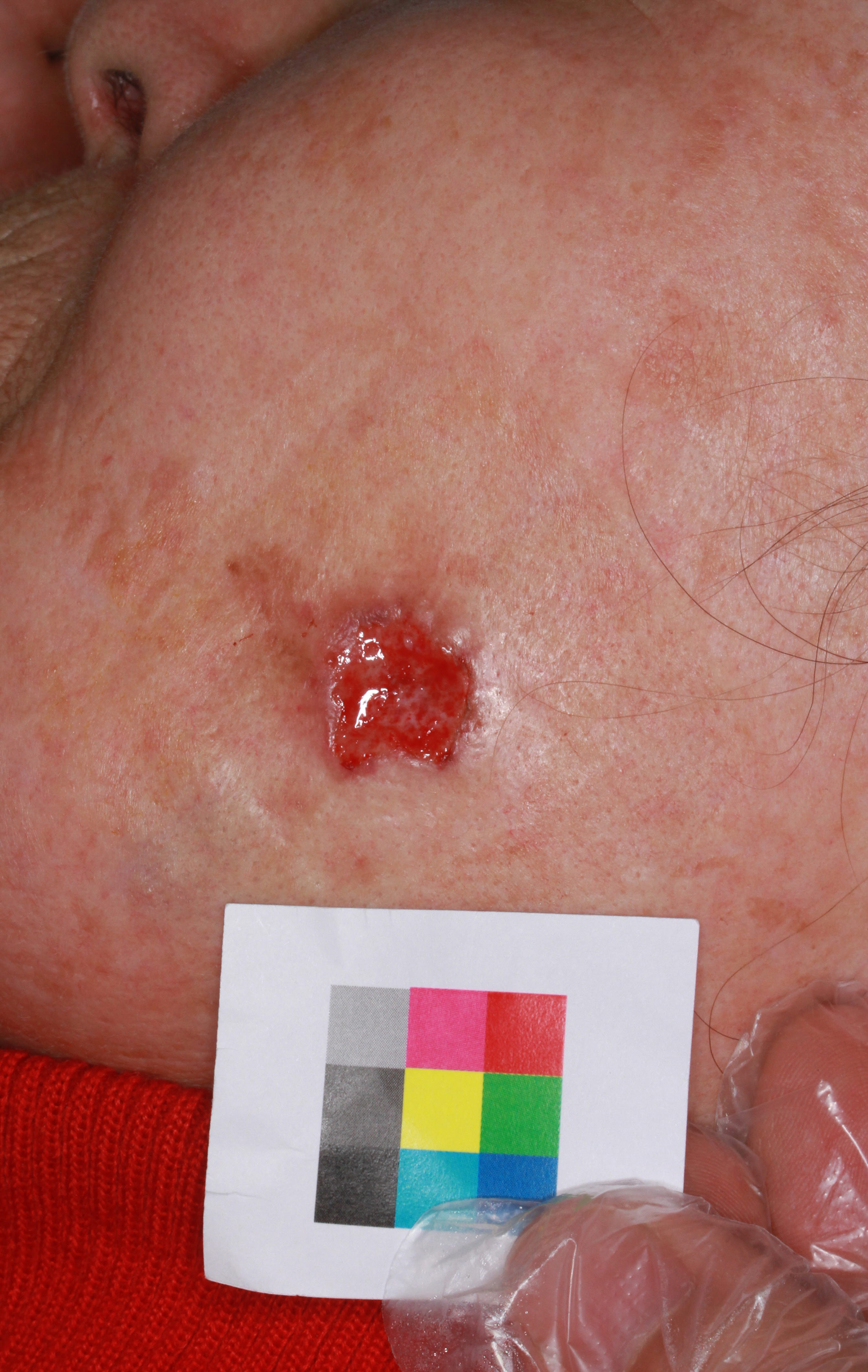

Supplement: S4 File — (ZIP) [file pone.0163092.s004.zip › 0219.jpg]

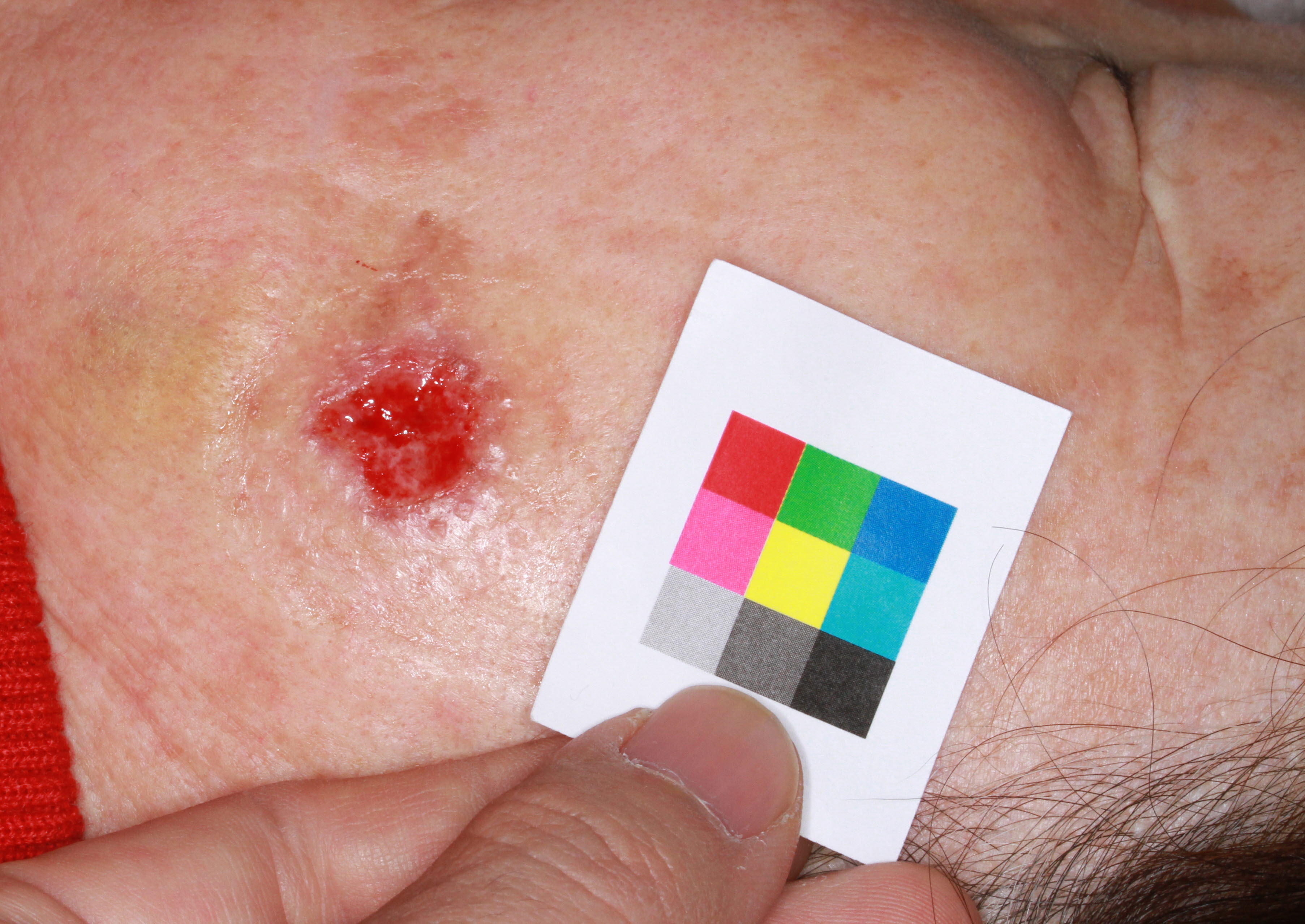

Supplement: S4 File — (ZIP) [file pone.0163092.s004.zip › 0221.jpg]

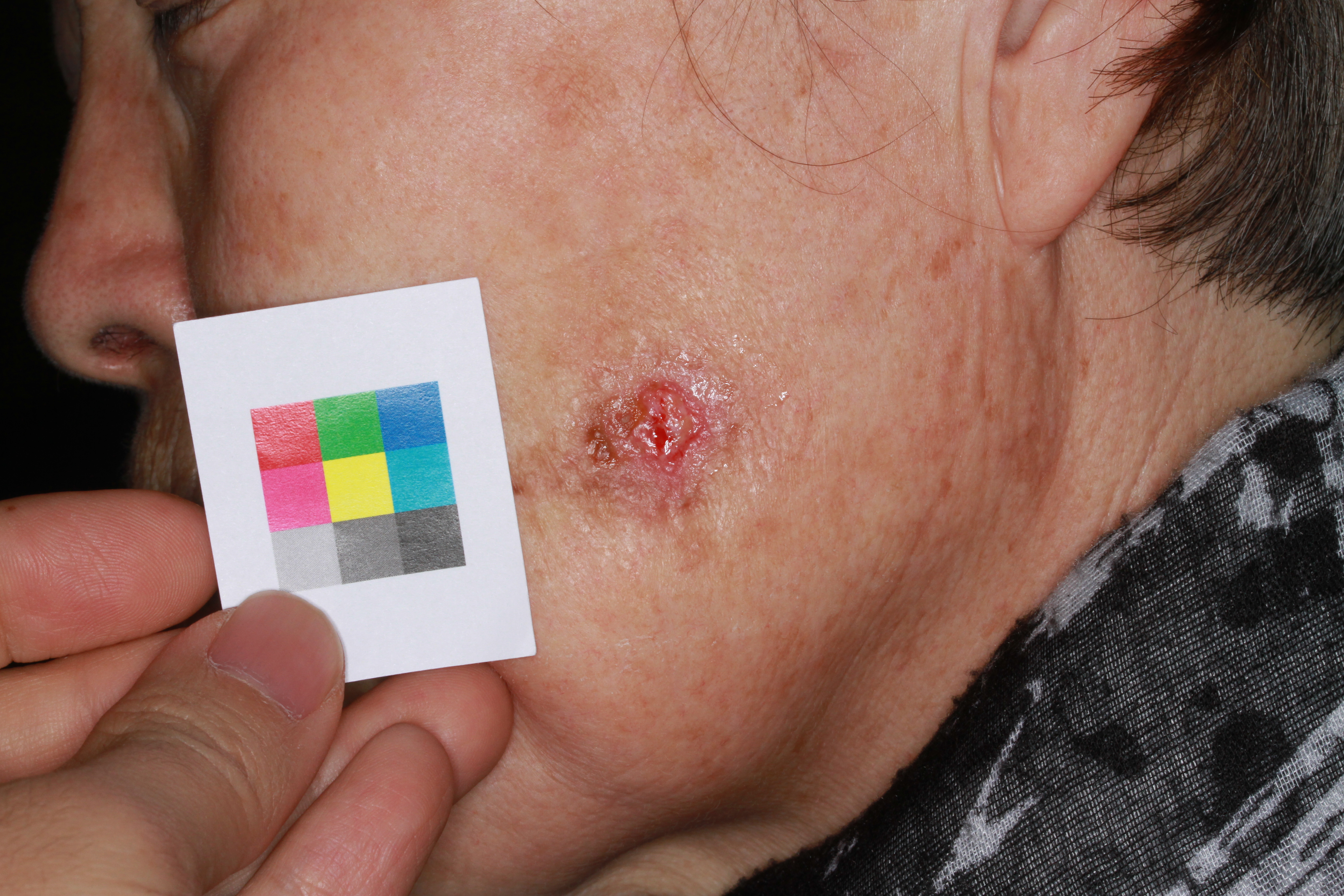

Supplement: S4 File — (ZIP) [file pone.0163092.s004.zip › 0307.JPG]

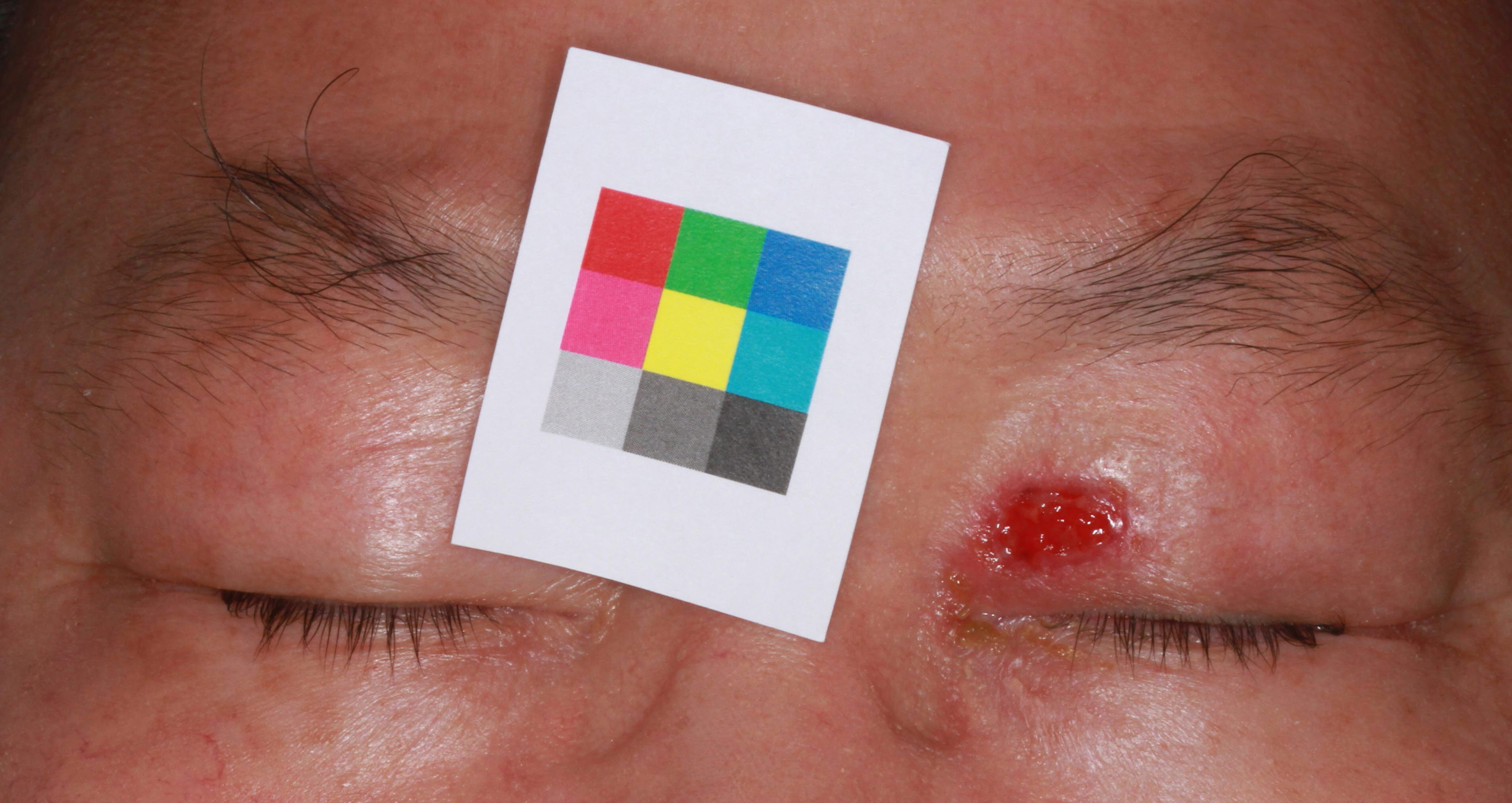

Supplement: S5 File — (ZIP) [file pone.0163092.s005.zip › 31212.jpg]

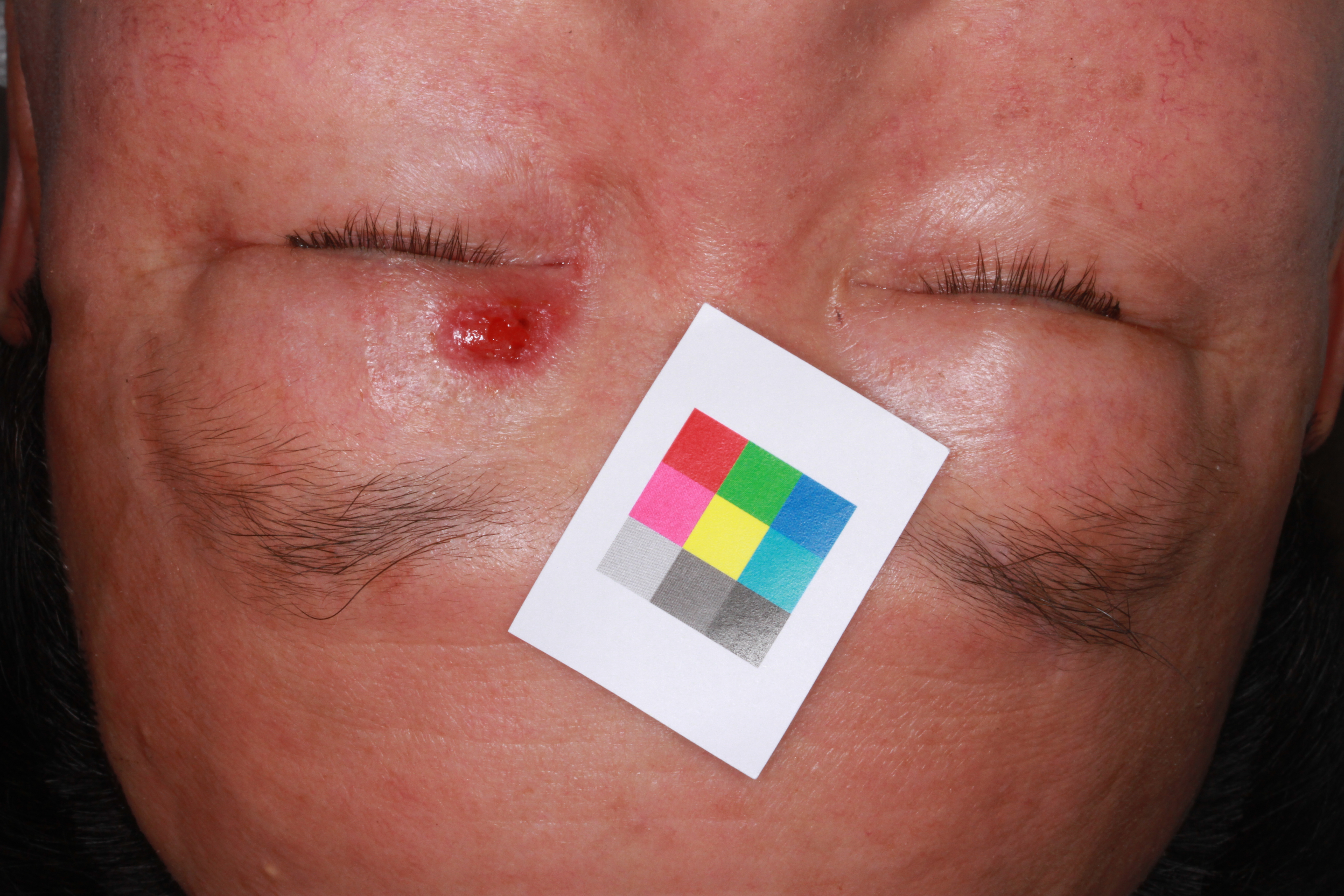

Supplement: S5 File — (ZIP) [file pone.0163092.s005.zip › 31214.JPG]

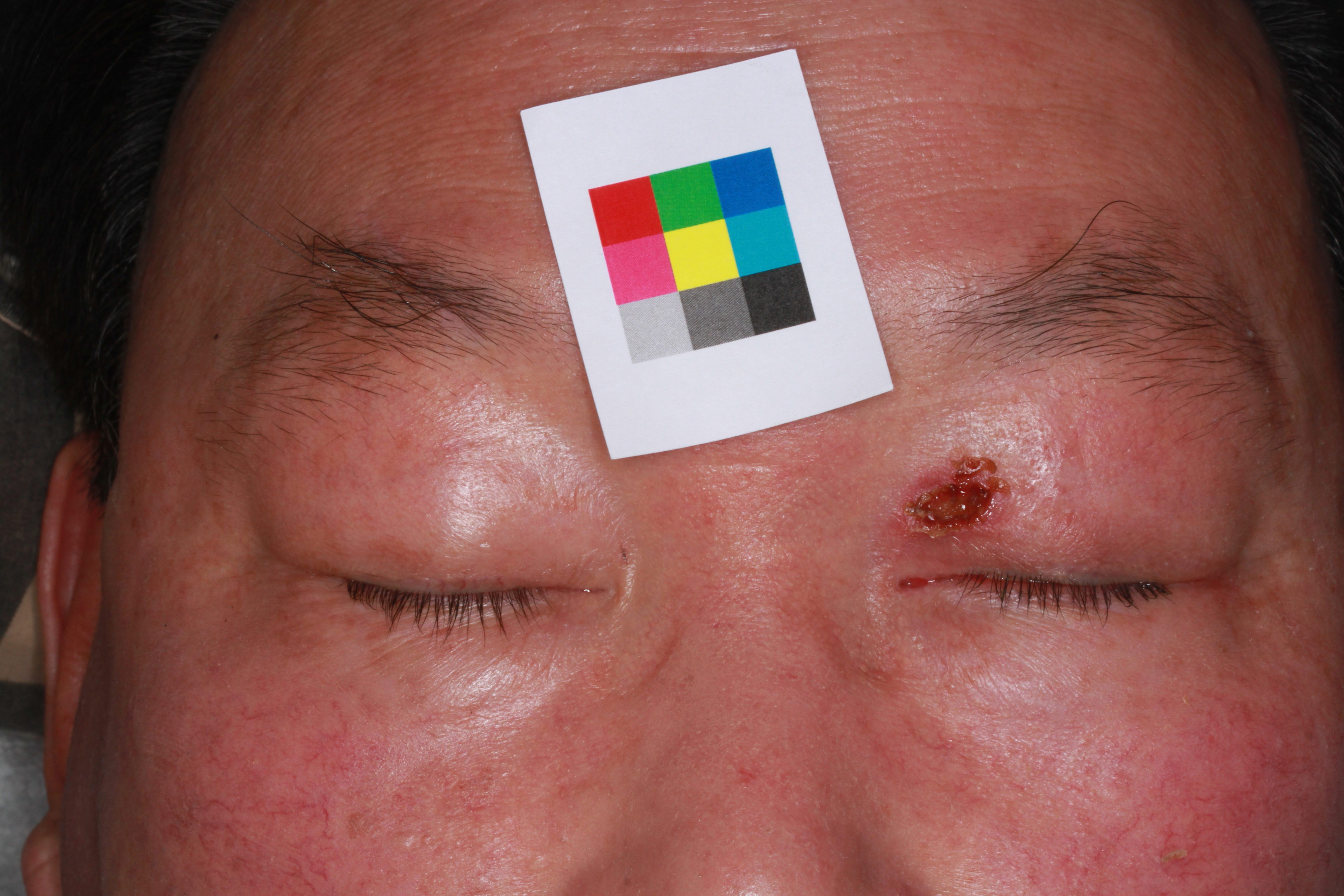

Supplement: S5 File — (ZIP) [file pone.0163092.s005.zip › 31217.JPG]

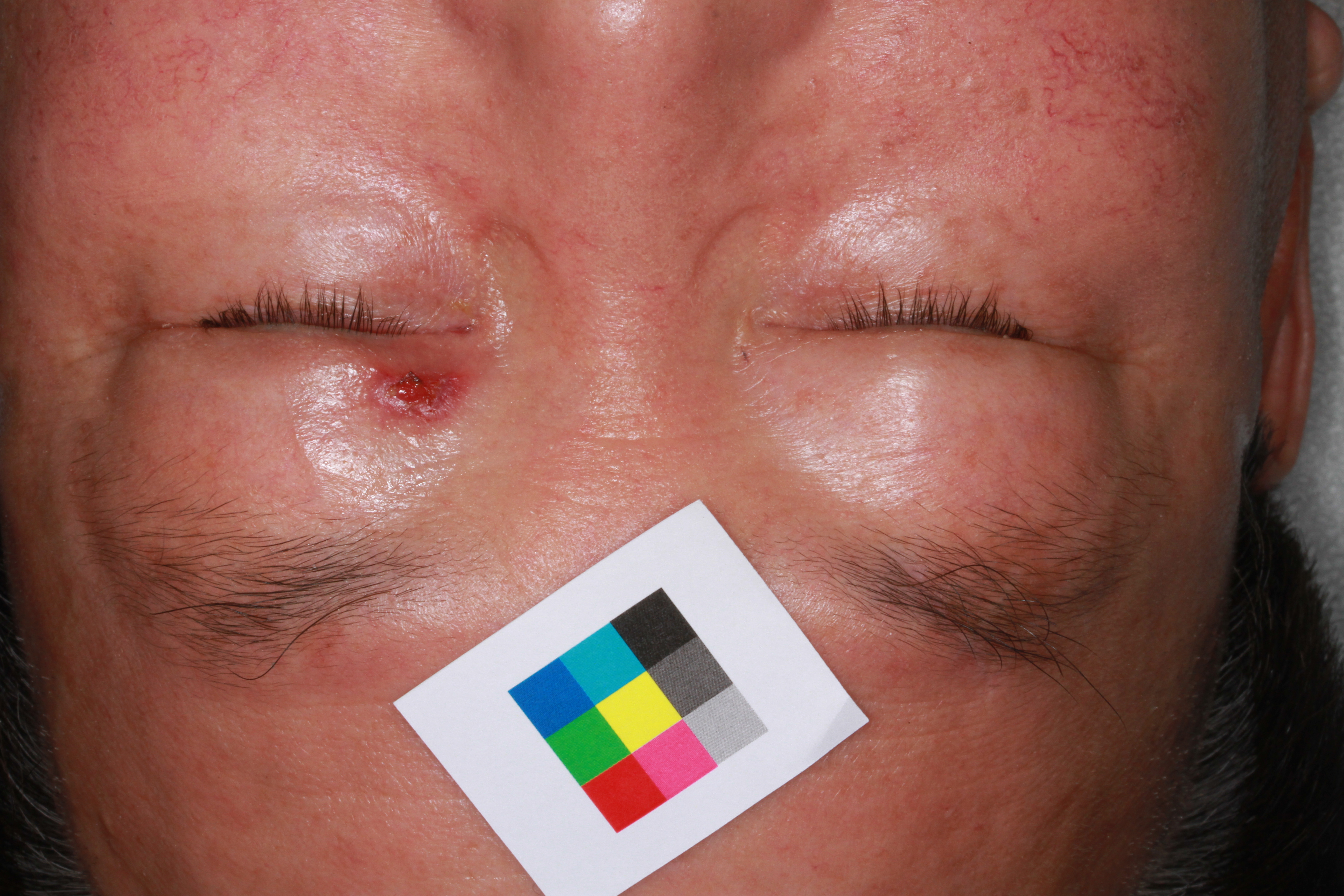

Supplement: S5 File — (ZIP) [file pone.0163092.s005.zip › 31221.JPG]

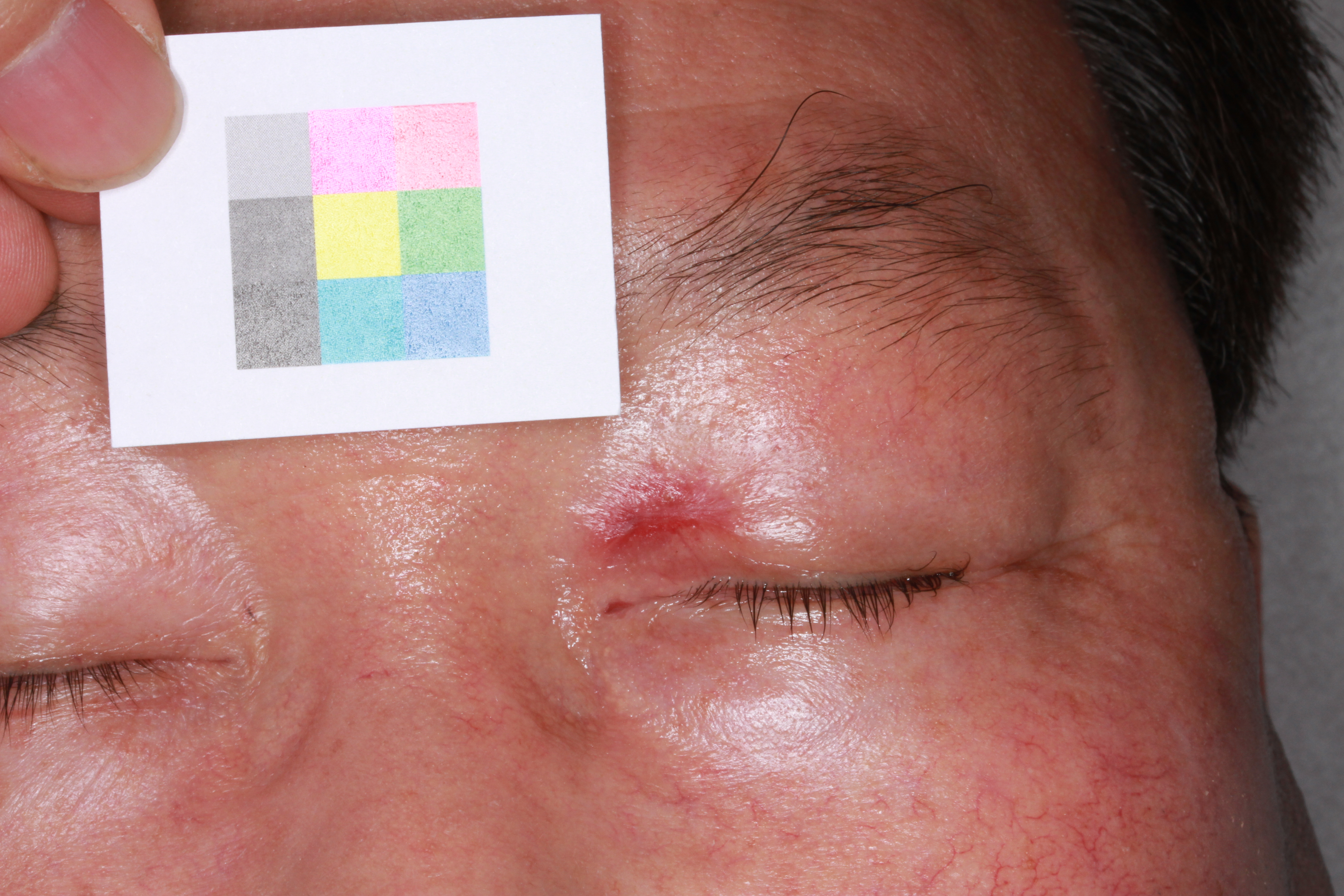

Supplement: S5 File — (ZIP) [file pone.0163092.s005.zip › 31228.JPG]

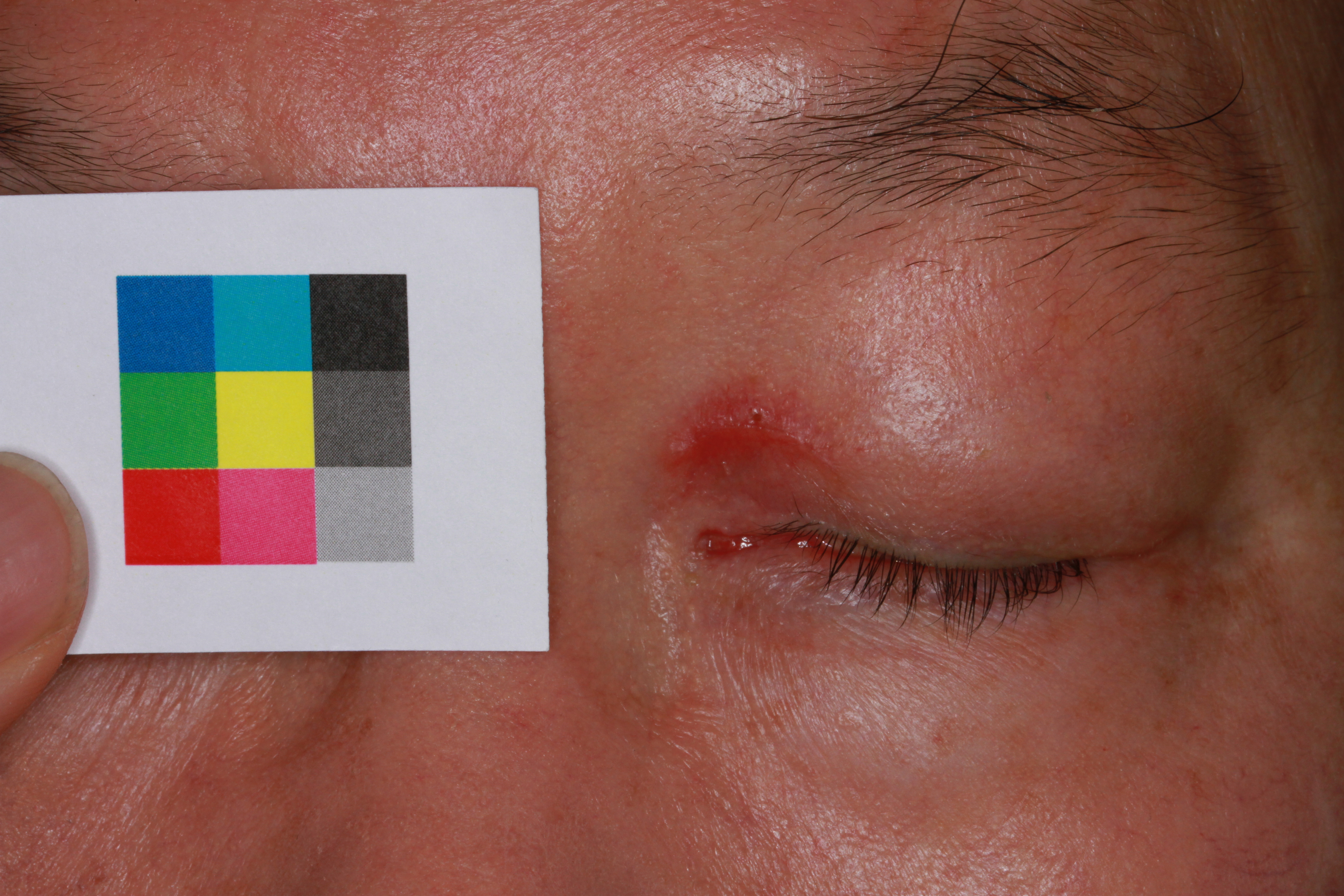

Supplement: S5 File — (ZIP) [file pone.0163092.s005.zip › 40110.JPG]

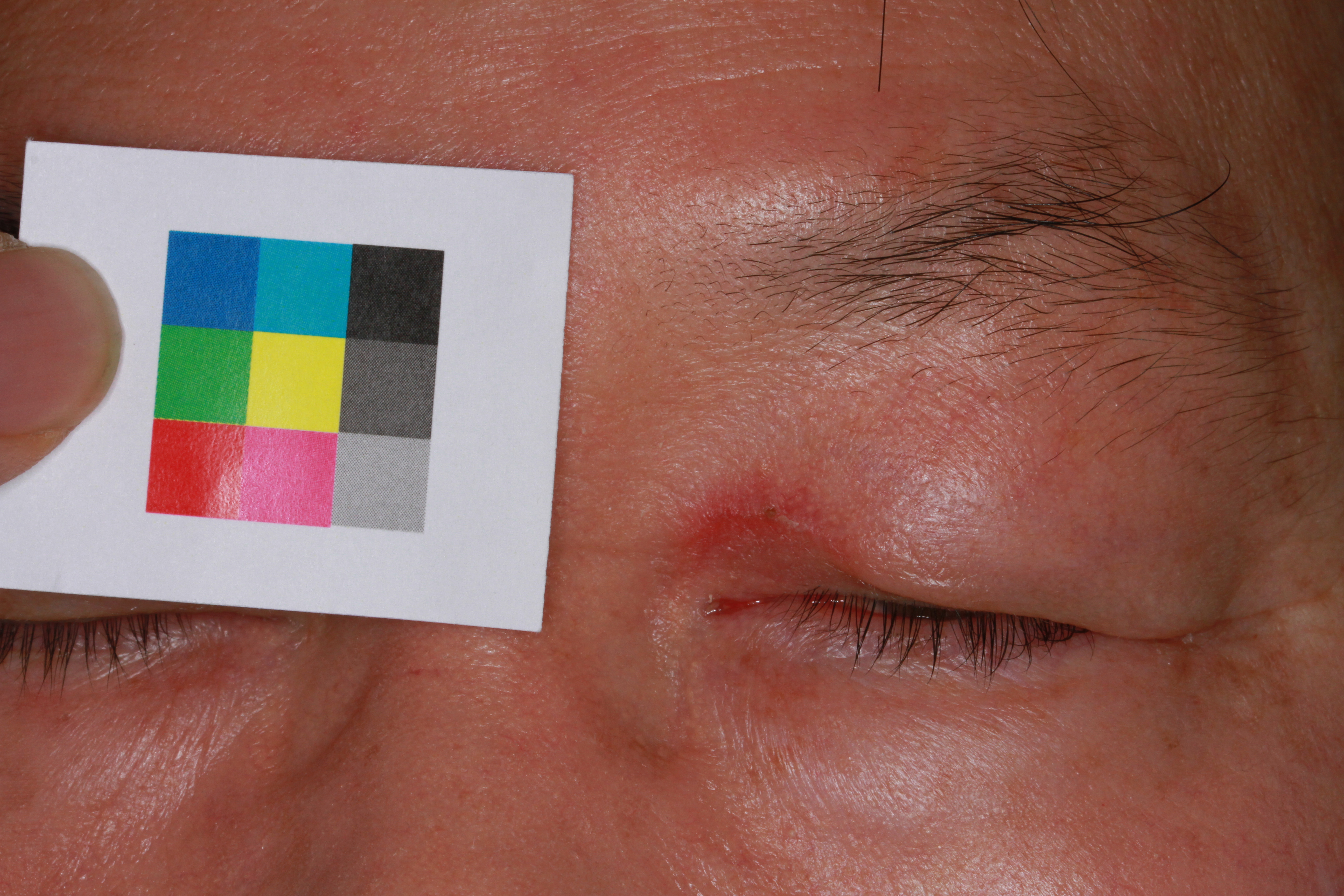

Supplement: S5 File — (ZIP) [file pone.0163092.s005.zip › 40124.JPG]

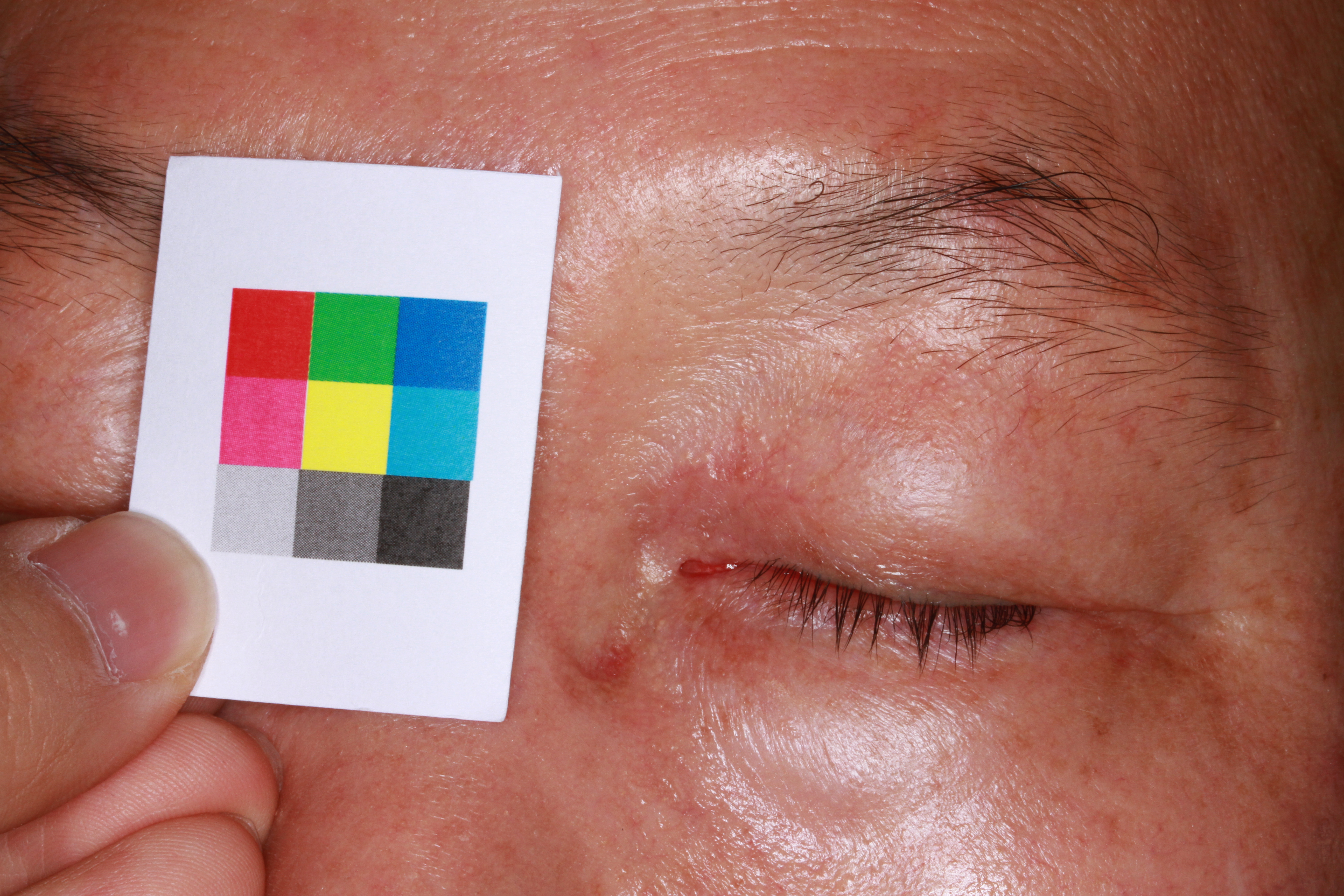

Supplement: S5 File — (ZIP) [file pone.0163092.s005.zip › 40711.JPG]

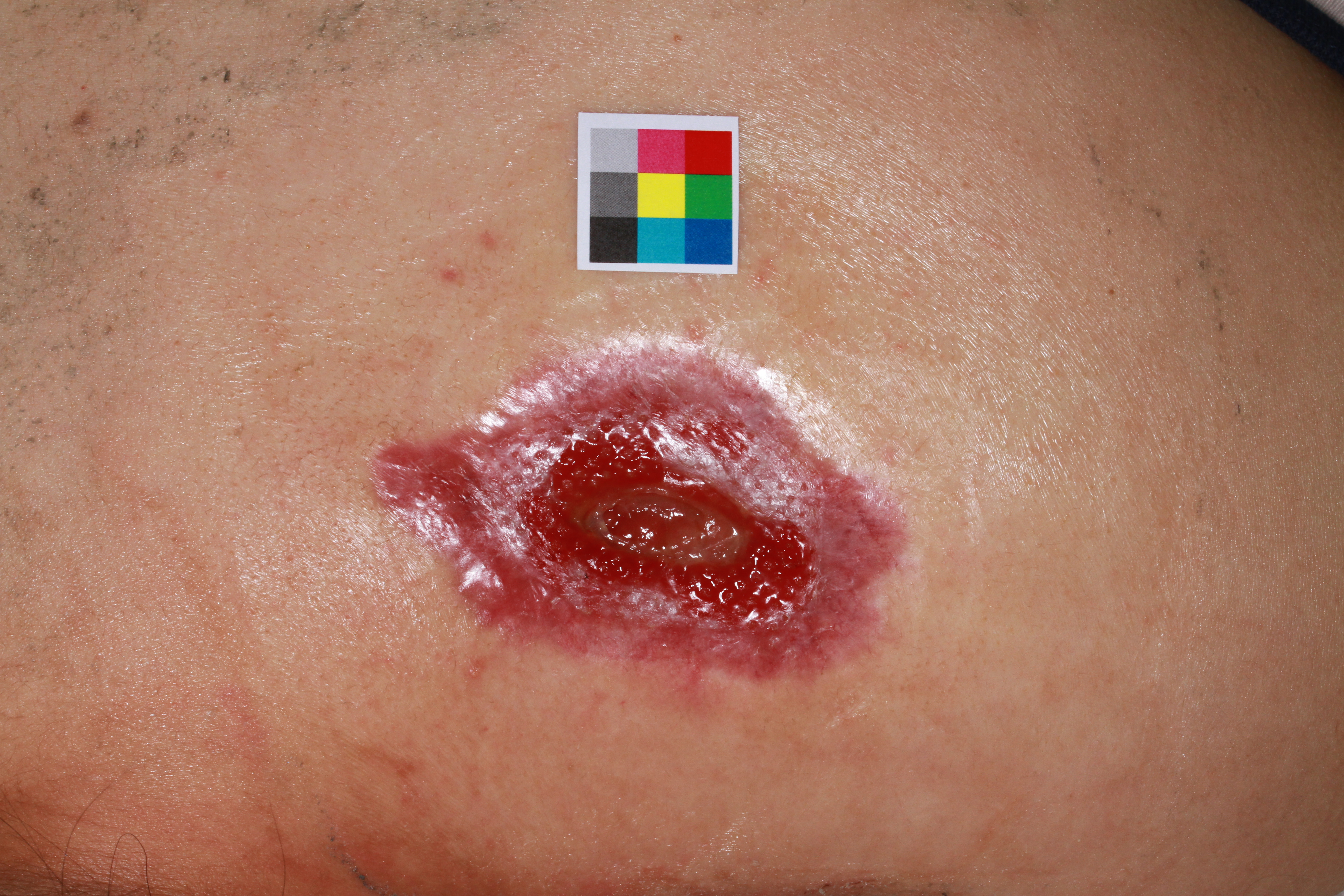

Supplement: S6 File — (ZIP) [file pone.0163092.s006.zip › 30329.JPG]

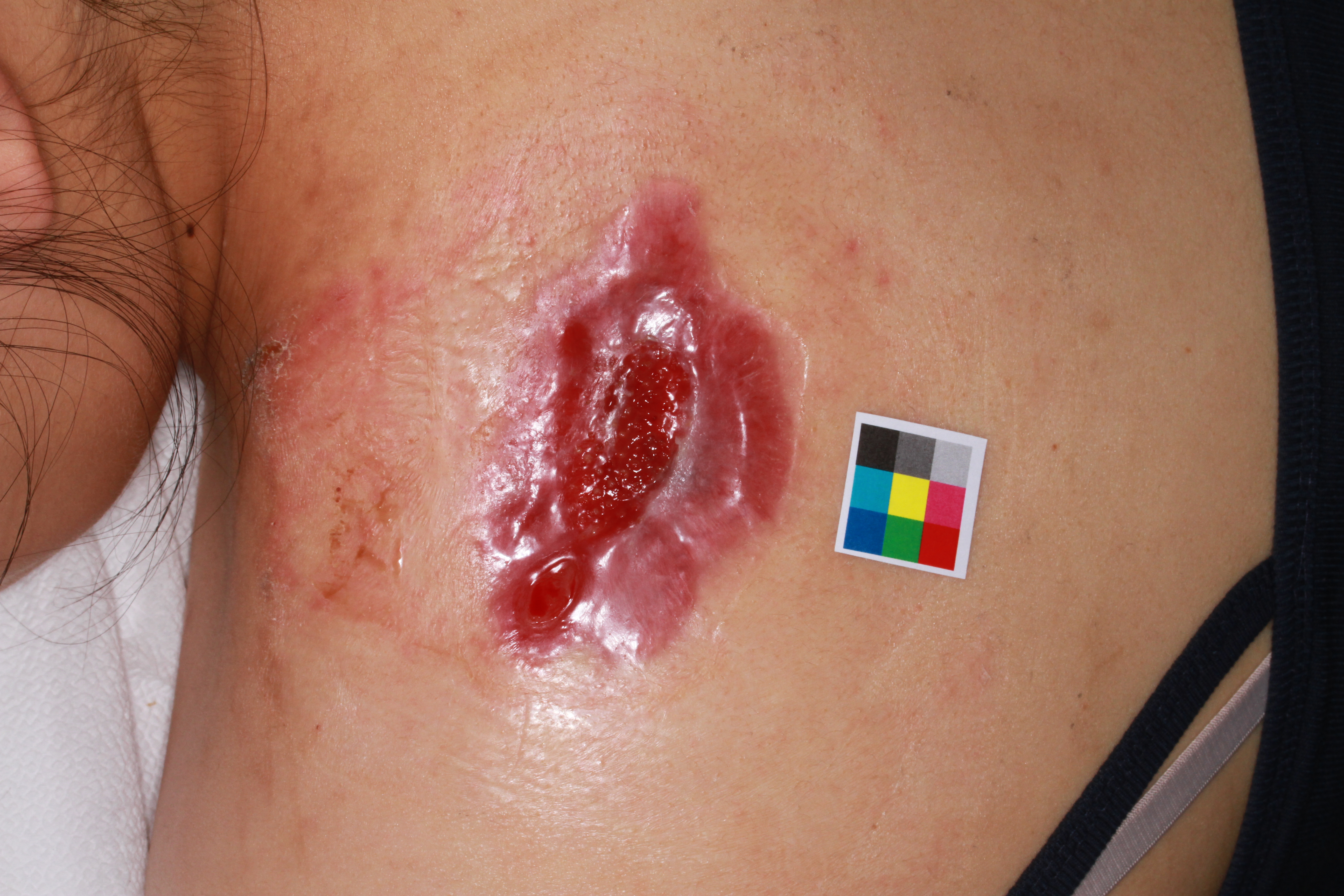

Supplement: S6 File — (ZIP) [file pone.0163092.s006.zip › 30419.JPG]

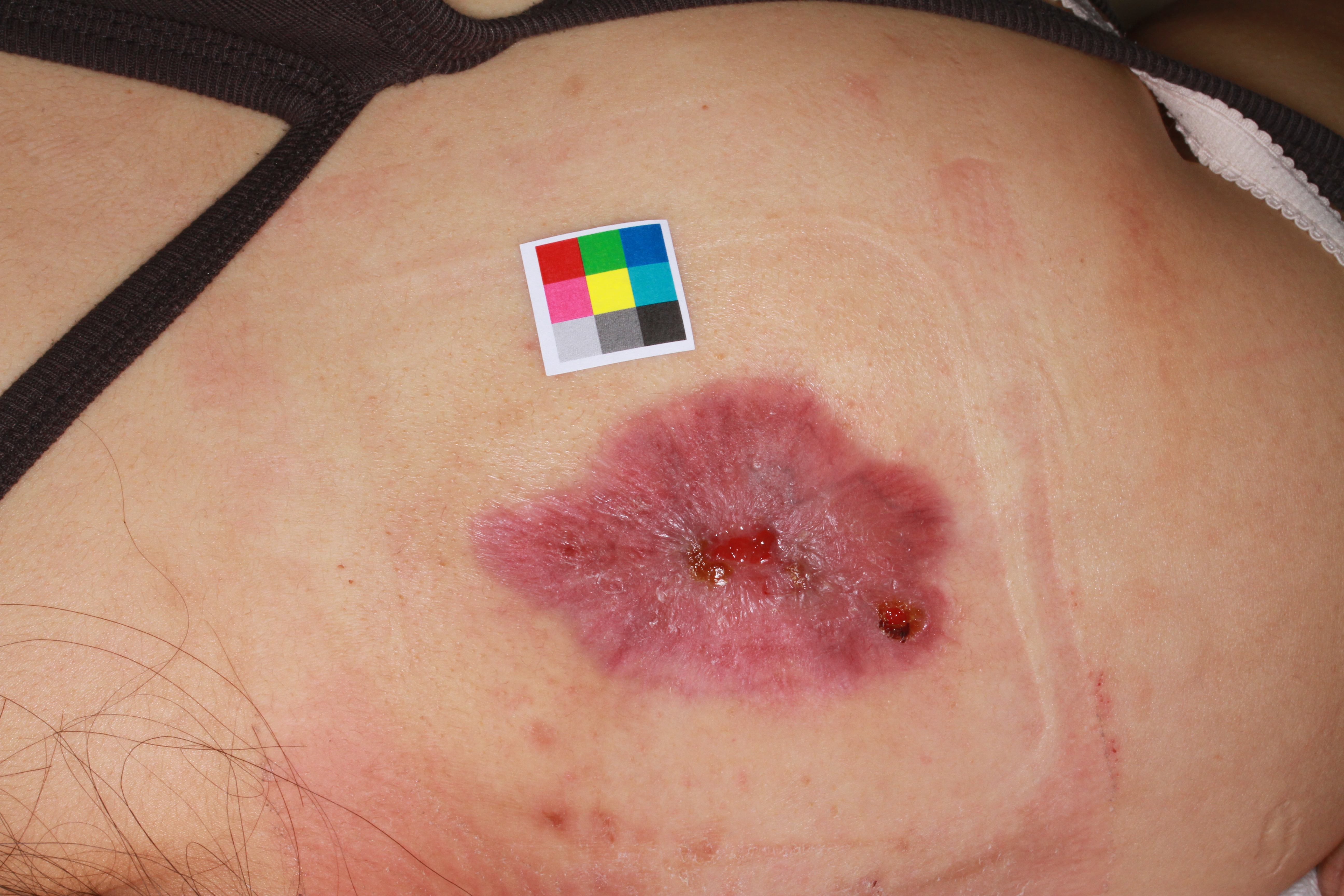

Supplement: S6 File — (ZIP) [file pone.0163092.s006.zip › 30424.JPG]

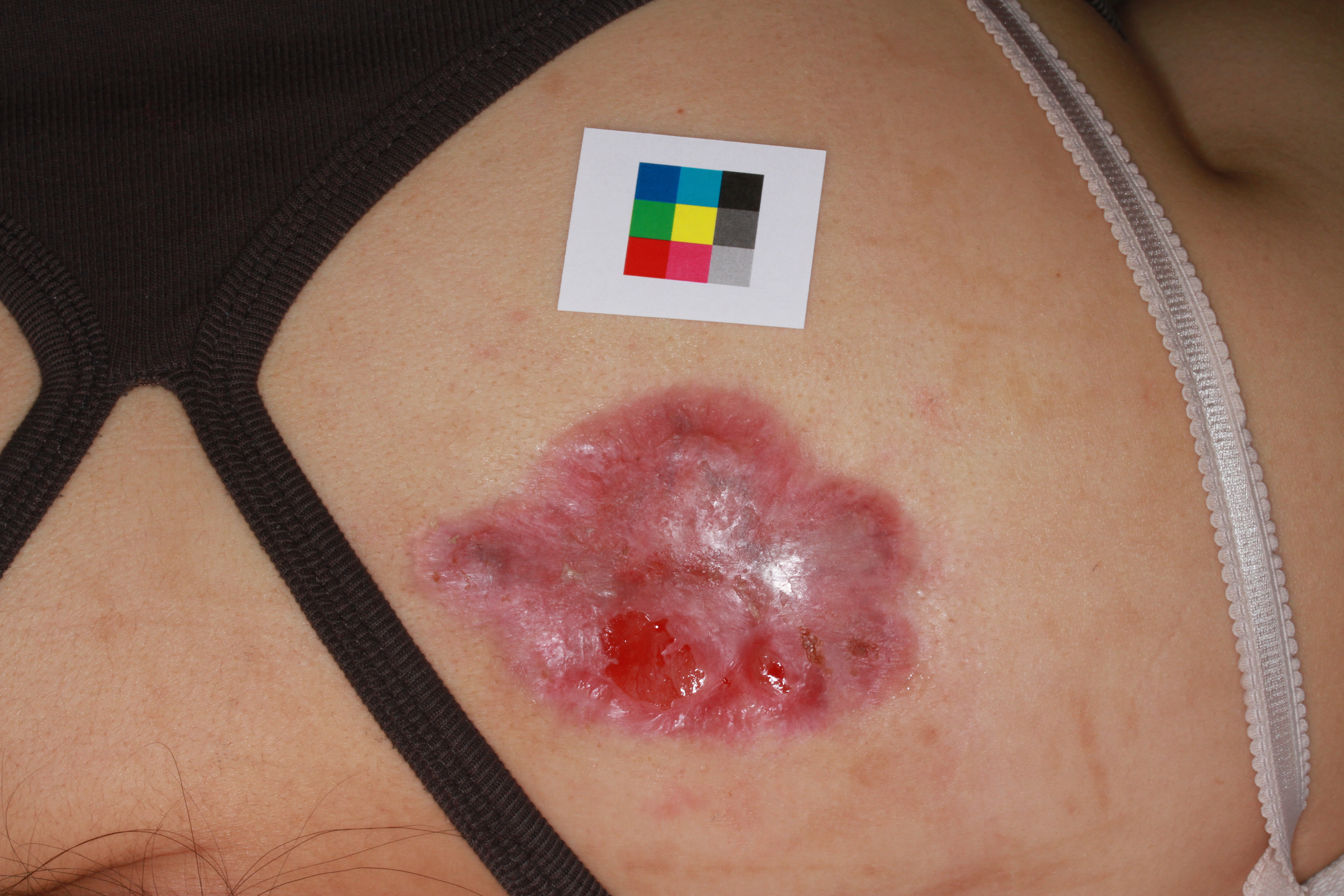

Supplement: S6 File — (ZIP) [file pone.0163092.s006.zip › 30511.JPG]

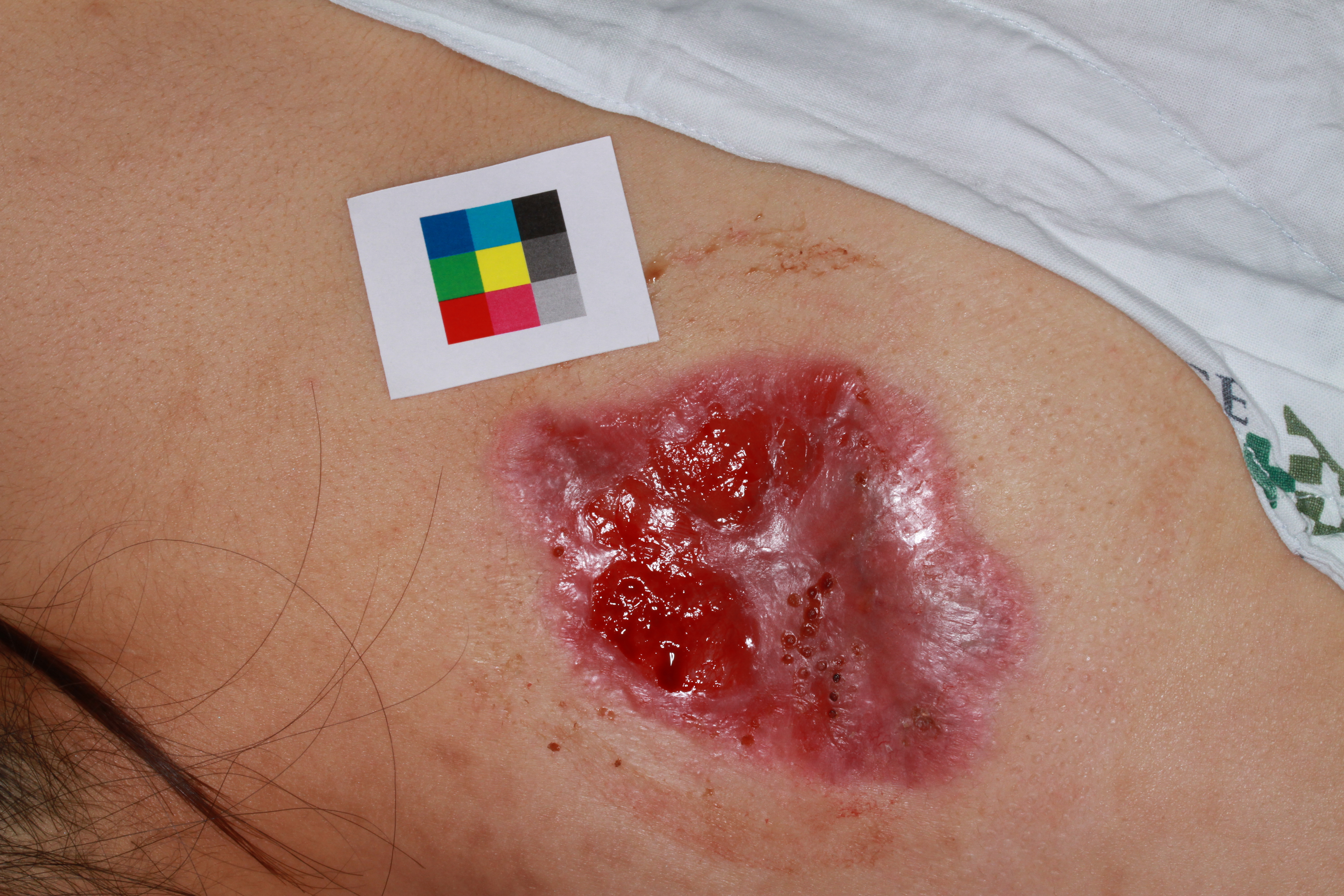

Supplement: S6 File — (ZIP) [file pone.0163092.s006.zip › 30514.JPG]

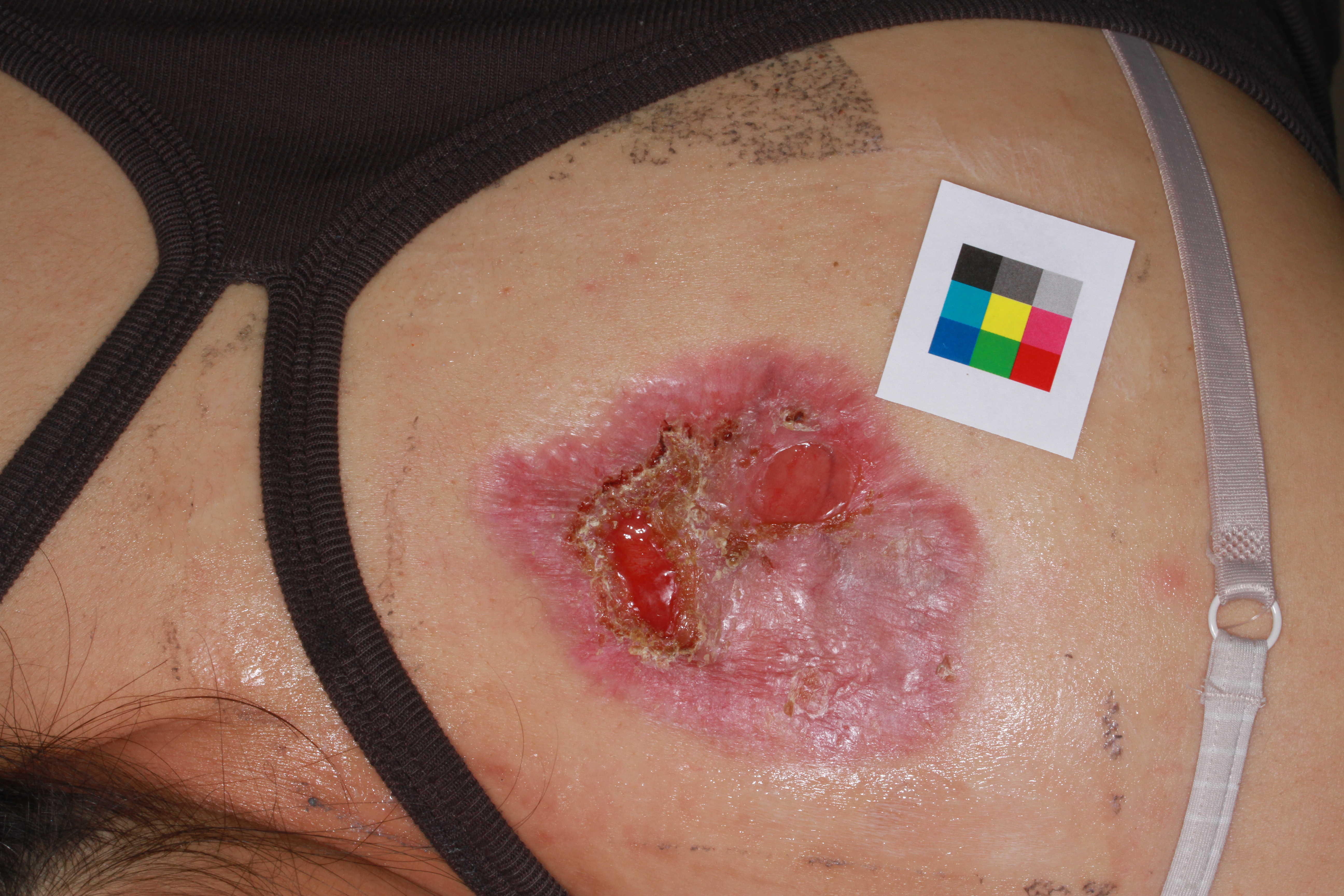

Supplement: S6 File — (ZIP) [file pone.0163092.s006.zip › 30528.JPG]

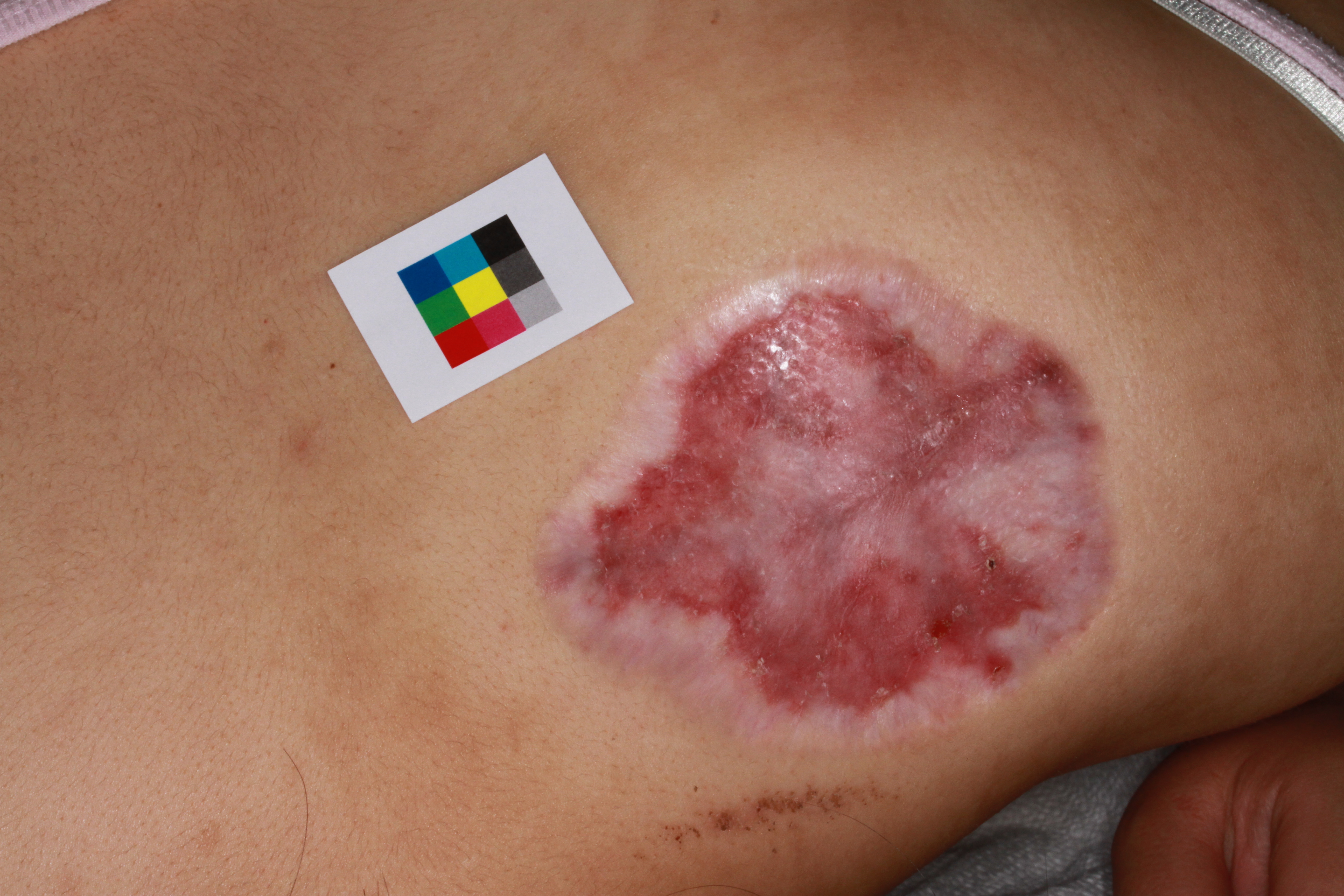

Supplement: S6 File — (ZIP) [file pone.0163092.s006.zip › 31012.JPG]

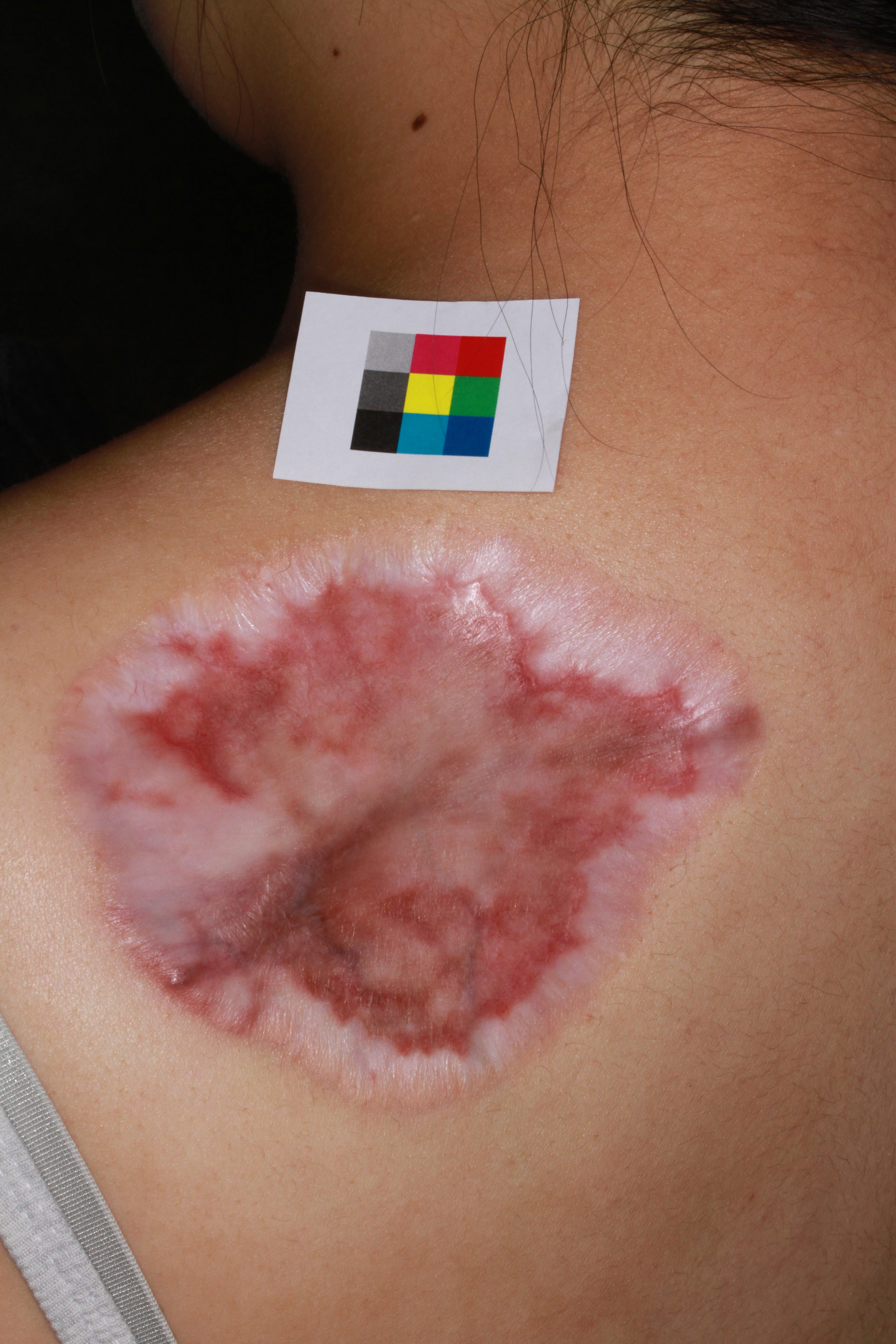

Supplement: S6 File — (ZIP) [file pone.0163092.s006.zip › 31123.JPG]

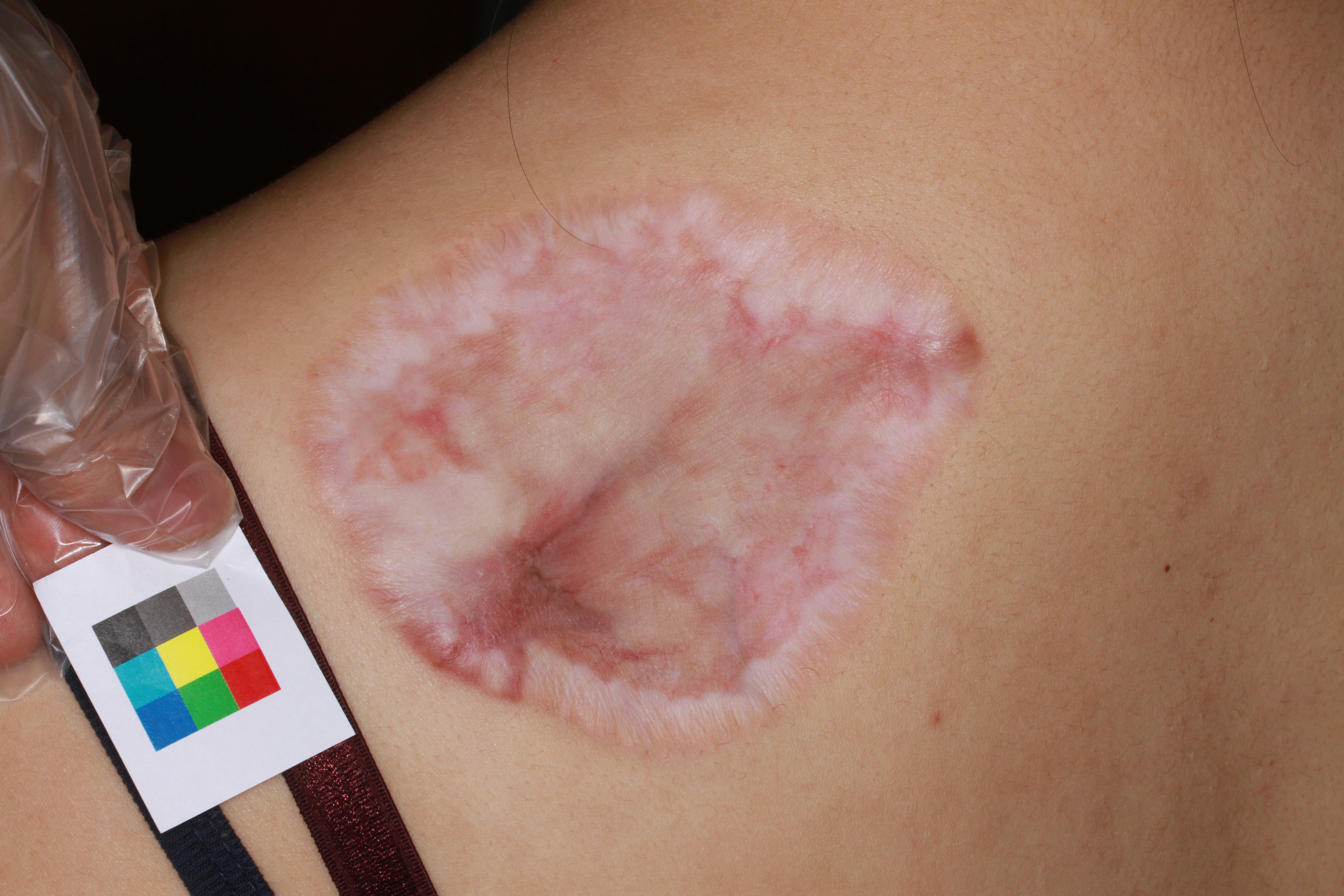

Supplement: S6 File — (ZIP) [file pone.0163092.s006.zip › 40215.JPG]

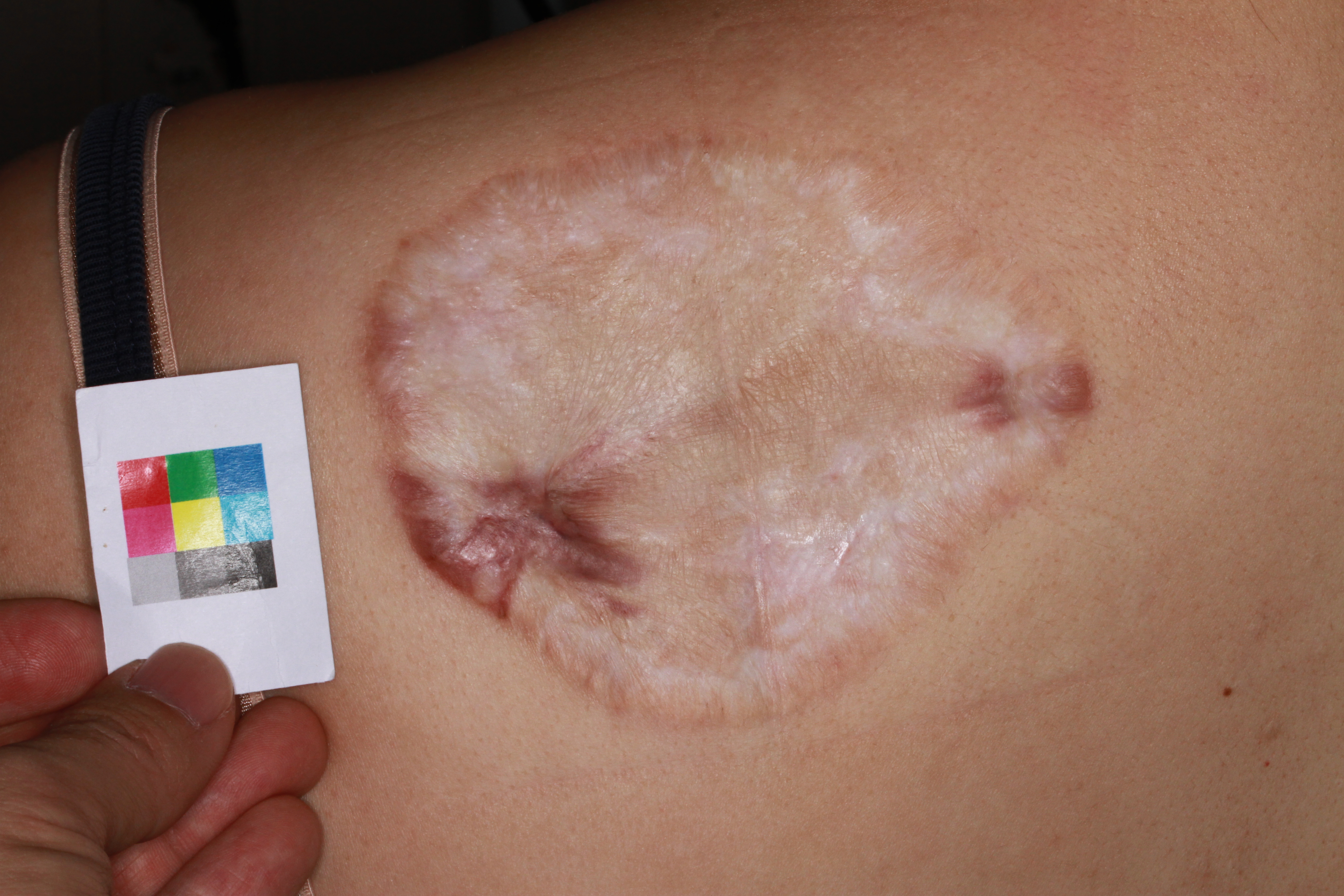

Supplement: S6 File — (ZIP) [file pone.0163092.s006.zip › 41110.JPG]

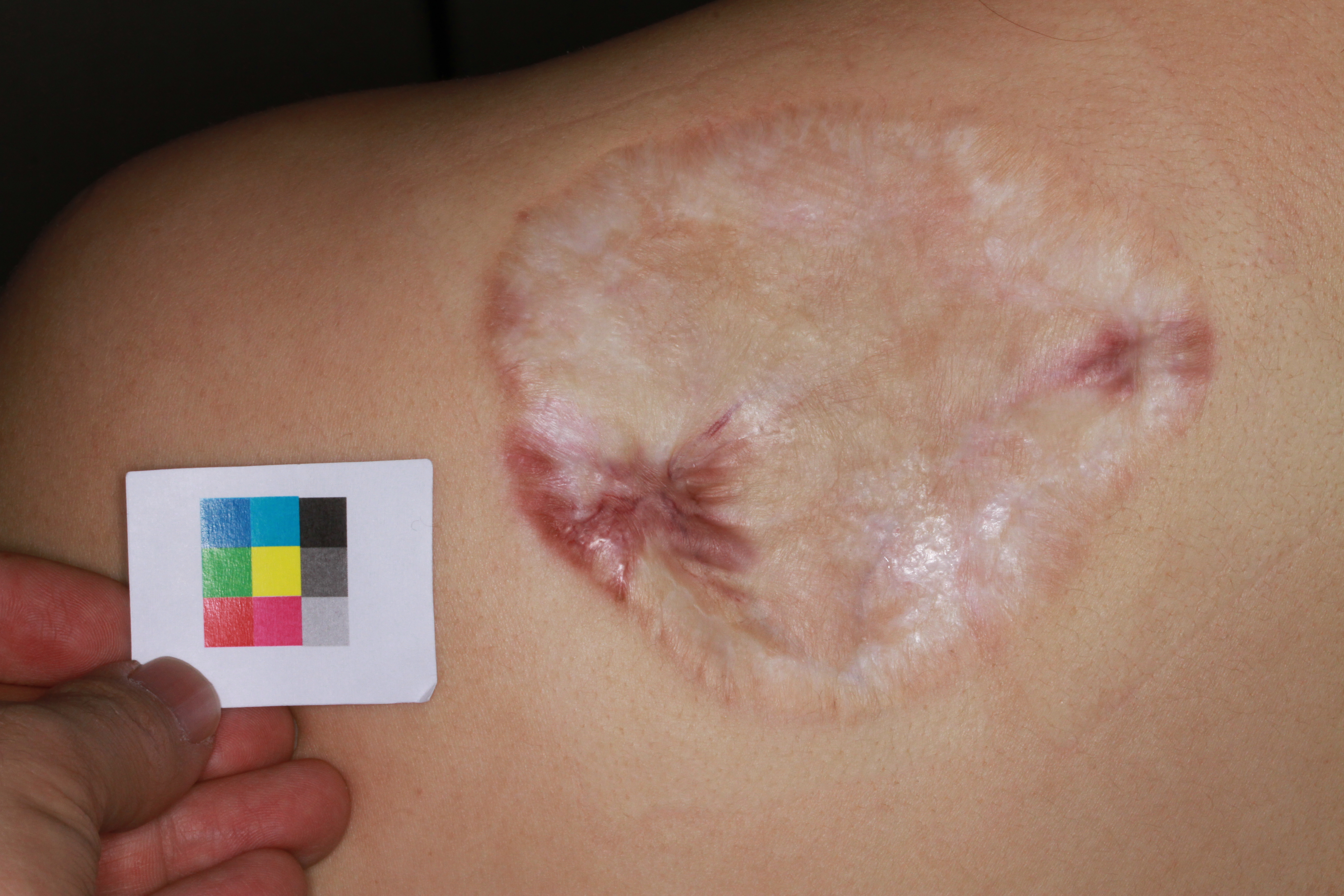

Supplement: S6 File — (ZIP) [file pone.0163092.s006.zip › 41215.JPG]

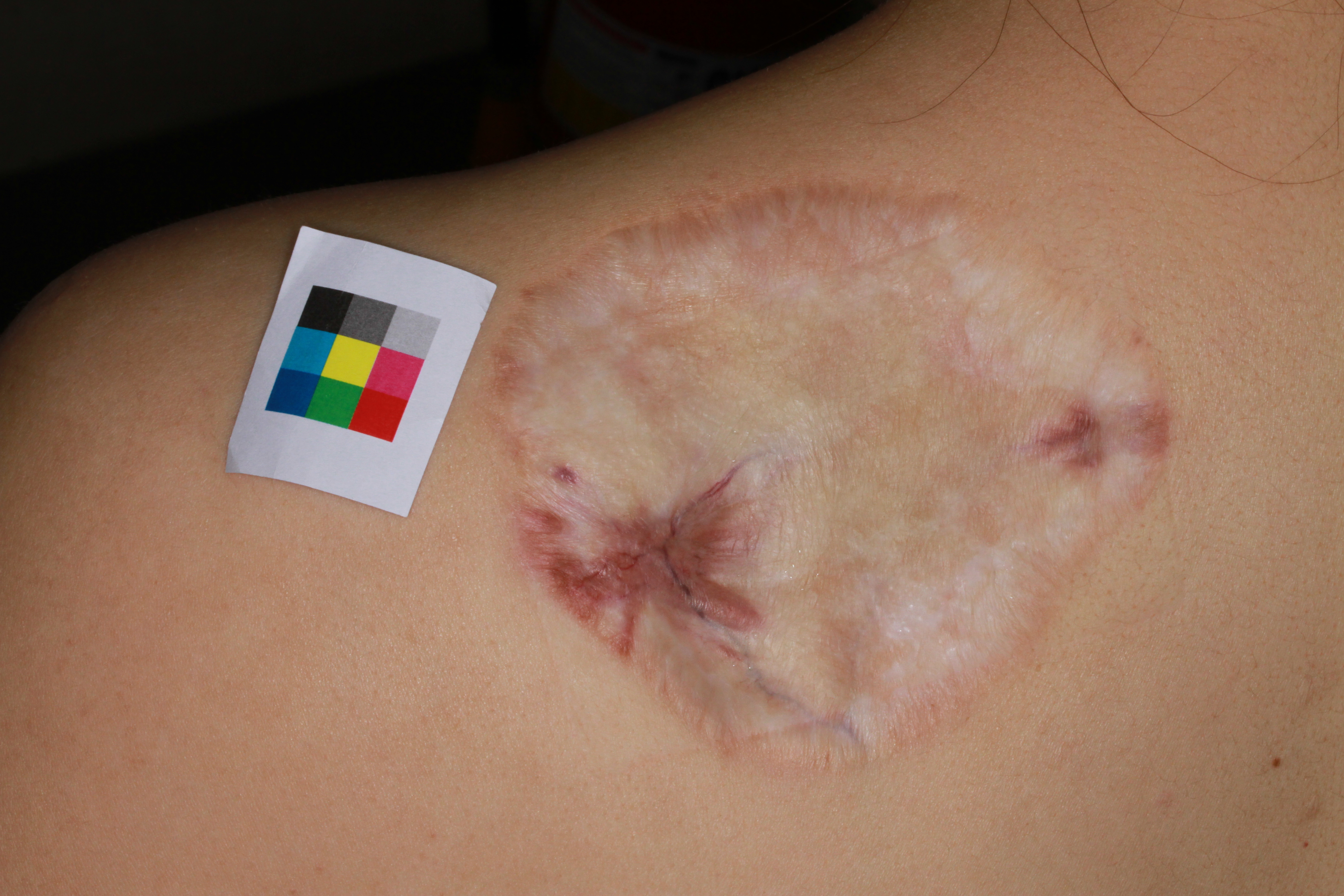

Supplement: S6 File — (ZIP) [file pone.0163092.s006.zip › 50112.JPG]

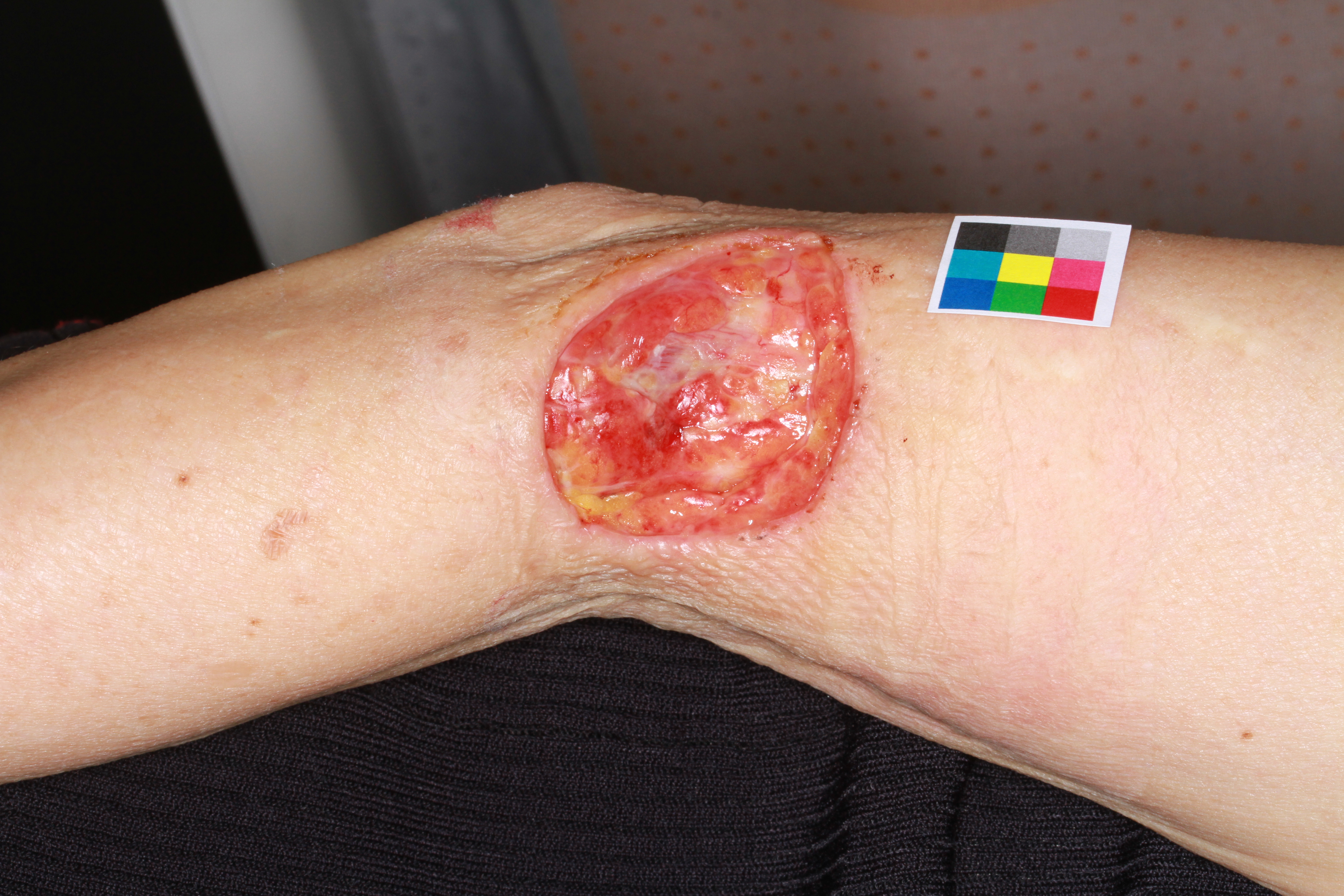

Supplement: S7 File — (ZIP) [file pone.0163092.s007.zip › 0423.JPG]

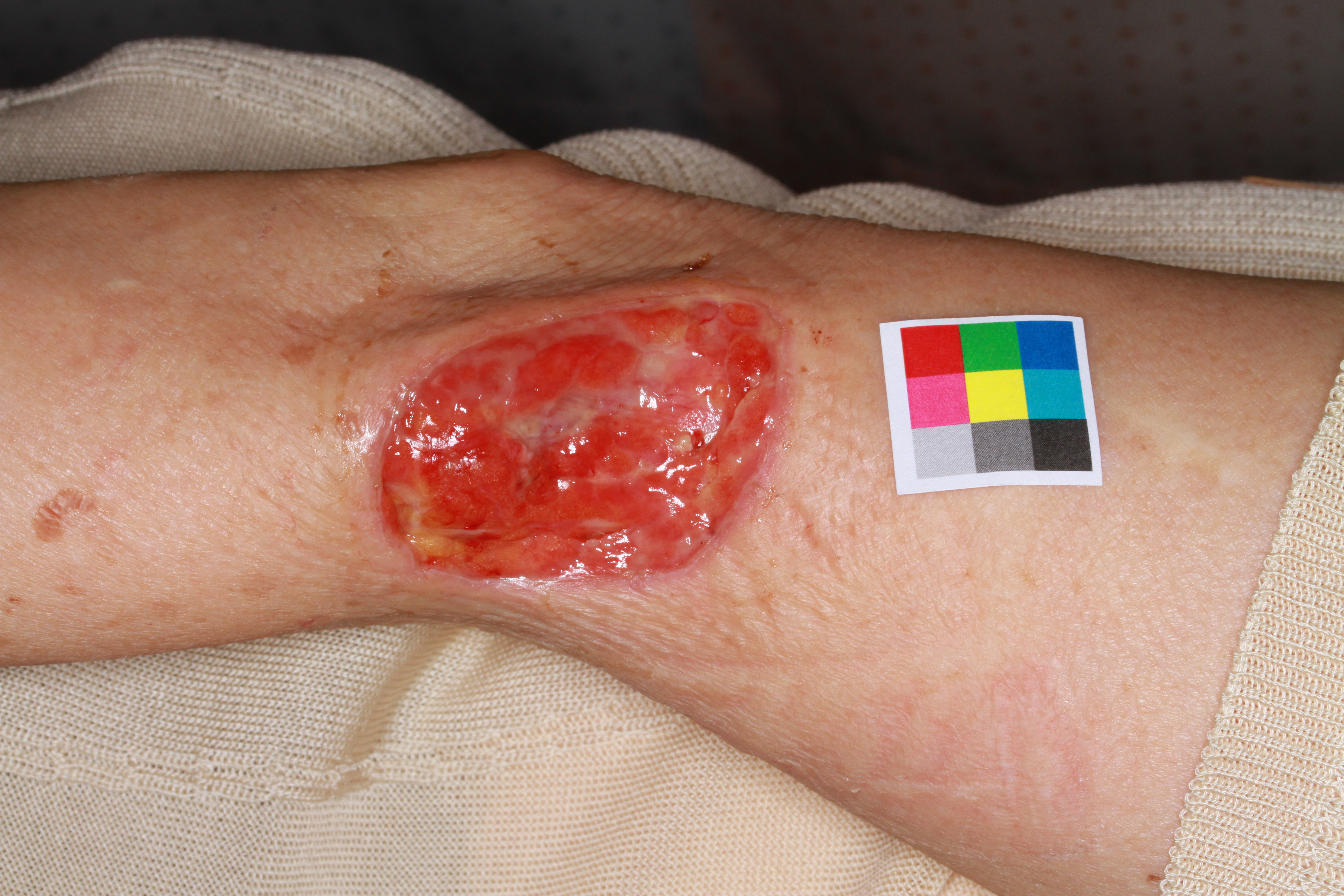

Supplement: S7 File — (ZIP) [file pone.0163092.s007.zip › 0426.JPG]

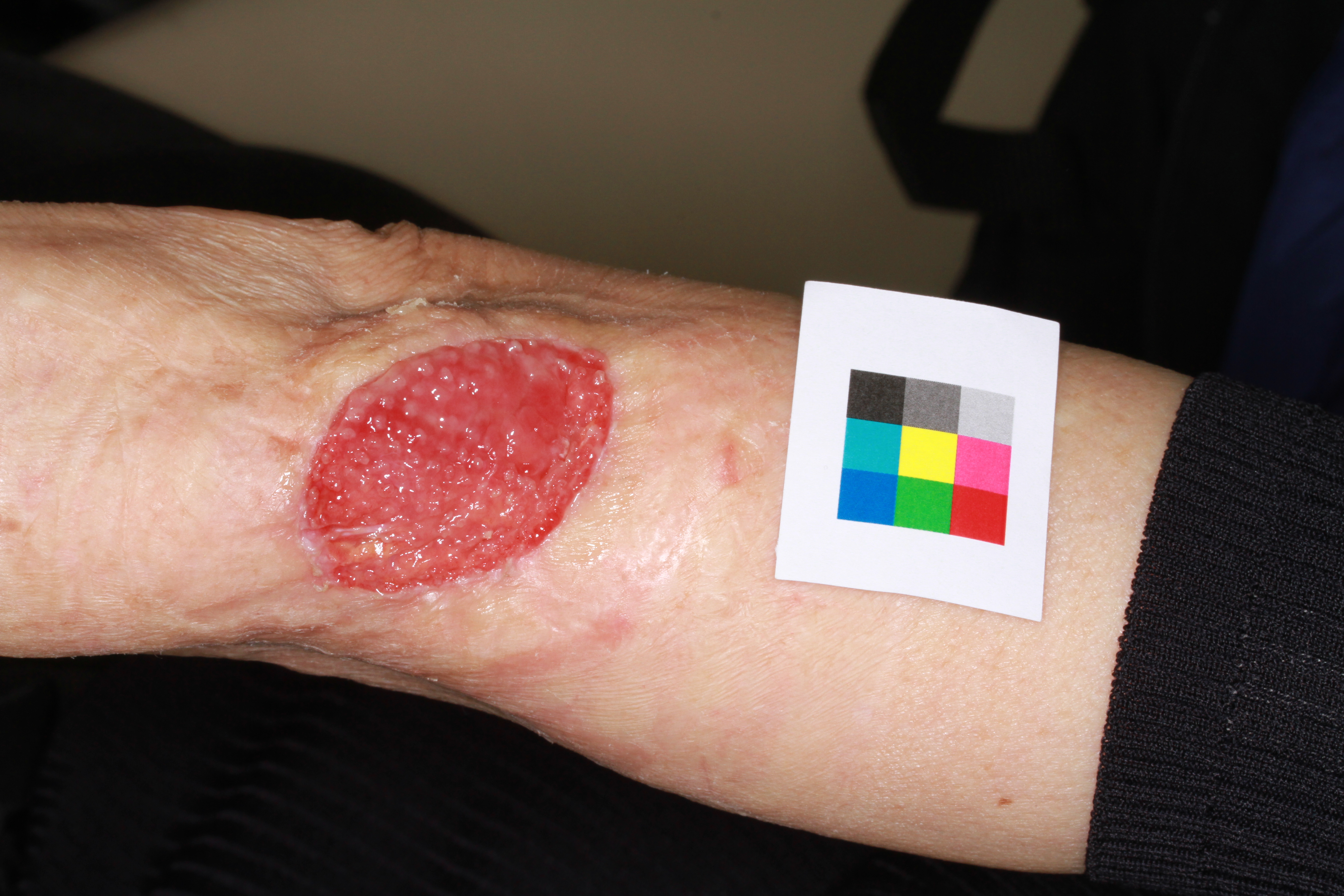

Supplement: S7 File — (ZIP) [file pone.0163092.s007.zip › 0509.JPG]

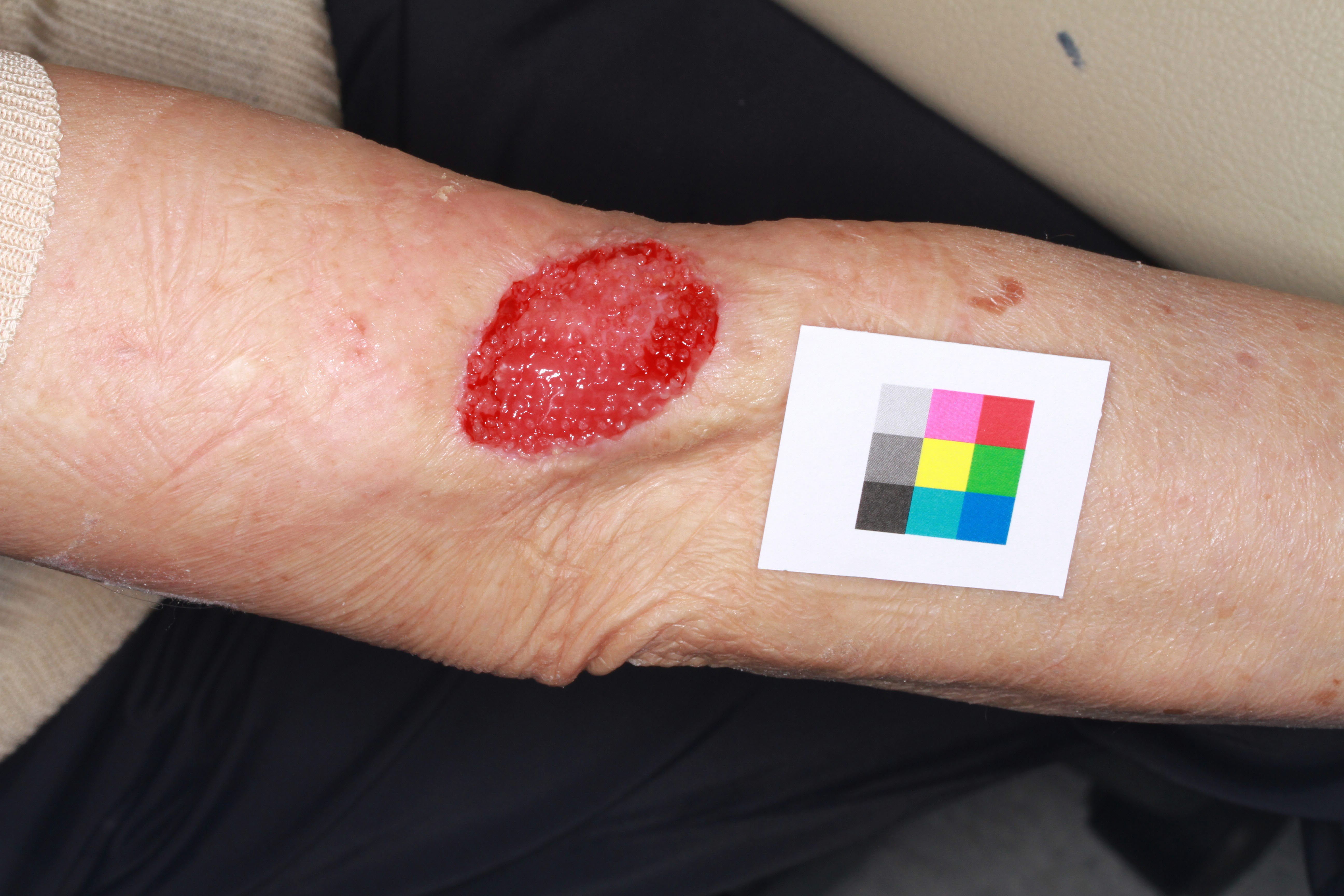

Supplement: S7 File — (ZIP) [file pone.0163092.s007.zip › 0514.JPG]

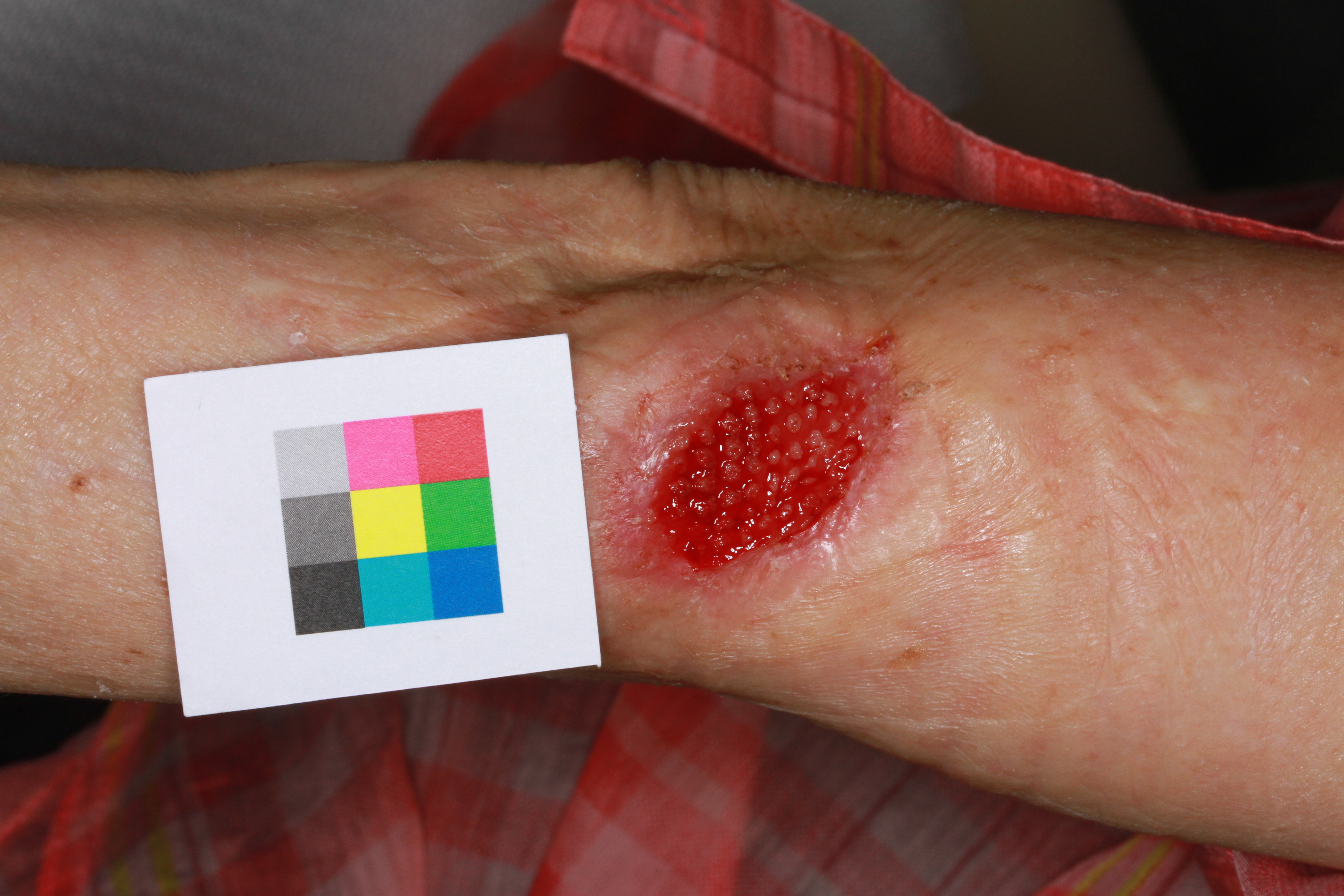

Supplement: S7 File — (ZIP) [file pone.0163092.s007.zip › 0530.JPG]

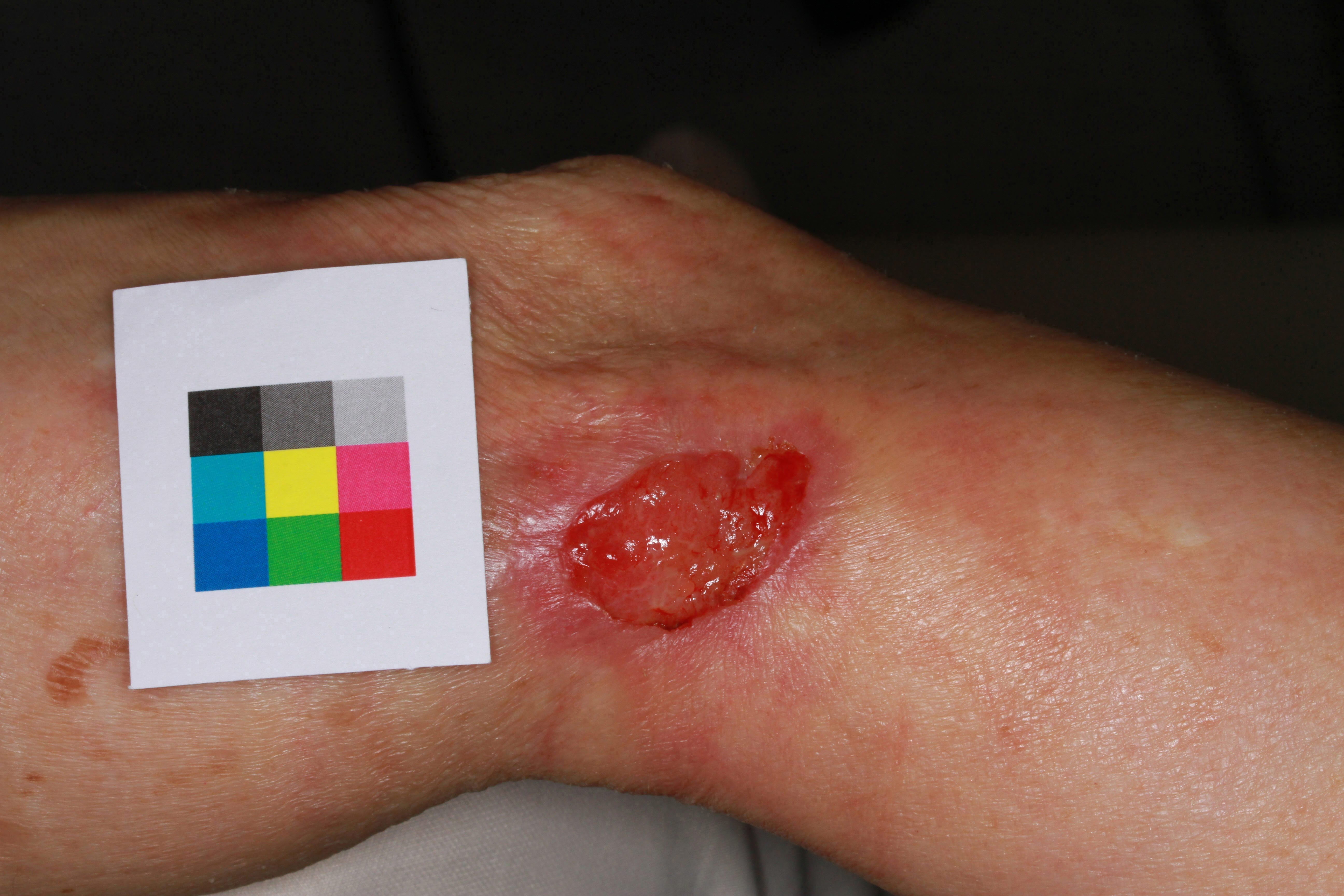

Supplement: S7 File — (ZIP) [file pone.0163092.s007.zip › 0611.JPG]

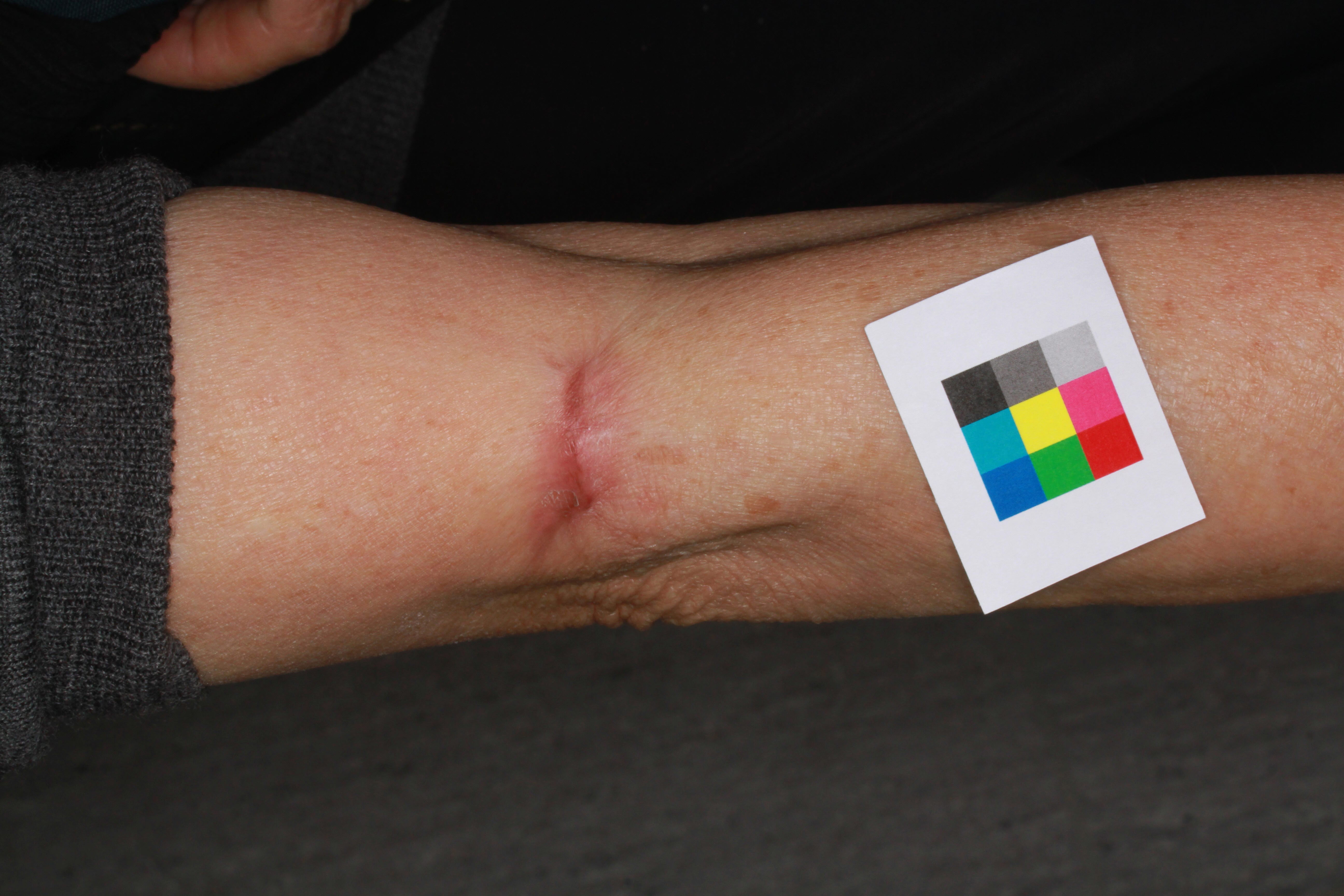

Supplement: S7 File — (ZIP) [file pone.0163092.s007.zip › 1112.JPG]

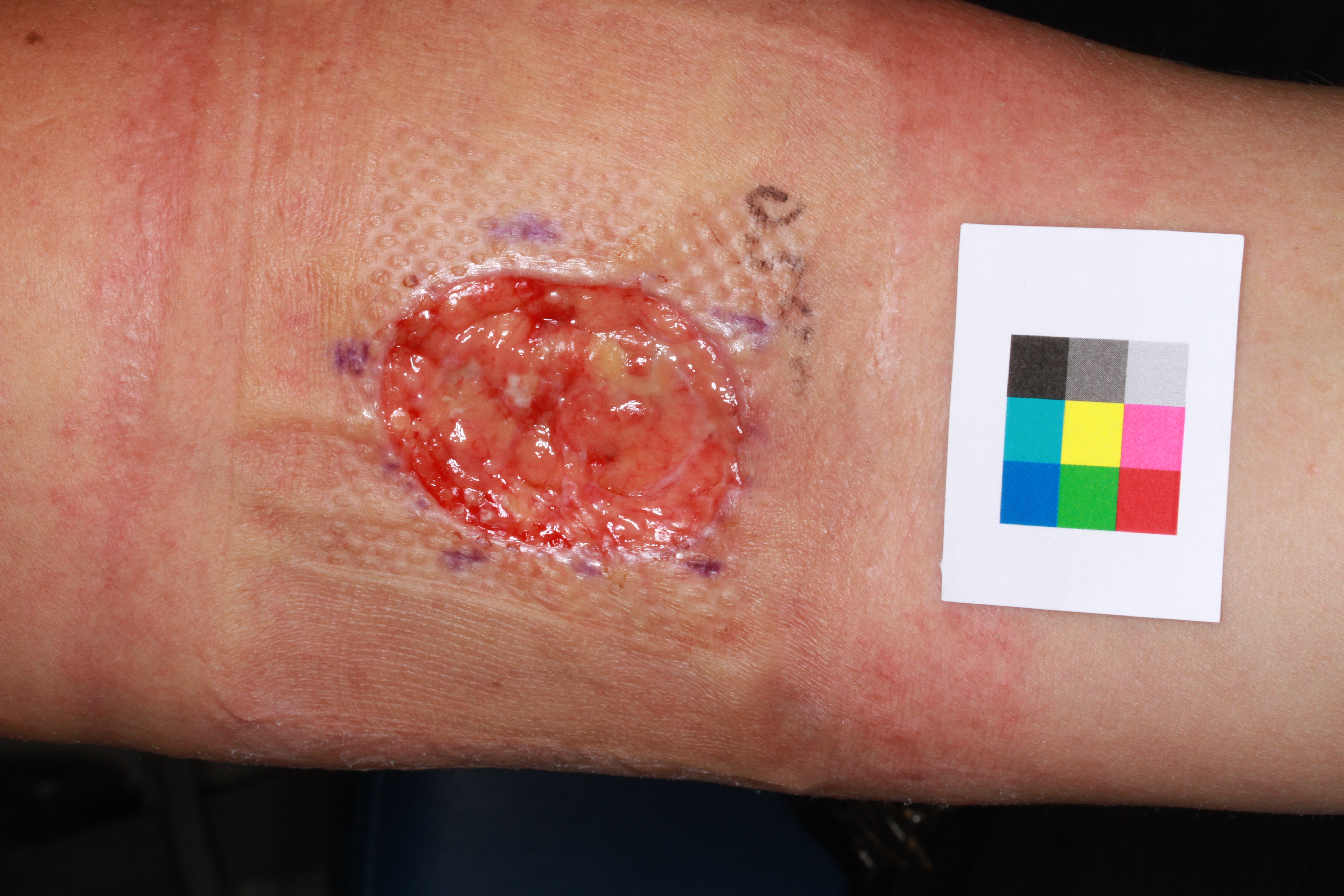

Supplement: S8 File — (ZIP) [file pone.0163092.s008.zip › 0810.JPG]

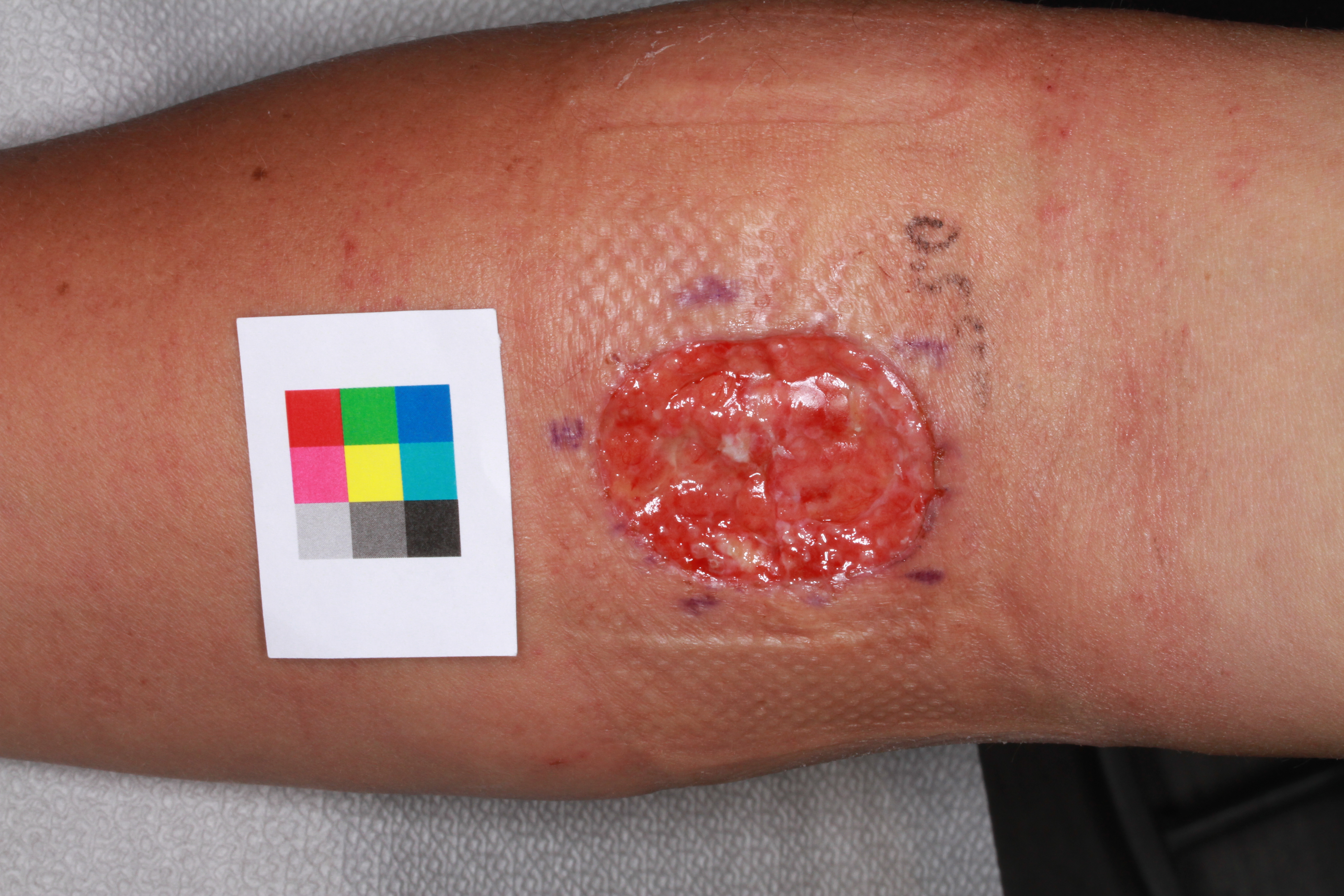

Supplement: S8 File — (ZIP) [file pone.0163092.s008.zip › 0813.JPG]

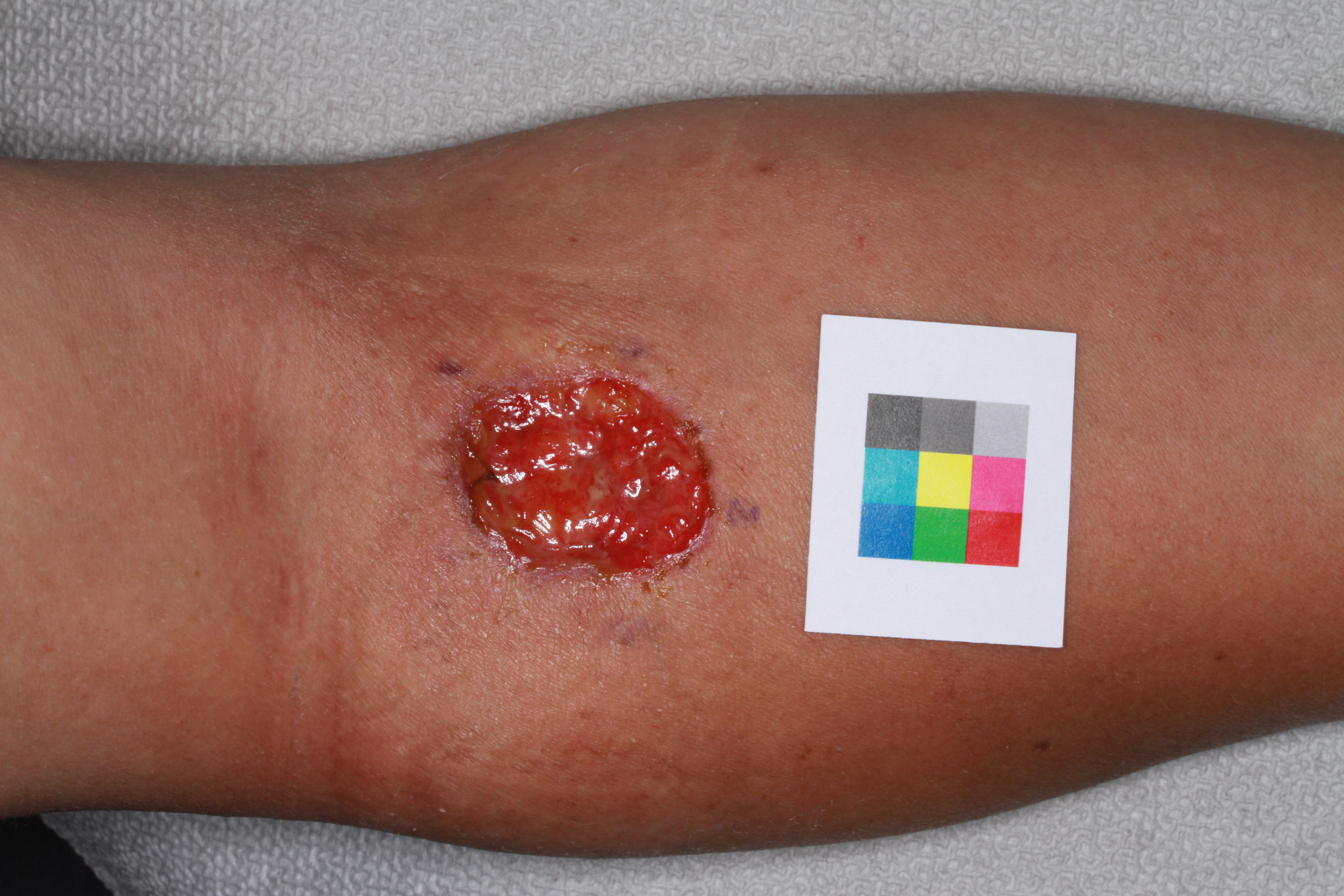

Supplement: S8 File — (ZIP) [file pone.0163092.s008.zip › 0820.JPG]

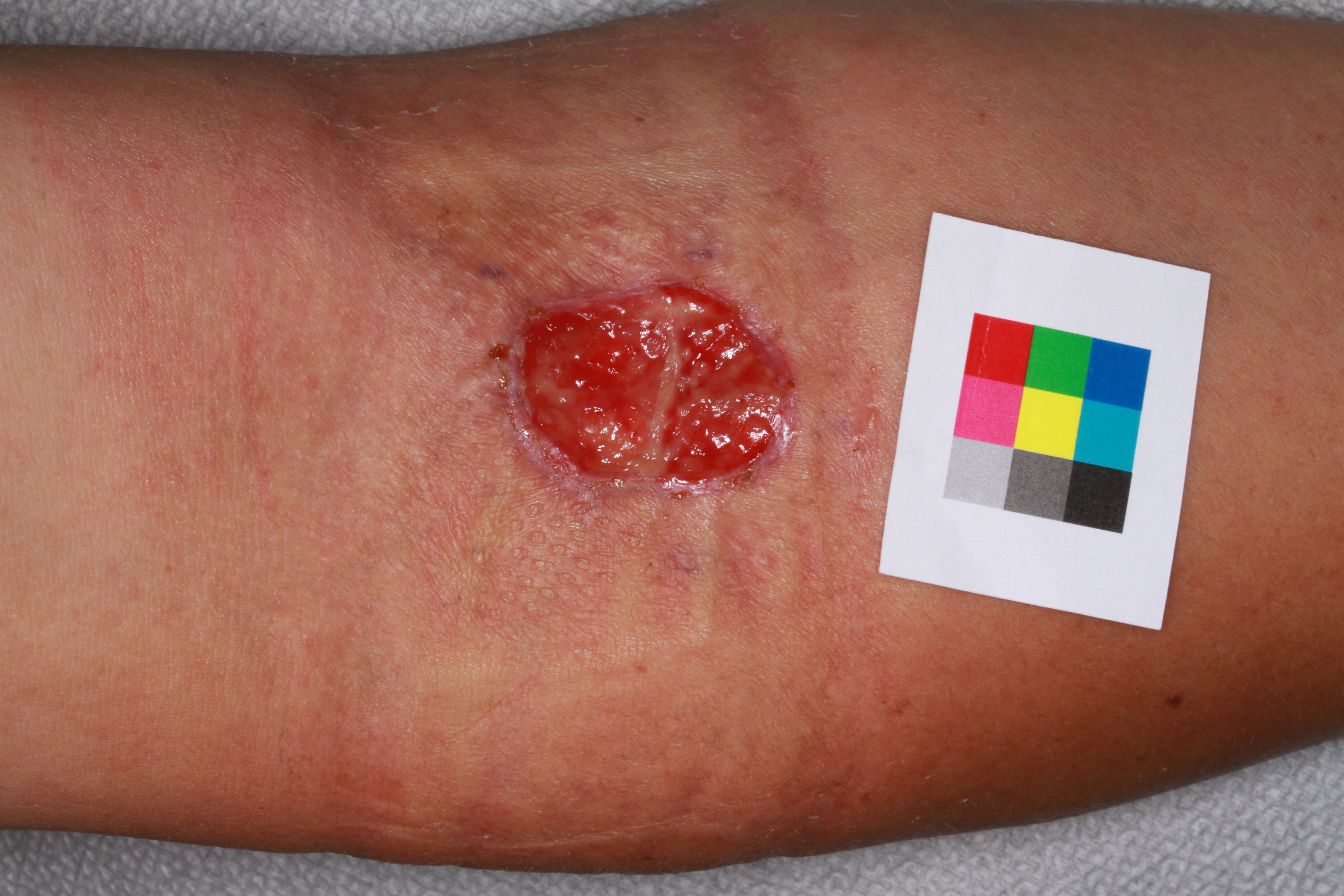

Supplement: S8 File — (ZIP) [file pone.0163092.s008.zip › 0824.JPG]

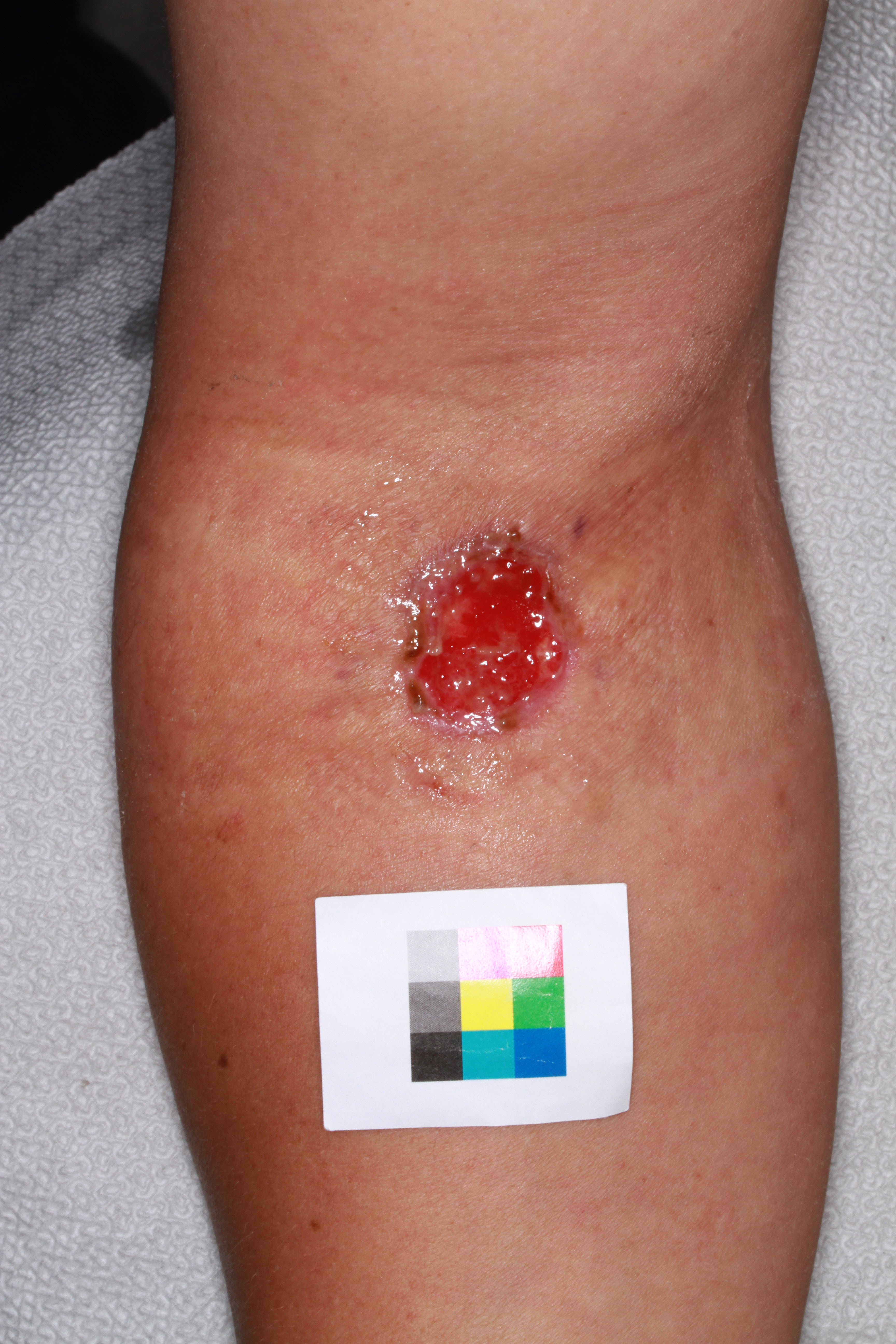

Supplement: S8 File — (ZIP) [file pone.0163092.s008.zip › 0827.JPG]

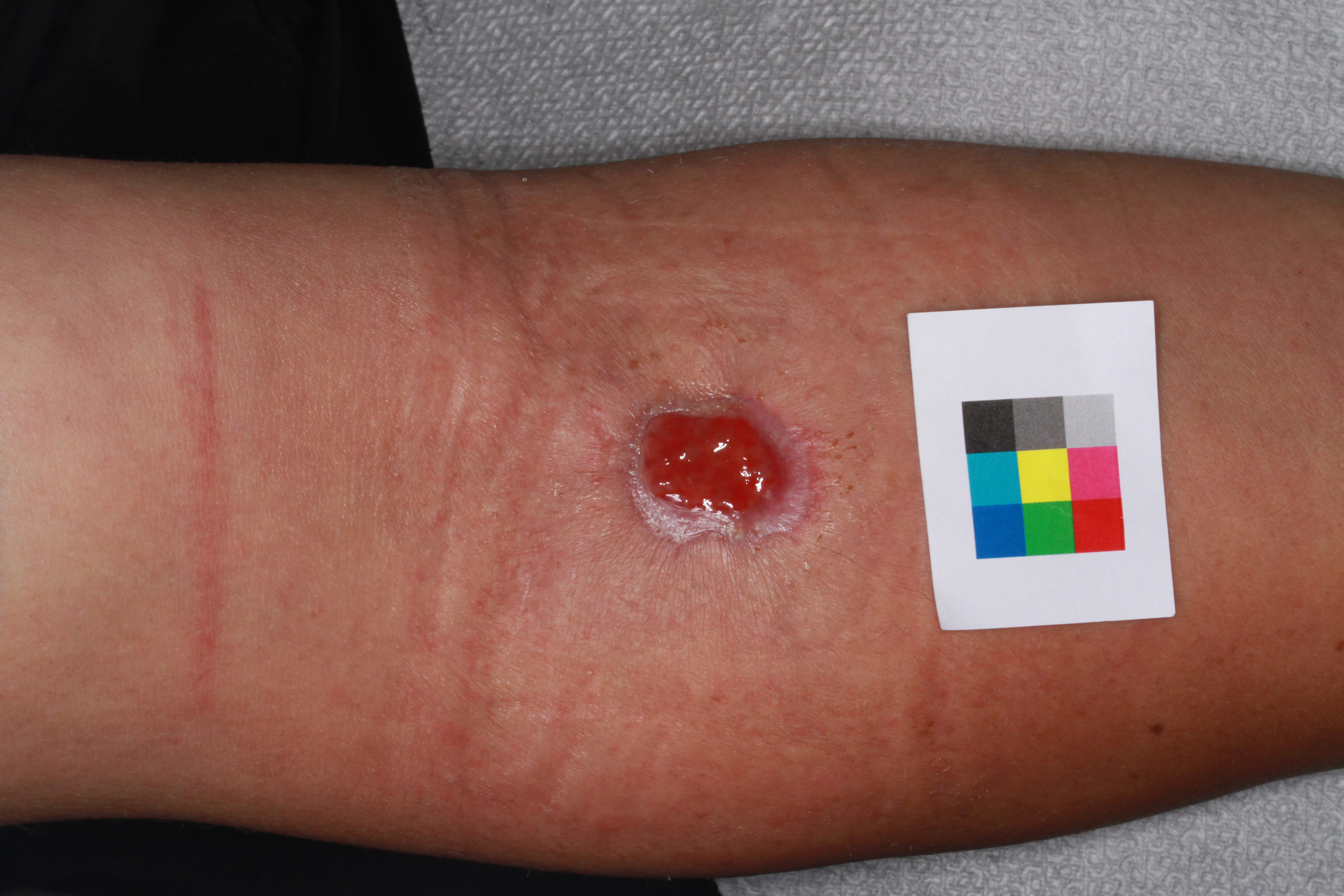

Supplement: S8 File — (ZIP) [file pone.0163092.s008.zip › 0831.JPG]

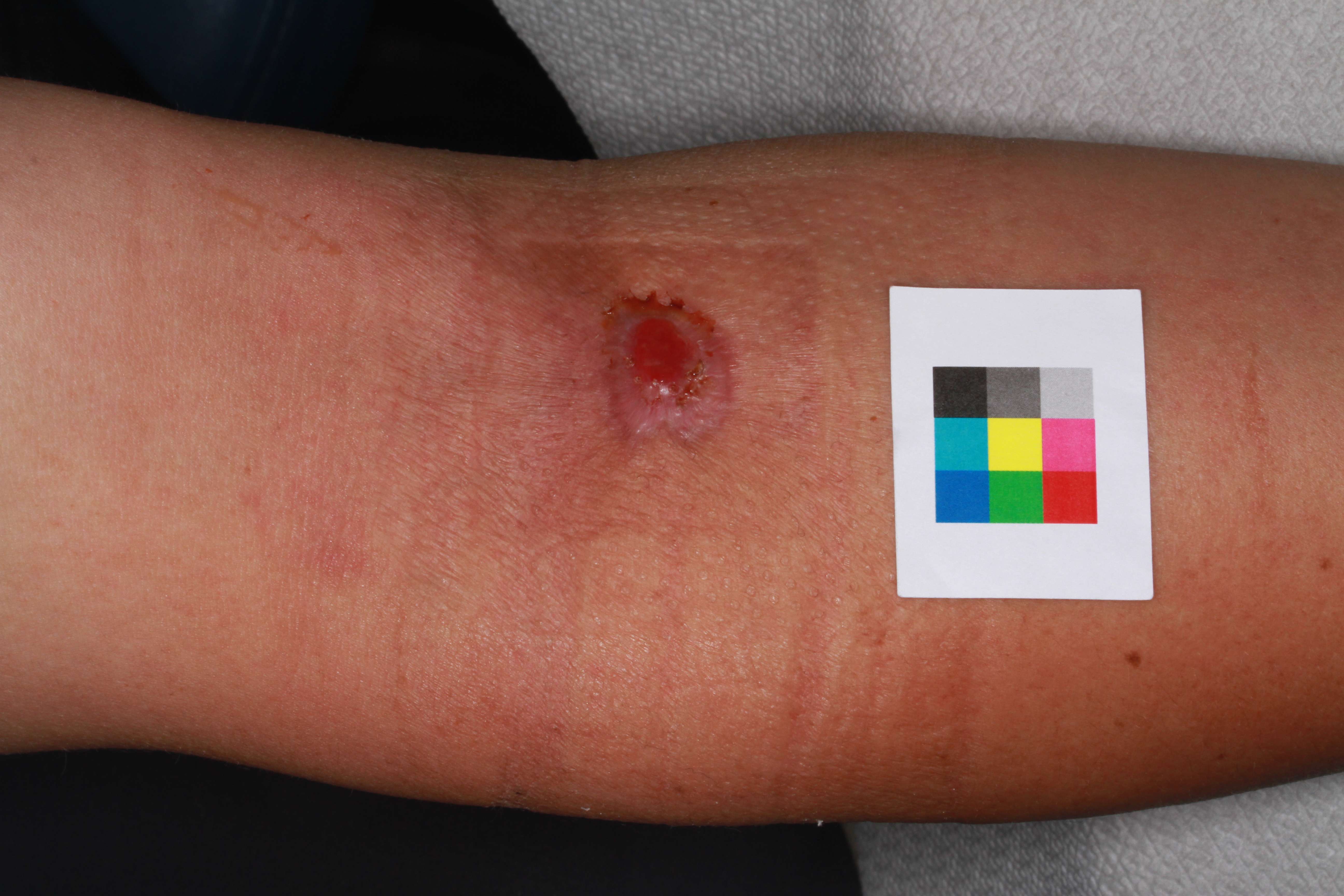

Supplement: S8 File — (ZIP) [file pone.0163092.s008.zip › 0914.JPG]

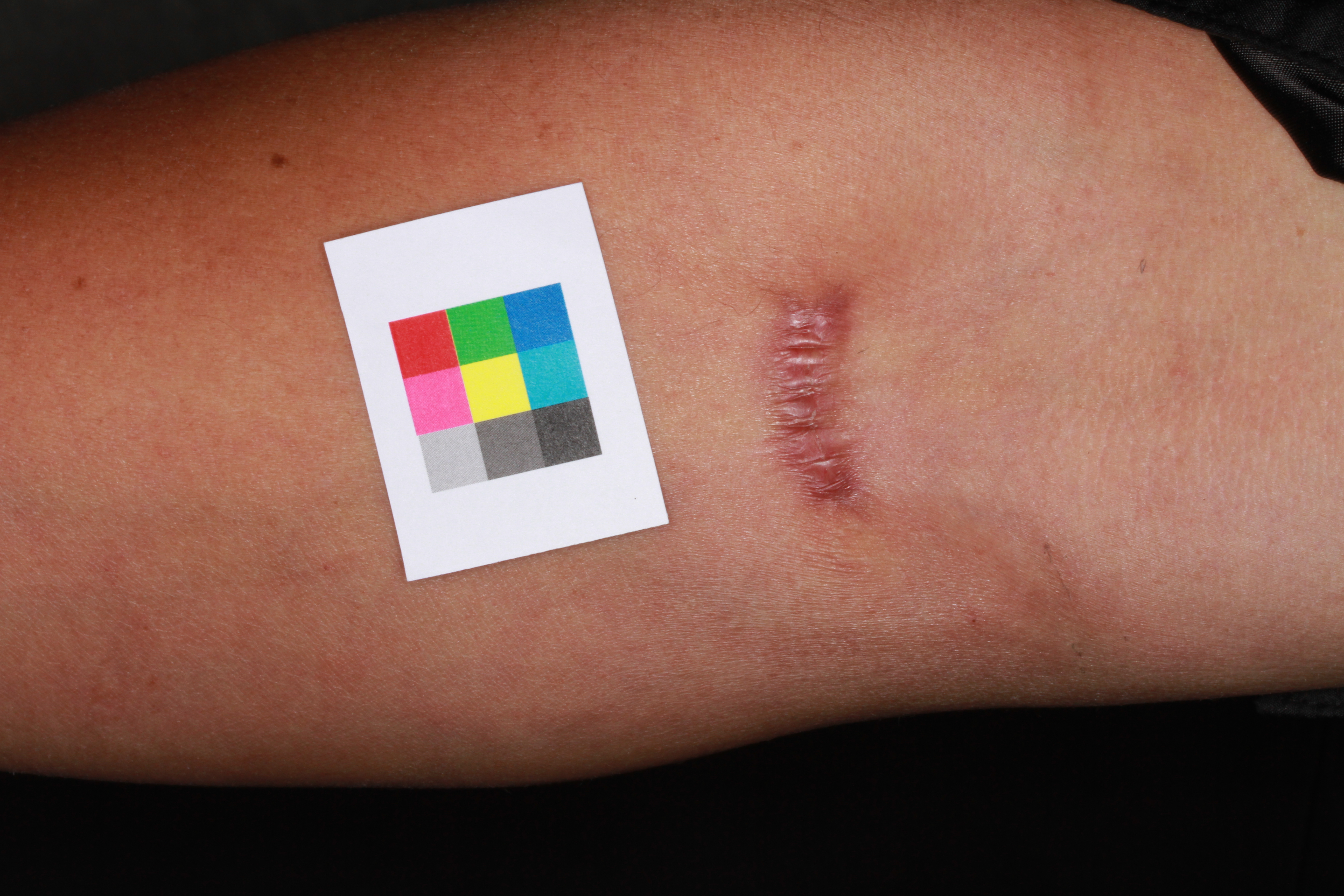

Supplement: S8 File — (ZIP) [file pone.0163092.s008.zip › 1026.JPG]

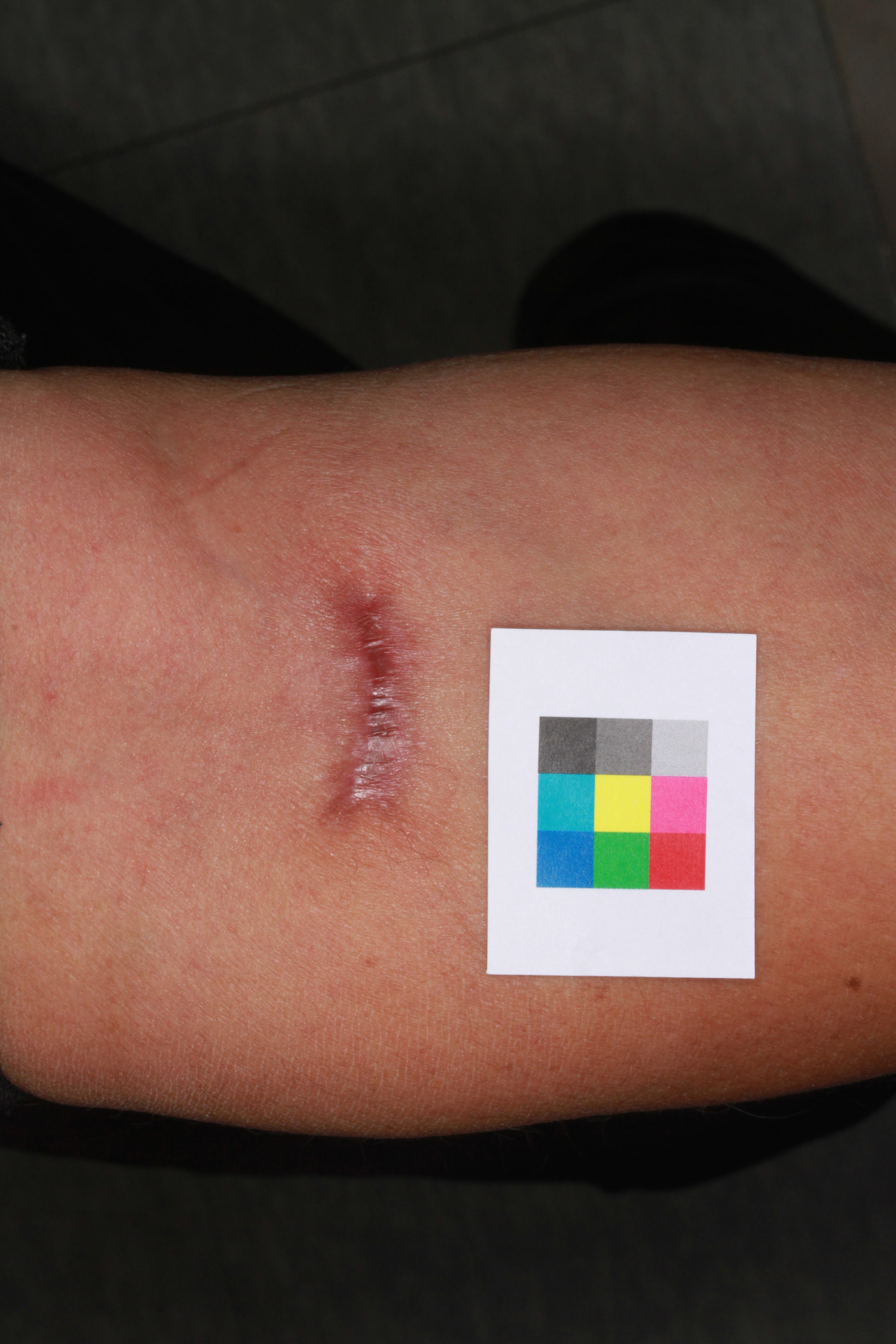

Supplement: S8 File — (ZIP) [file pone.0163092.s008.zip › 1123.JPG]

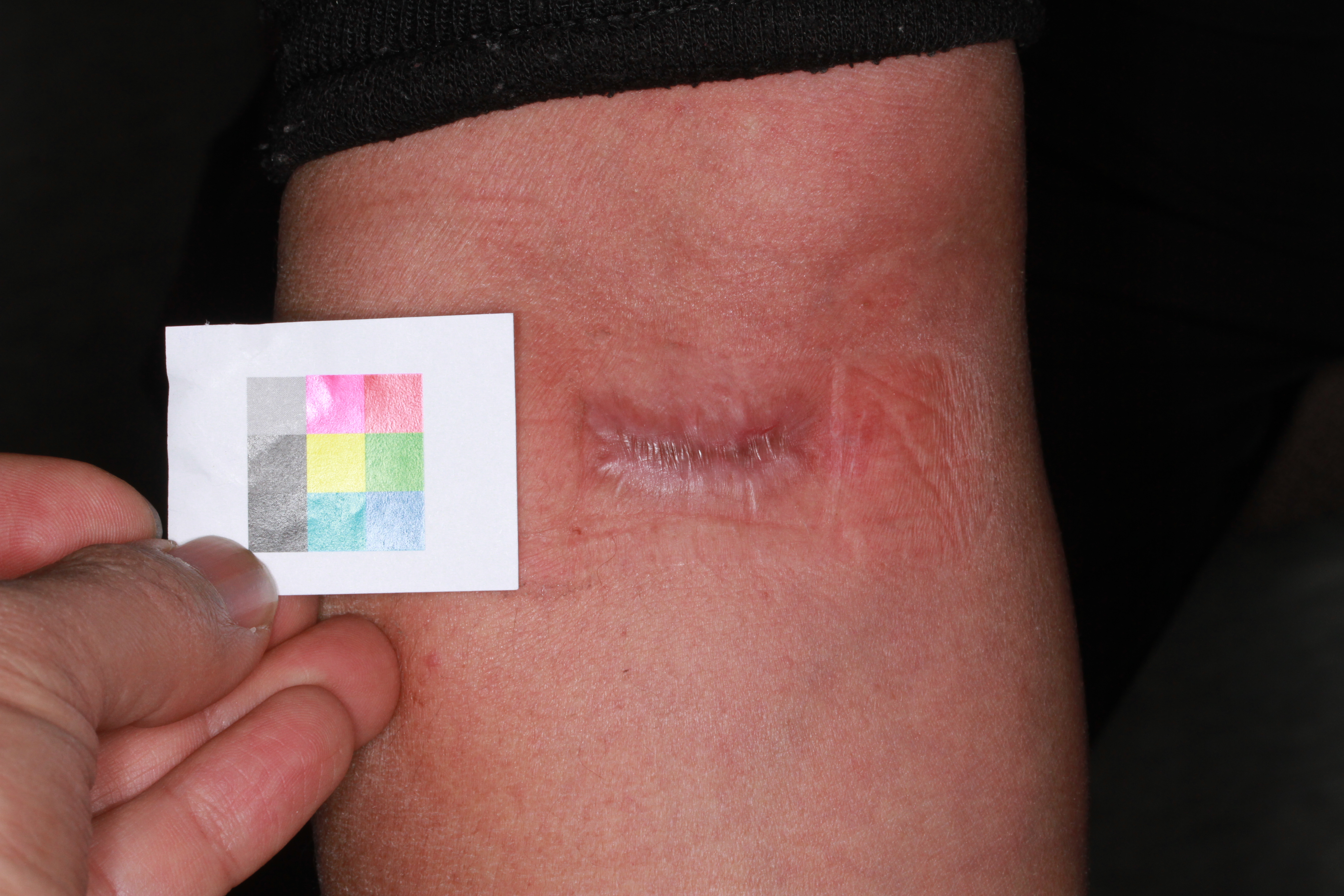

Supplement: S8 File — (ZIP) [file pone.0163092.s008.zip › 40215.JPG]

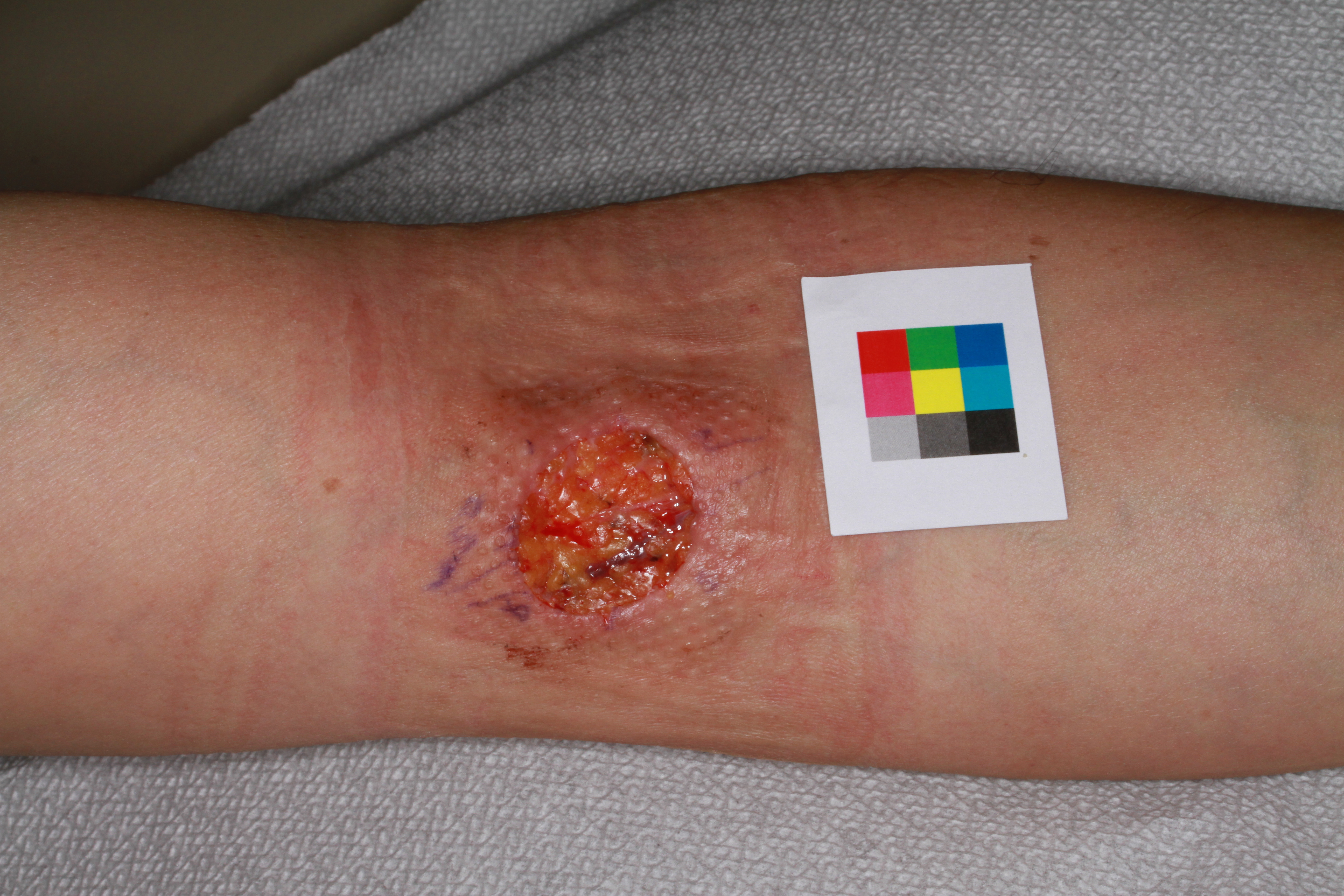

Supplement: S9 File — (ZIP) [file pone.0163092.s009.zip › 0904.JPG]

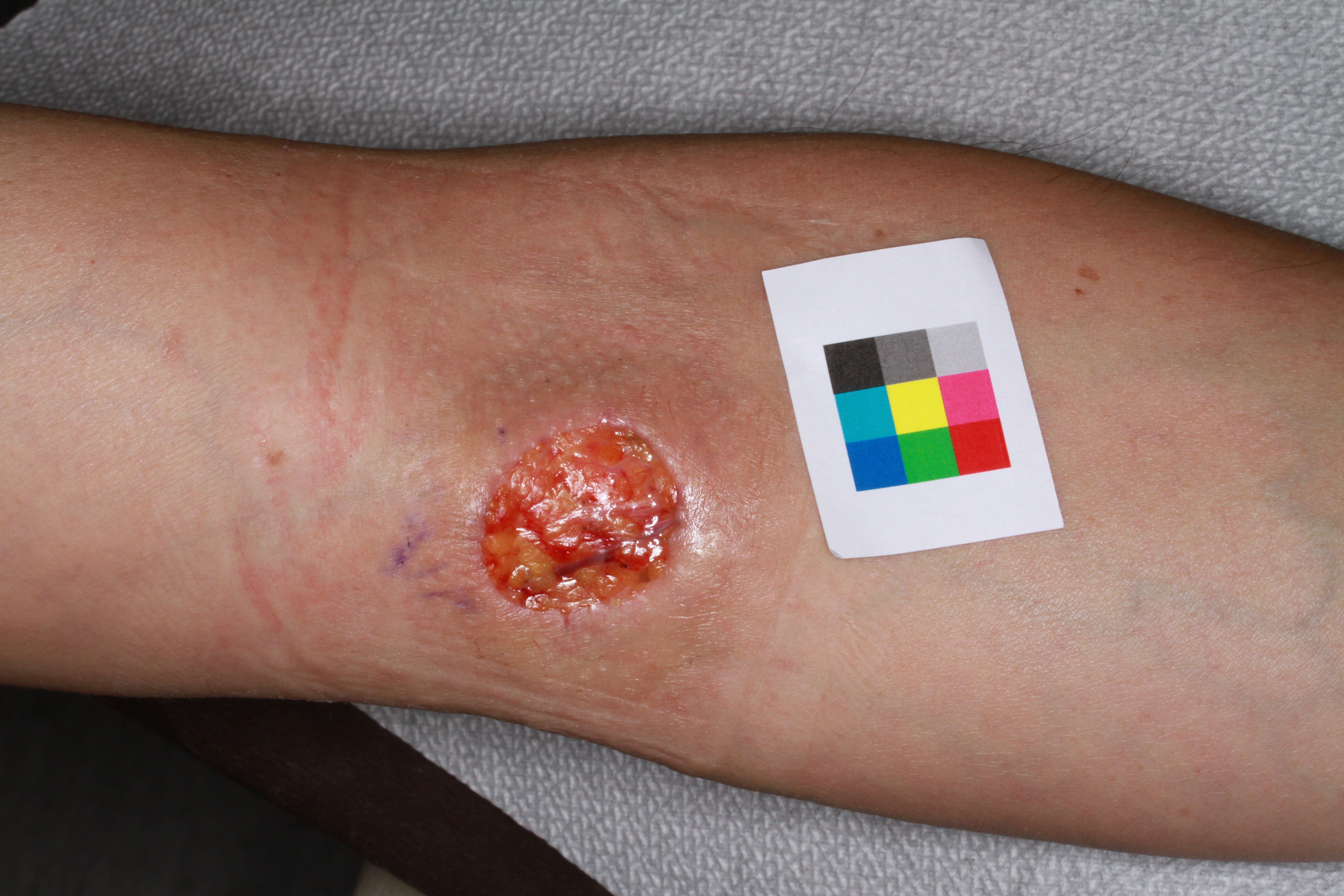

Supplement: S9 File — (ZIP) [file pone.0163092.s009.zip › 0906.JPG]

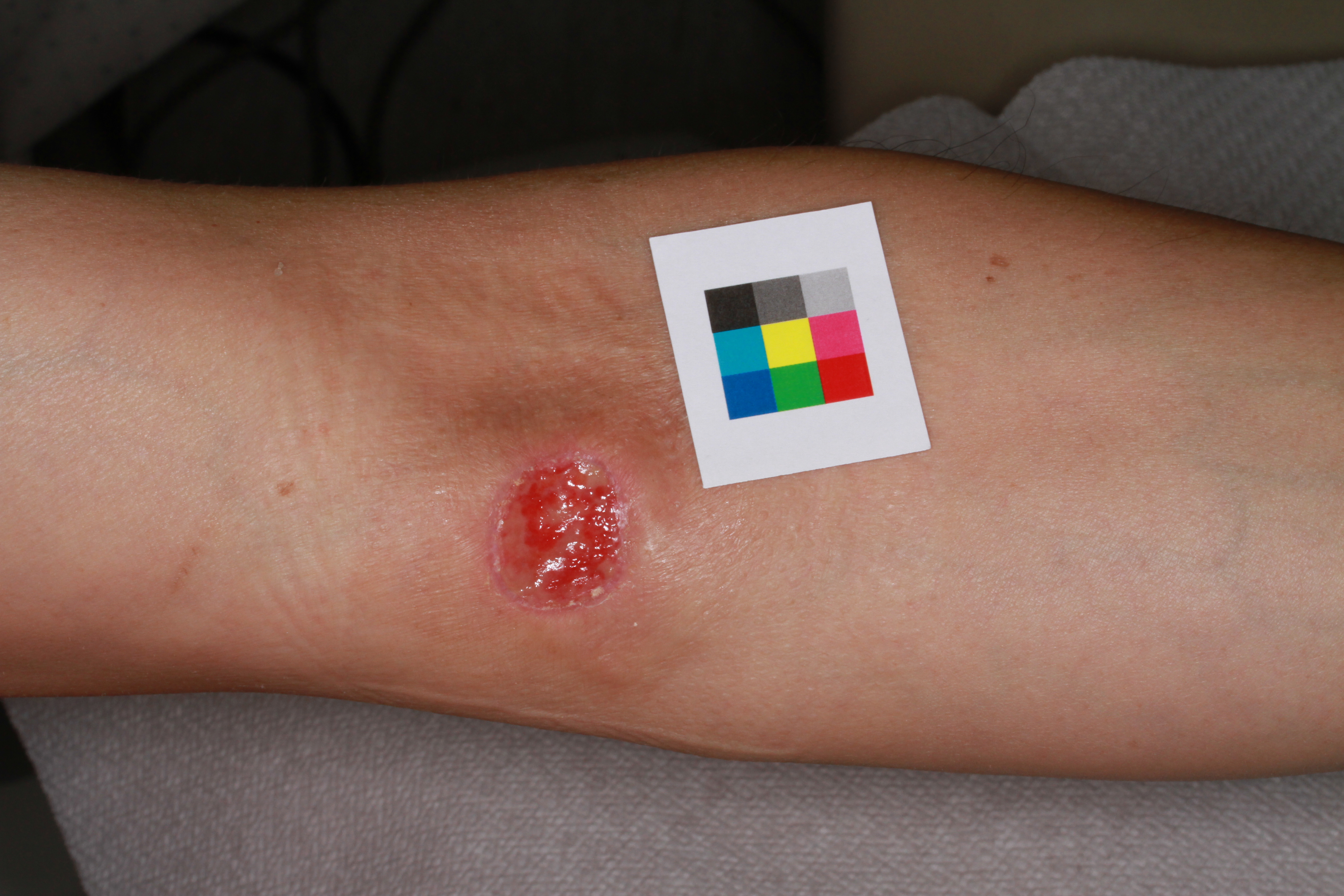

Supplement: S9 File — (ZIP) [file pone.0163092.s009.zip › 0917.JPG]

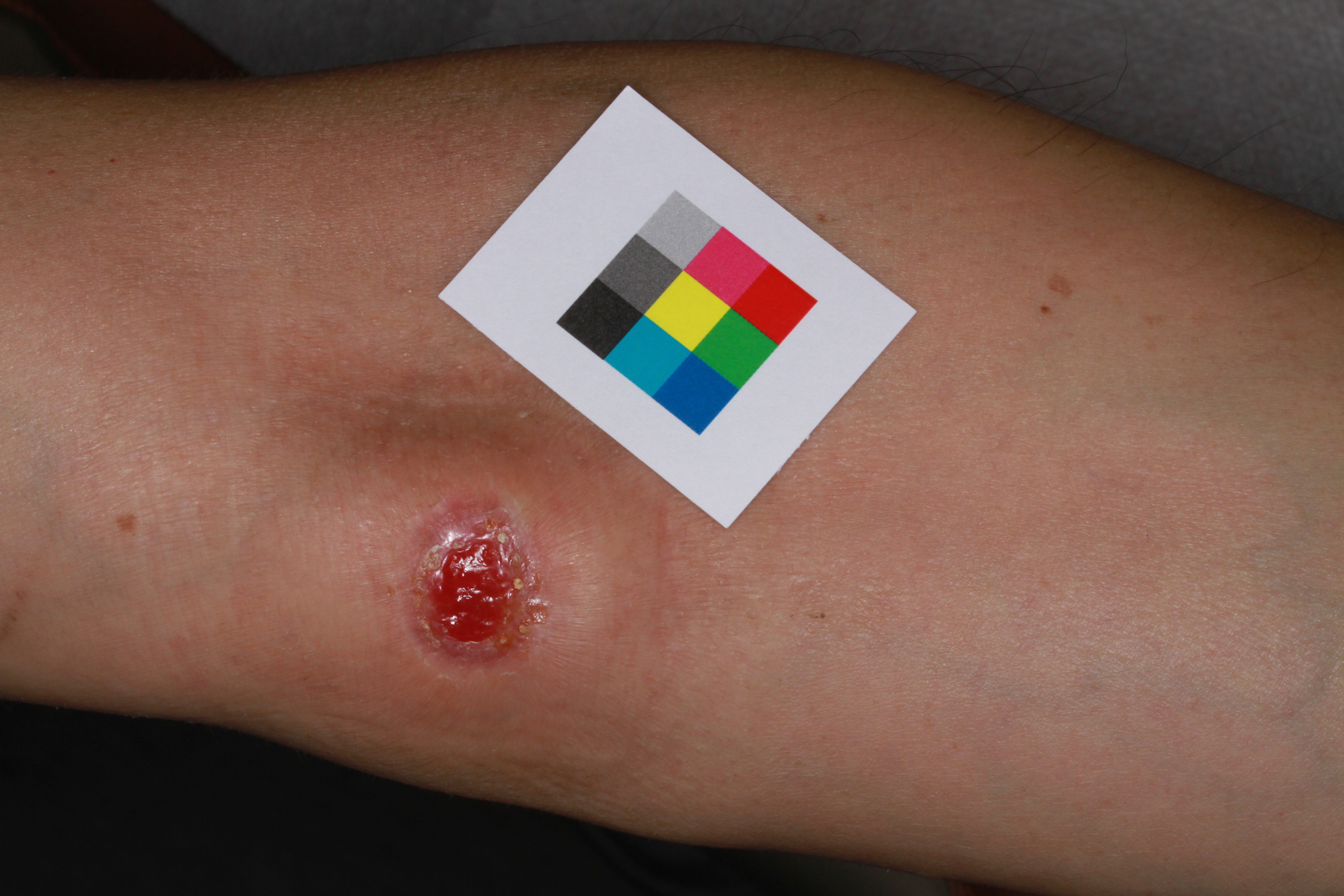

Supplement: S9 File — (ZIP) [file pone.0163092.s009.zip › 0924.JPG]

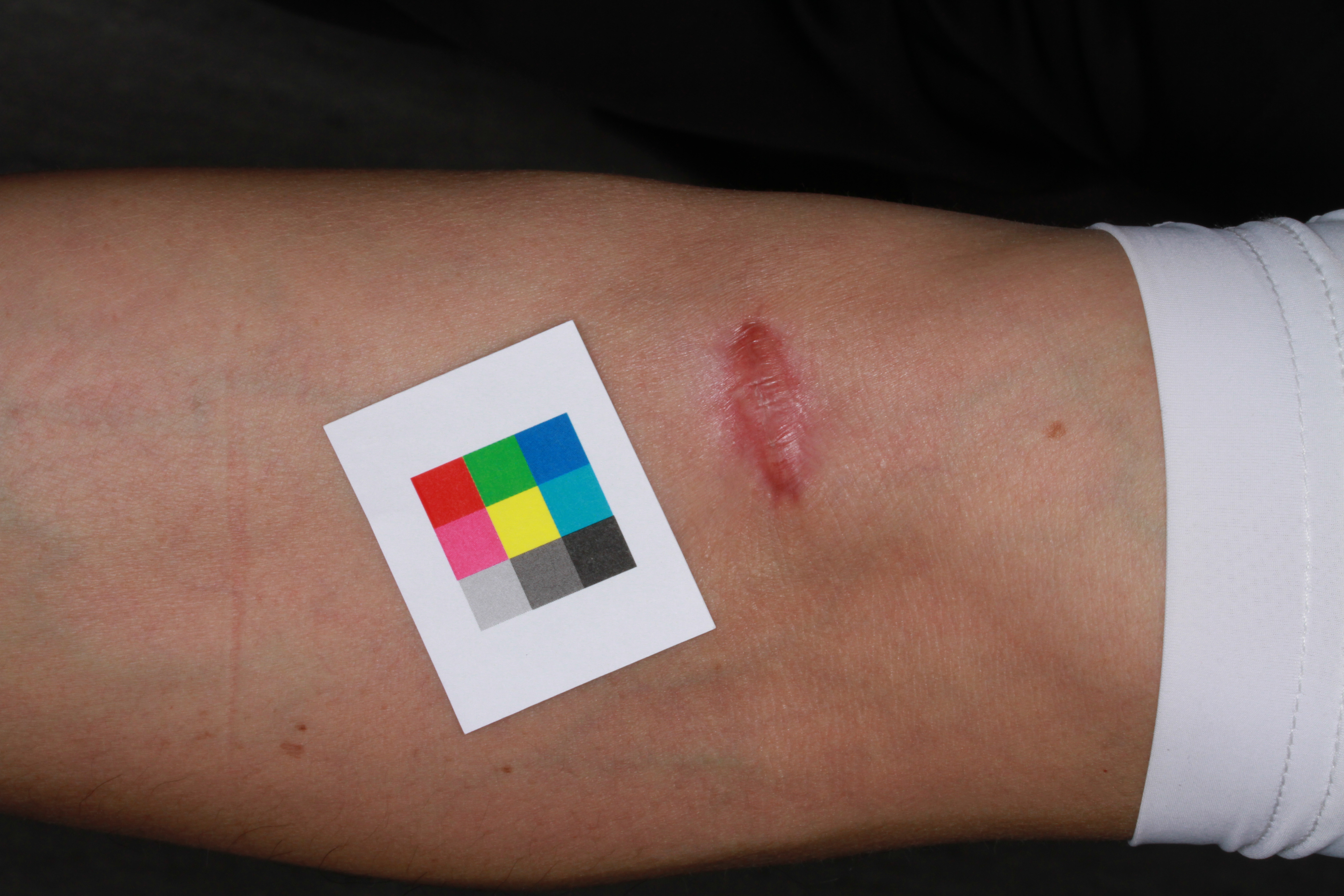

Supplement: S9 File — (ZIP) [file pone.0163092.s009.zip › 1022.JPG]

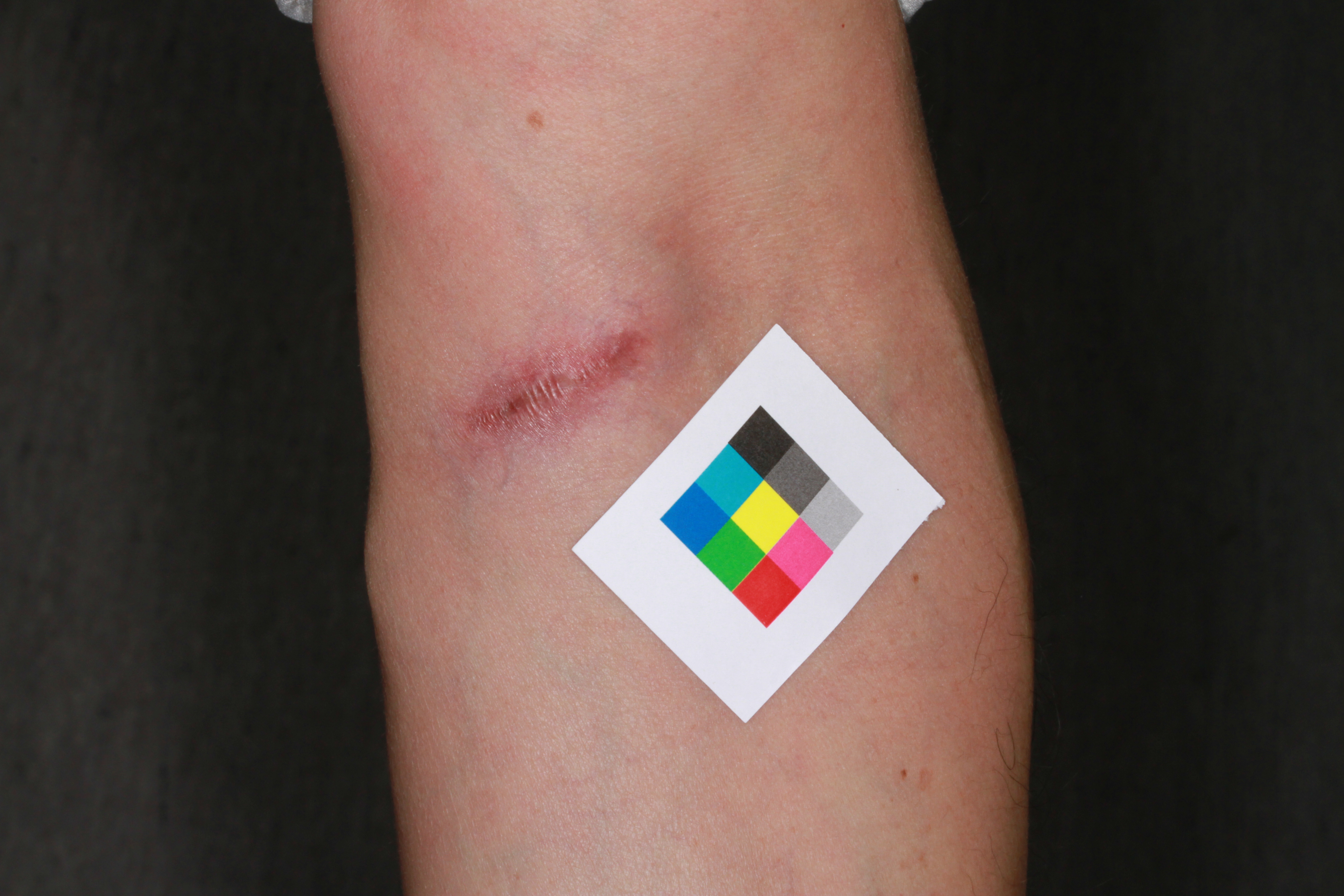

Supplement: S9 File — (ZIP) [file pone.0163092.s009.zip › 1126.JPG]

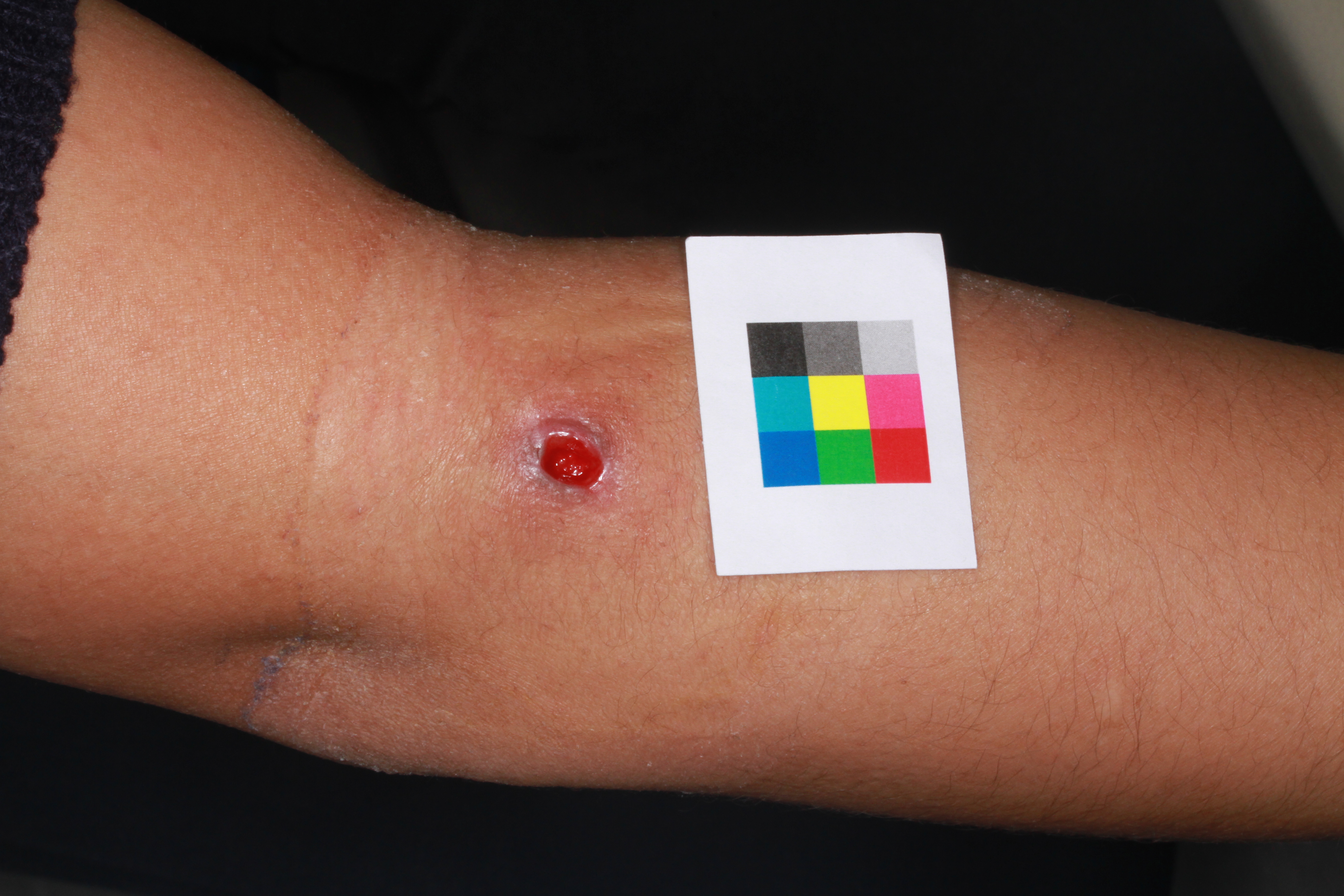

Supplement: S10 File — (ZIP) [file pone.0163092.s010.zip › 1128.JPG]

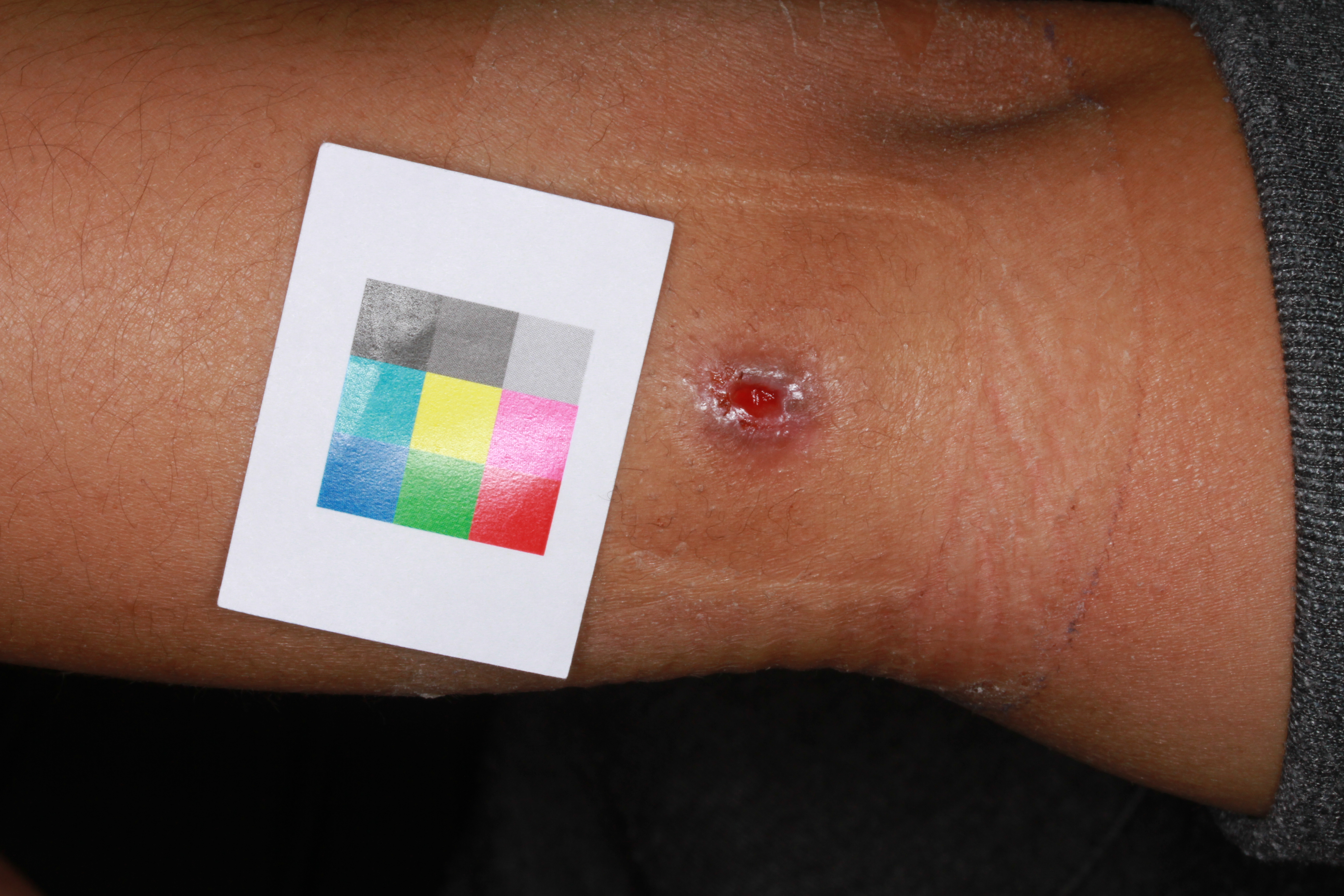

Supplement: S10 File — (ZIP) [file pone.0163092.s010.zip › 1130.JPG]

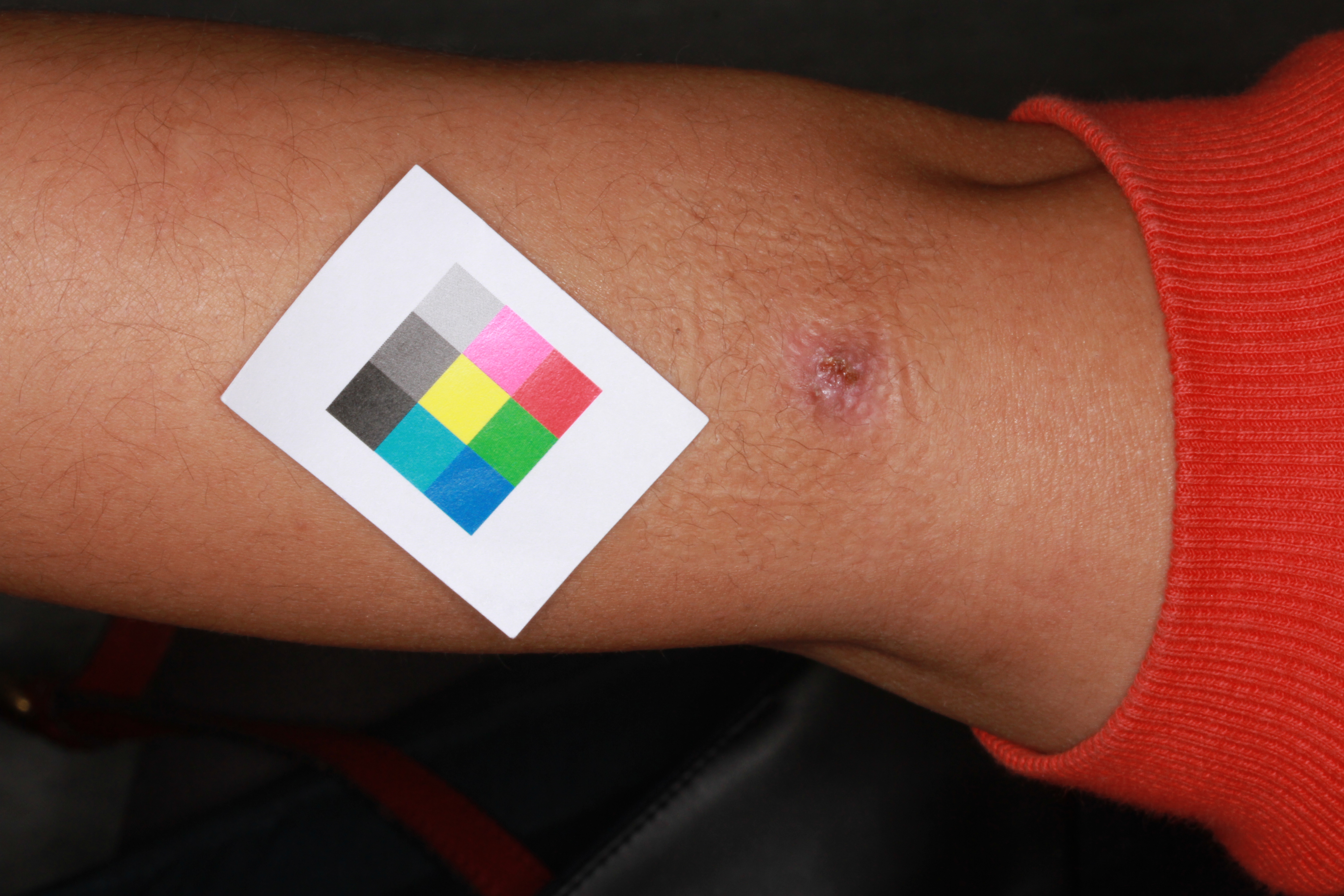

Supplement: S10 File — (ZIP) [file pone.0163092.s010.zip › 1220.JPG]

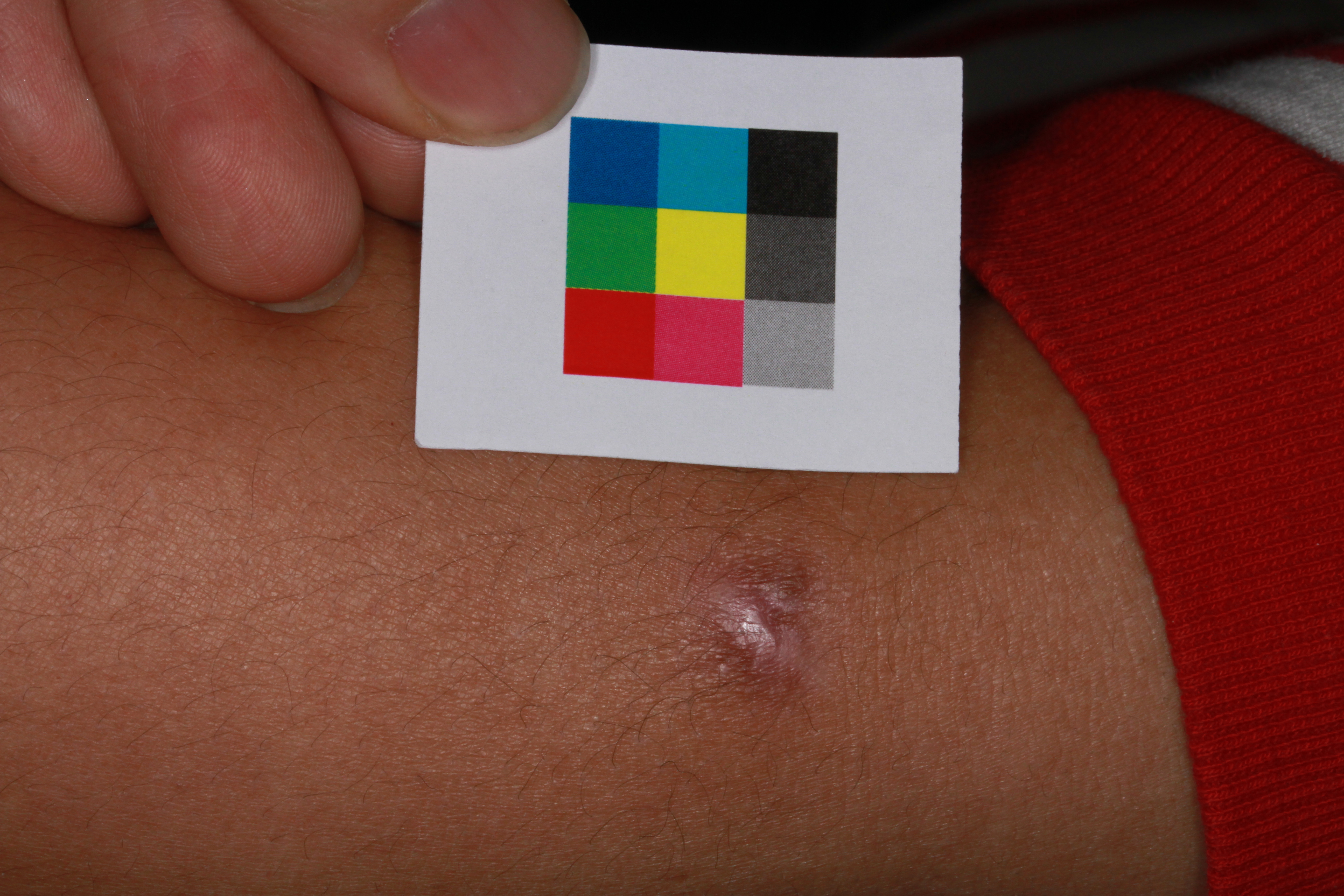

Supplement: S10 File — (ZIP) [file pone.0163092.s010.zip › 40124.JPG]

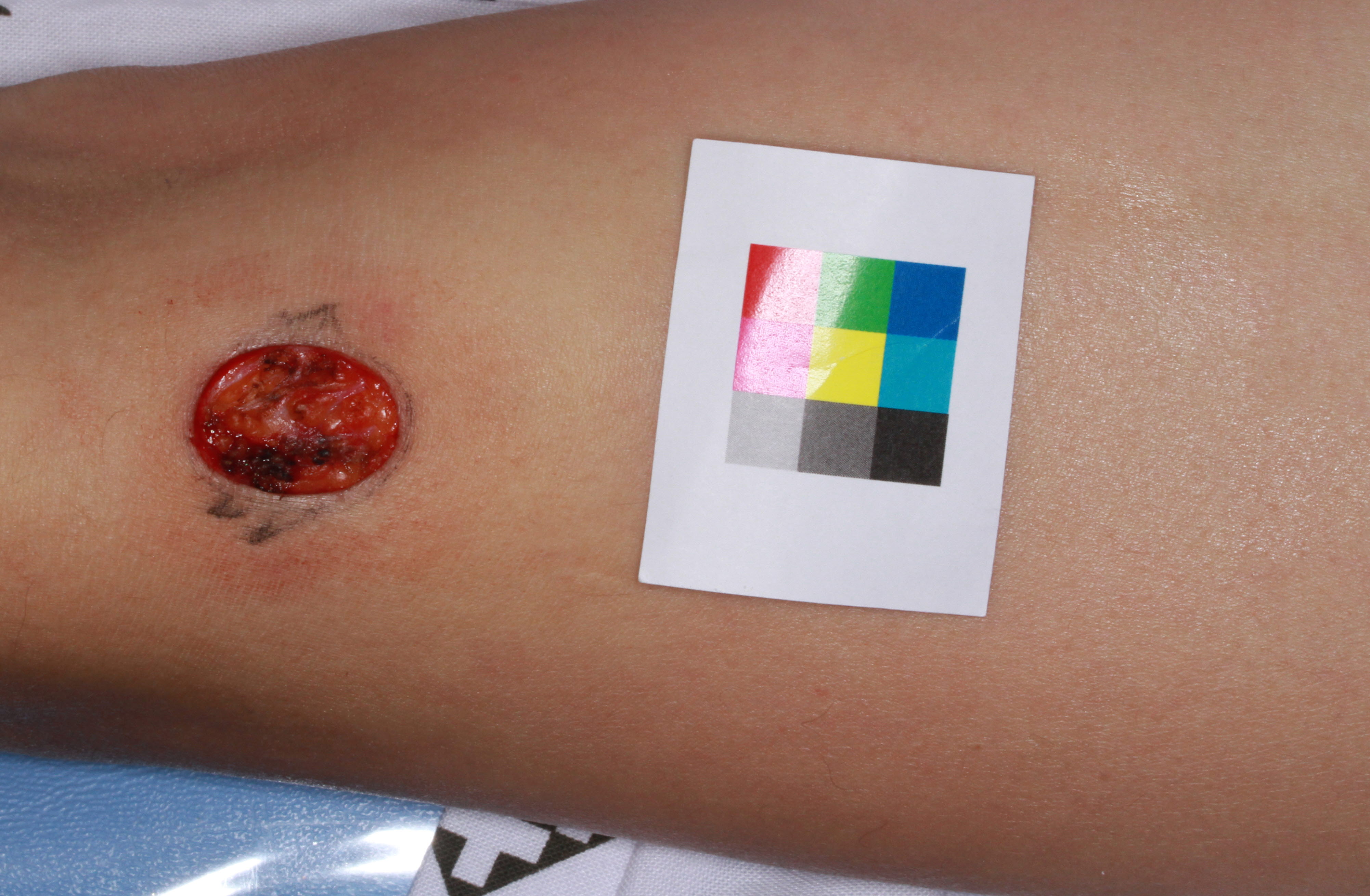

Supplement: S11 File — (ZIP) [file pone.0163092.s011.zip › 31022.jpg]

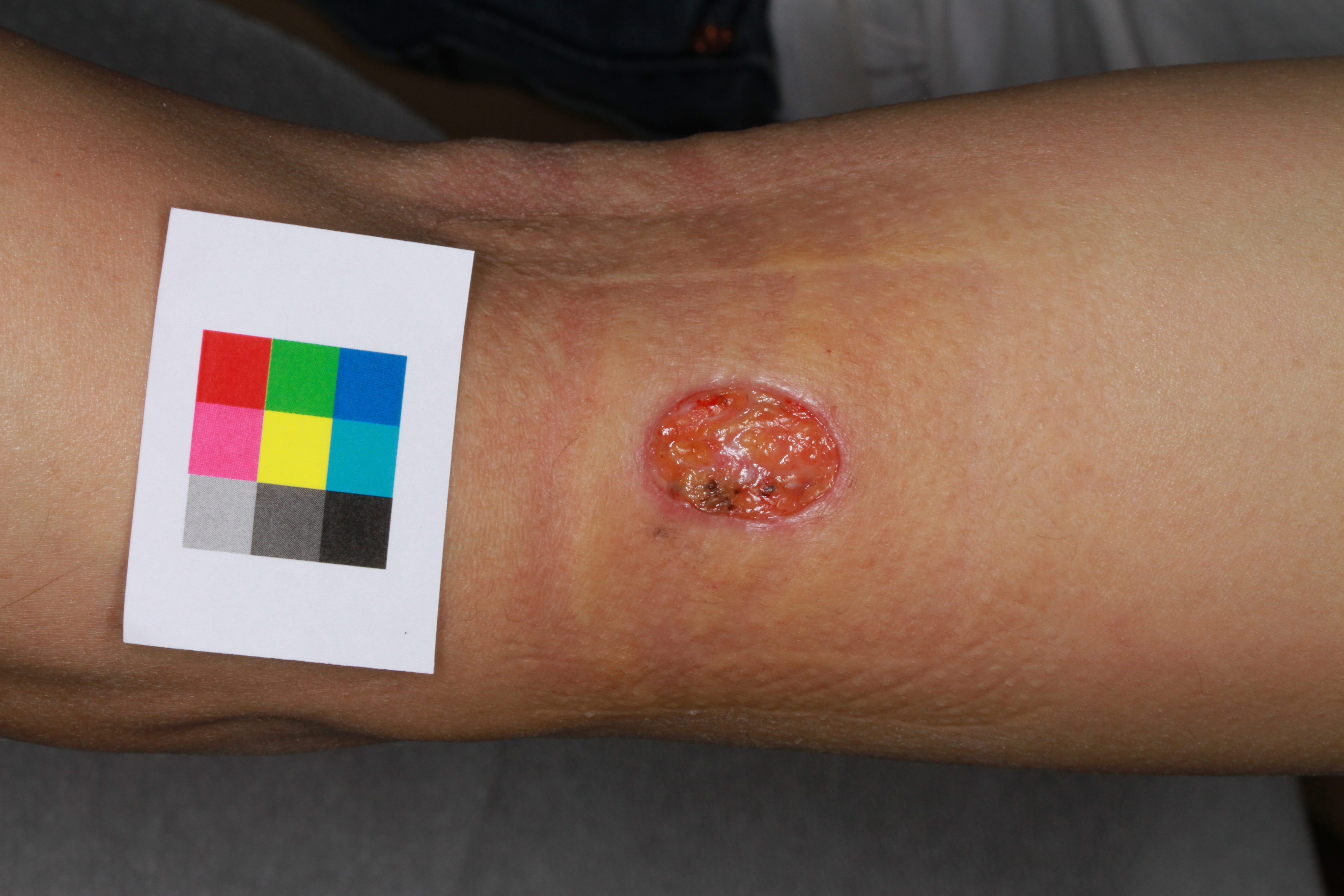

Supplement: S11 File — (ZIP) [file pone.0163092.s011.zip › 31026.JPG]

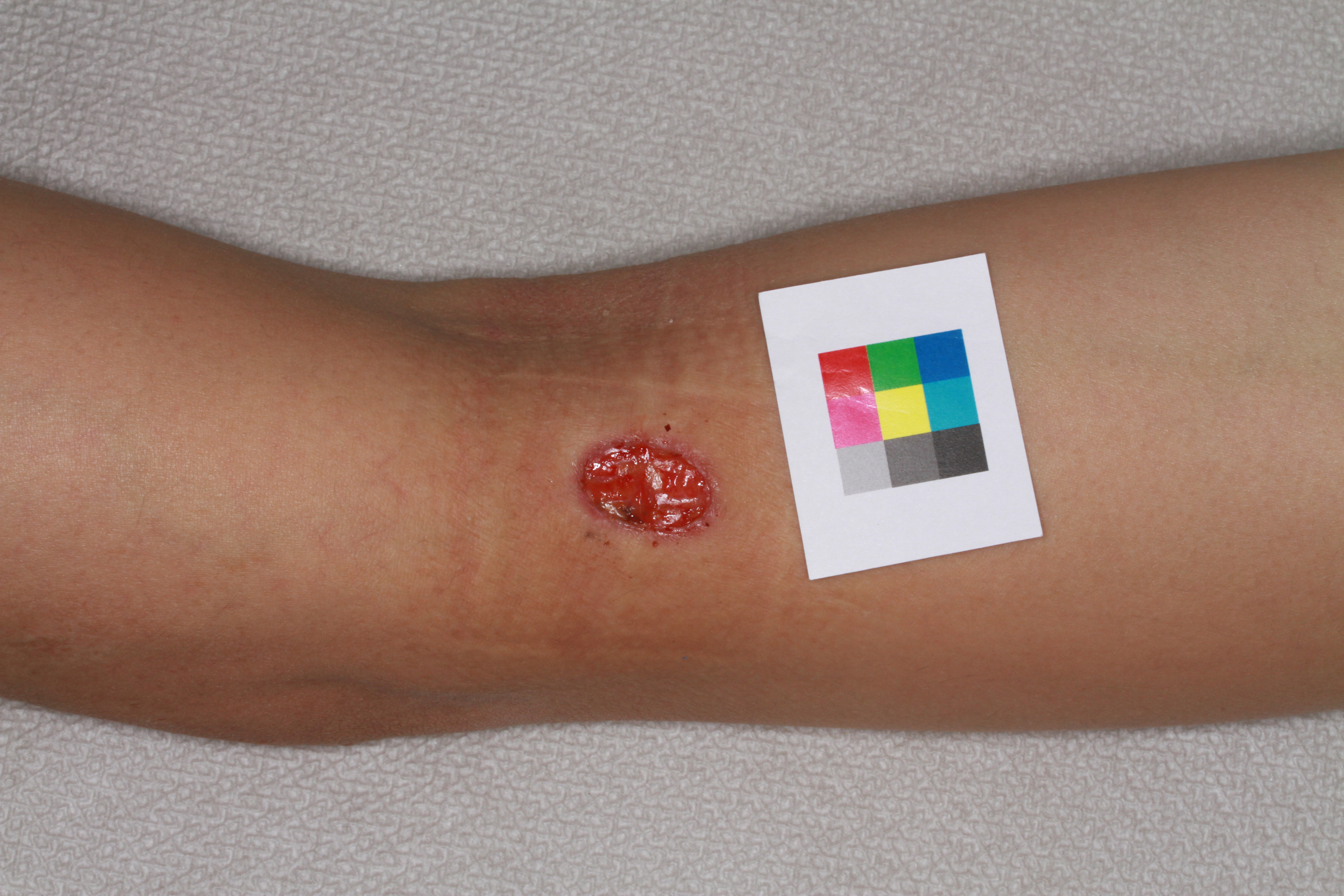

Supplement: S11 File — (ZIP) [file pone.0163092.s011.zip › 31031.JPG]

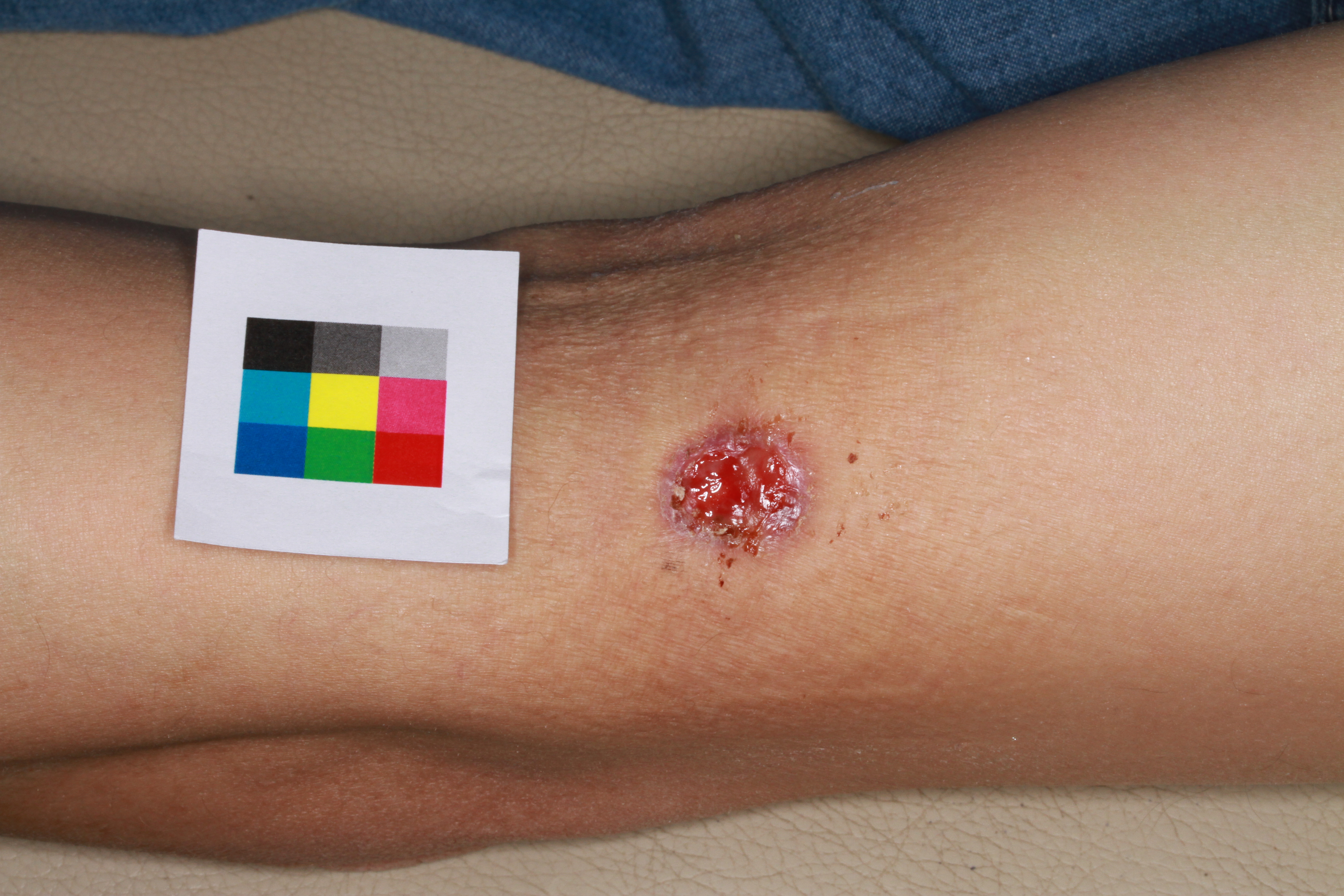

Supplement: S11 File — (ZIP) [file pone.0163092.s011.zip › 31107.JPG]

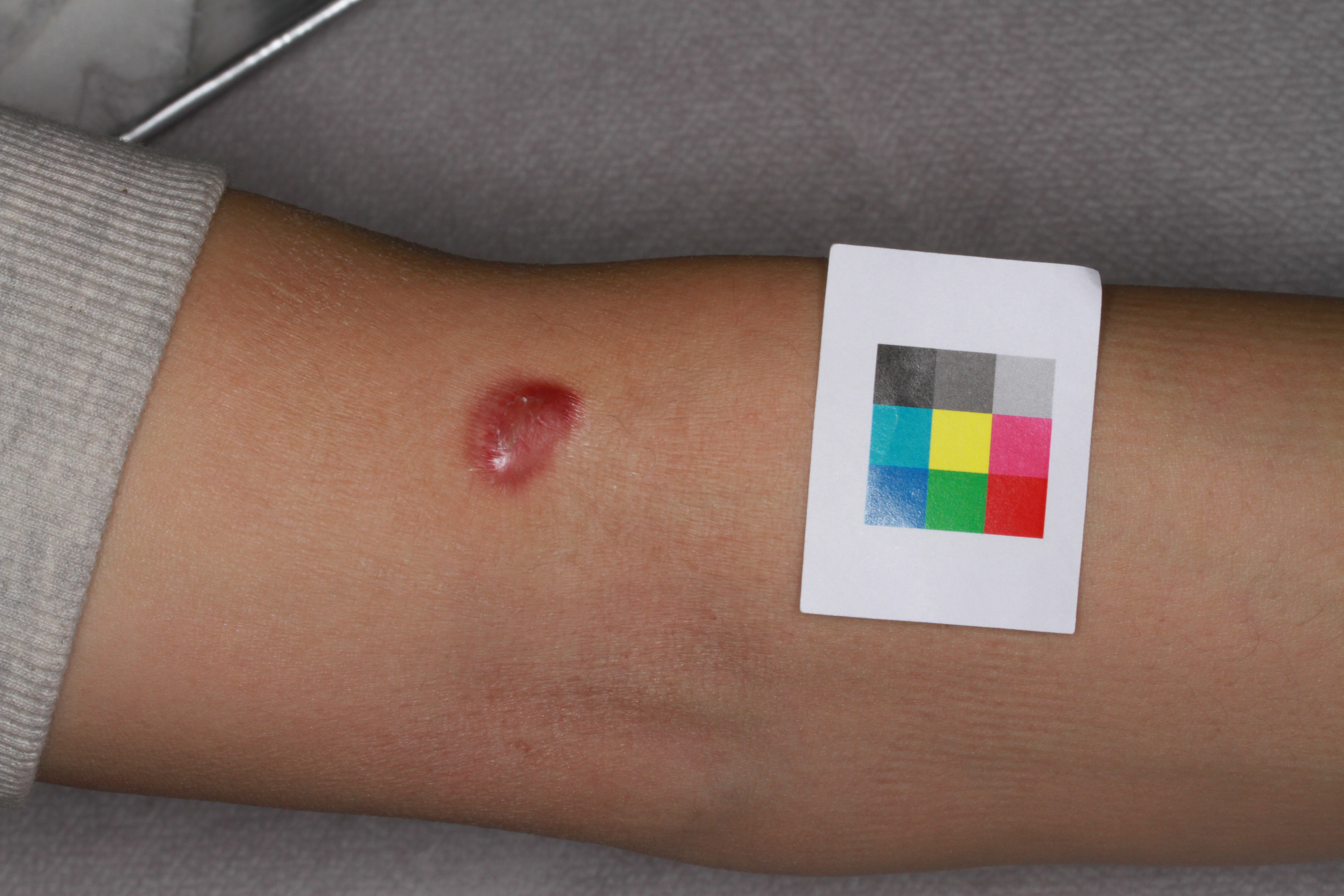

Supplement: S11 File — (ZIP) [file pone.0163092.s011.zip › 31205.JPG]

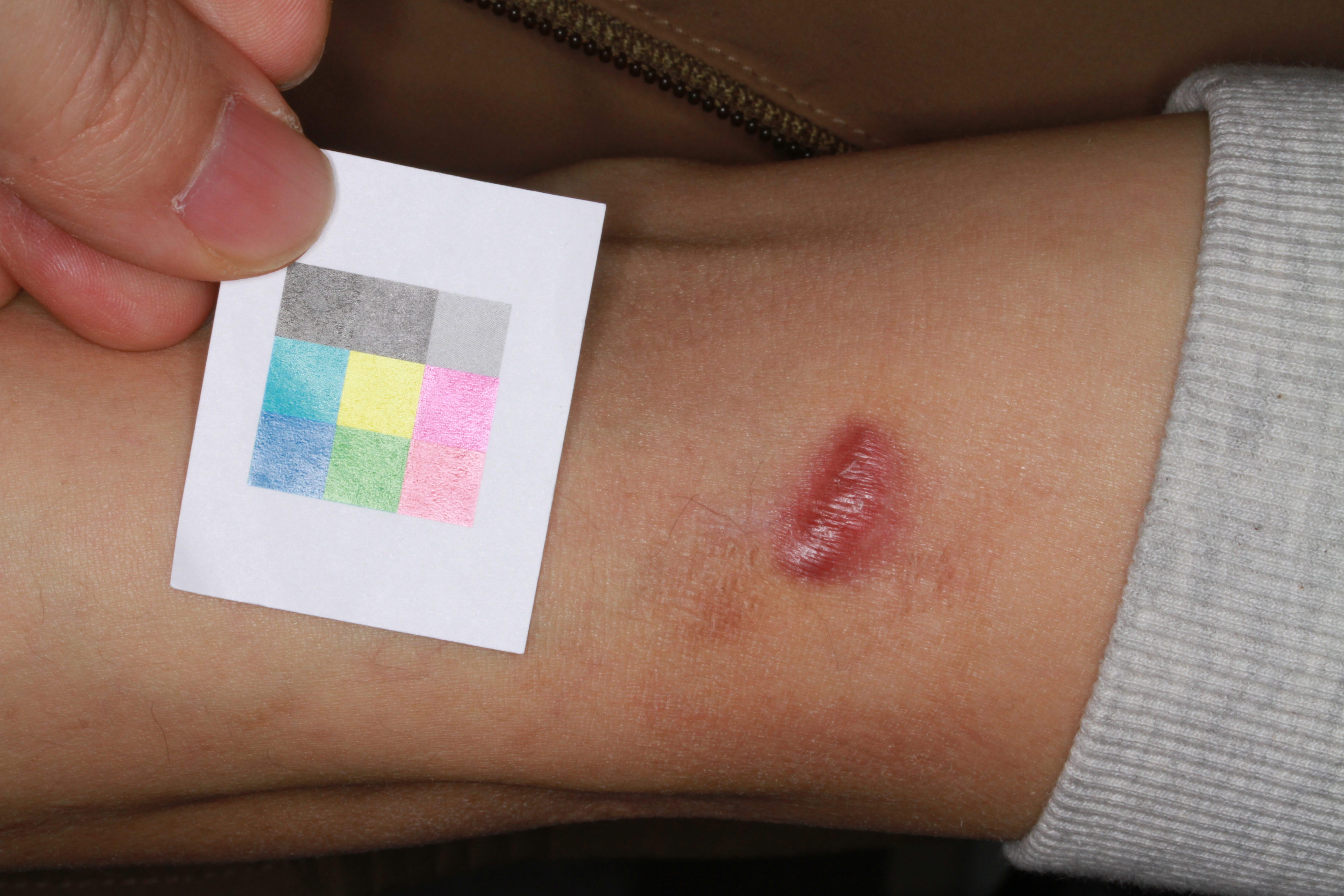

Supplement: S11 File — (ZIP) [file pone.0163092.s011.zip › 40103.JPG]

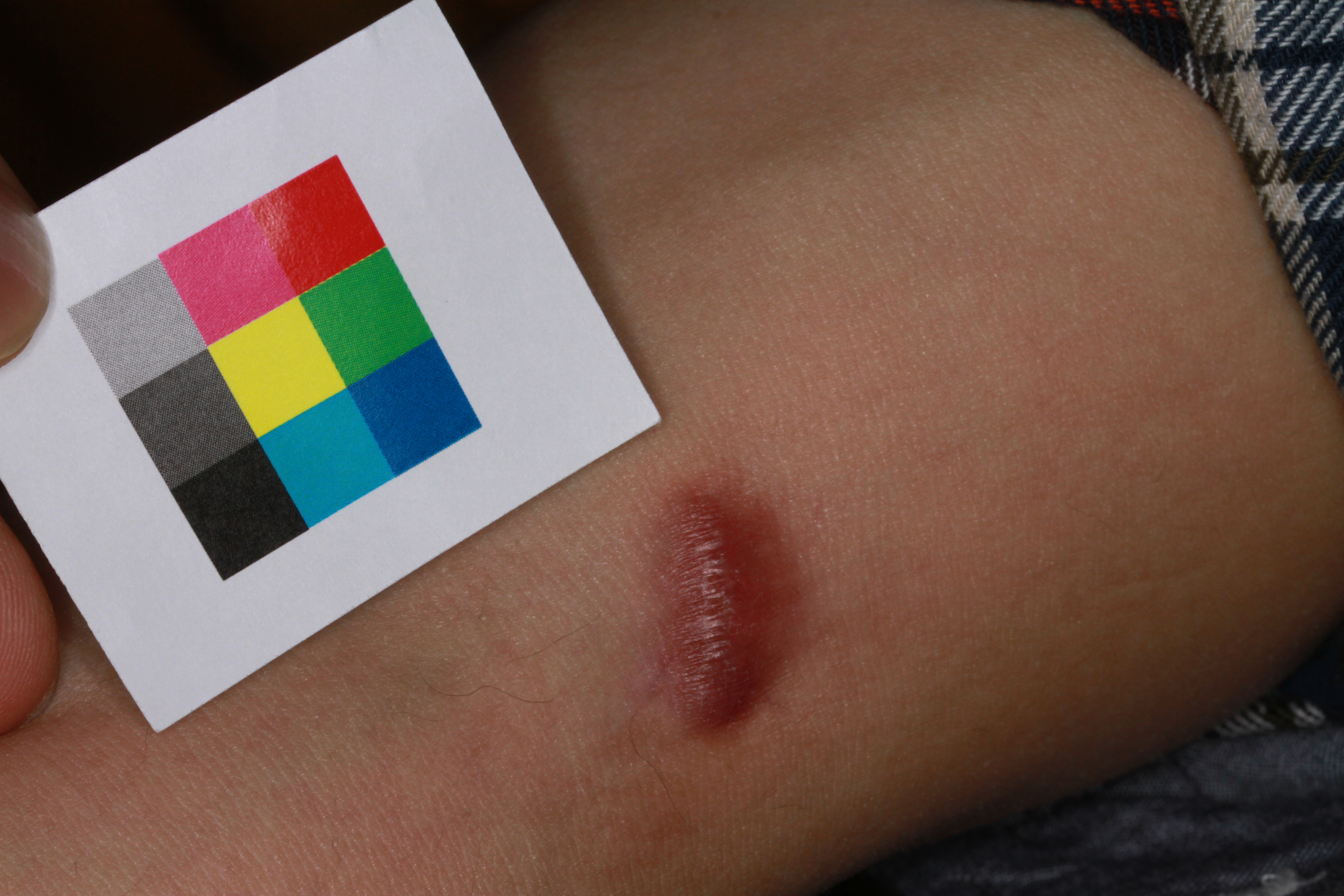

Supplement: S11 File — (ZIP) [file pone.0163092.s011.zip › 40207.JPG]

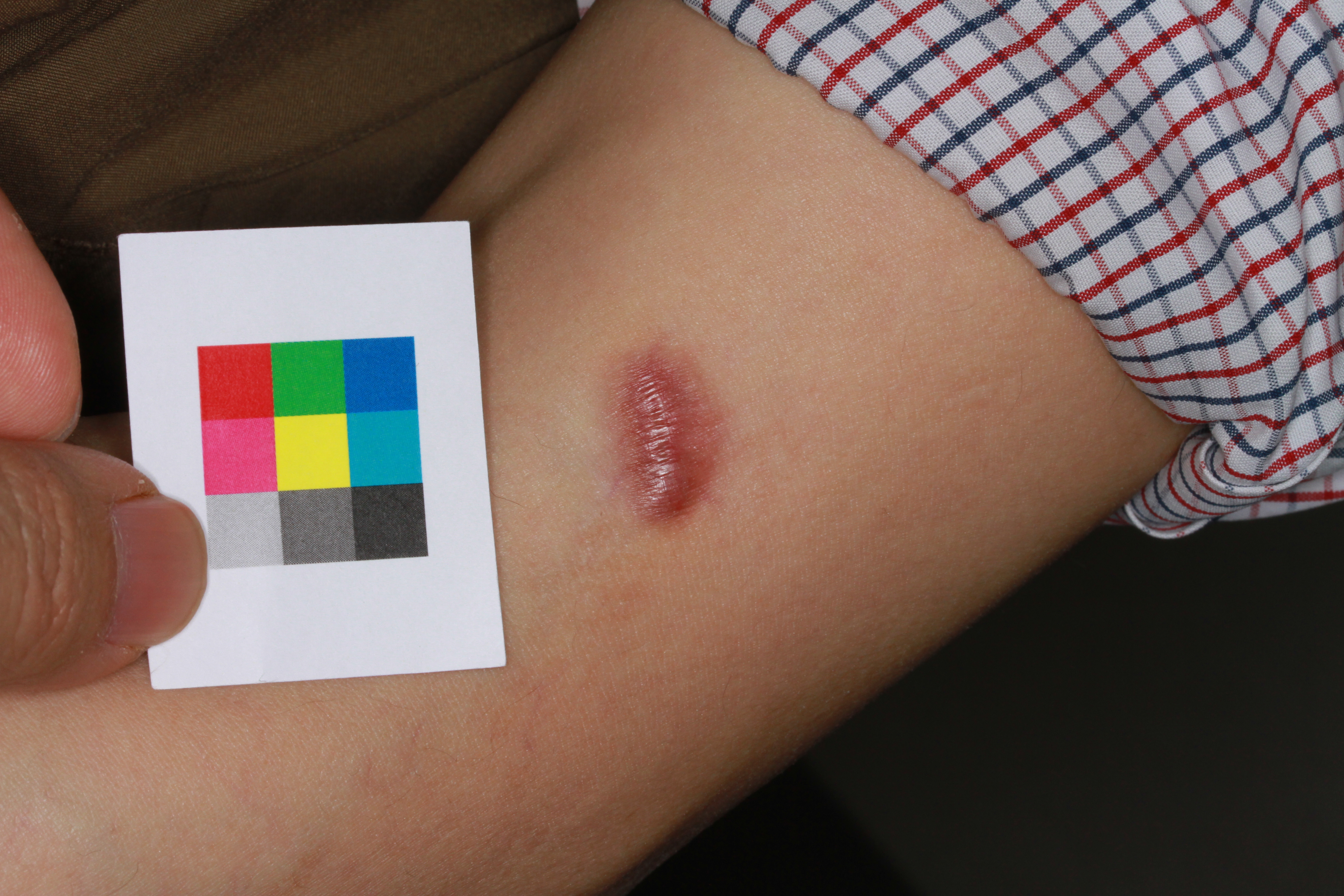

Supplement: S11 File — (ZIP) [file pone.0163092.s011.zip › 40311.JPG]

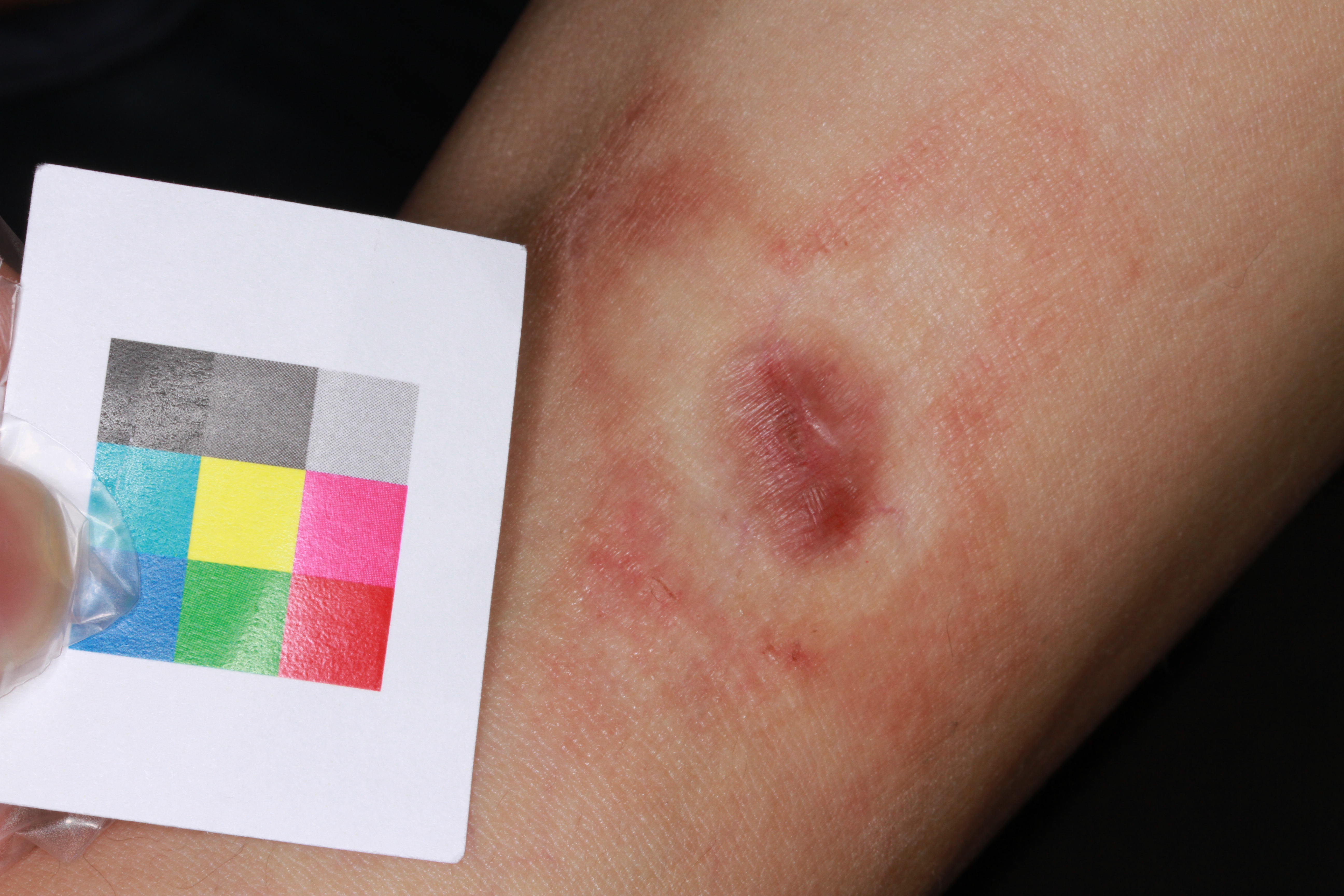

Supplement: S11 File — (ZIP) [file pone.0163092.s011.zip › 40408.JPG]

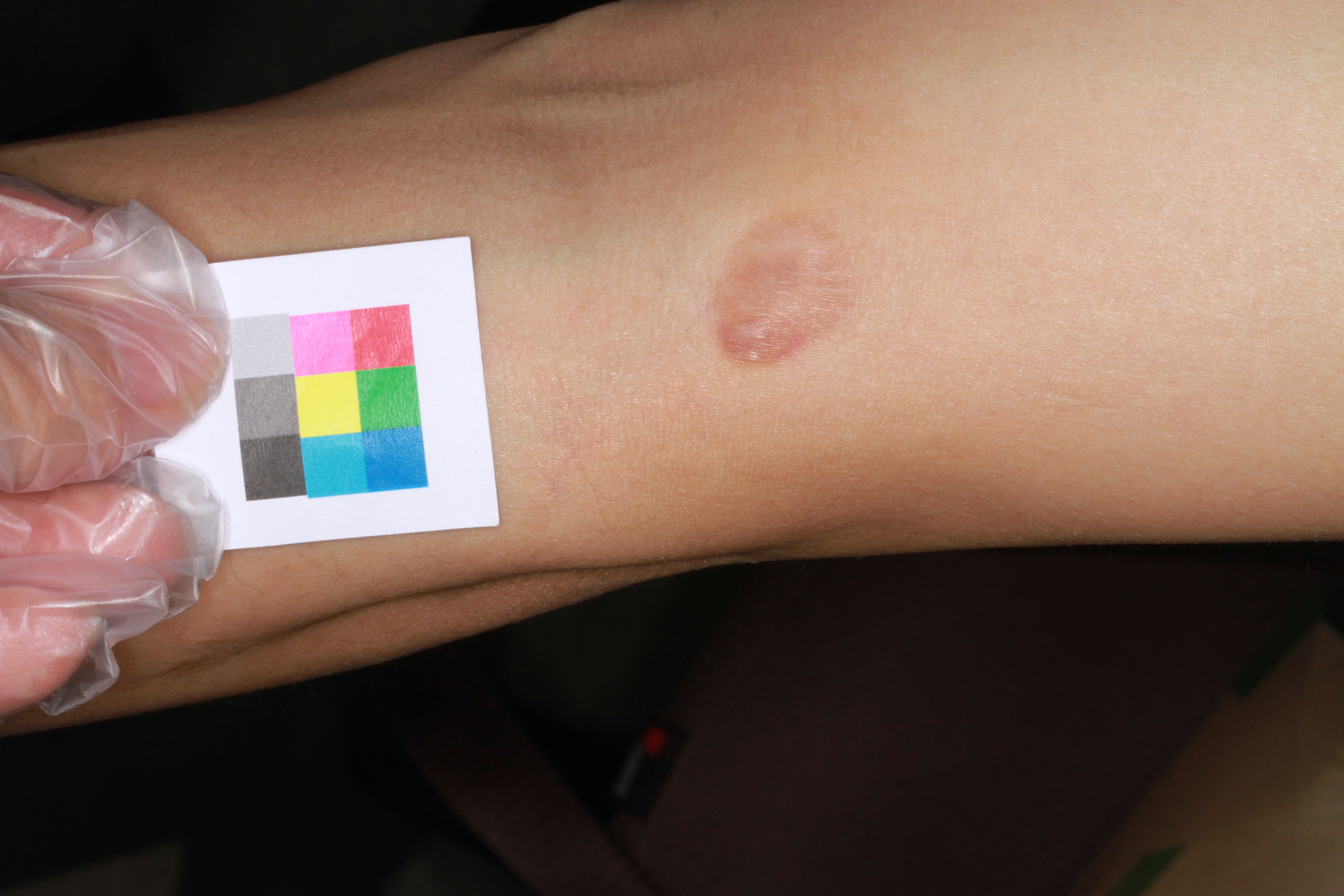

Supplement: S11 File — (ZIP) [file pone.0163092.s011.zip › 41024.JPG]

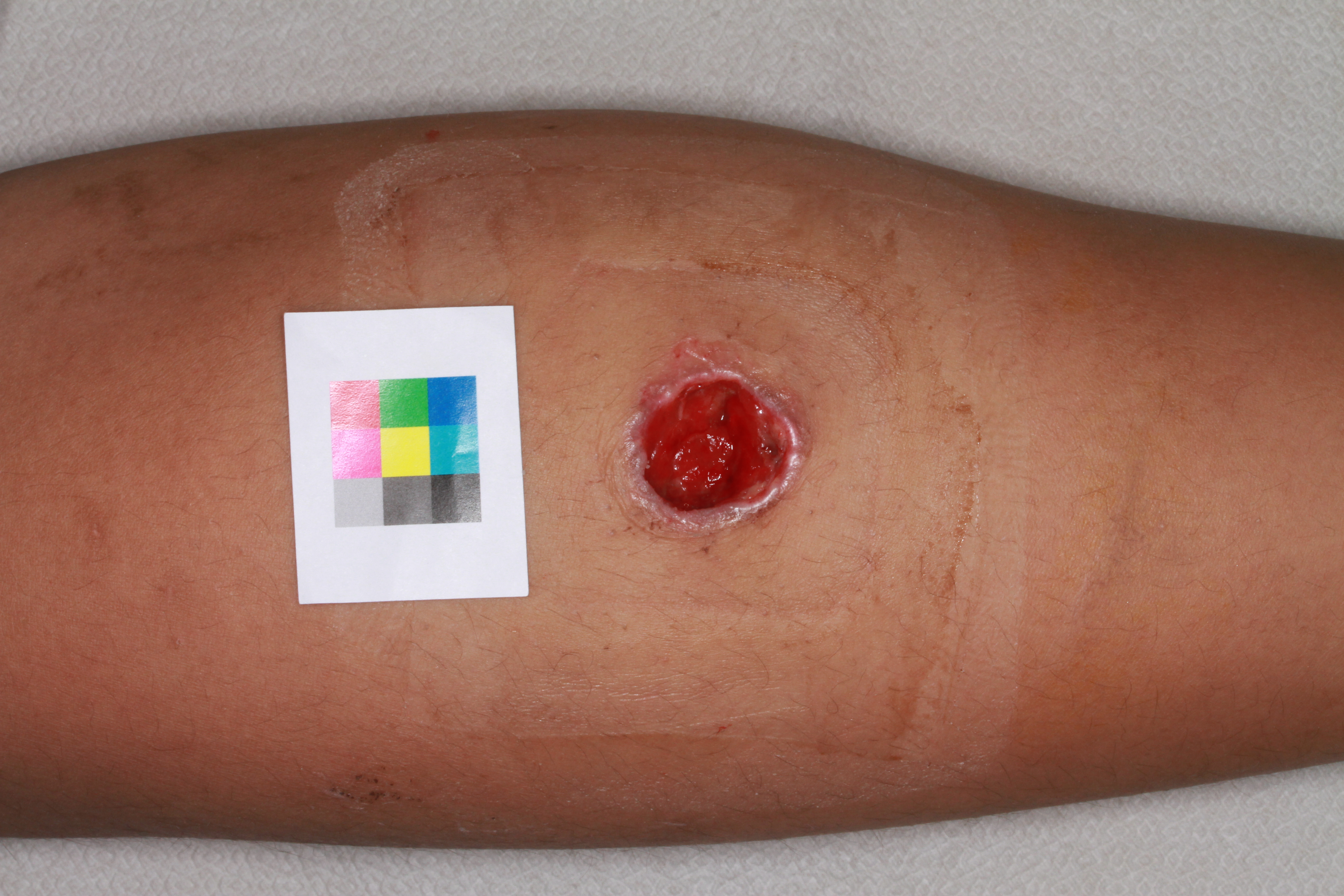

Supplement: S12 File — (ZIP) [file pone.0163092.s012.zip › 31005.JPG]

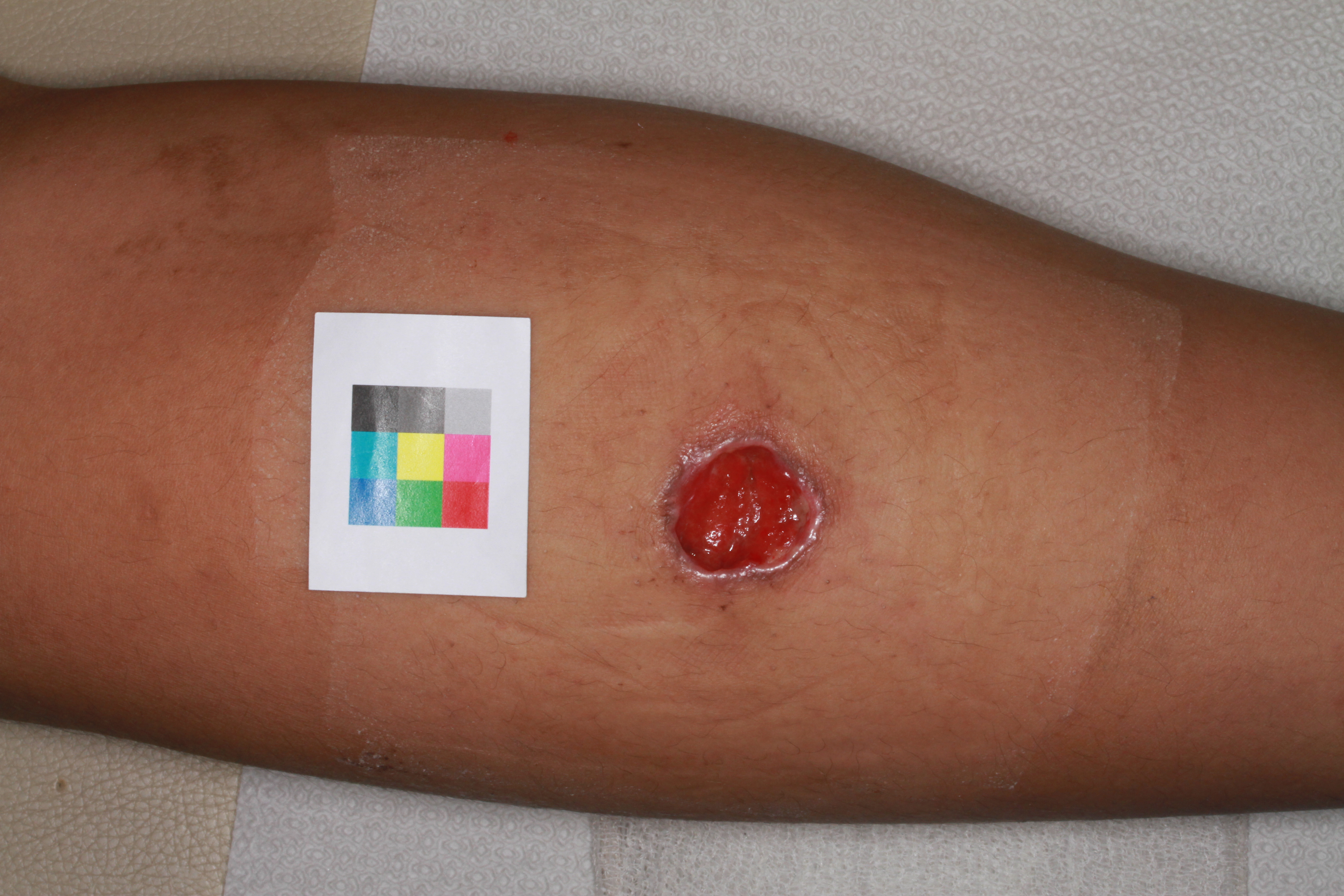

Supplement: S12 File — (ZIP) [file pone.0163092.s012.zip › 31008.JPG]

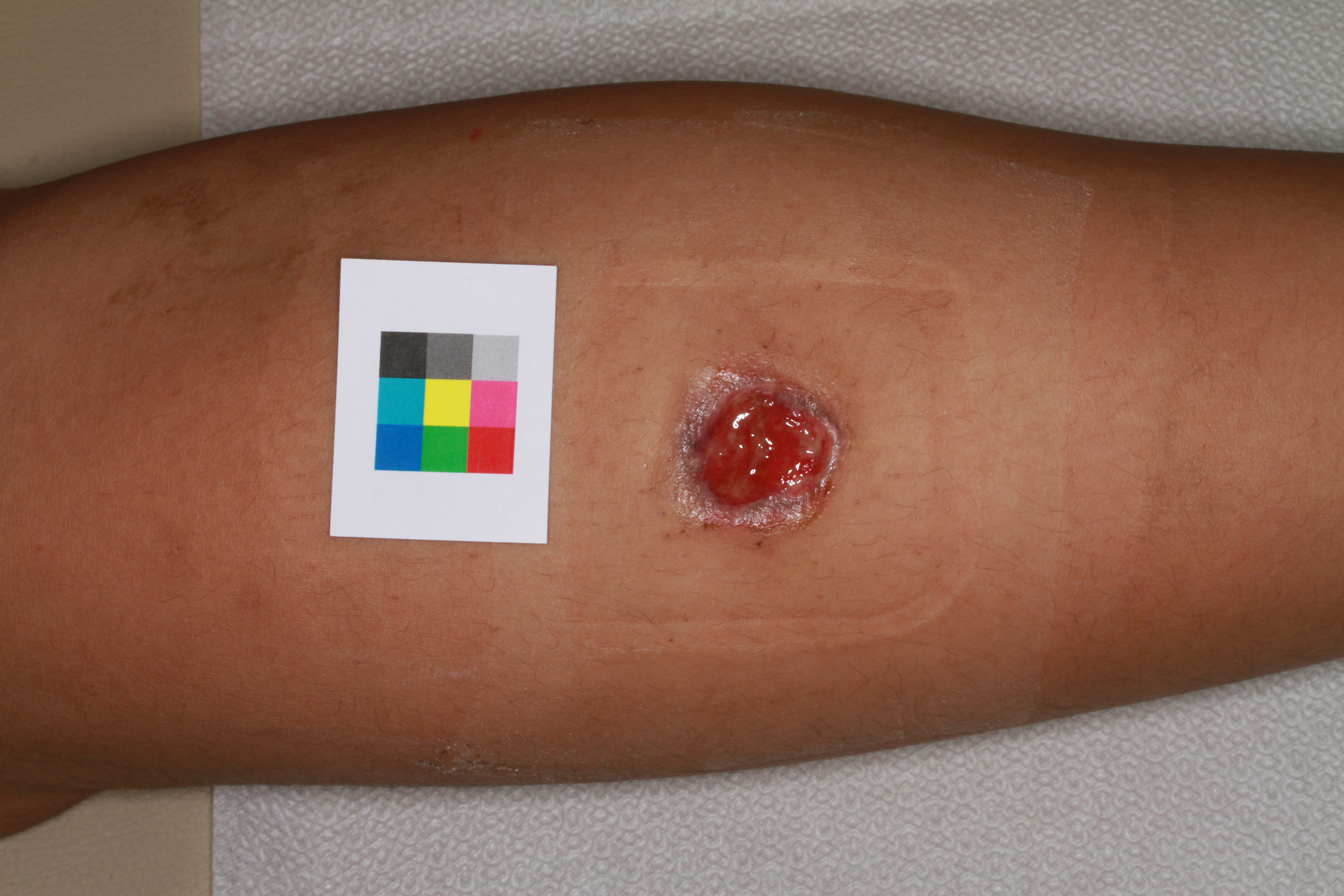

Supplement: S12 File — (ZIP) [file pone.0163092.s012.zip › 31012.JPG]

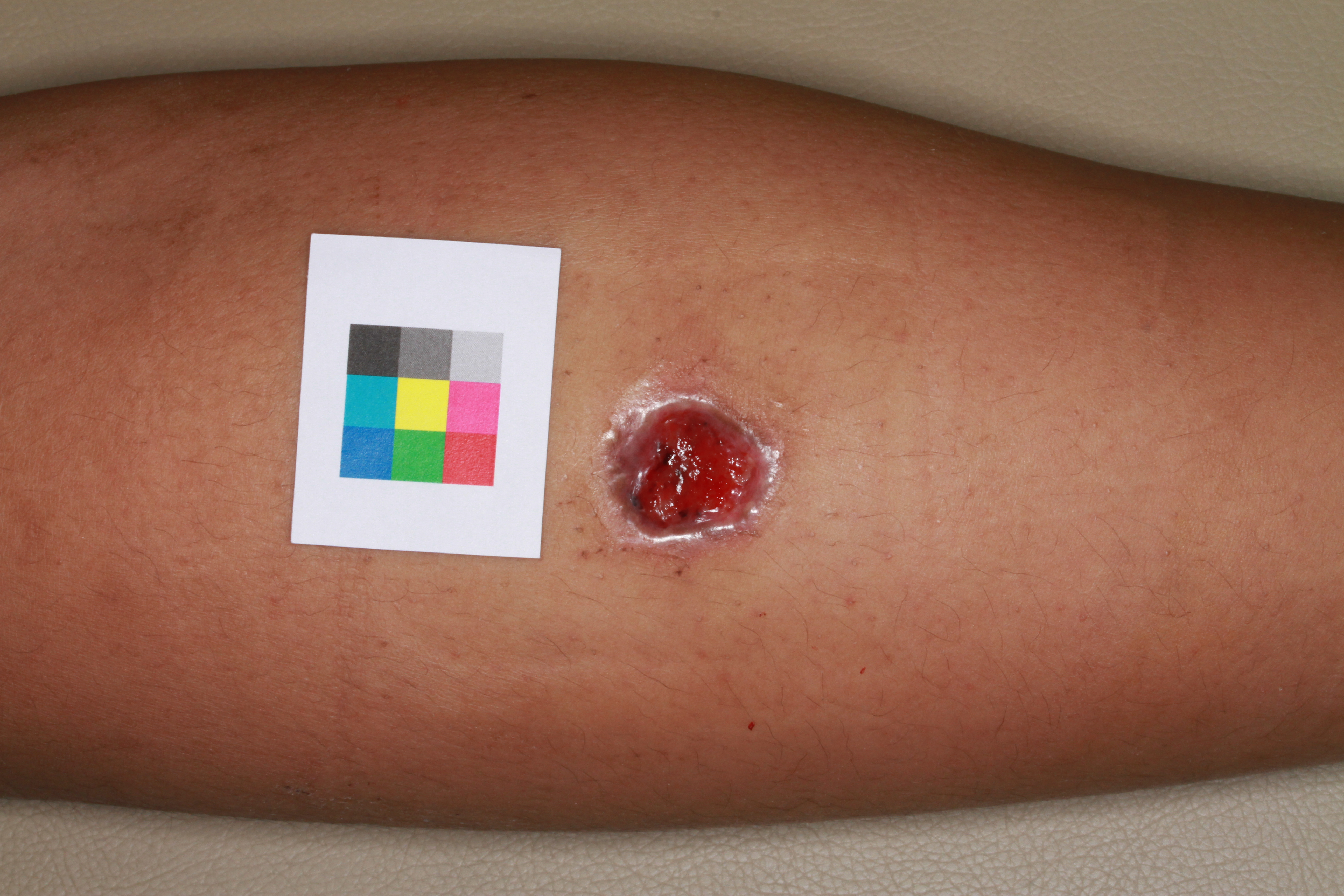

Supplement: S12 File — (ZIP) [file pone.0163092.s012.zip › 31015.JPG]

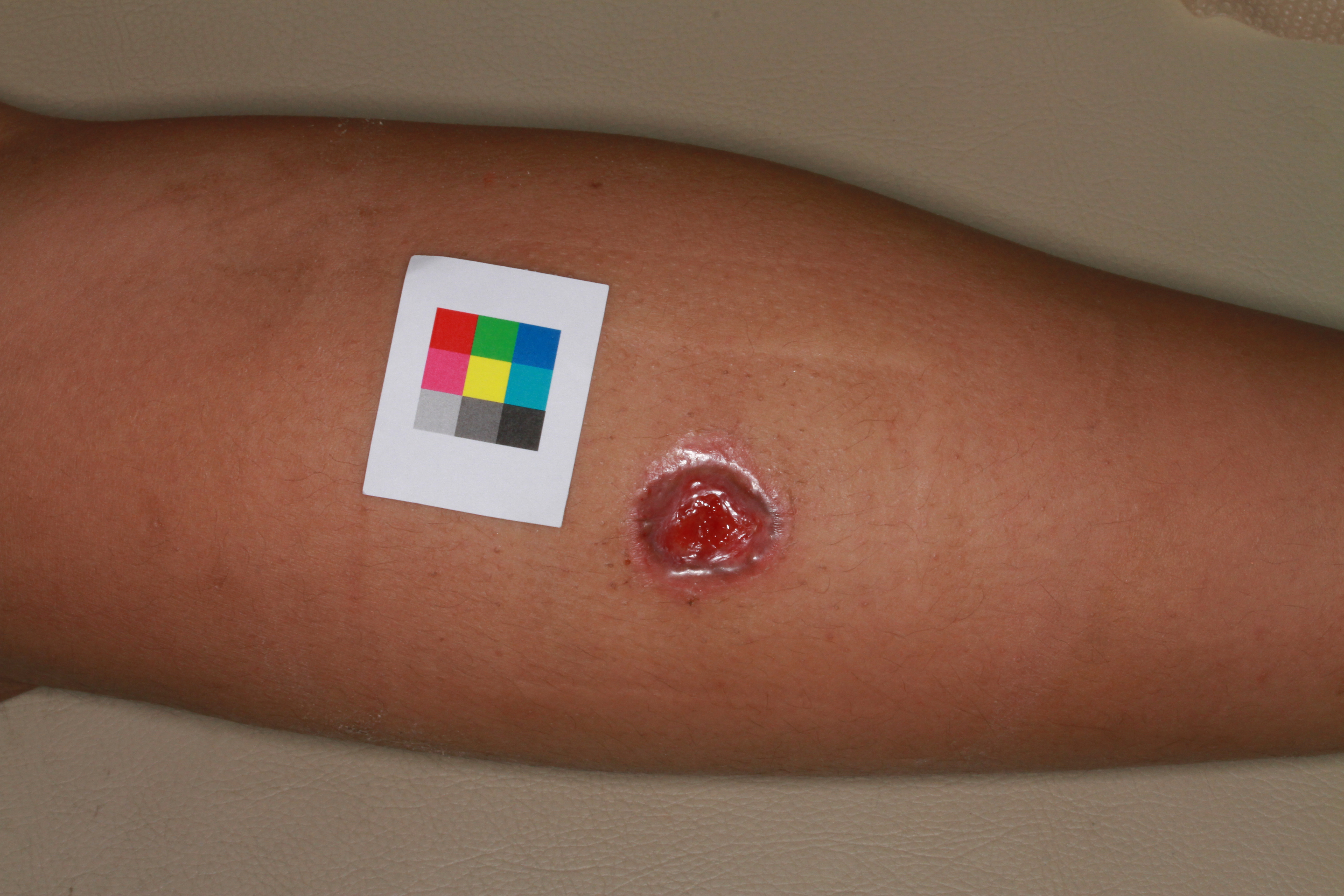

Supplement: S12 File — (ZIP) [file pone.0163092.s012.zip › 31018.JPG]

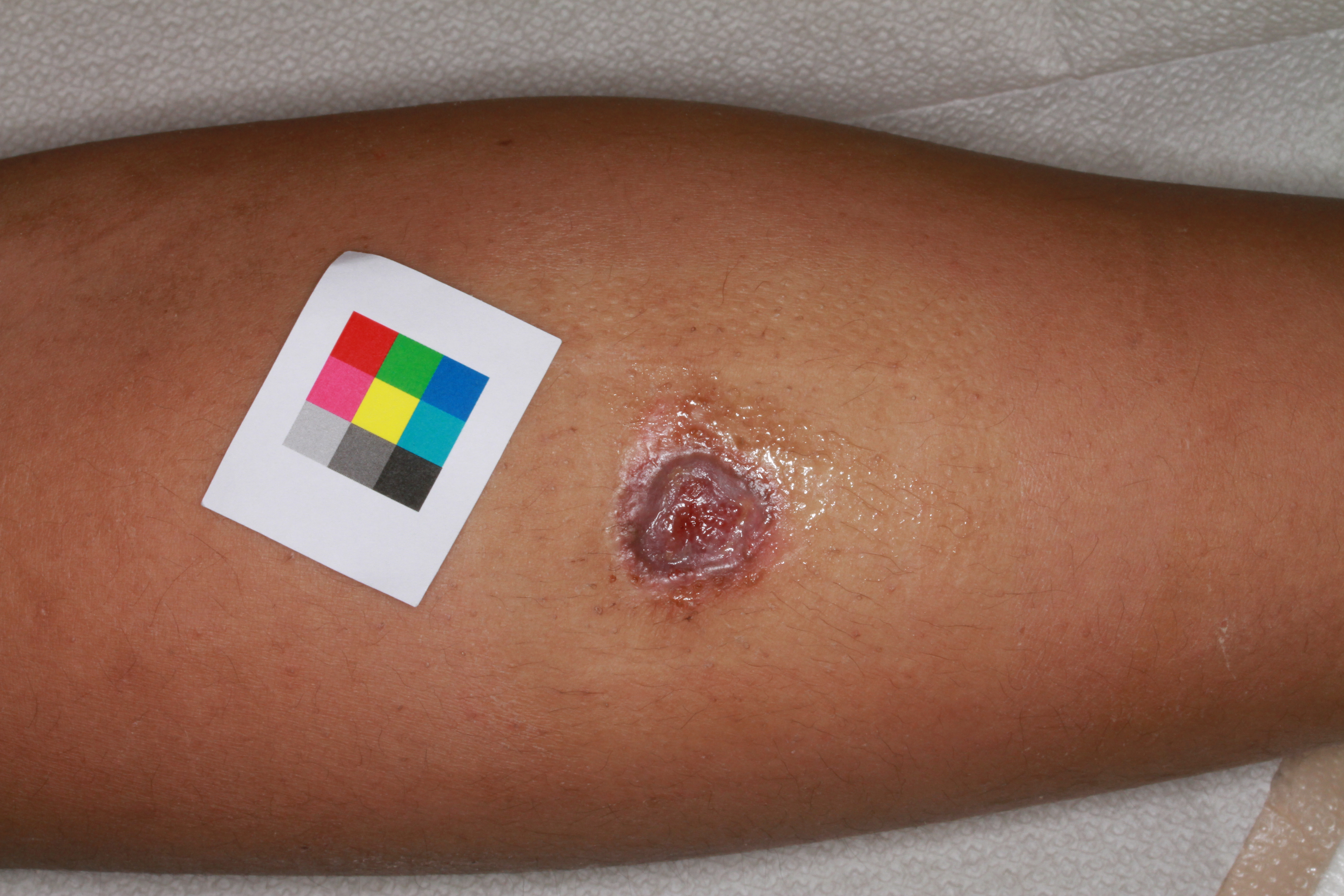

Supplement: S12 File — (ZIP) [file pone.0163092.s012.zip › 31022.JPG]

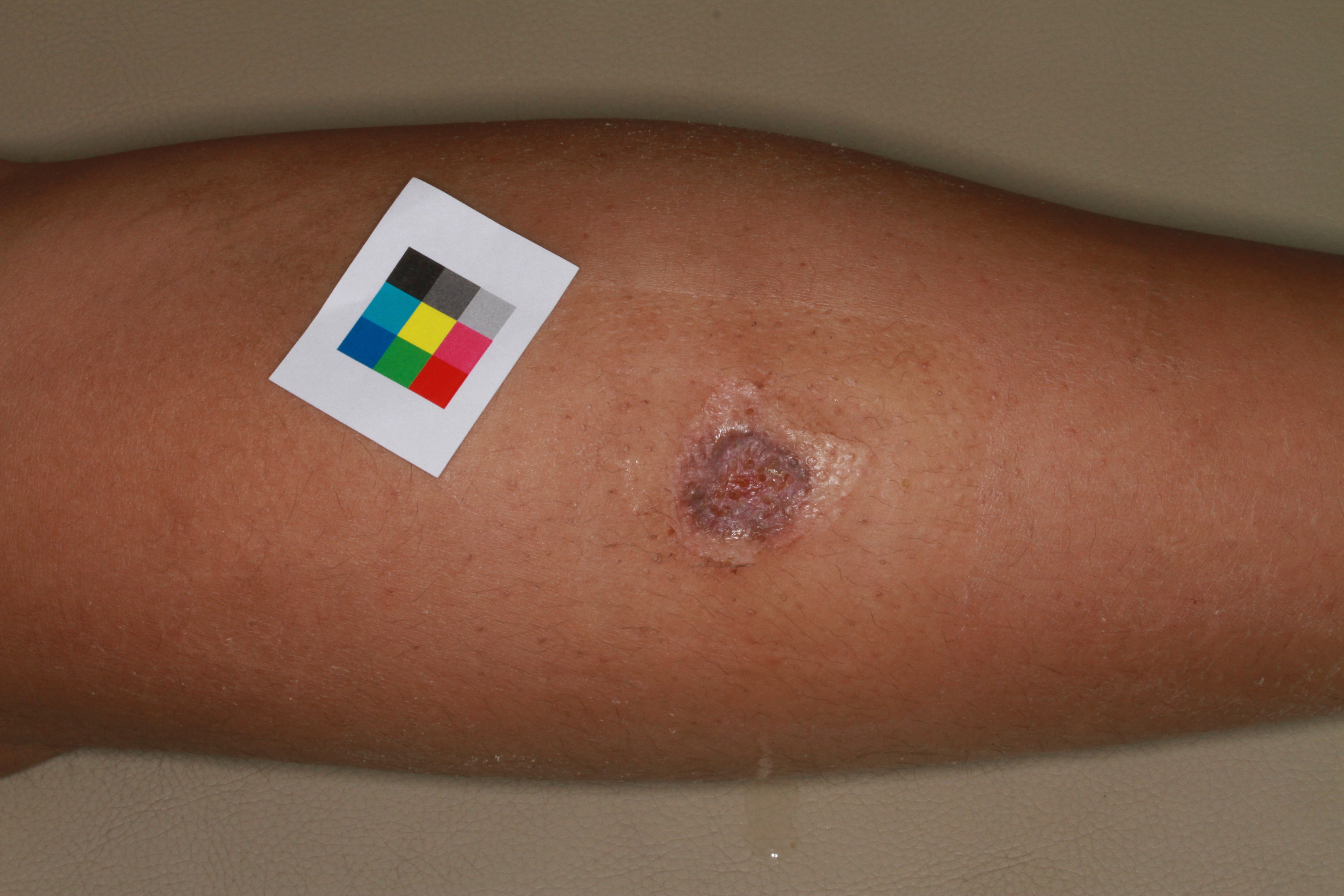

Supplement: S12 File — (ZIP) [file pone.0163092.s012.zip › 31030.JPG]

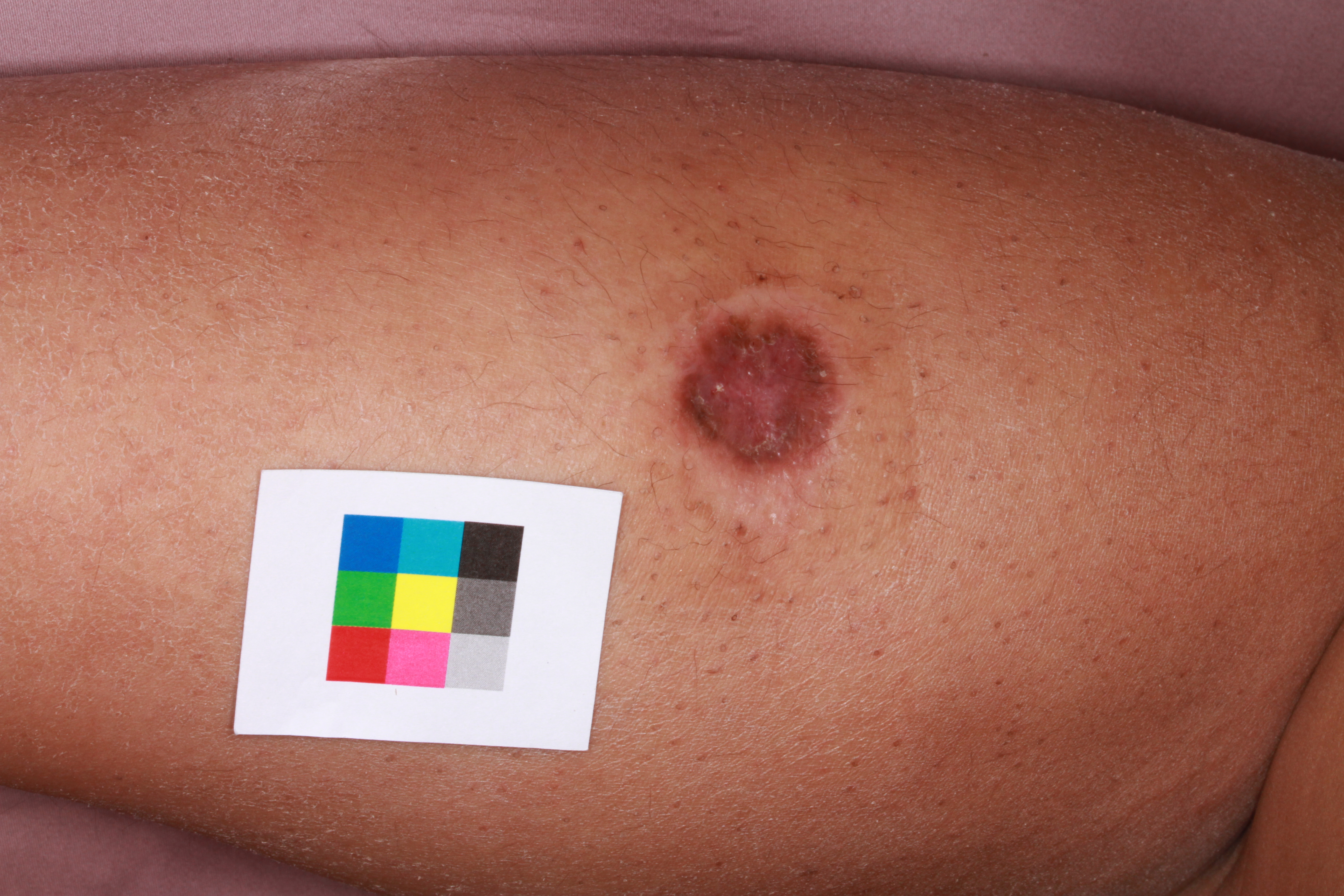

Supplement: S12 File — (ZIP) [file pone.0163092.s012.zip › 31108.JPG]

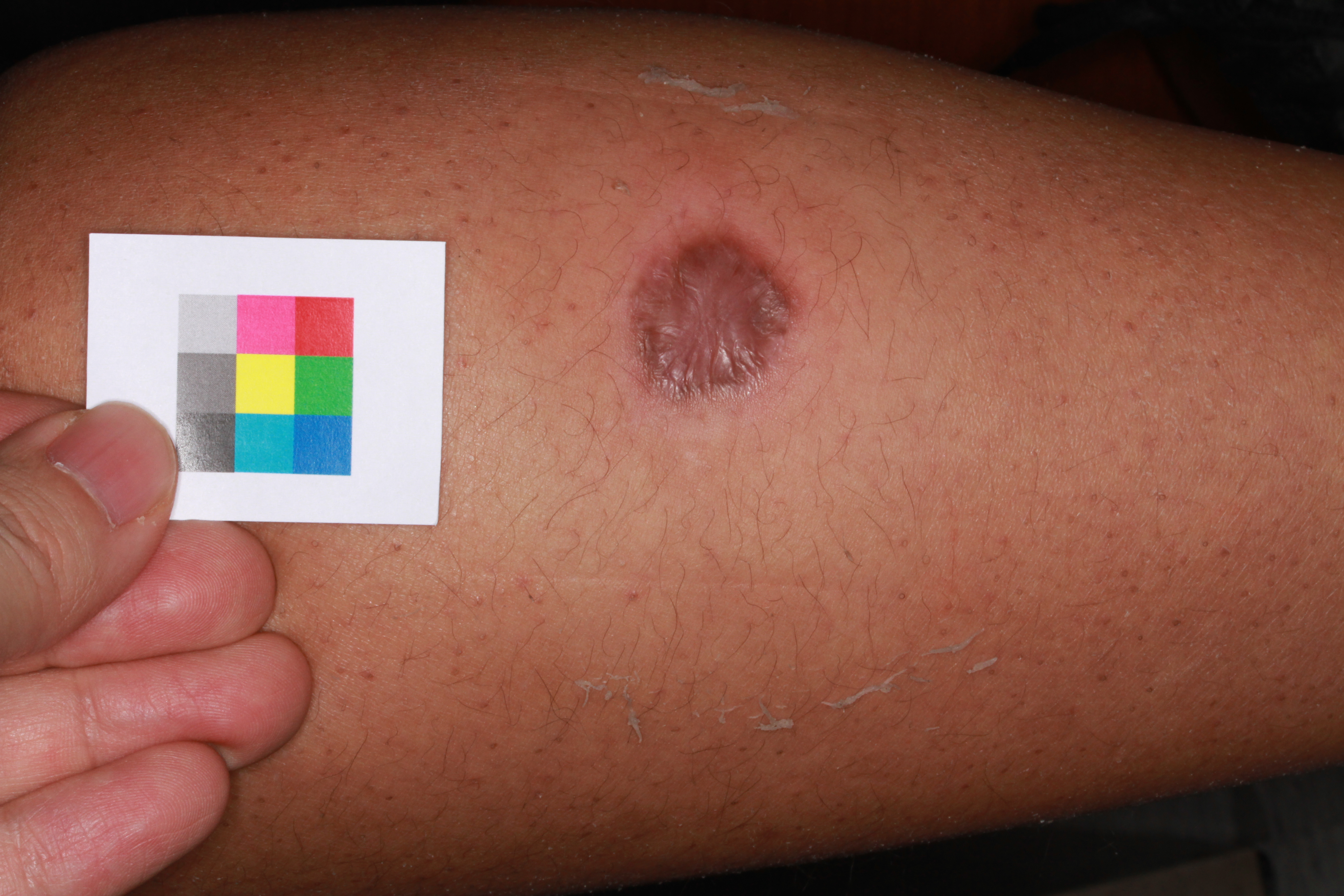

Supplement: S12 File — (ZIP) [file pone.0163092.s012.zip › 31220.JPG]

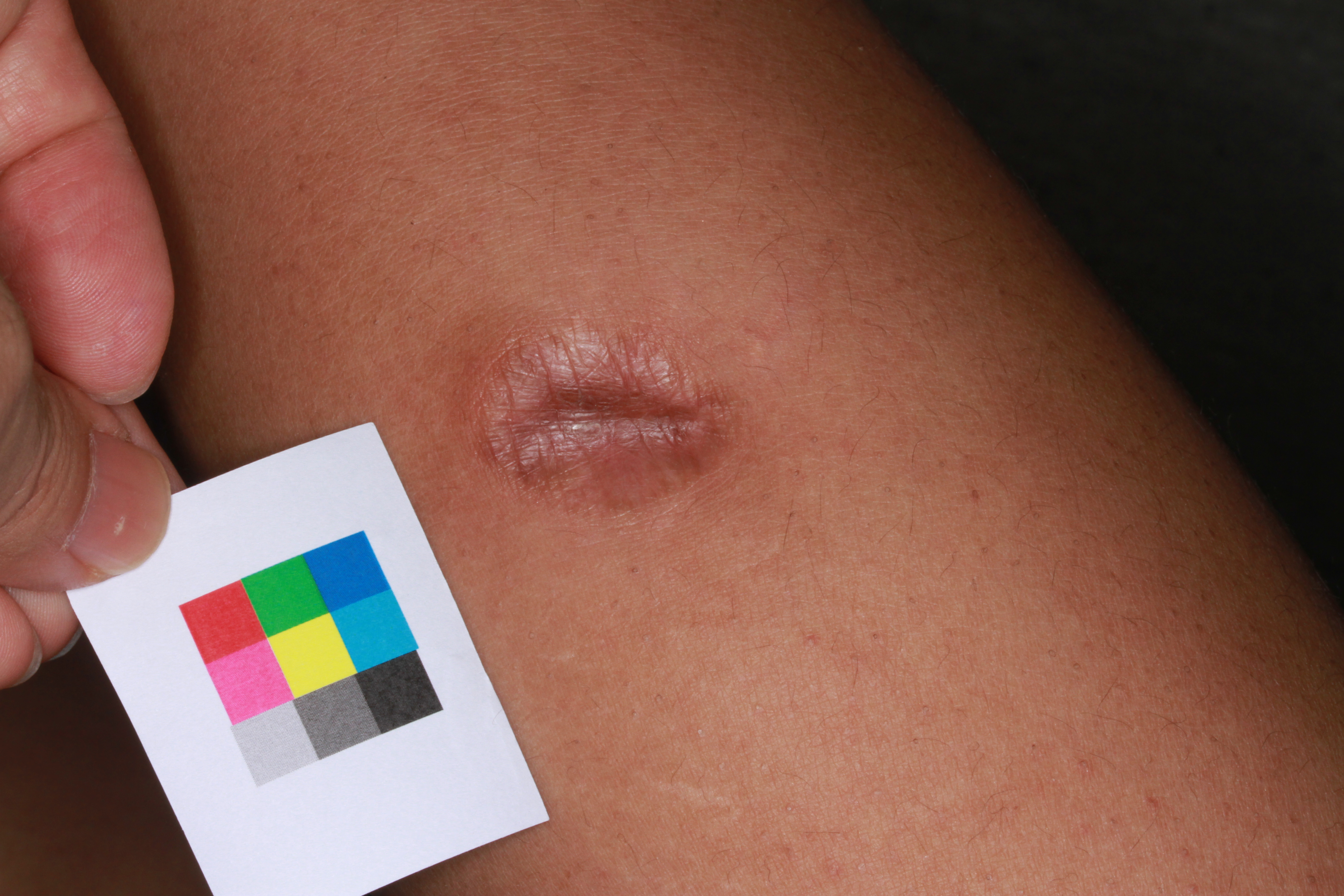

Supplement: S12 File — (ZIP) [file pone.0163092.s012.zip › 40812.JPG]

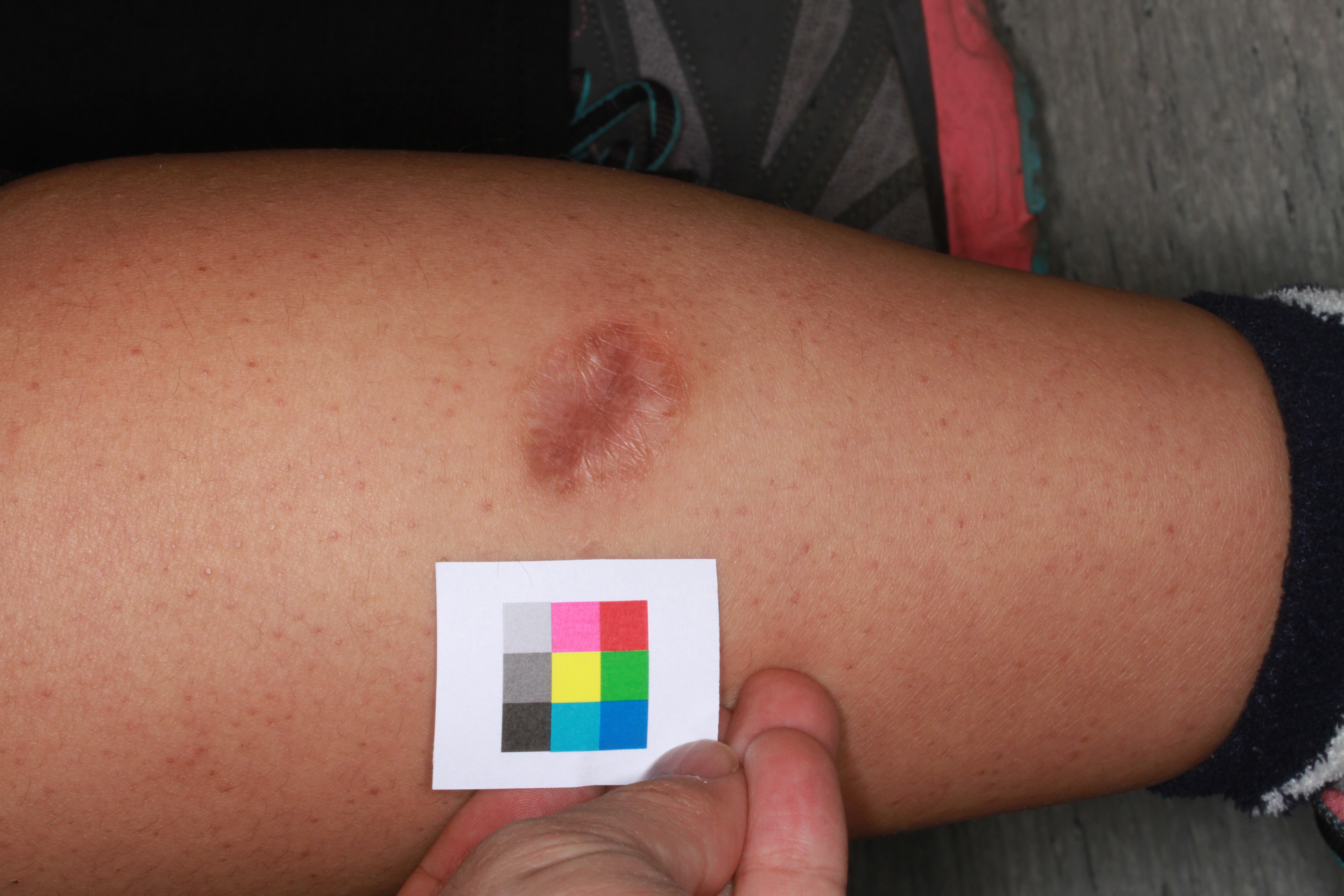

Supplement: S12 File — (ZIP) [file pone.0163092.s012.zip › 41229.JPG]

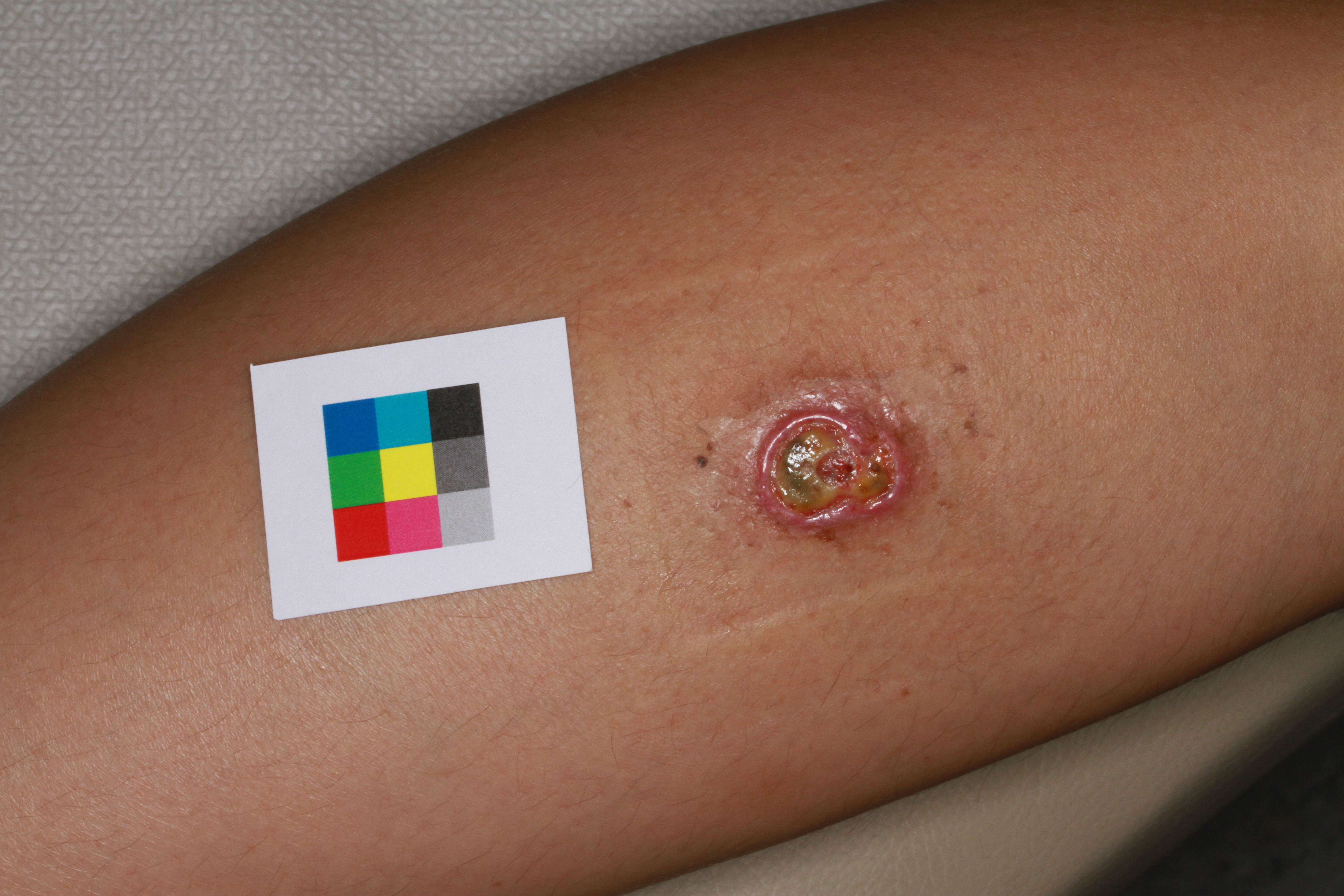

Supplement: S13 File — (ZIP) [file pone.0163092.s013.zip › 31001.JPG]

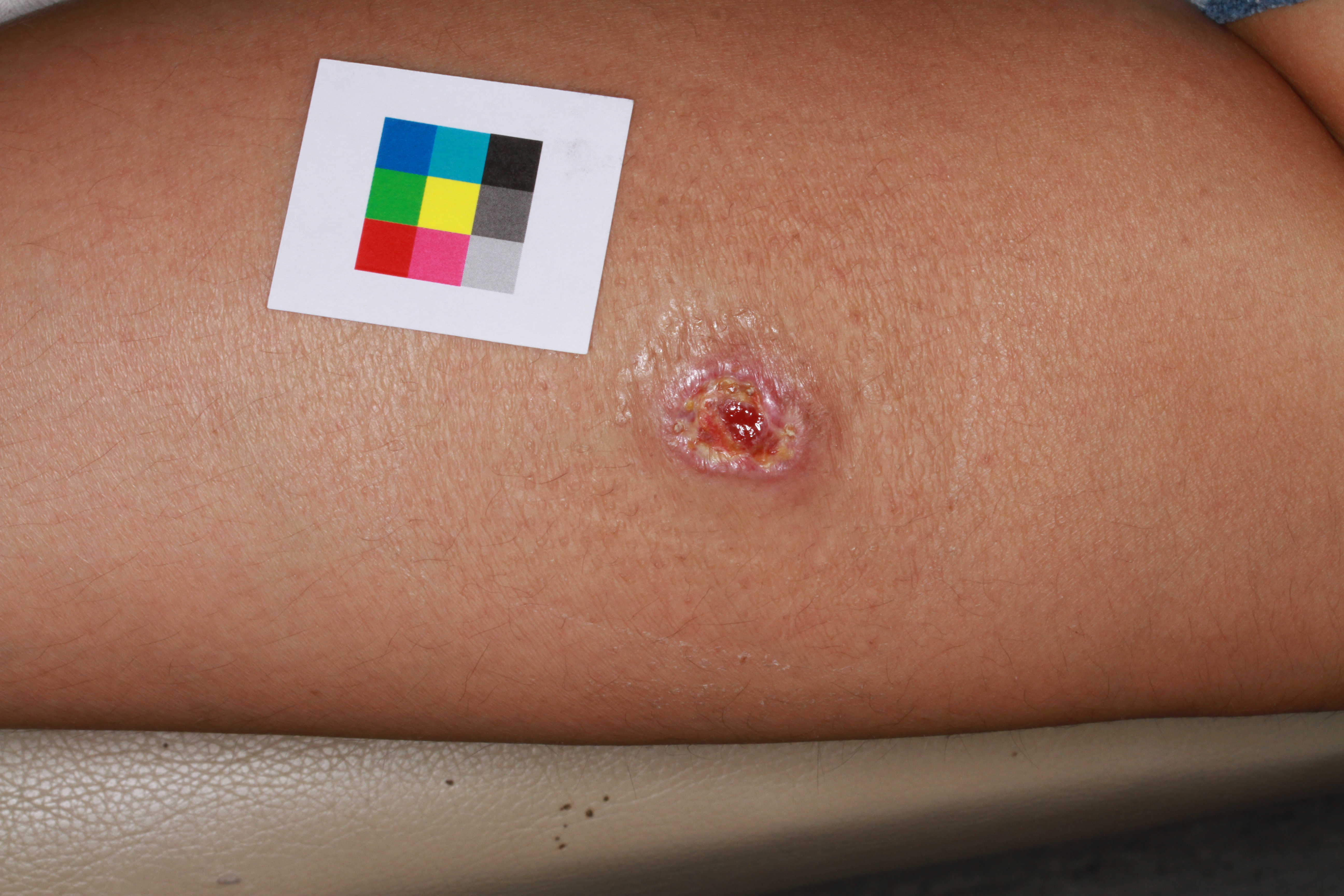

Supplement: S13 File — (ZIP) [file pone.0163092.s013.zip › 31011.JPG]
