# Supplementary figures and images for: Sequential Change of Wound Calculated by Image Analysis Using a Color Patch Method during a Secondary Intention Healing (part 2 of 3)
Source: PLoS One. 2016 Sep 20;11(9):e0163092. doi: 10.1371/journal.pone.0163092 (PMC5029888; doi:10.1371/journal.pone.0163092)

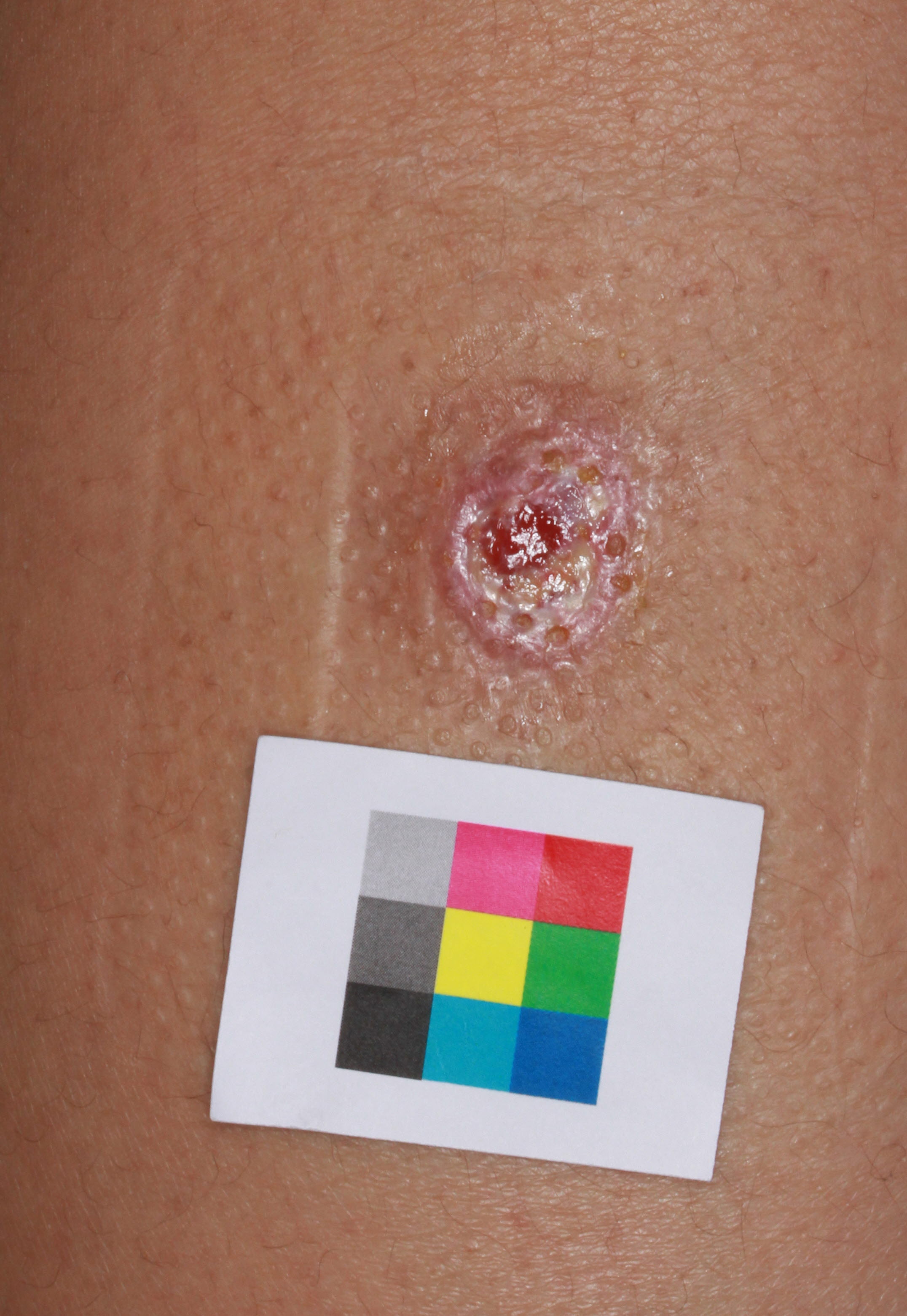

Supplement: S13 File — (ZIP) [file pone.0163092.s013.zip › 31015.jpg]

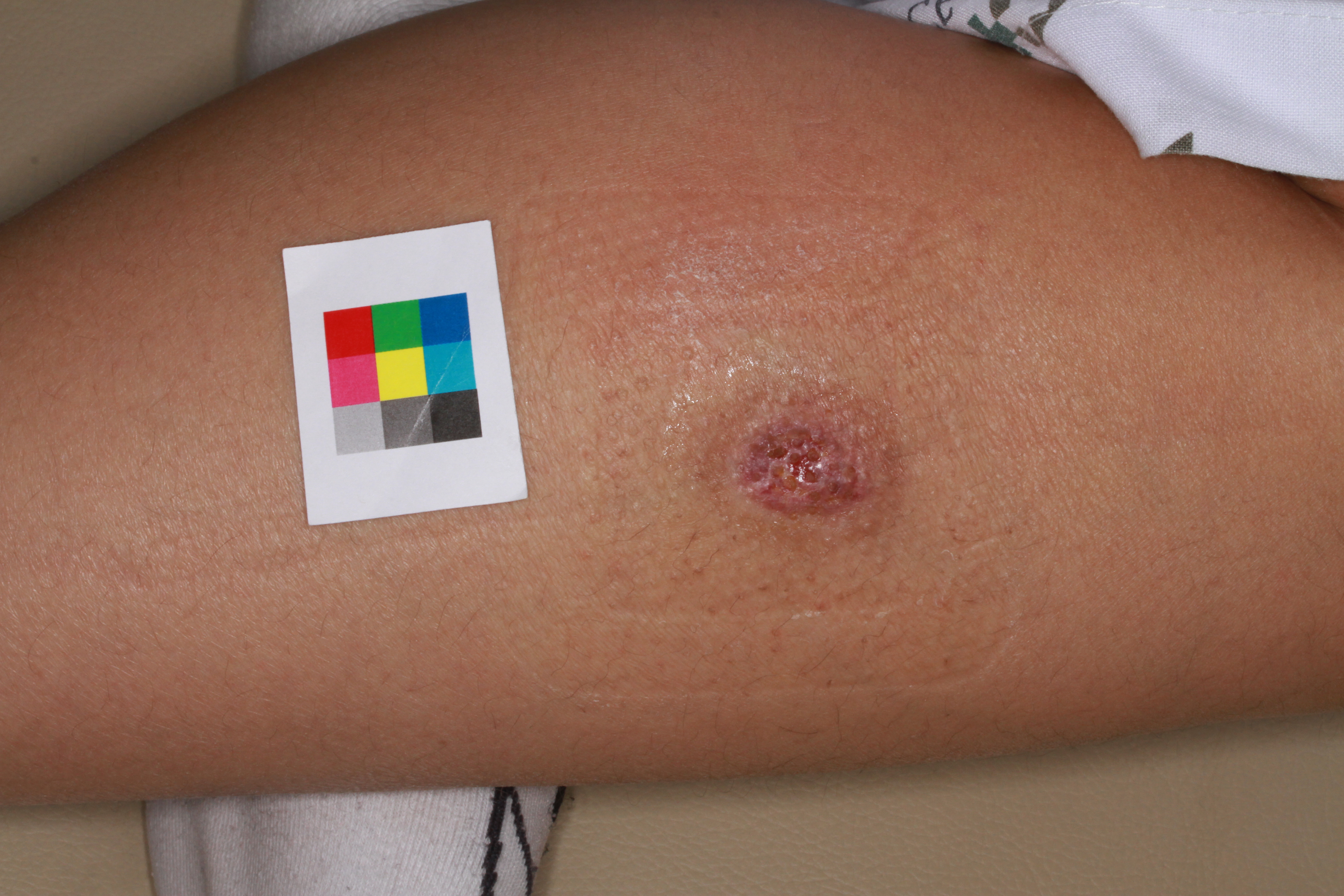

Supplement: S13 File — (ZIP) [file pone.0163092.s013.zip › 31022.JPG]

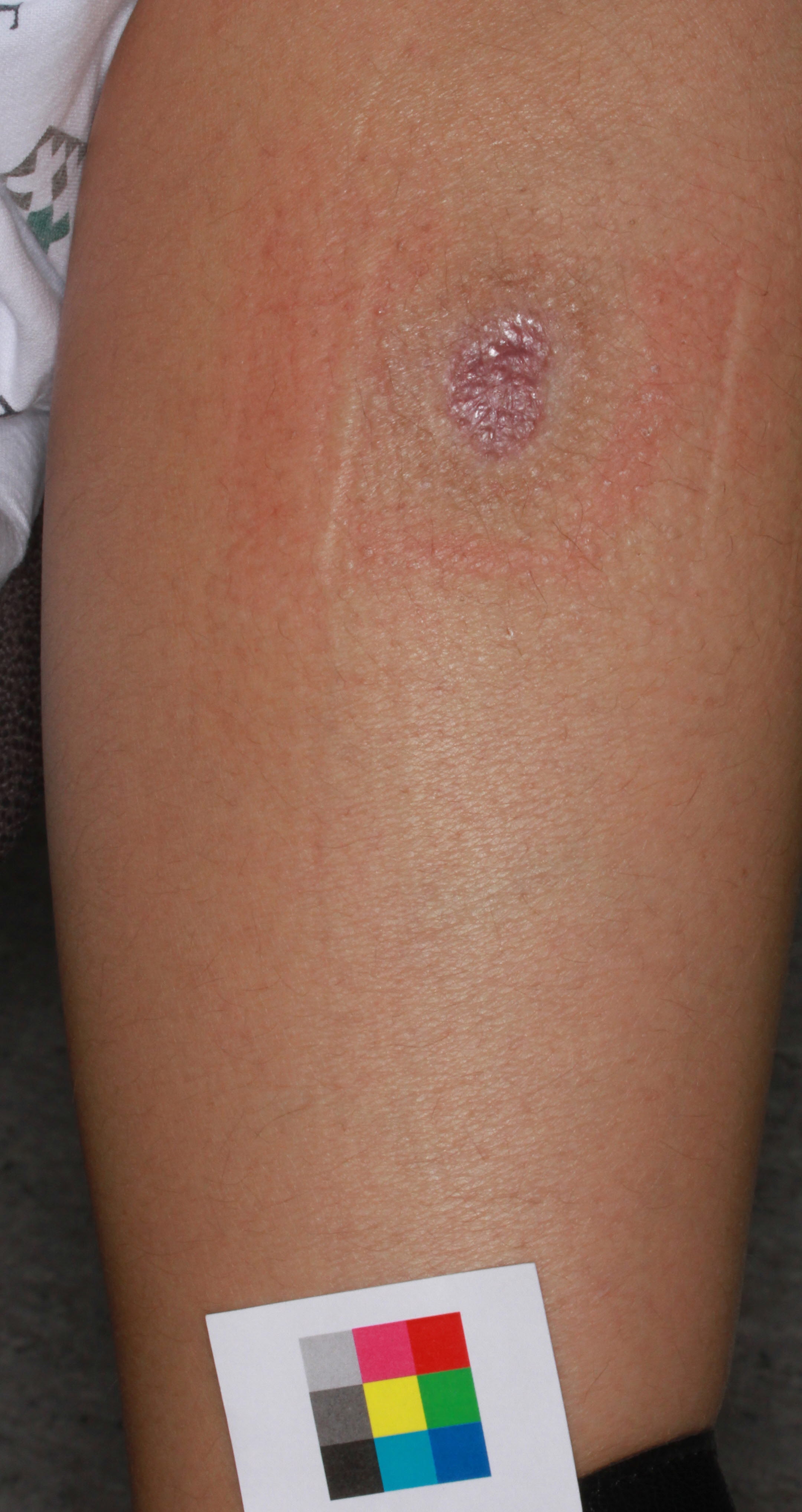

Supplement: S13 File — (ZIP) [file pone.0163092.s013.zip › 31108.jpg]

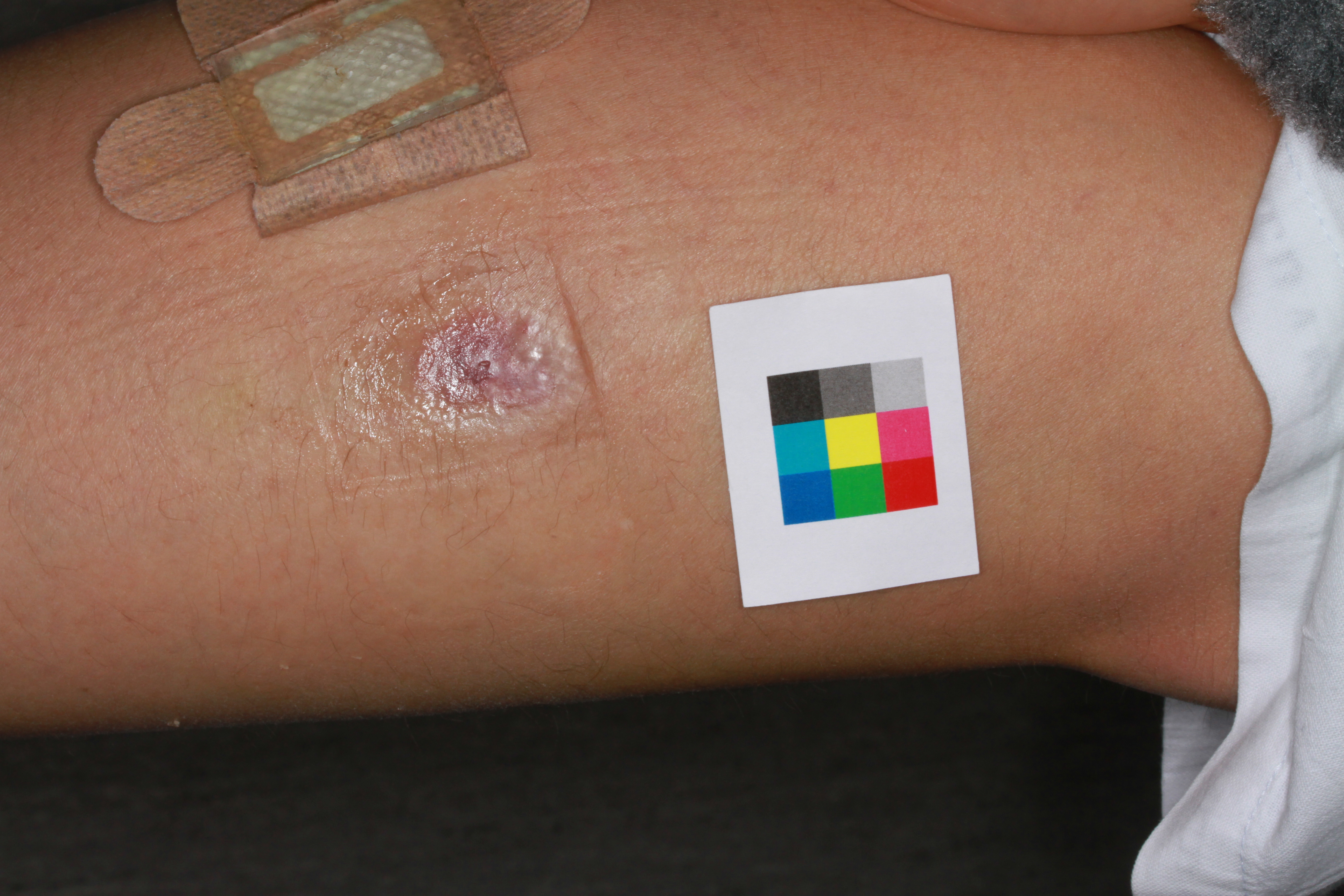

Supplement: S13 File — (ZIP) [file pone.0163092.s013.zip › 31122.JPG]

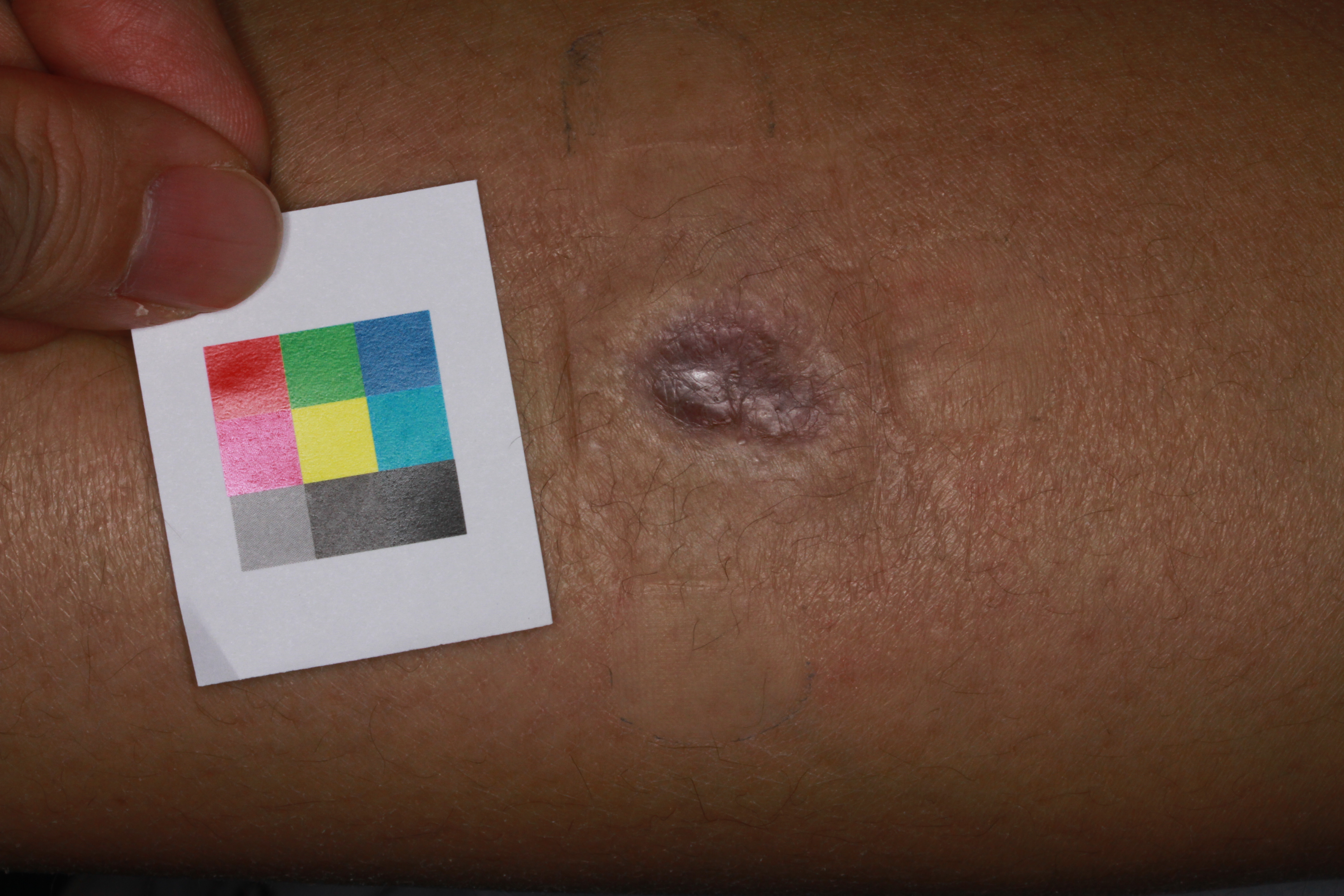

Supplement: S13 File — (ZIP) [file pone.0163092.s013.zip › 31220.JPG]

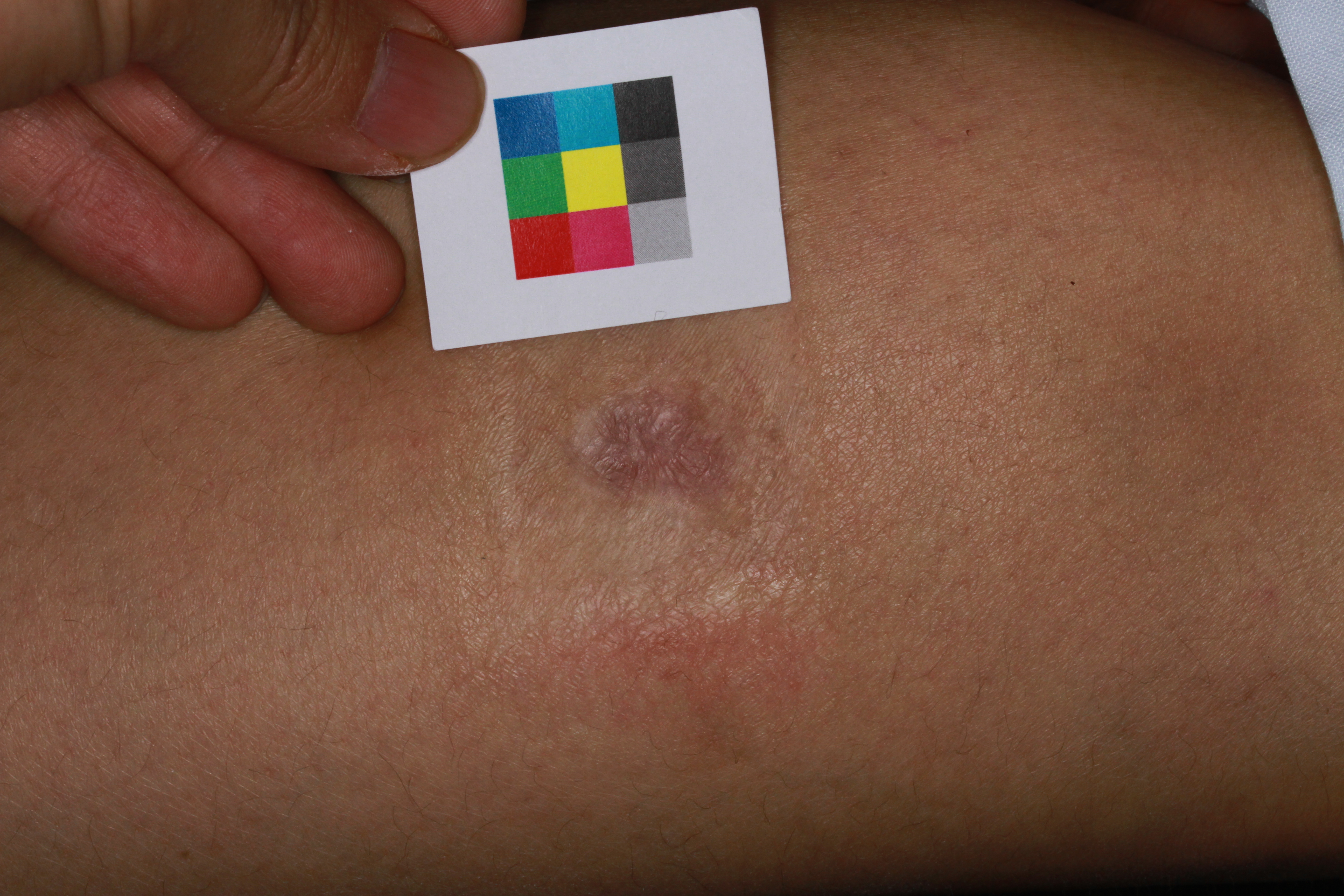

Supplement: S13 File — (ZIP) [file pone.0163092.s013.zip › 40311.JPG]

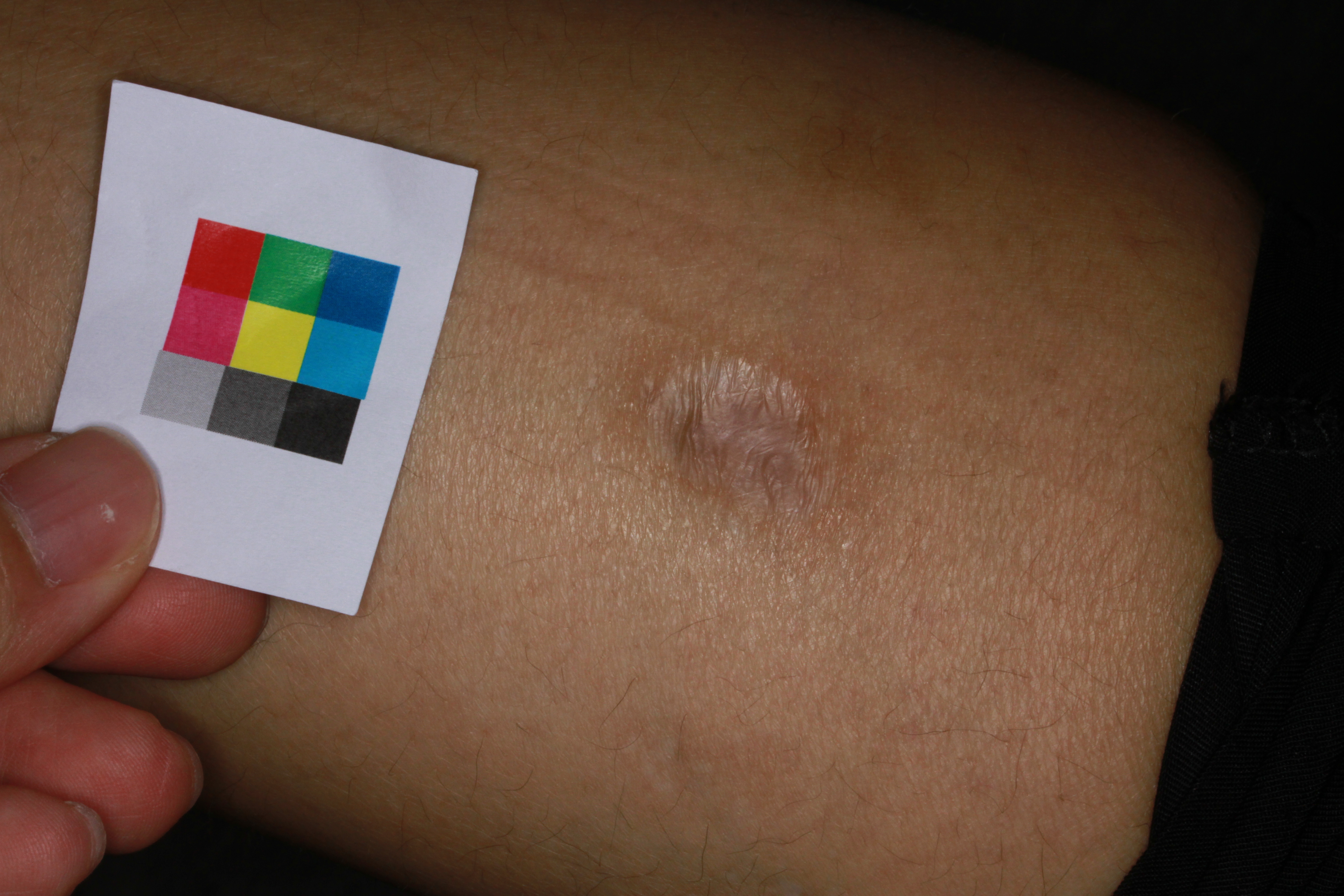

Supplement: S13 File — (ZIP) [file pone.0163092.s013.zip › 40901.JPG]

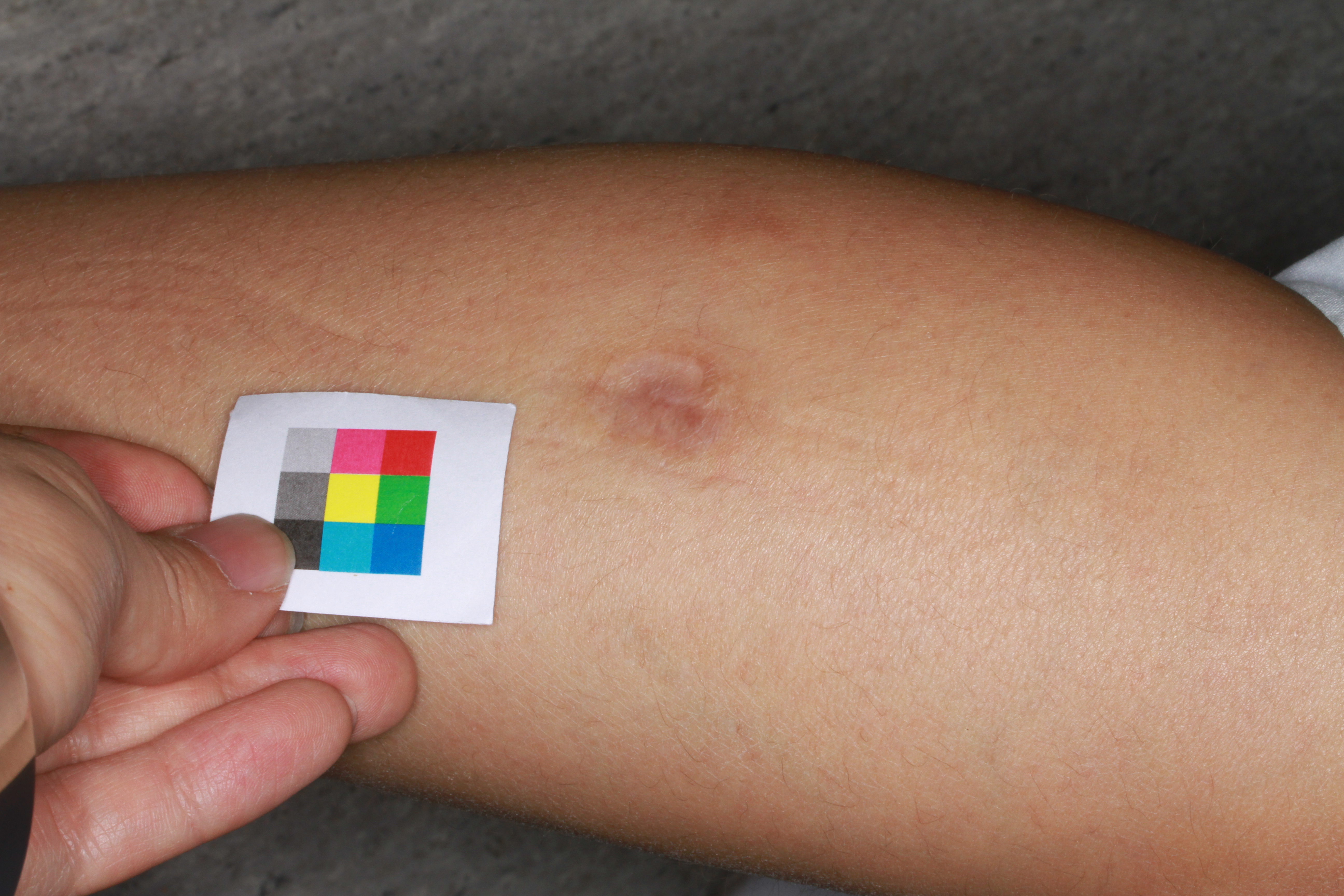

Supplement: S13 File — (ZIP) [file pone.0163092.s013.zip › 41208.JPG]

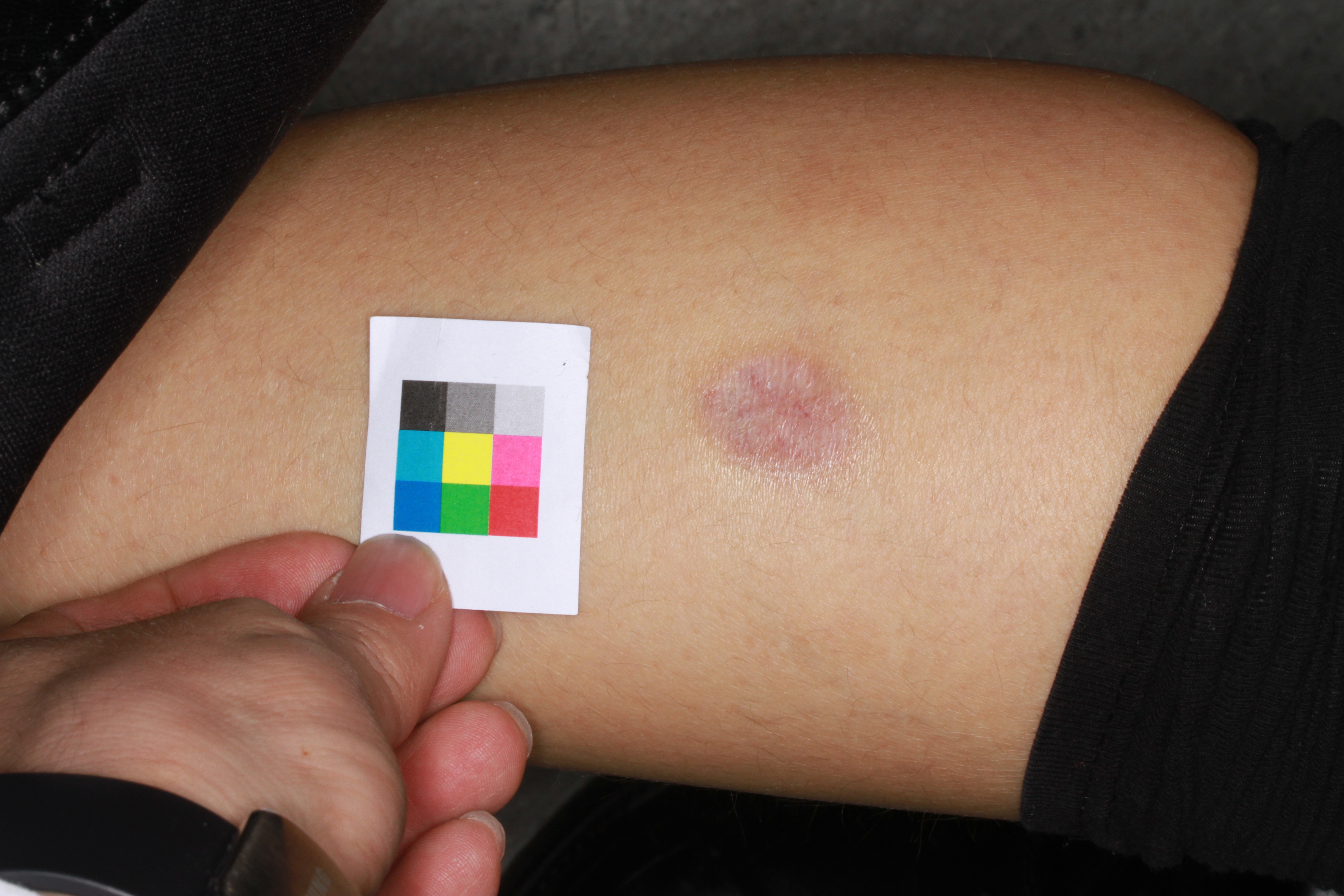

Supplement: S13 File — (ZIP) [file pone.0163092.s013.zip › 50112.JPG]

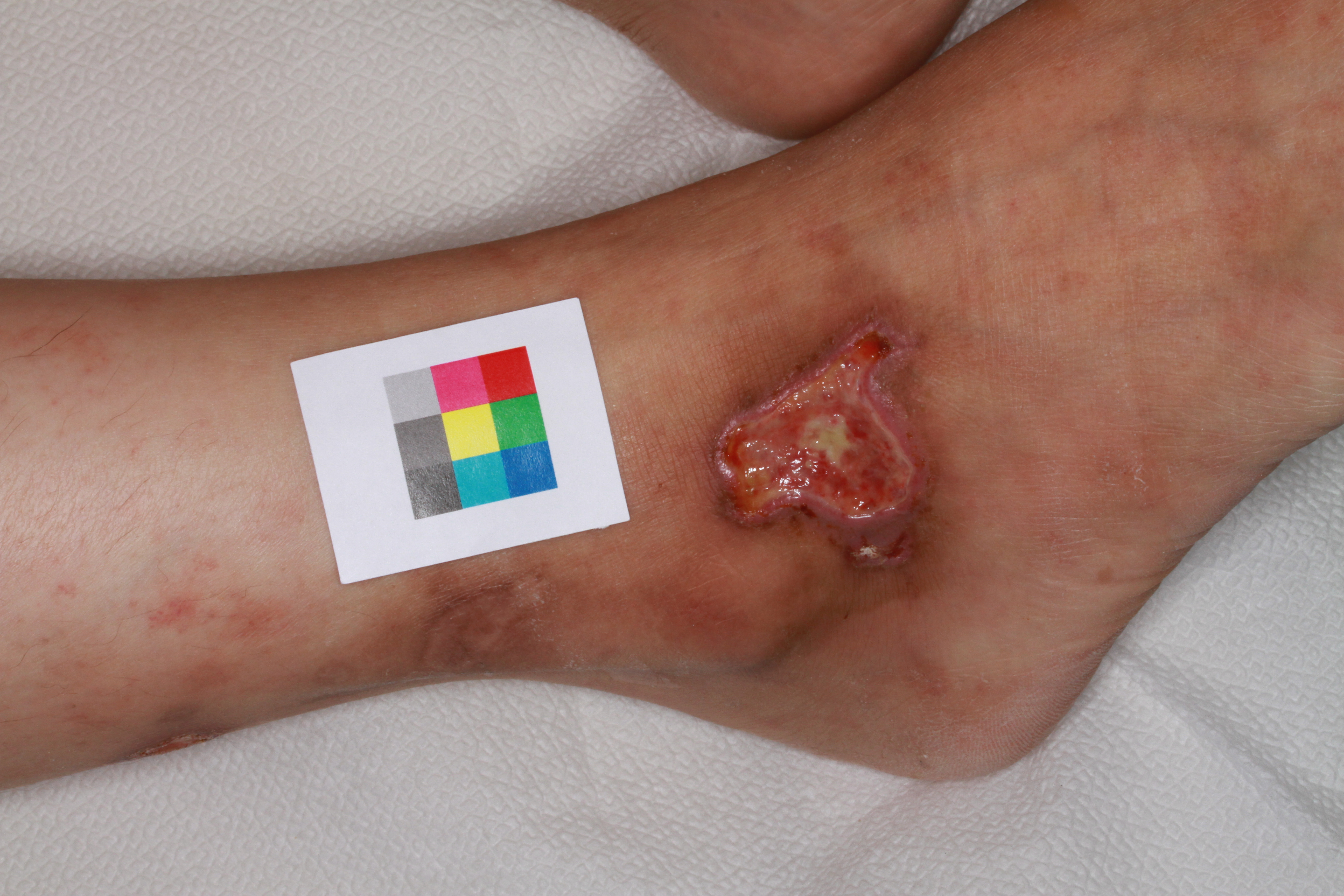

Supplement: S14 File — (ZIP) [file pone.0163092.s014.zip › 30813.JPG]

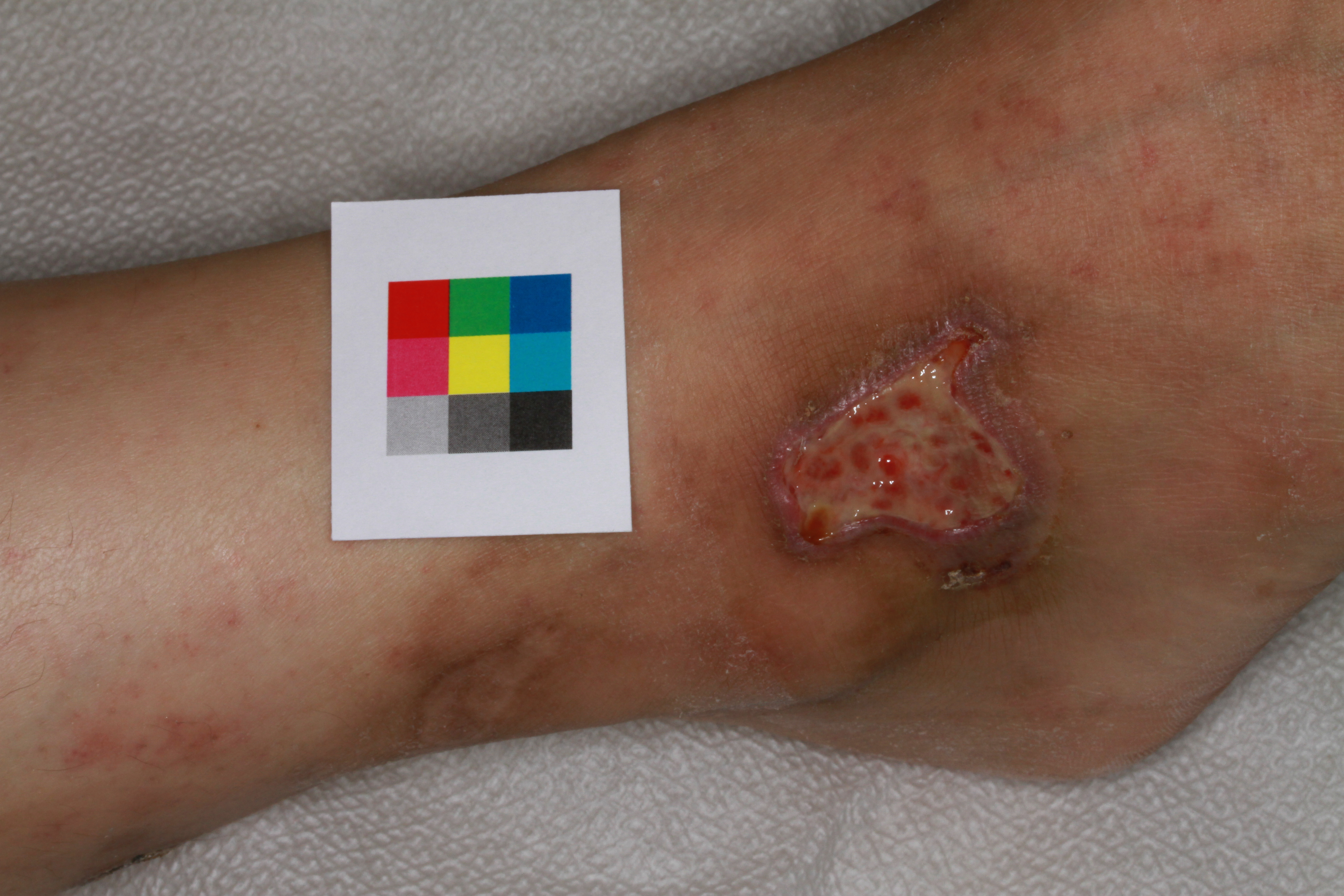

Supplement: S14 File — (ZIP) [file pone.0163092.s014.zip › 30821.JPG]

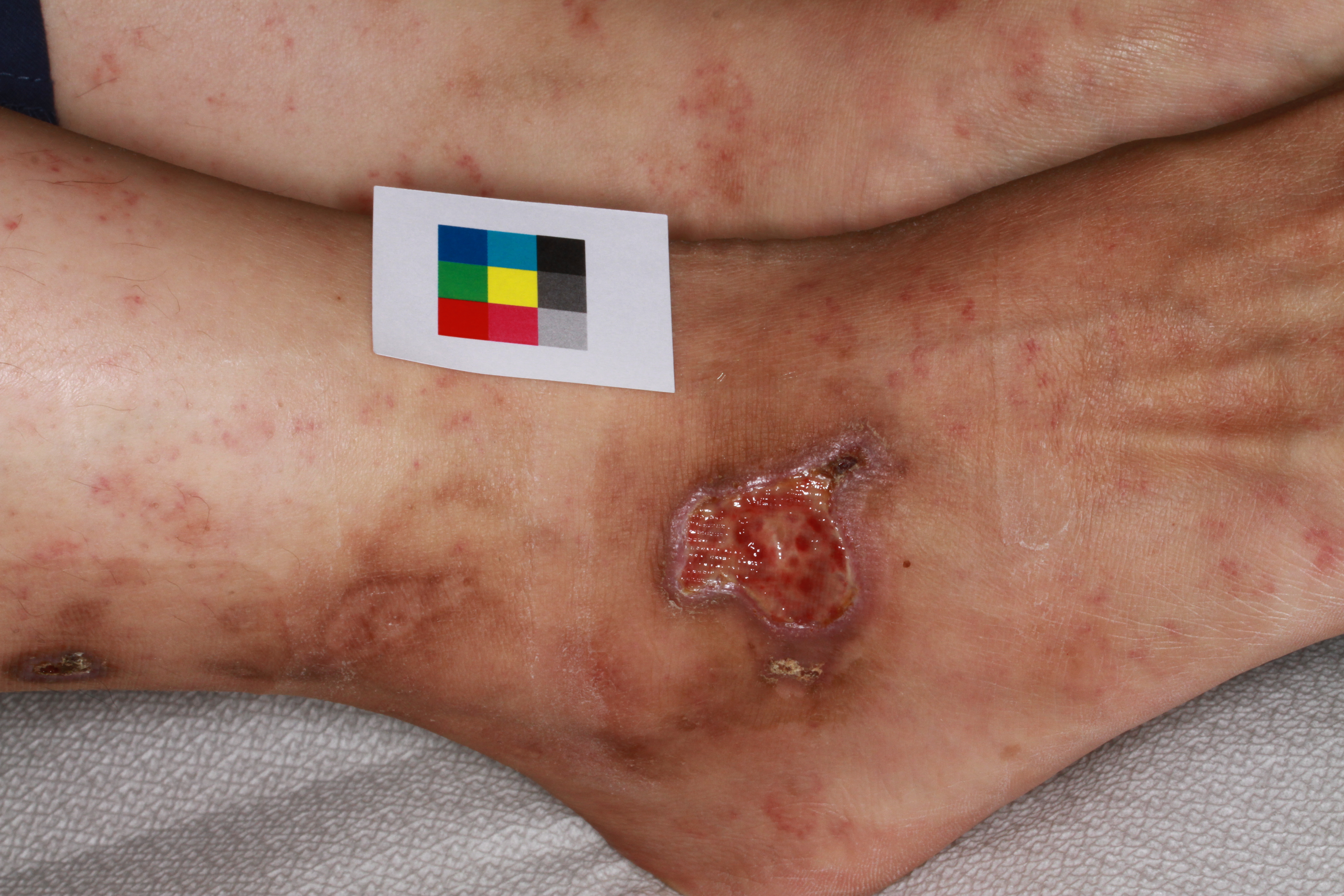

Supplement: S14 File — (ZIP) [file pone.0163092.s014.zip › 30828.JPG]

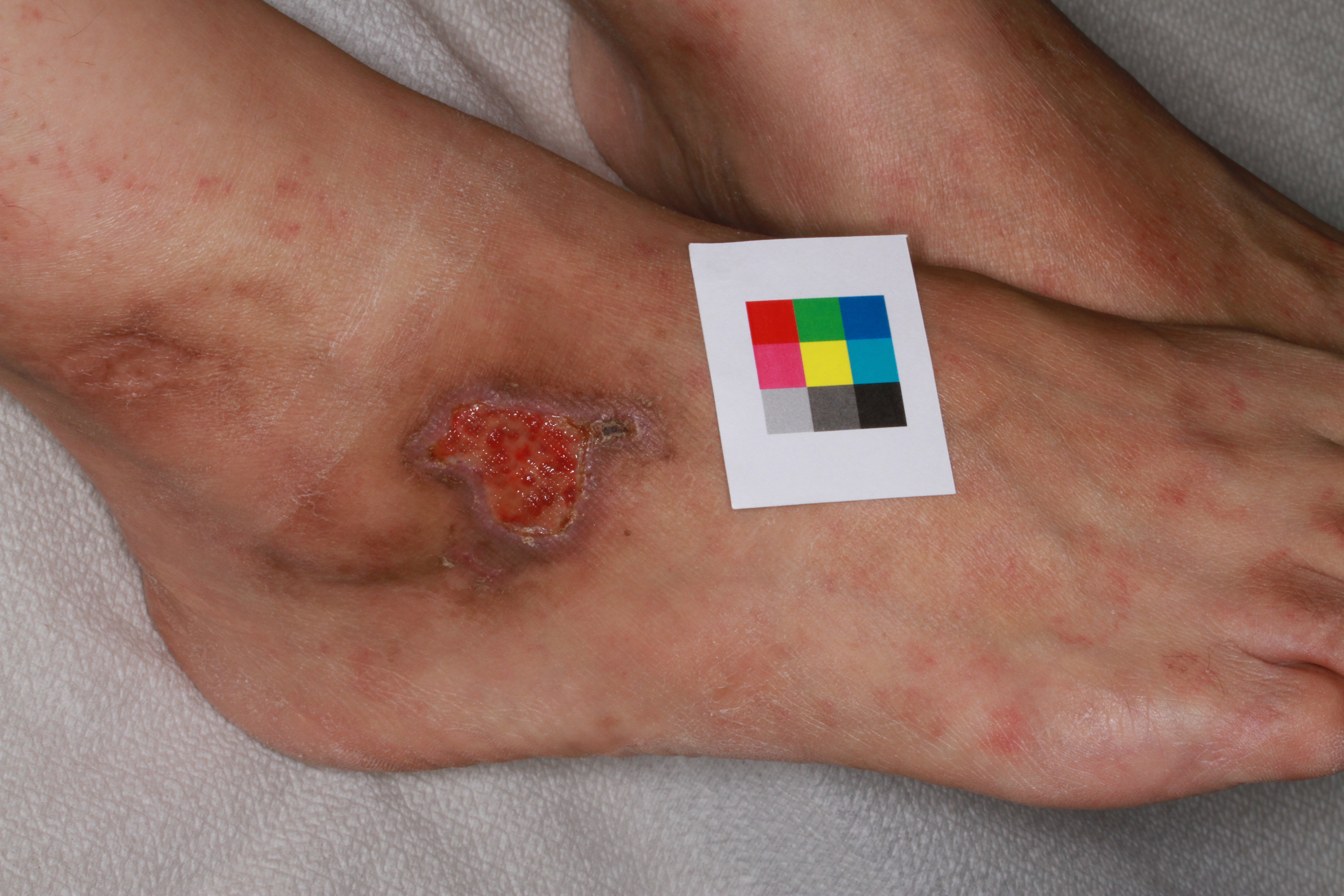

Supplement: S14 File — (ZIP) [file pone.0163092.s014.zip › 30904.JPG]

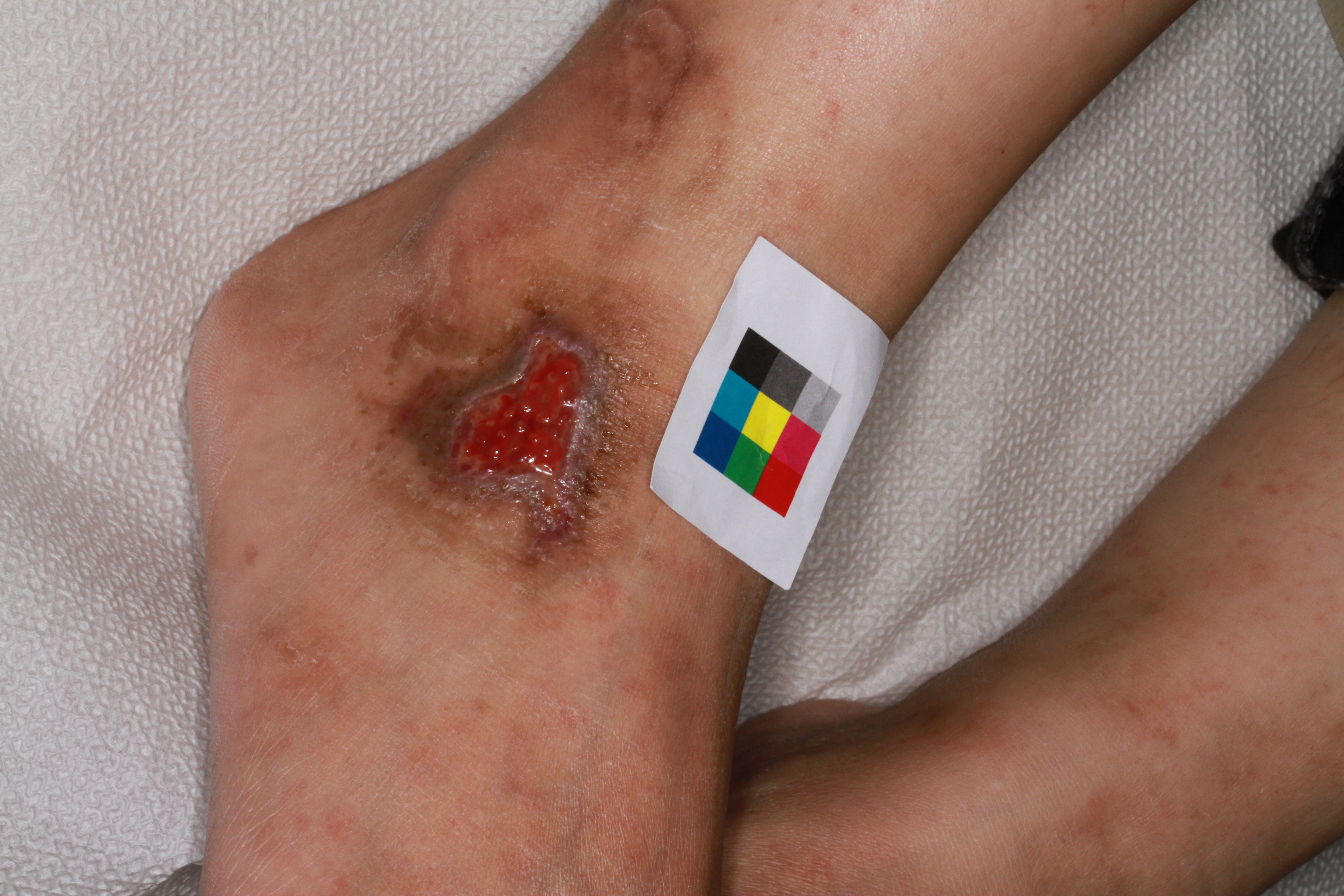

Supplement: S14 File — (ZIP) [file pone.0163092.s014.zip › 30911.JPG]

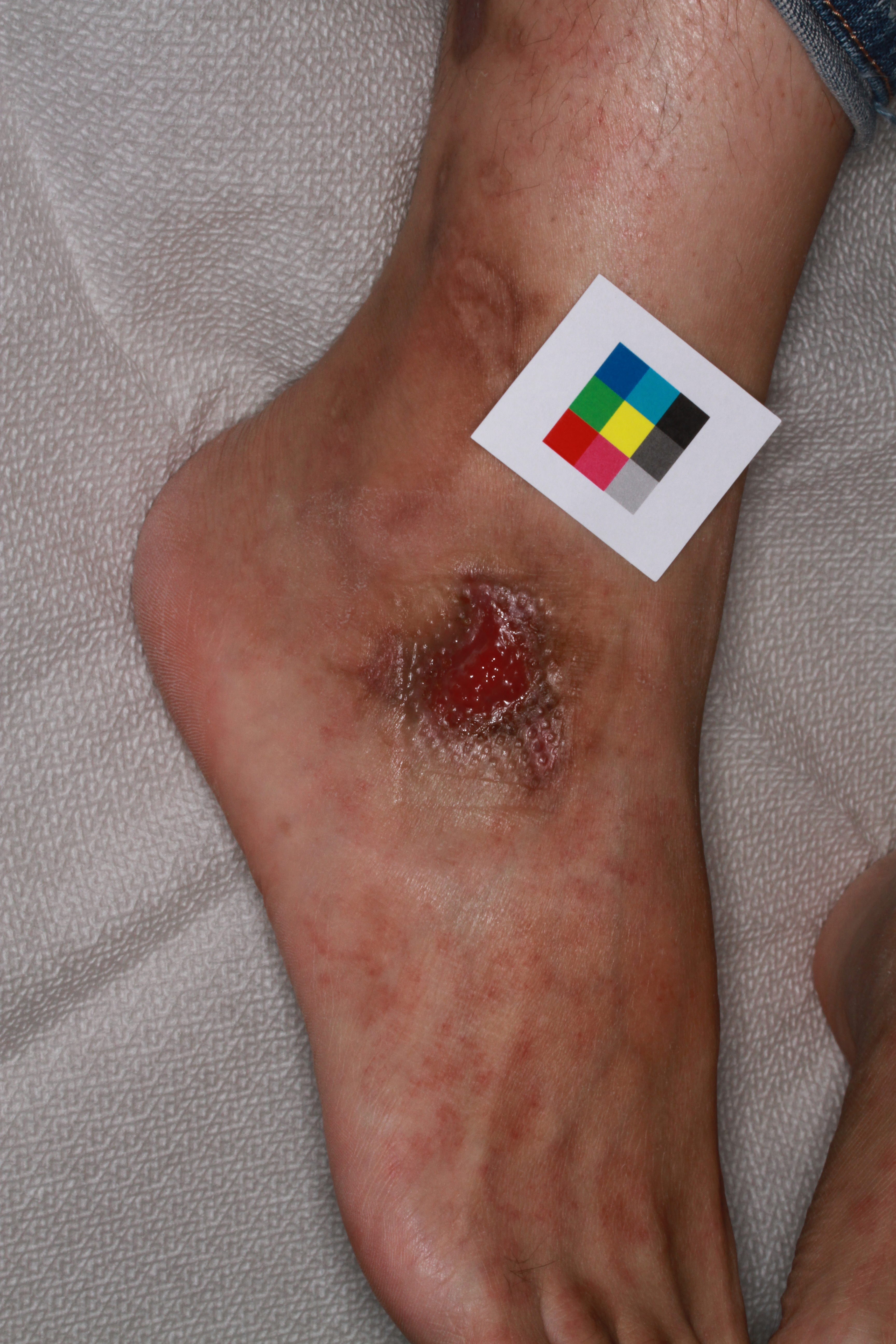

Supplement: S14 File — (ZIP) [file pone.0163092.s014.zip › 31008.JPG]

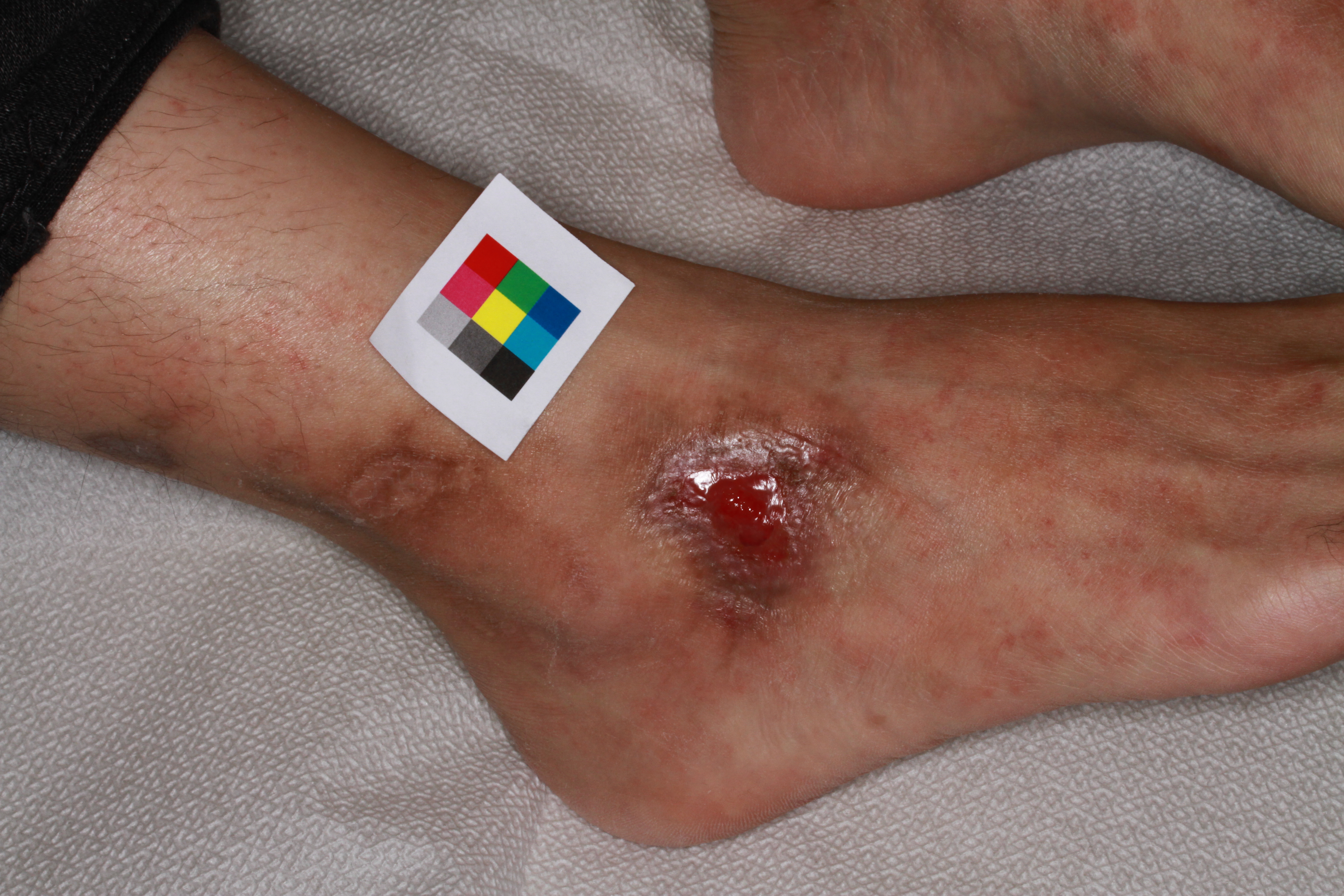

Supplement: S14 File — (ZIP) [file pone.0163092.s014.zip › 31015.JPG]

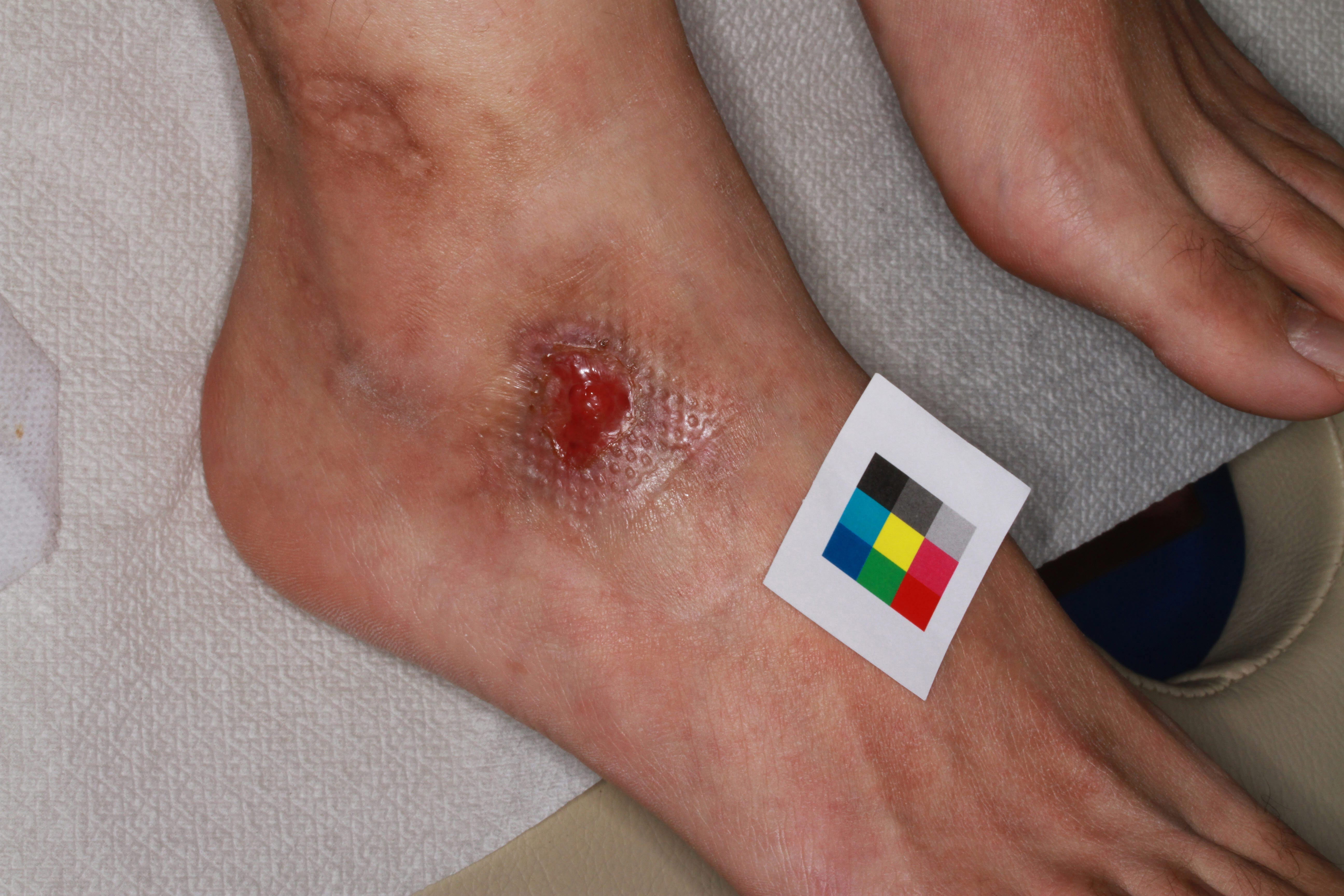

Supplement: S14 File — (ZIP) [file pone.0163092.s014.zip › 31106.JPG]

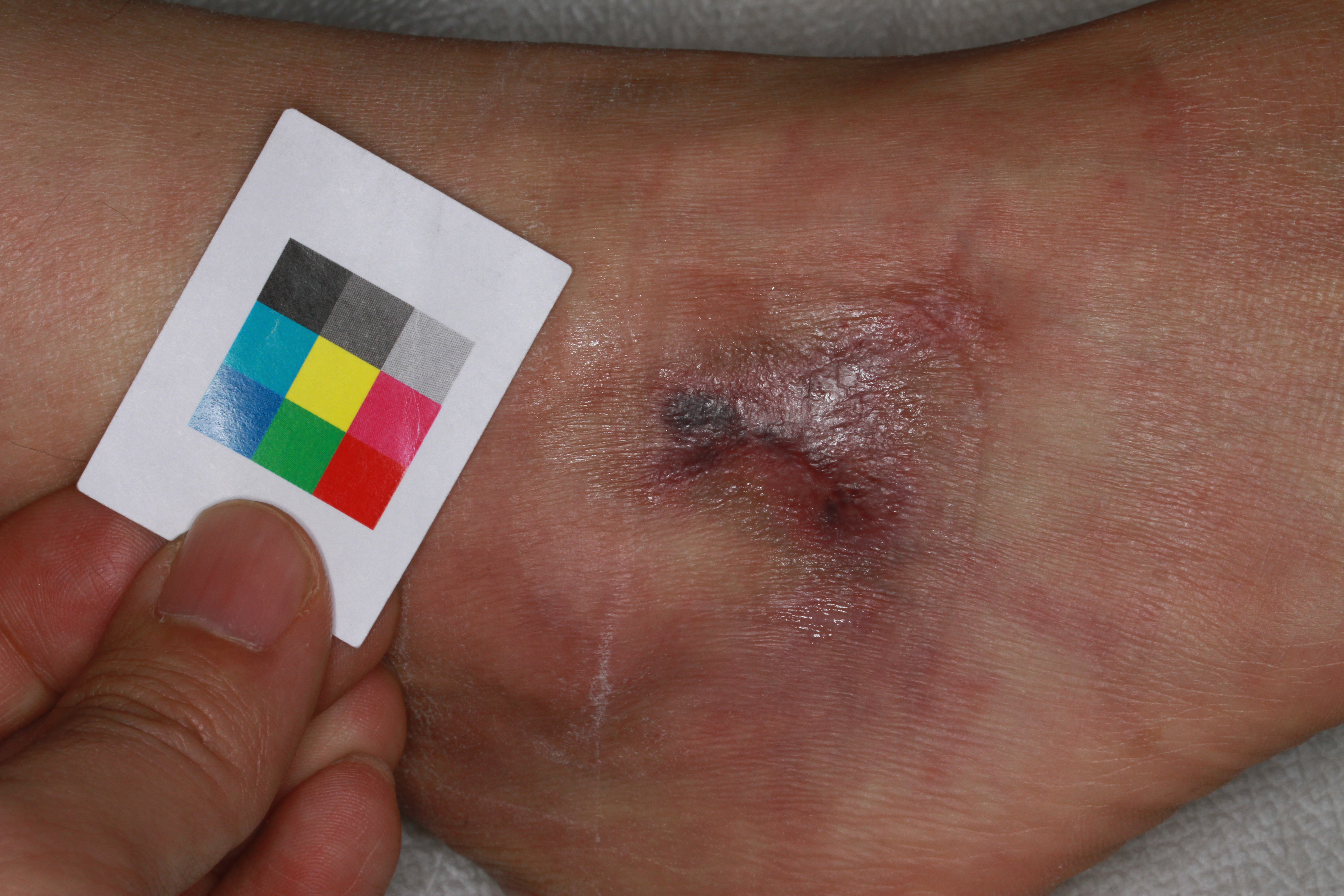

Supplement: S14 File — (ZIP) [file pone.0163092.s014.zip › 40205.JPG]

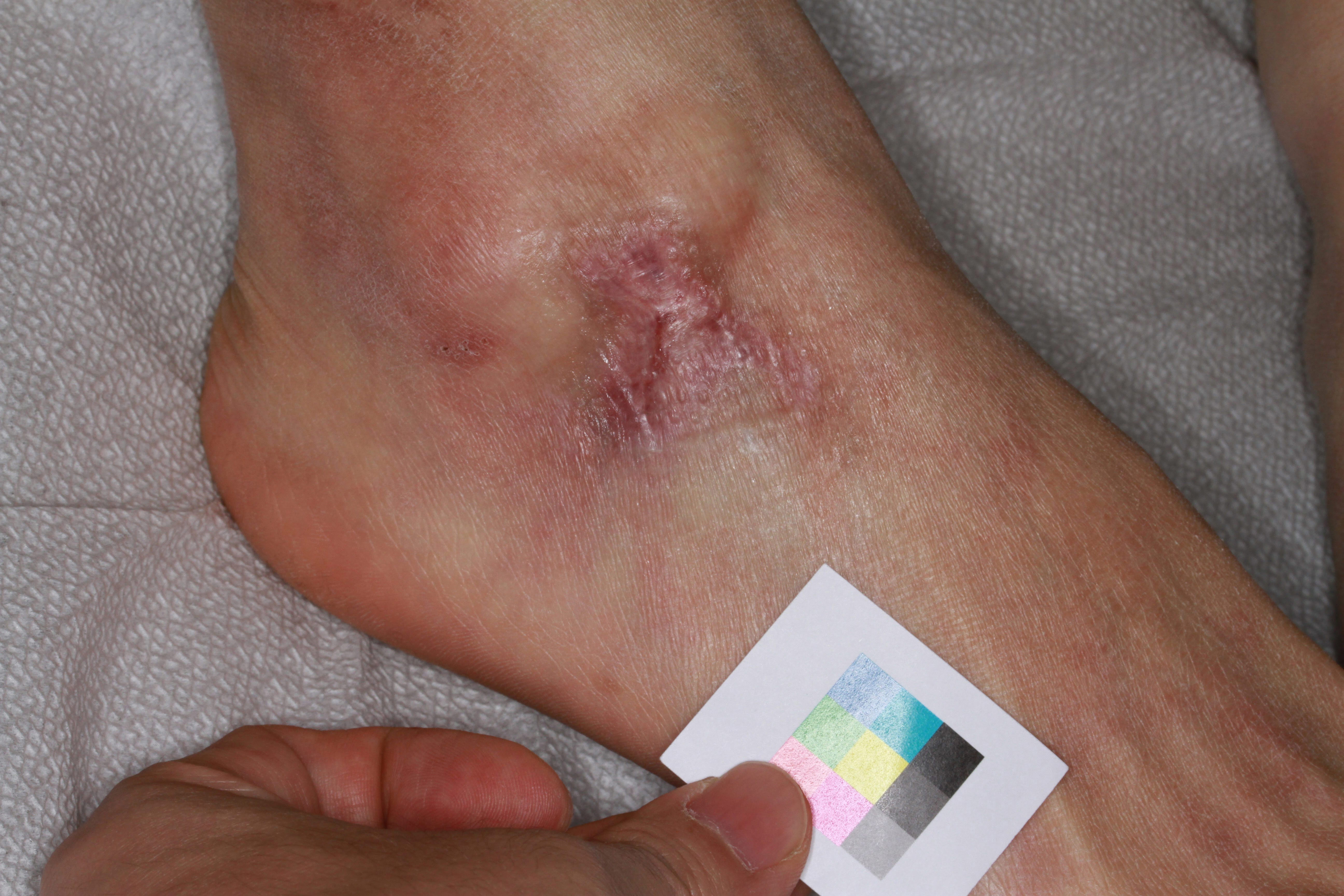

Supplement: S14 File — (ZIP) [file pone.0163092.s014.zip › 40219.JPG]

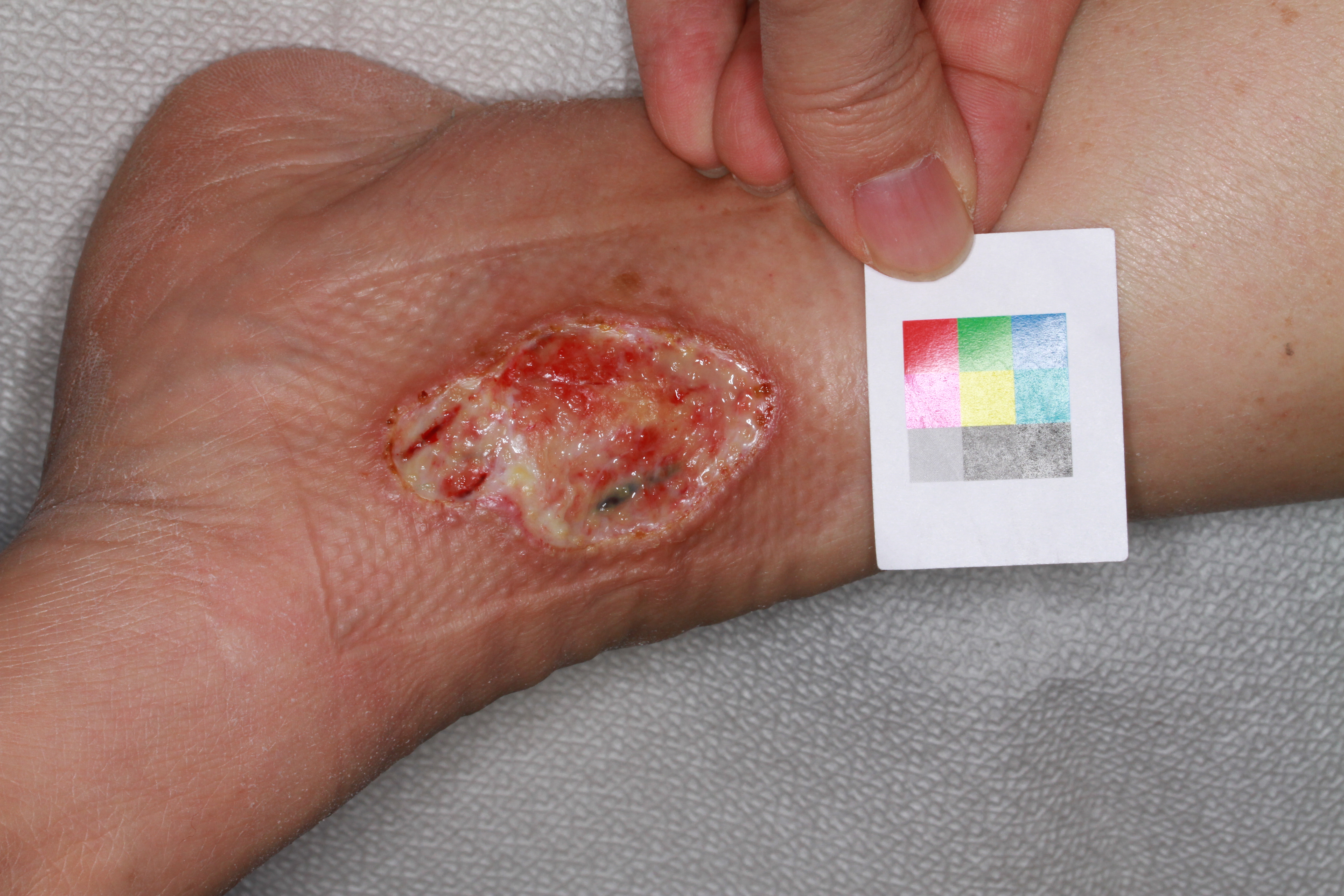

Supplement: S15 File — (ZIP) [file pone.0163092.s015.zip › 40124.JPG]

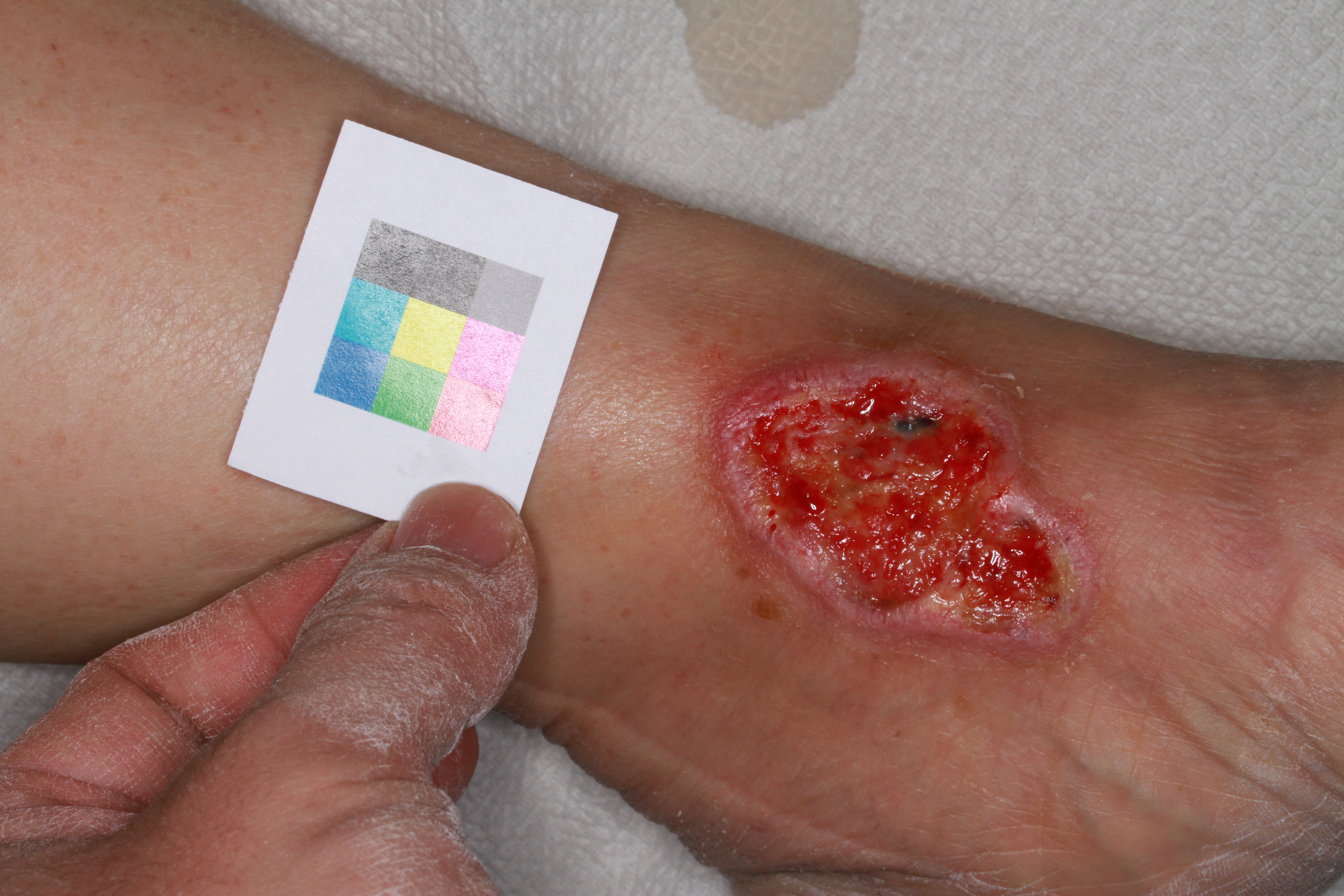

Supplement: S15 File — (ZIP) [file pone.0163092.s015.zip › 40204.JPG]

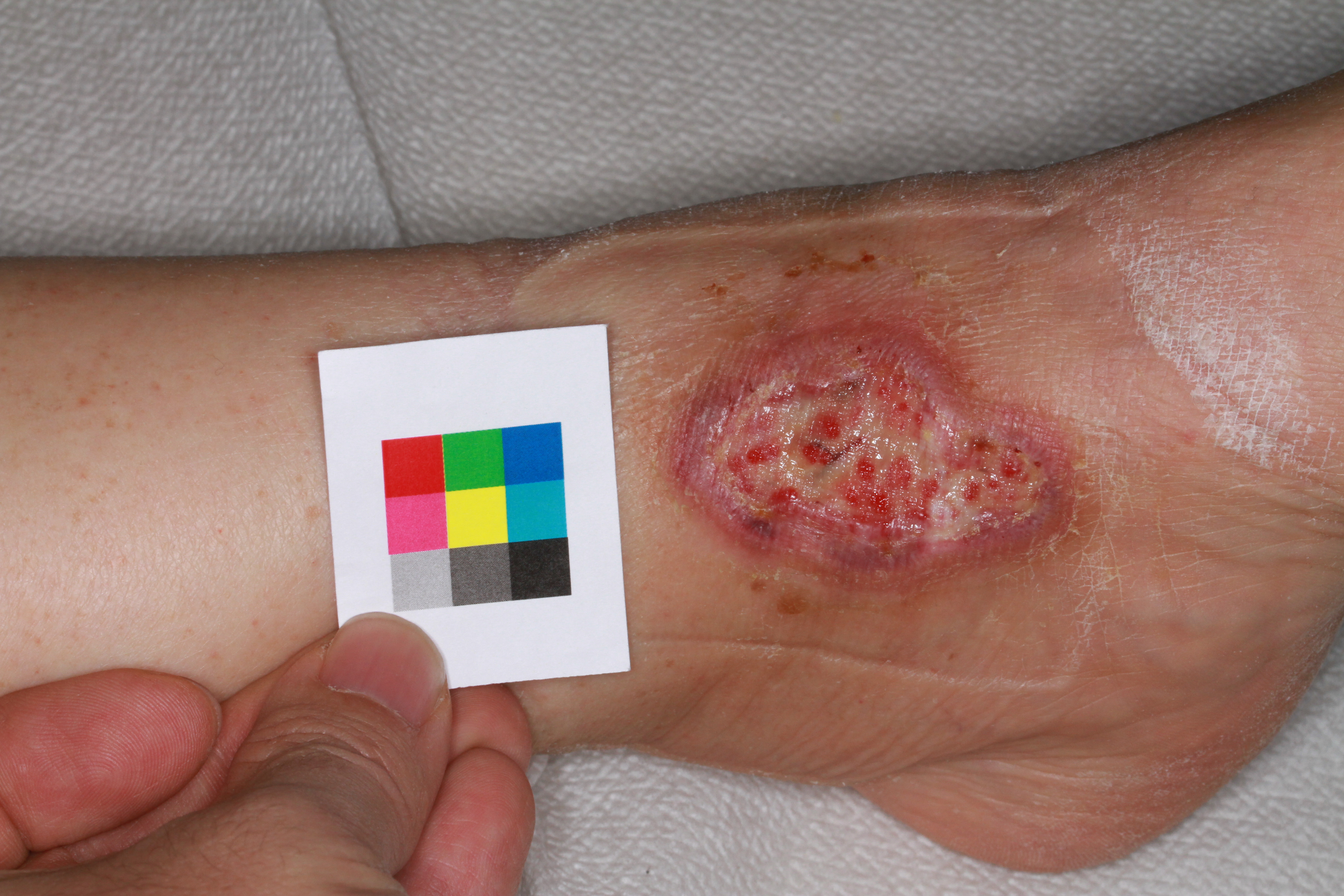

Supplement: S15 File — (ZIP) [file pone.0163092.s015.zip › 40213.JPG]

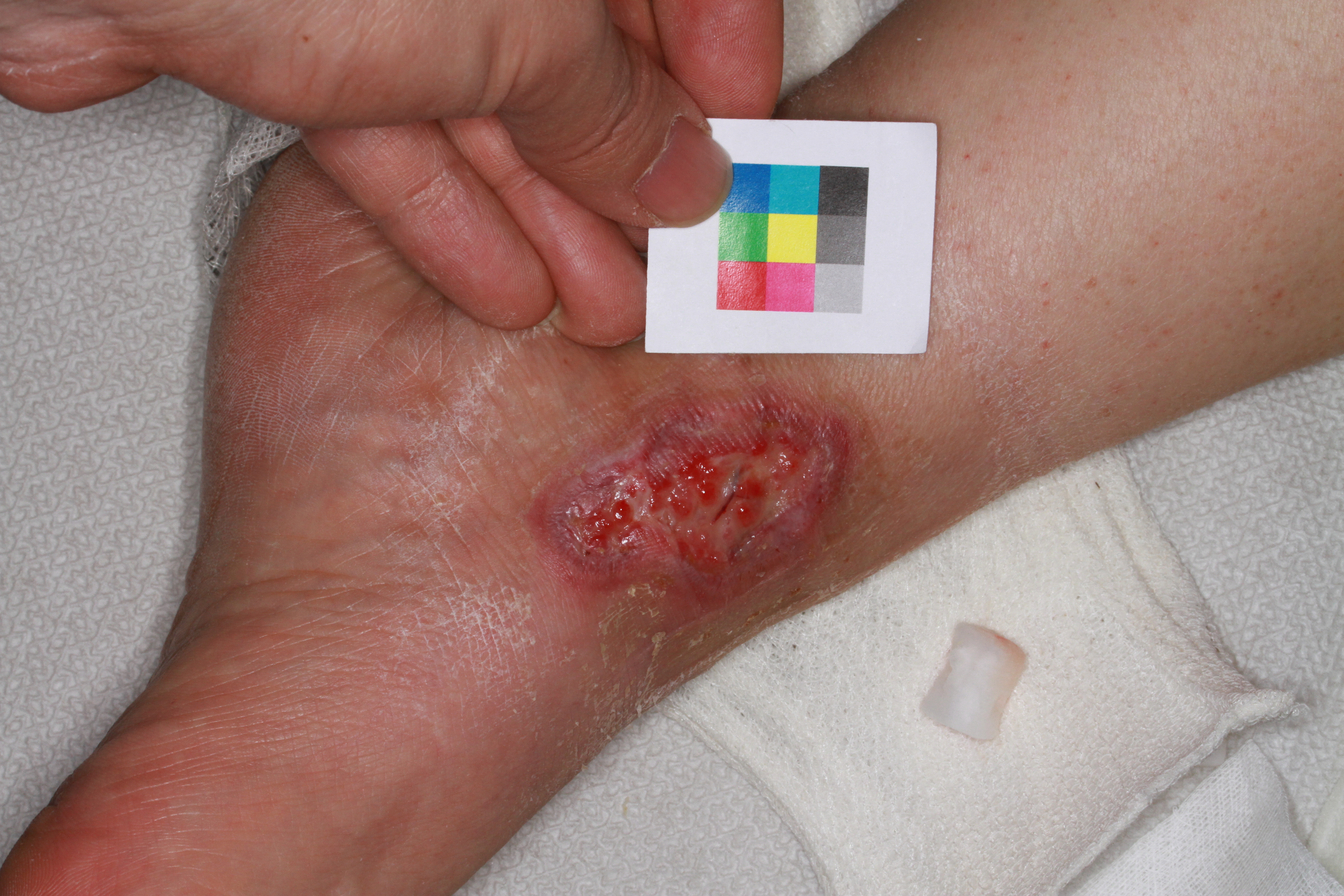

Supplement: S15 File — (ZIP) [file pone.0163092.s015.zip › 40218.JPG]

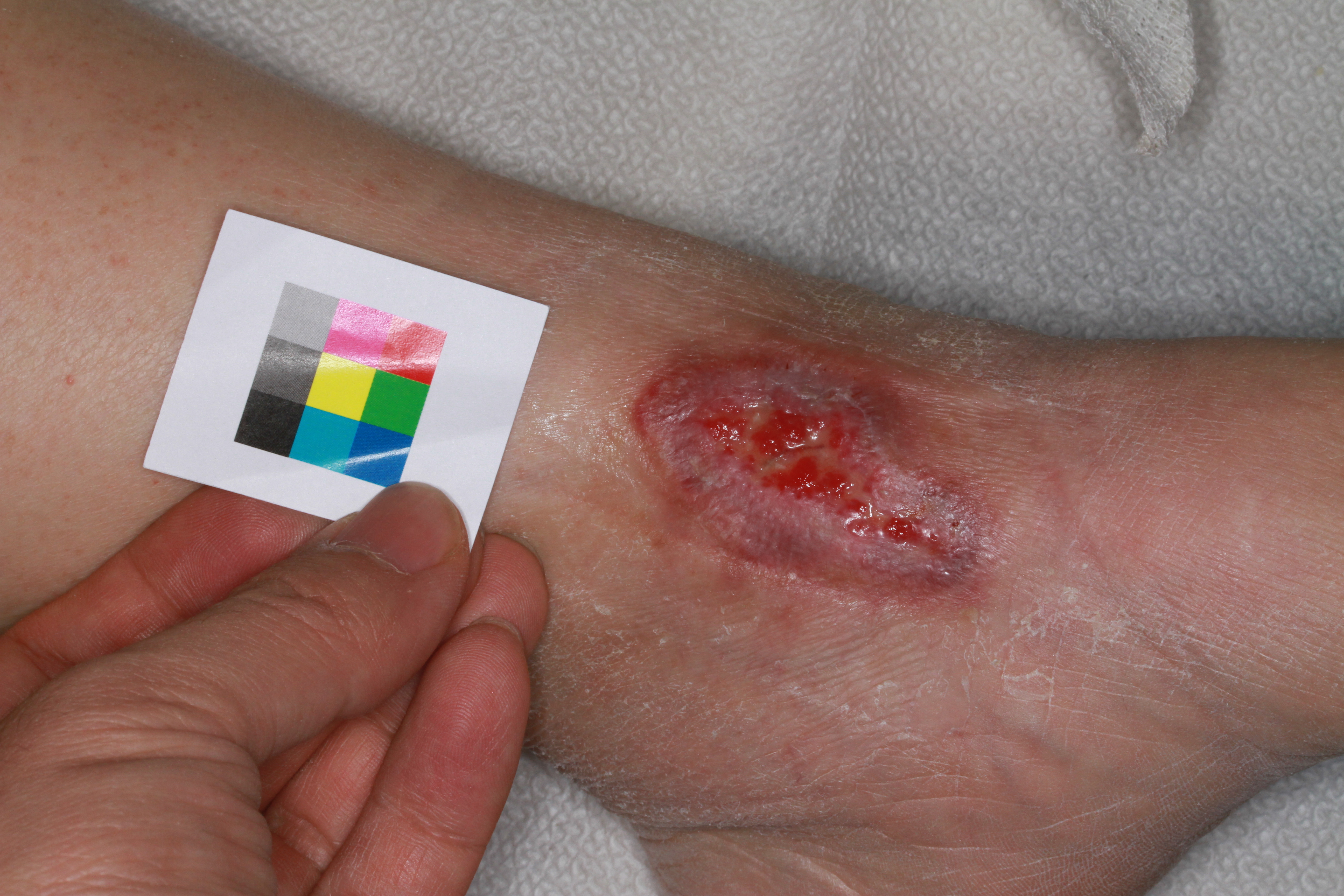

Supplement: S15 File — (ZIP) [file pone.0163092.s015.zip › 40225.JPG]

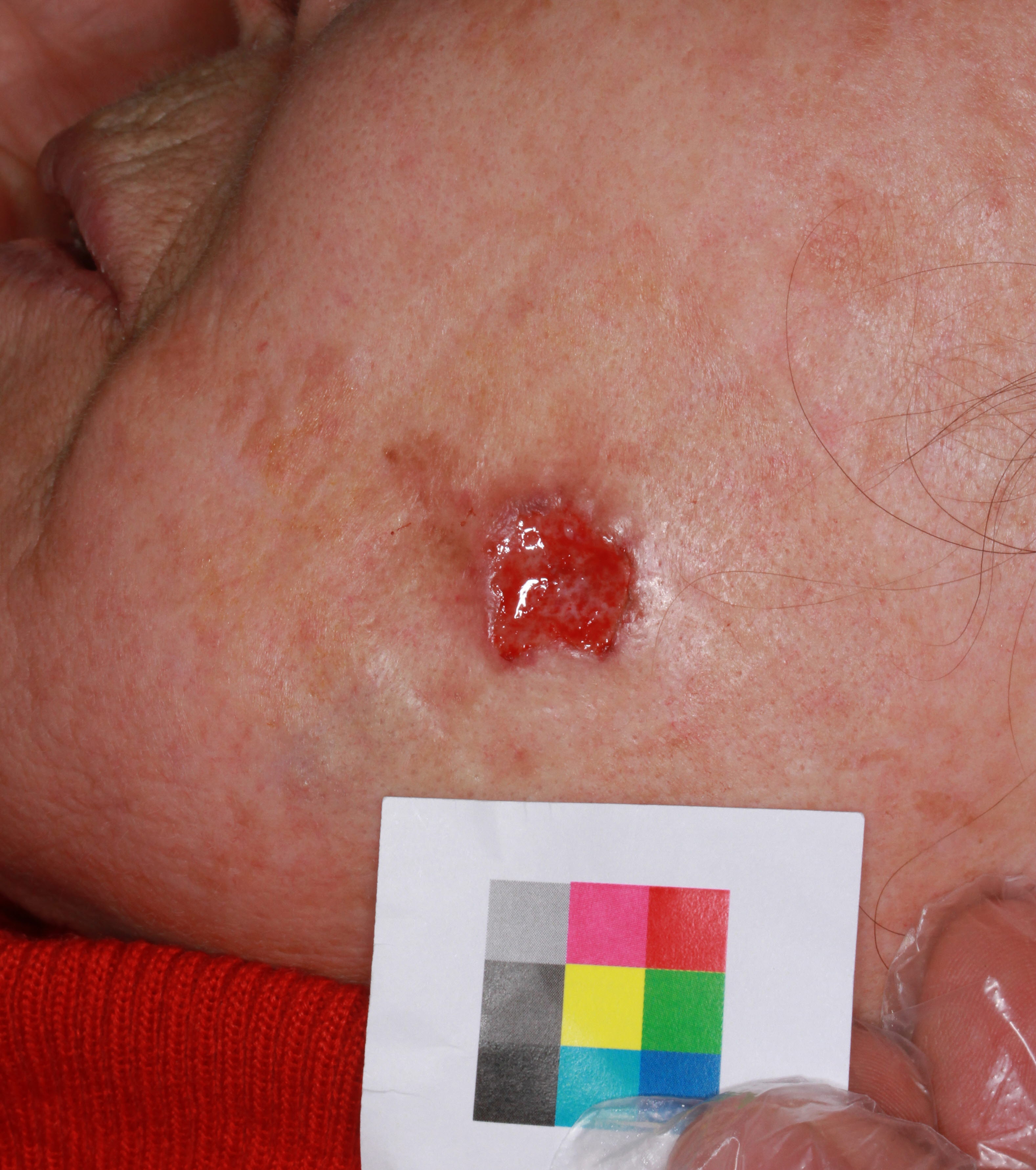

Supplement: S16 File — (ZIP) [file pone.0163092.s016.zip › 0219.jpg]

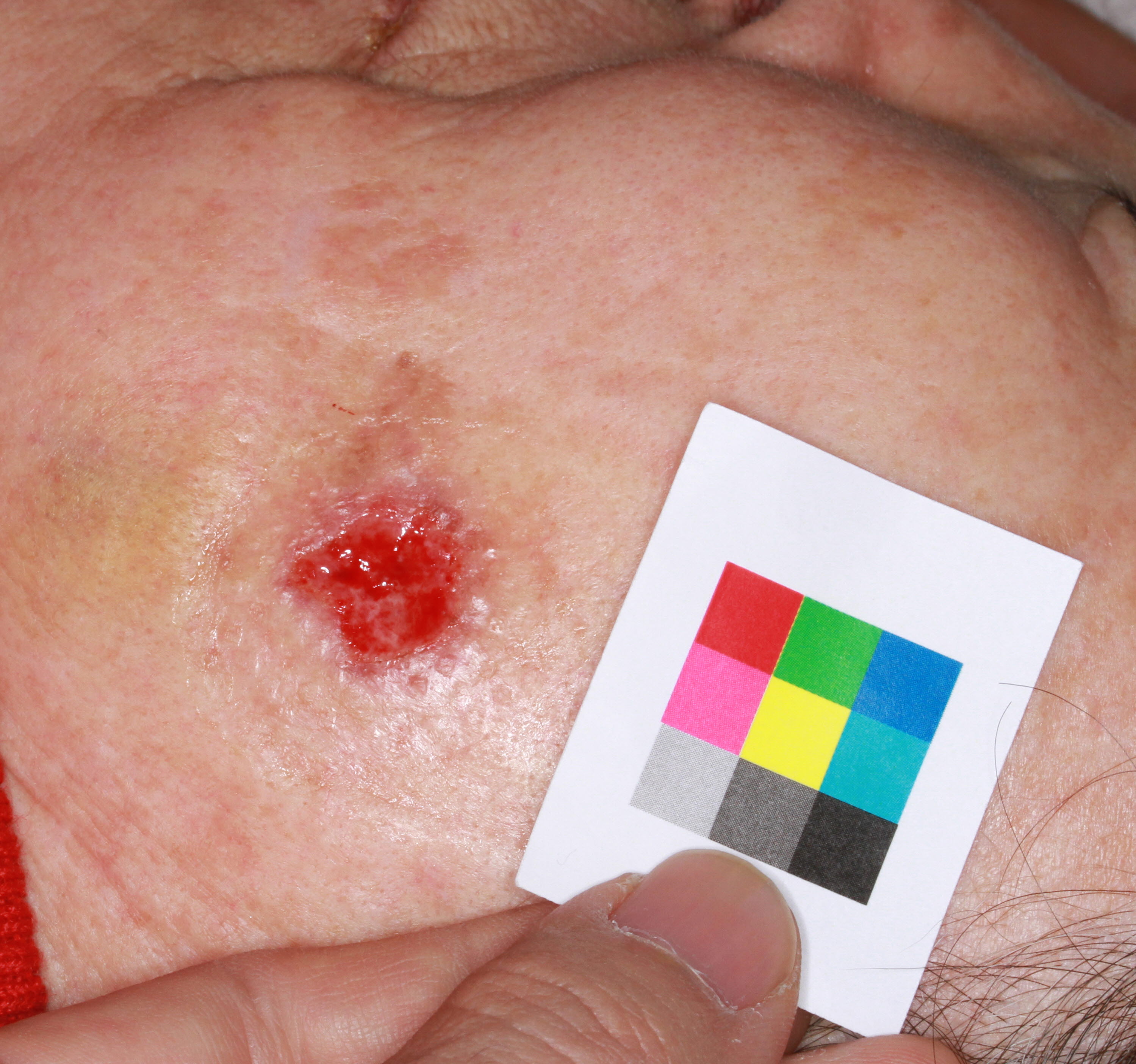

Supplement: S16 File — (ZIP) [file pone.0163092.s016.zip › 0221.jpg]

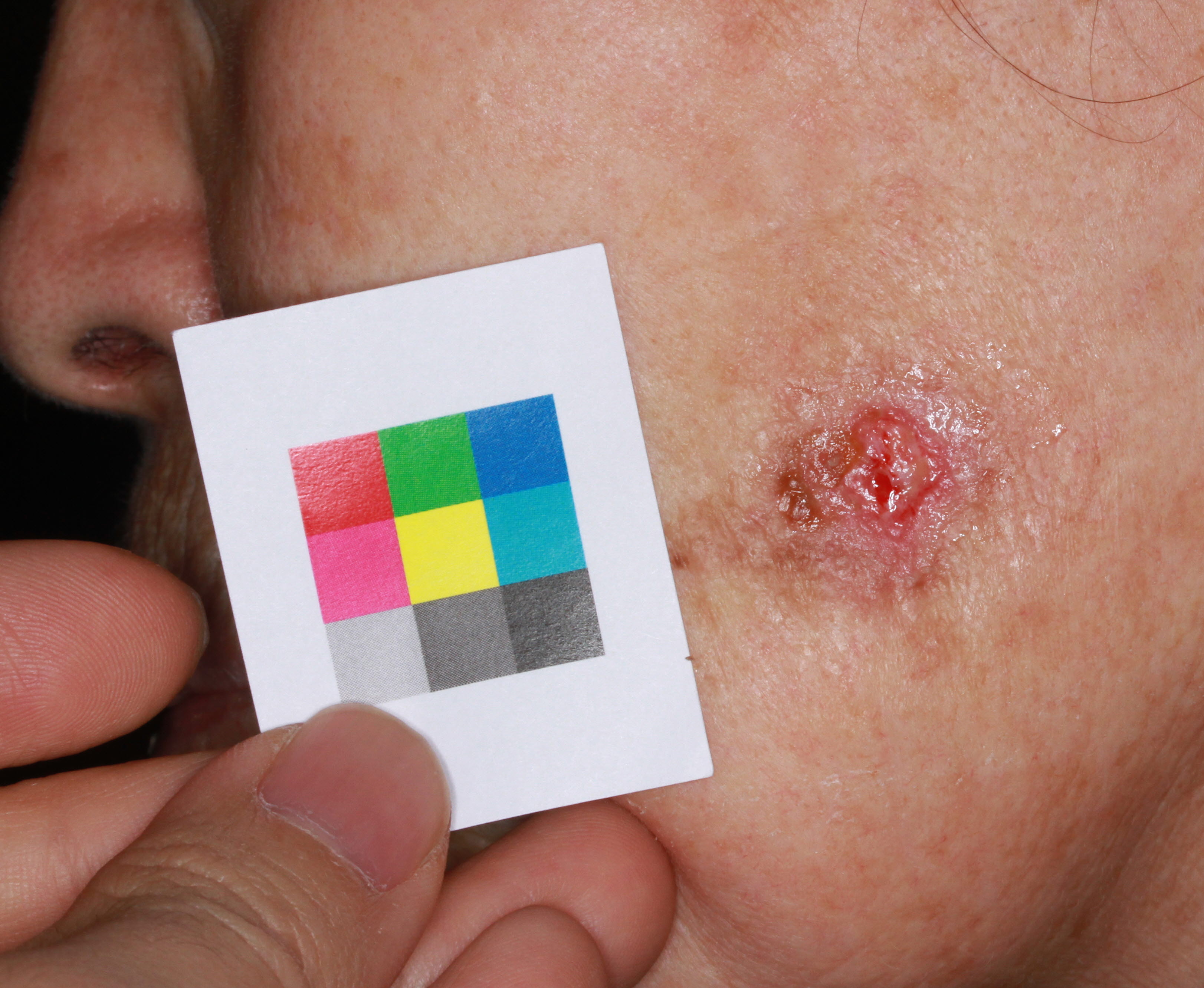

Supplement: S16 File — (ZIP) [file pone.0163092.s016.zip › 0307.jpg]

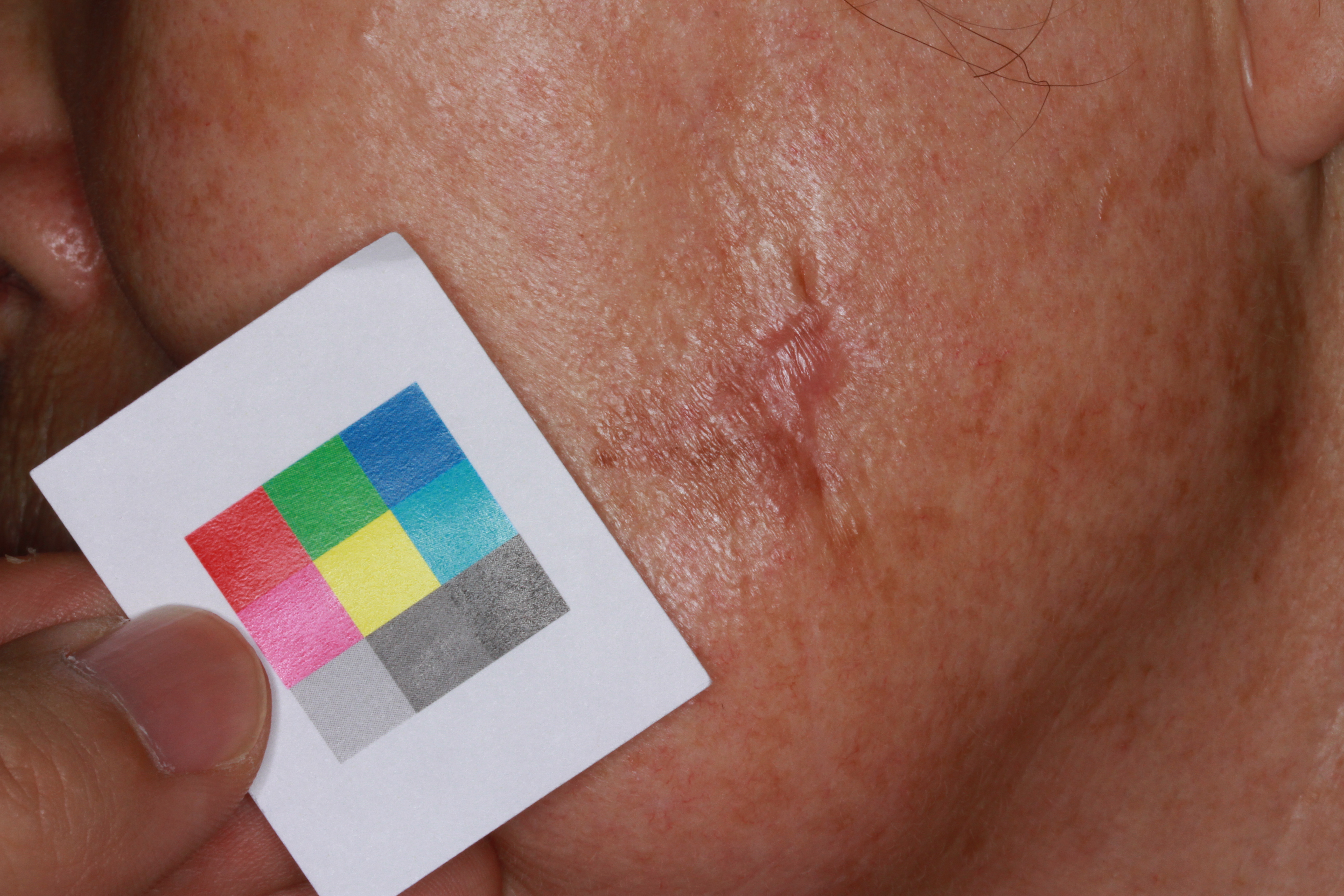

Supplement: S16 File — (ZIP) [file pone.0163092.s016.zip › 0611.JPG]

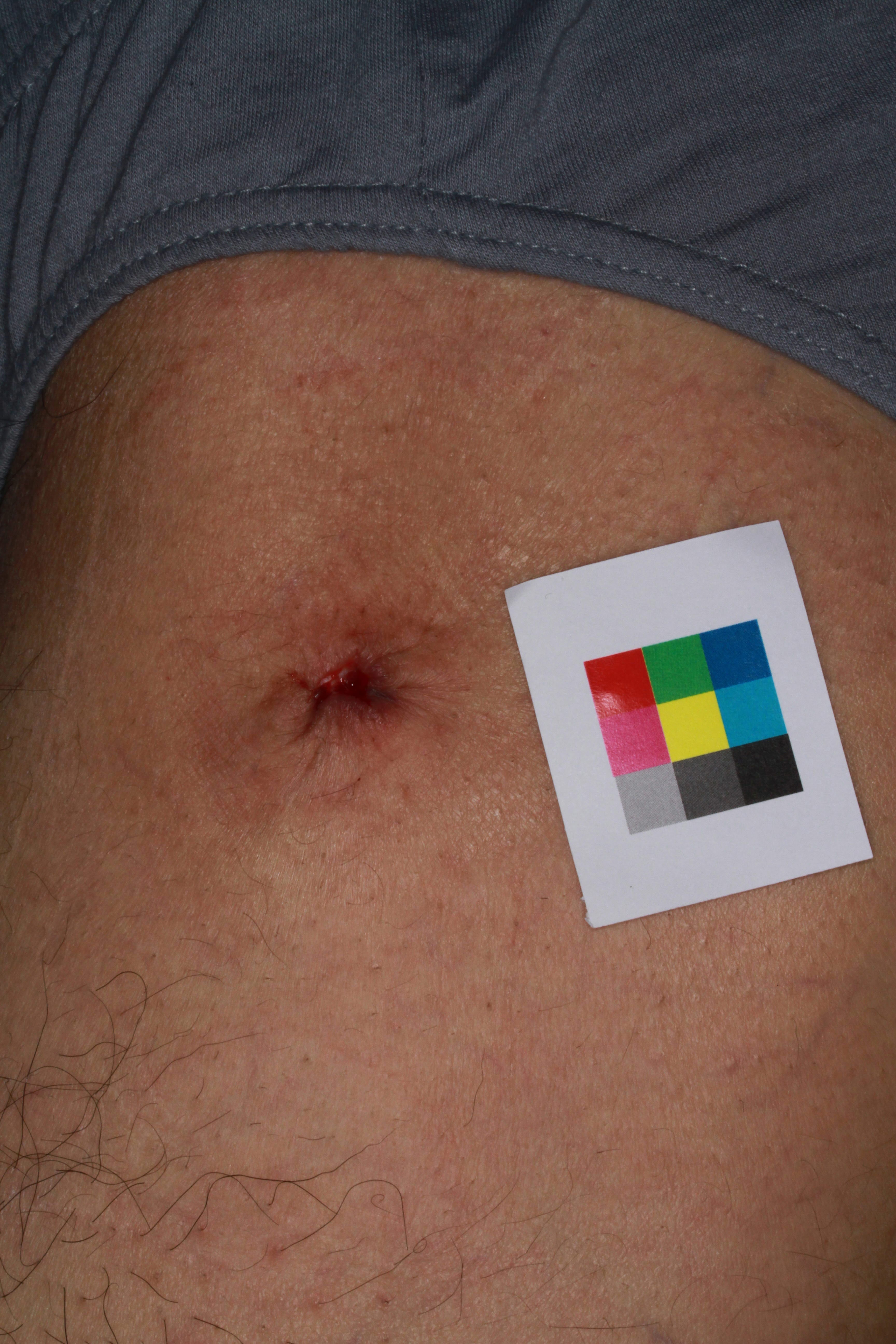

Supplement: S17 File — (ZIP) [file pone.0163092.s017.zip › 31127.JPG]

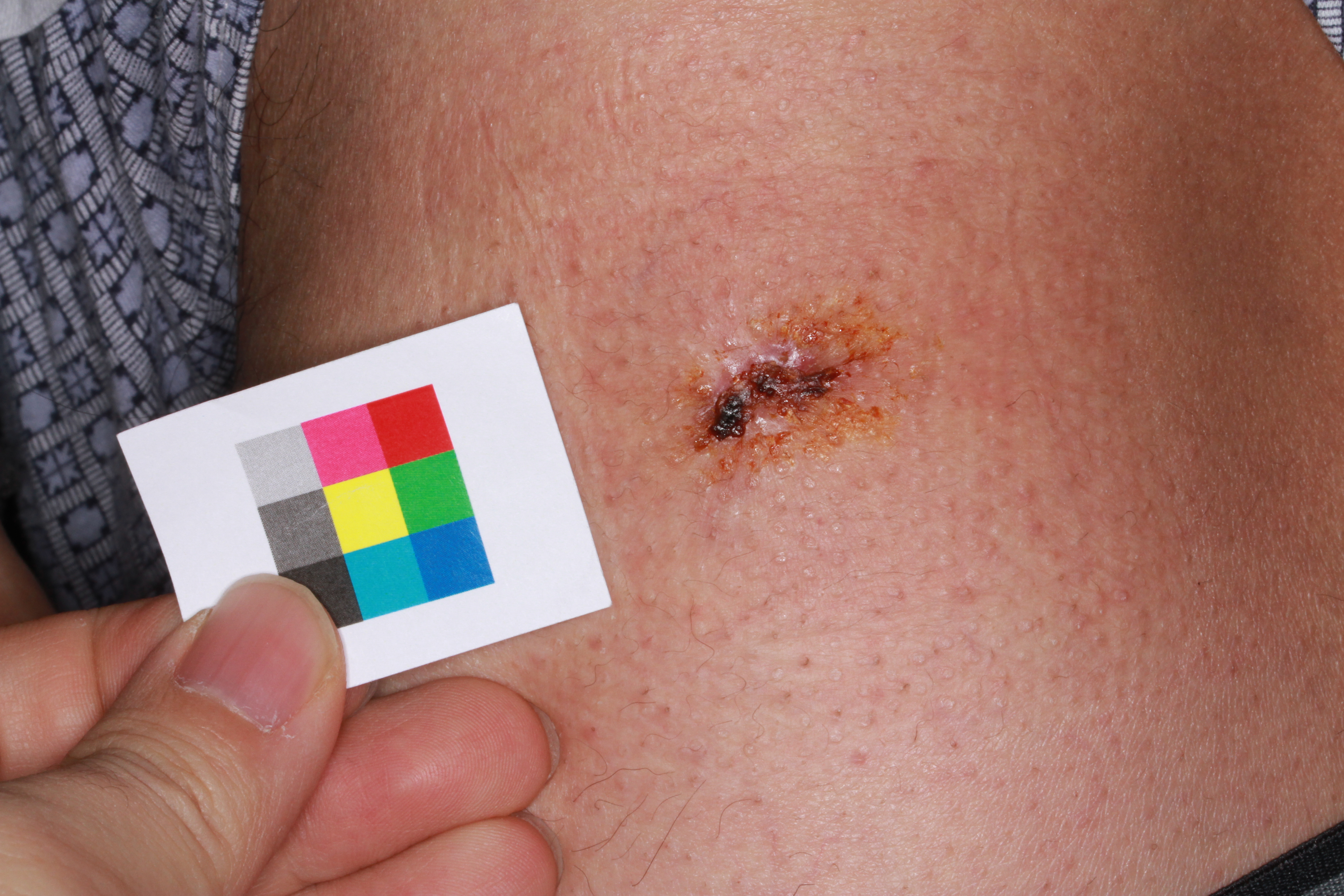

Supplement: S17 File — (ZIP) [file pone.0163092.s017.zip › 31226.JPG]

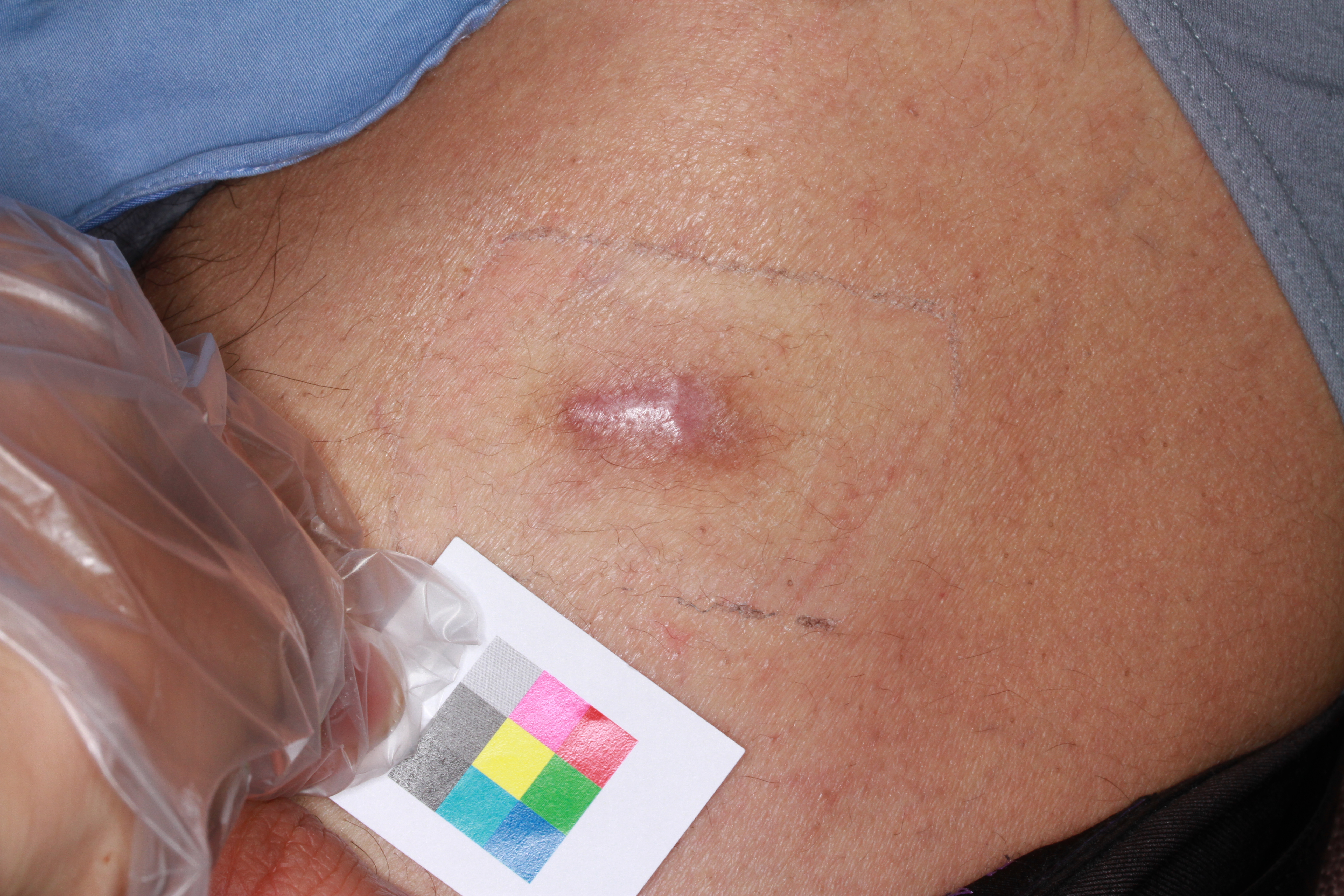

Supplement: S17 File — (ZIP) [file pone.0163092.s017.zip › 40314.JPG]

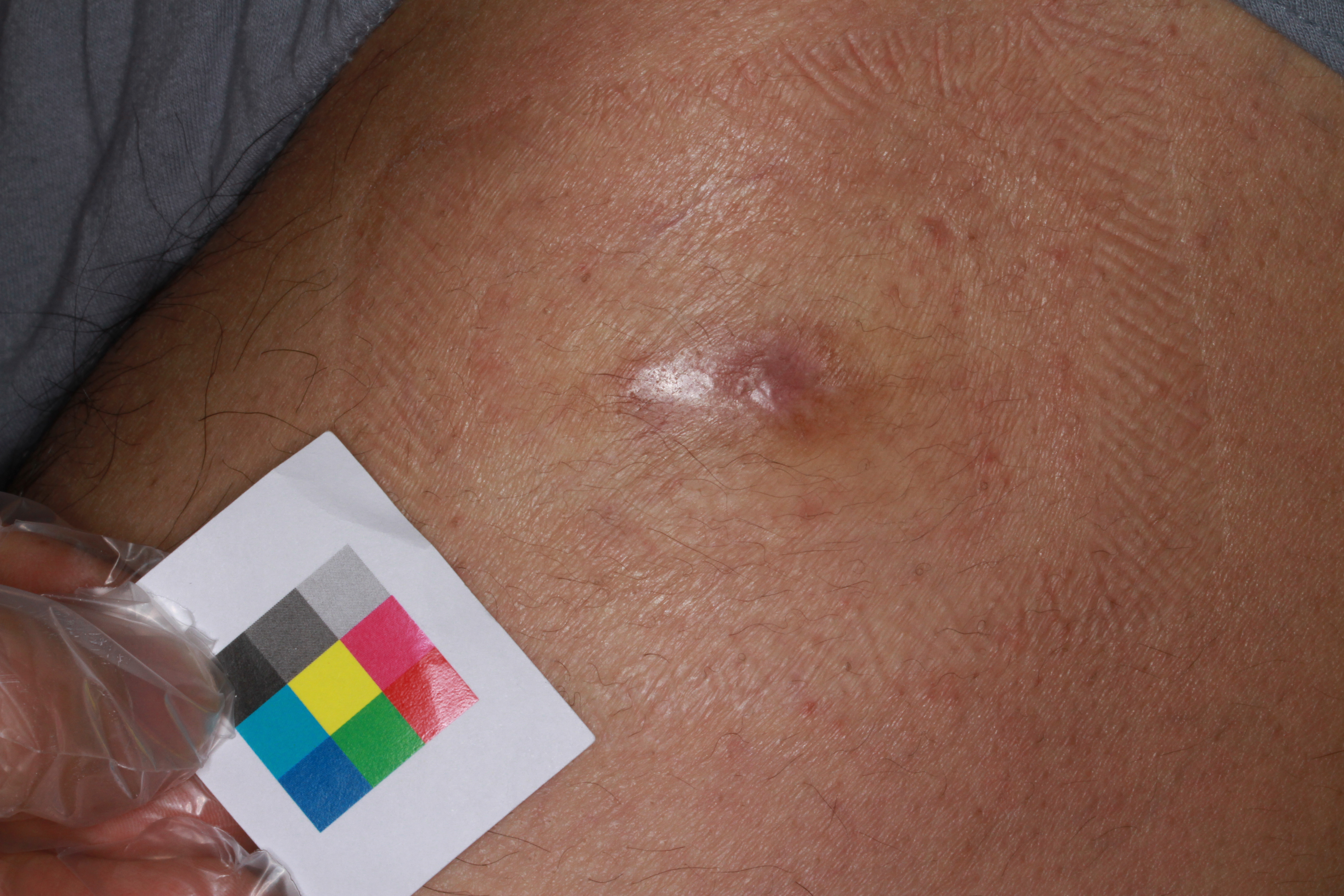

Supplement: S17 File — (ZIP) [file pone.0163092.s017.zip › 40411.JPG]

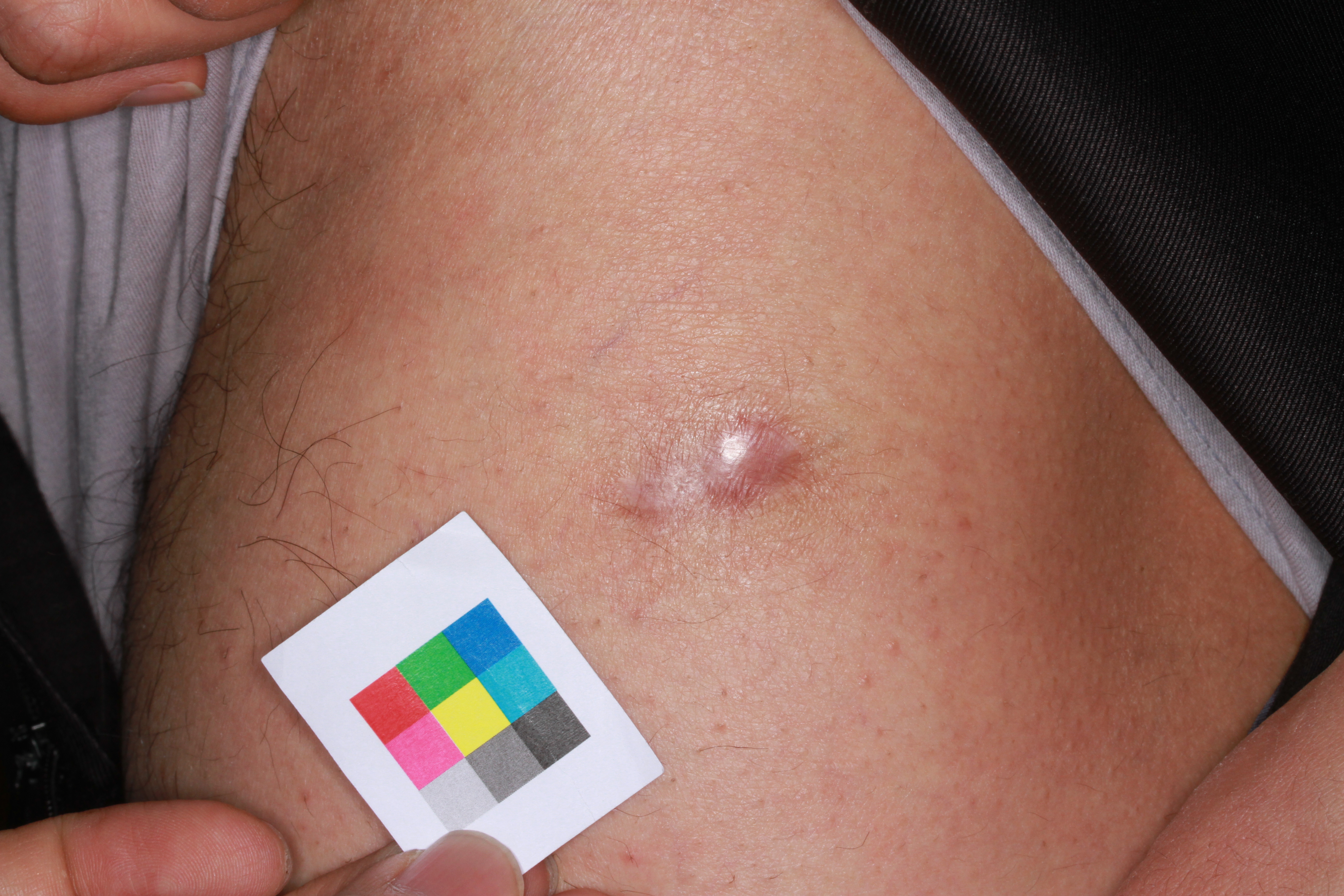

Supplement: S17 File — (ZIP) [file pone.0163092.s017.zip › 50123.JPG]

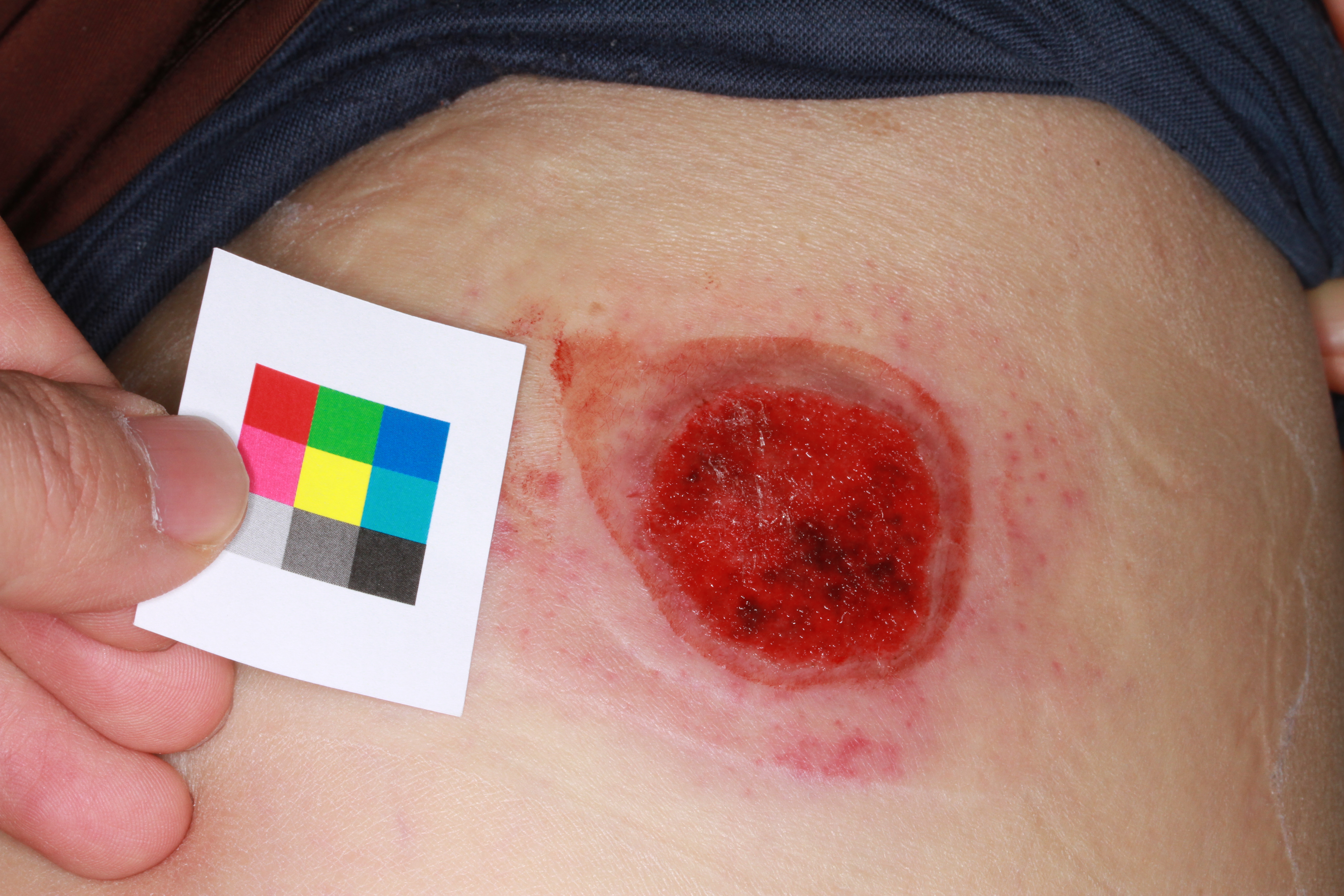

Supplement: S18 File — (ZIP) [file pone.0163092.s018.zip › 0305.JPG]

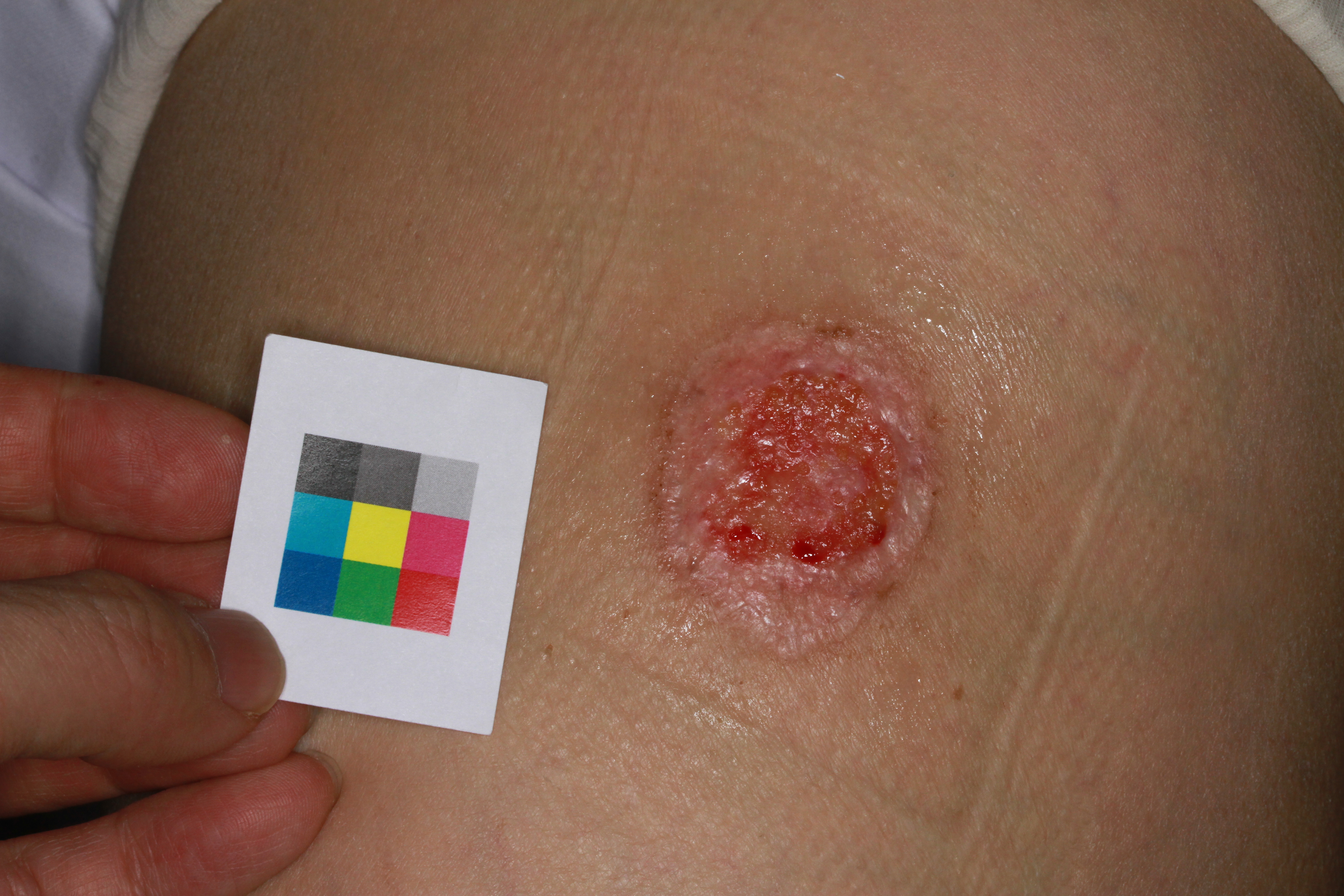

Supplement: S18 File — (ZIP) [file pone.0163092.s018.zip › 0325.JPG]

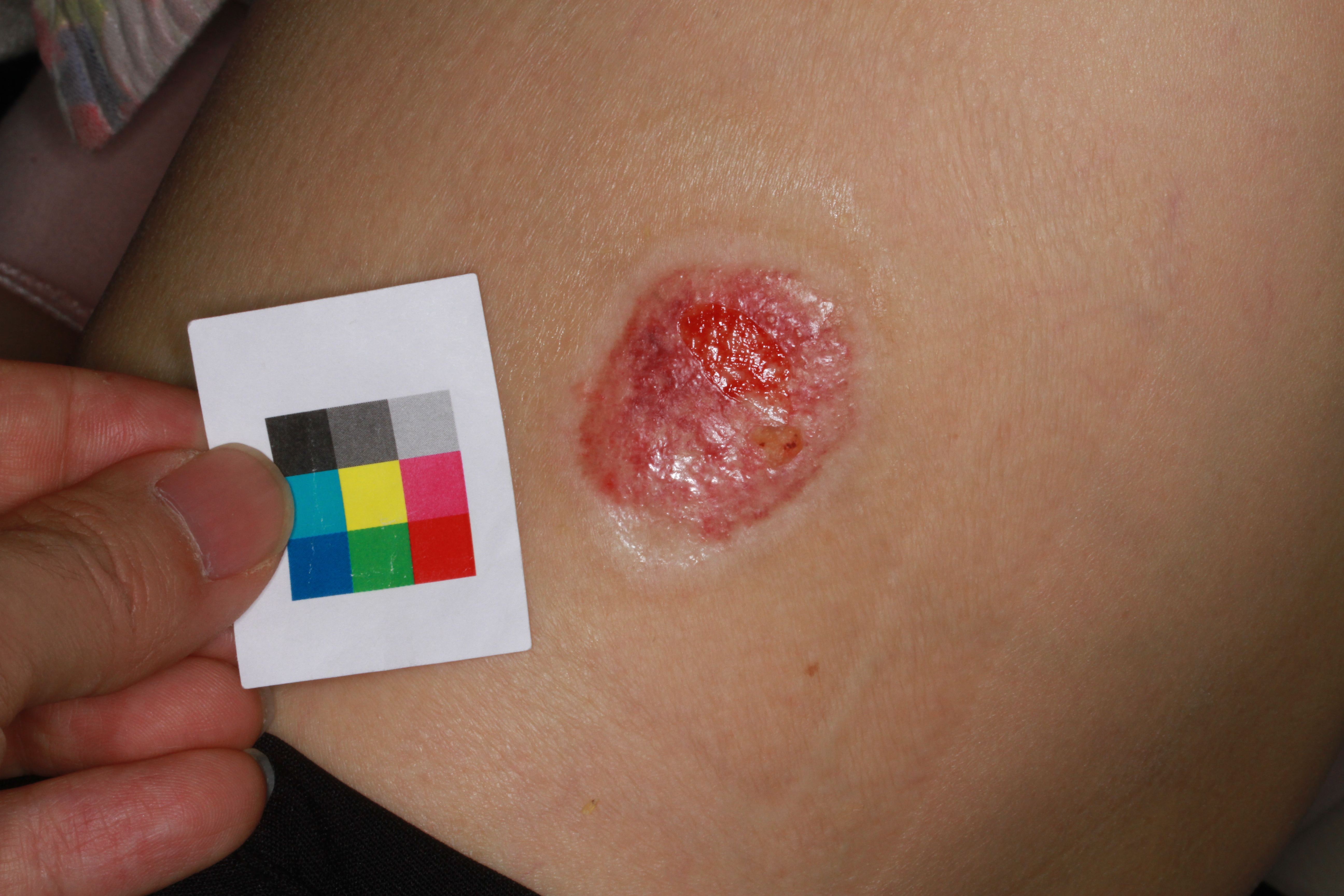

Supplement: S18 File — (ZIP) [file pone.0163092.s018.zip › 0422.JPG]

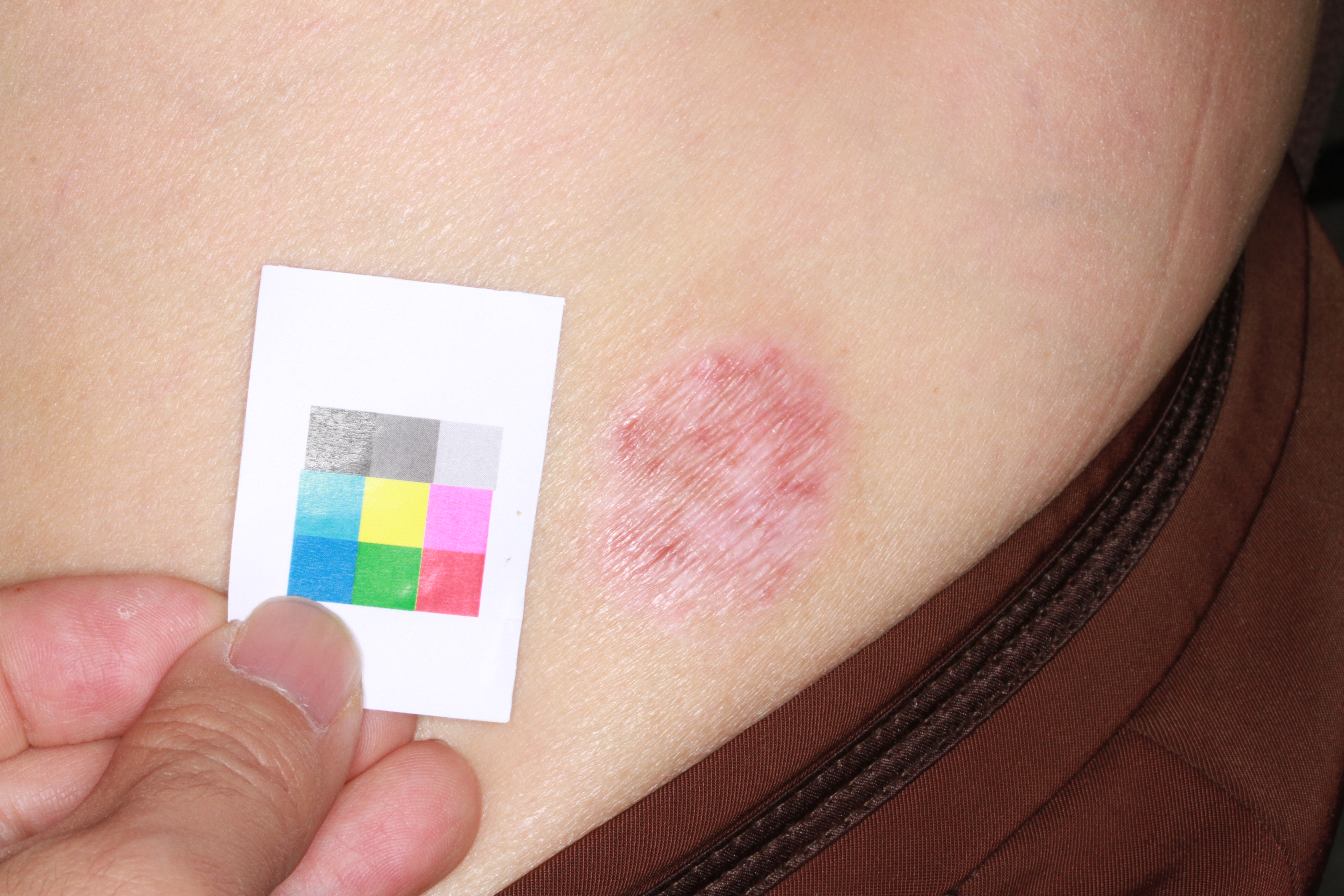

Supplement: S18 File — (ZIP) [file pone.0163092.s018.zip › 1031.JPG]

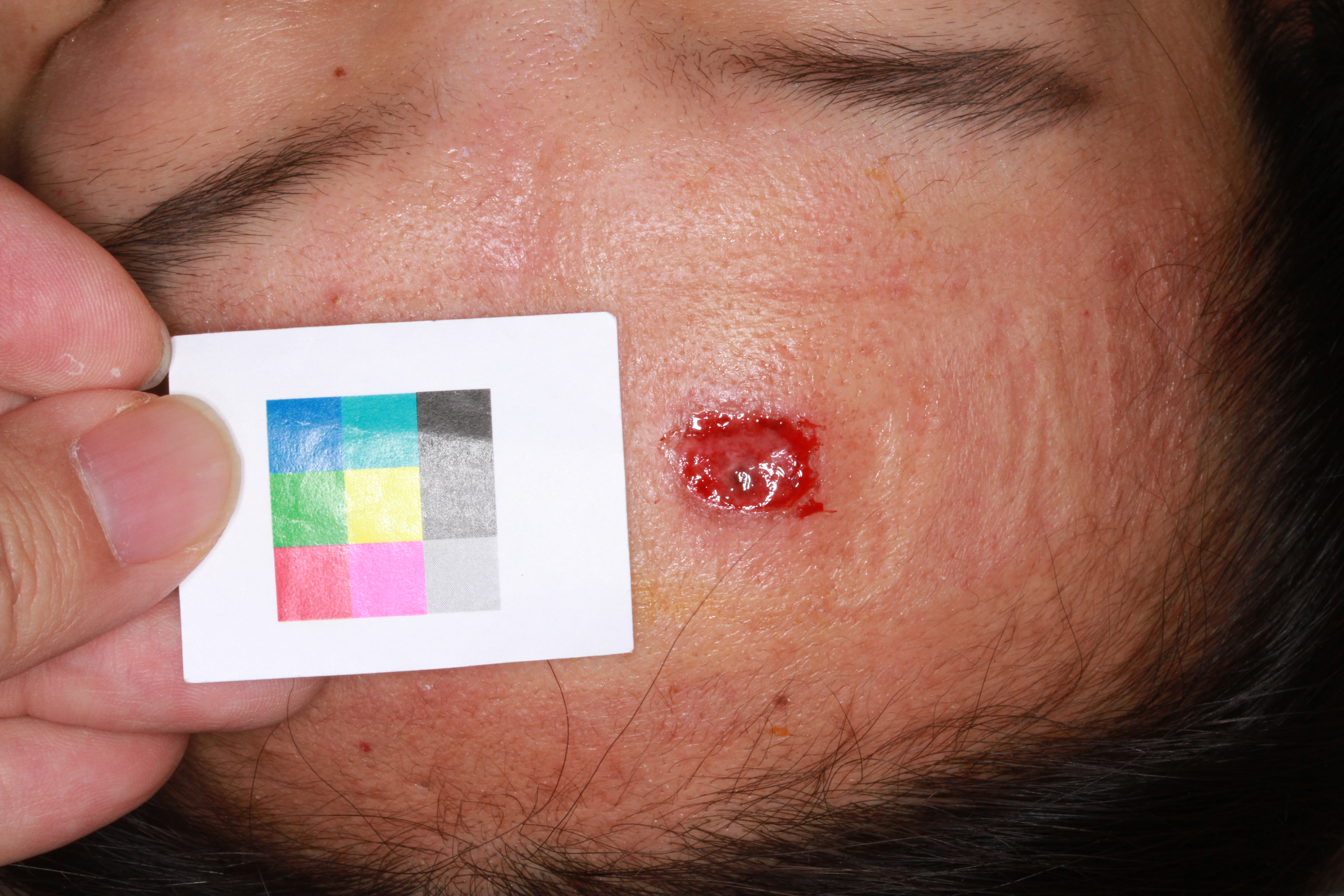

Supplement: S19 File — (ZIP) [file pone.0163092.s019.zip › 0528.JPG]

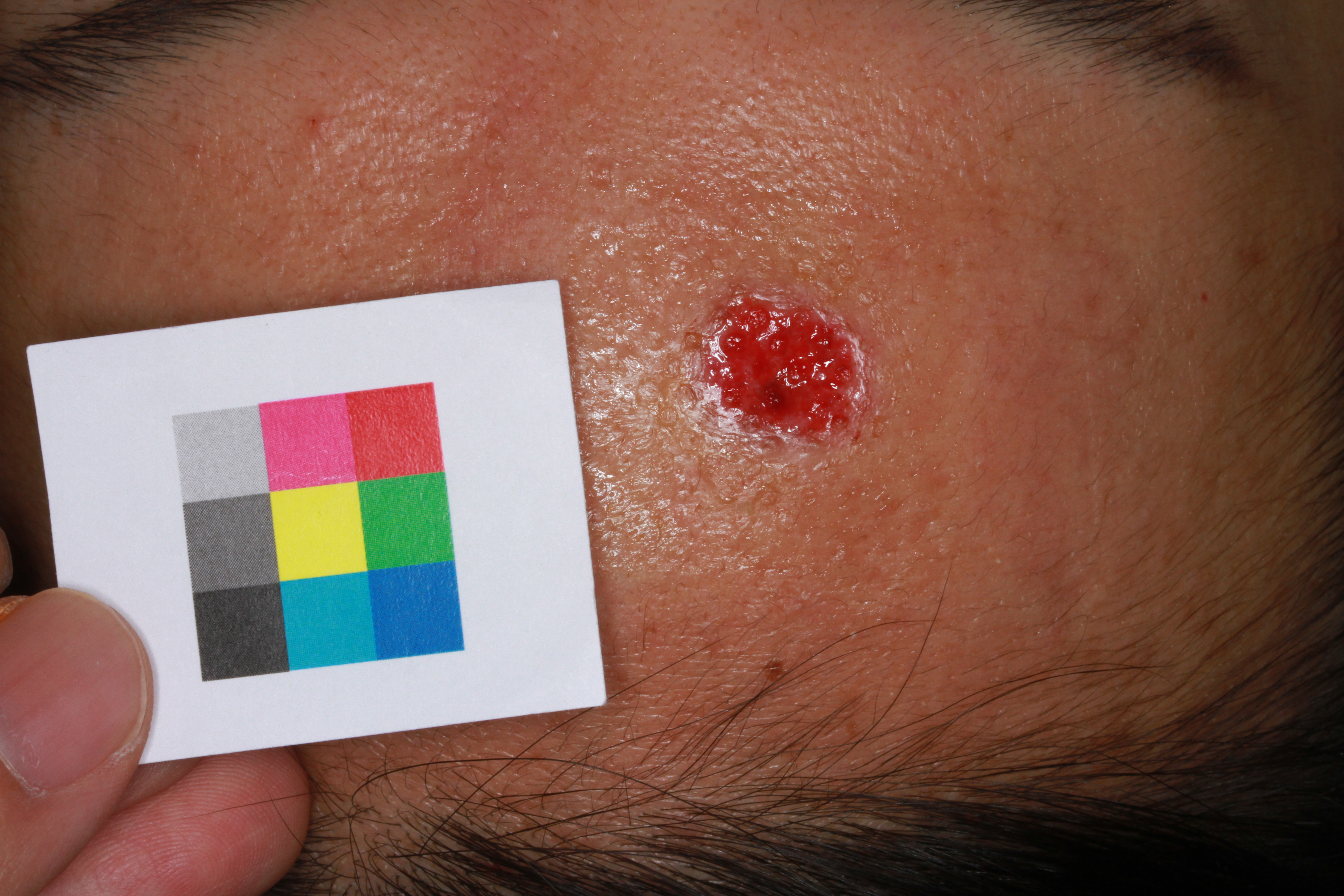

Supplement: S19 File — (ZIP) [file pone.0163092.s019.zip › 0530.JPG]

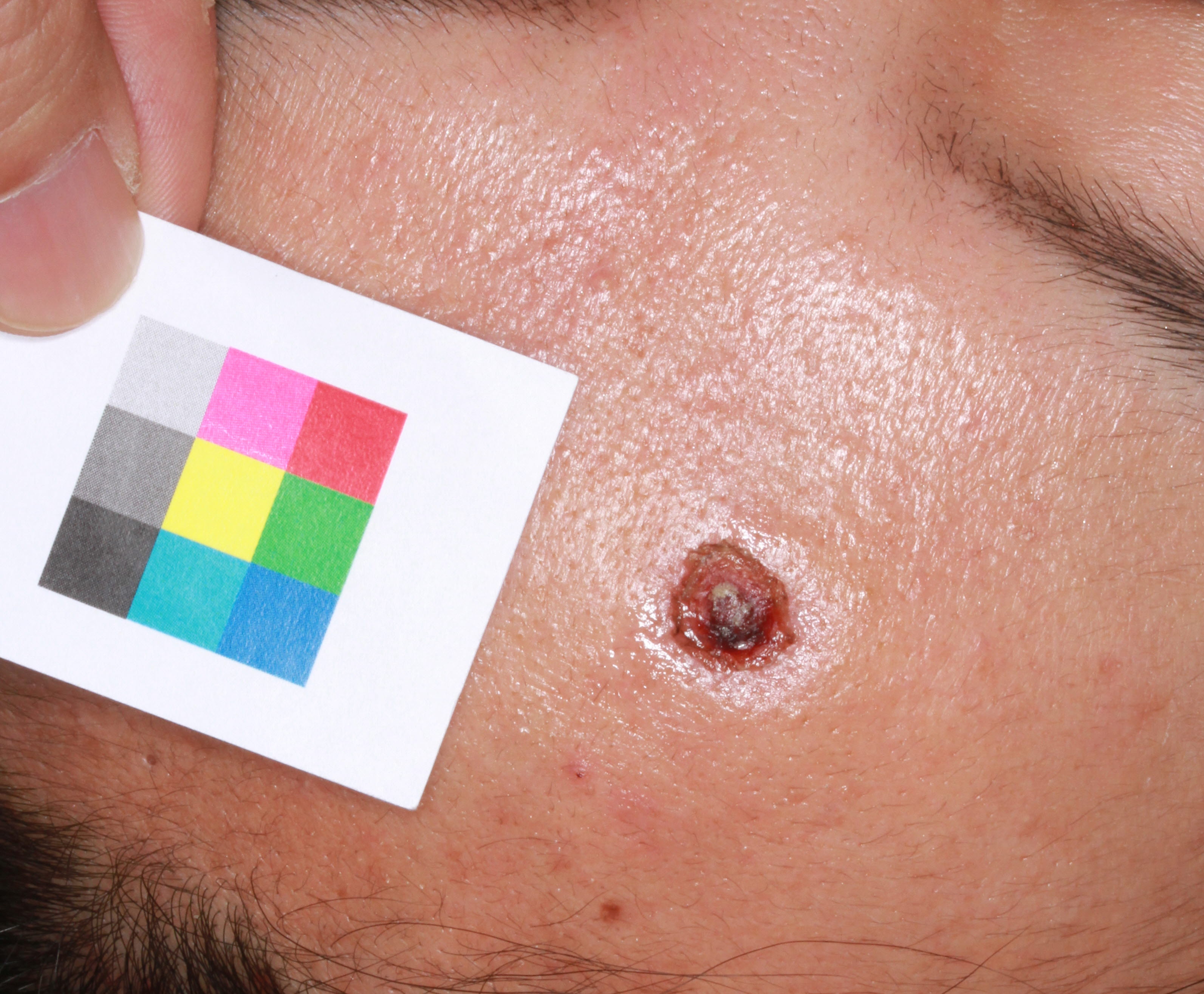

Supplement: S19 File — (ZIP) [file pone.0163092.s019.zip › 0610.jpg]

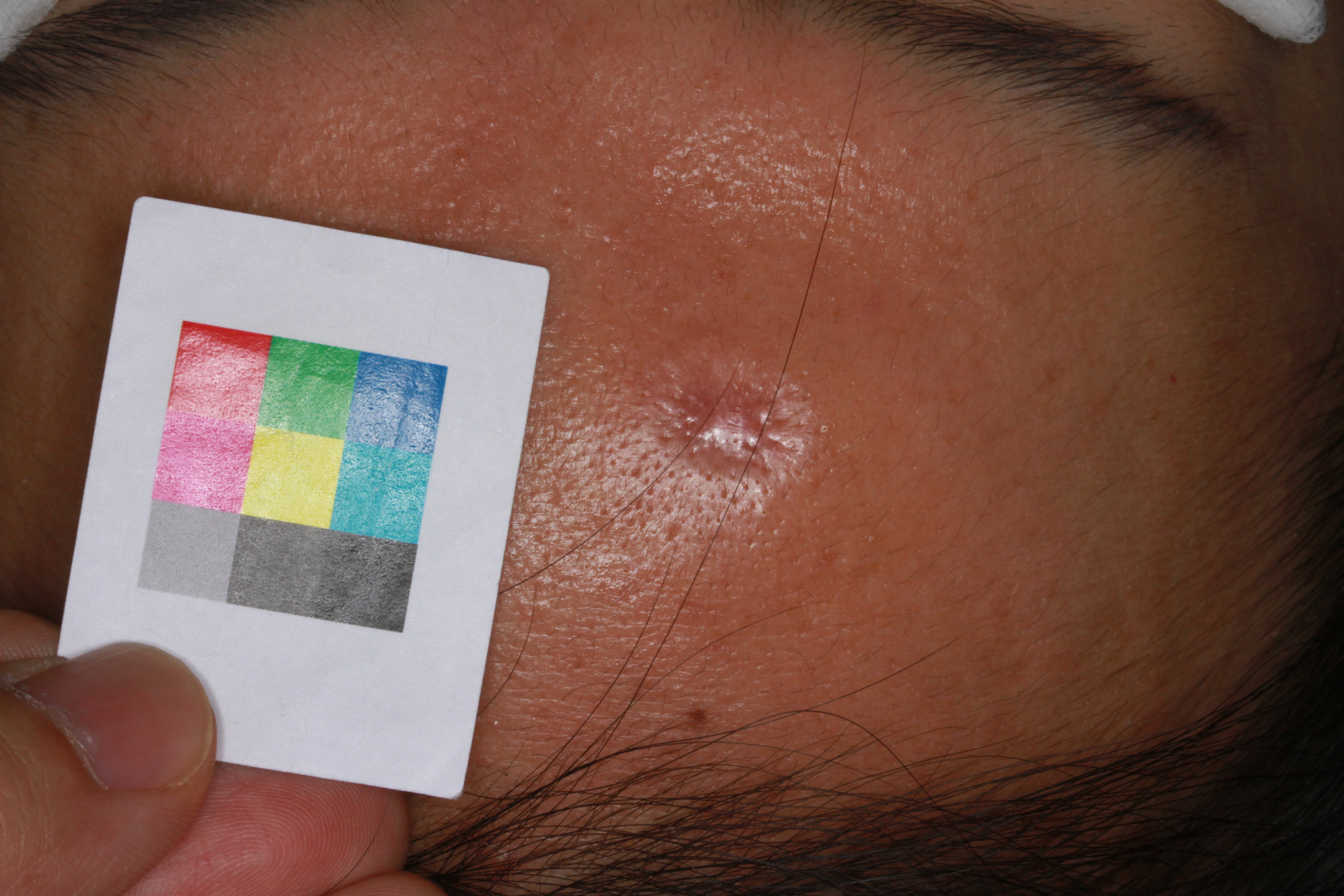

Supplement: S19 File — (ZIP) [file pone.0163092.s019.zip › 0624.JPG]

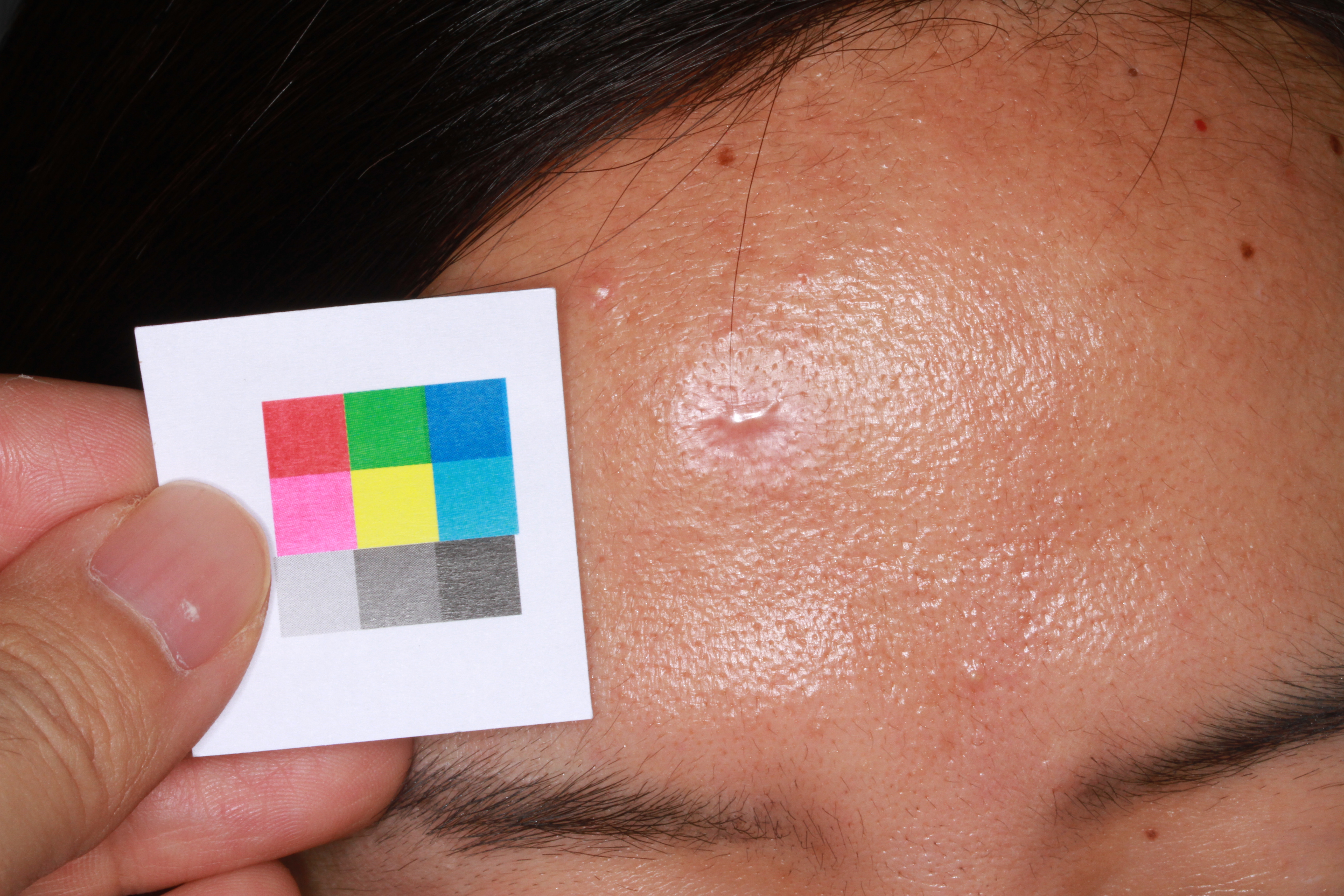

Supplement: S19 File — (ZIP) [file pone.0163092.s019.zip › 0722.JPG]

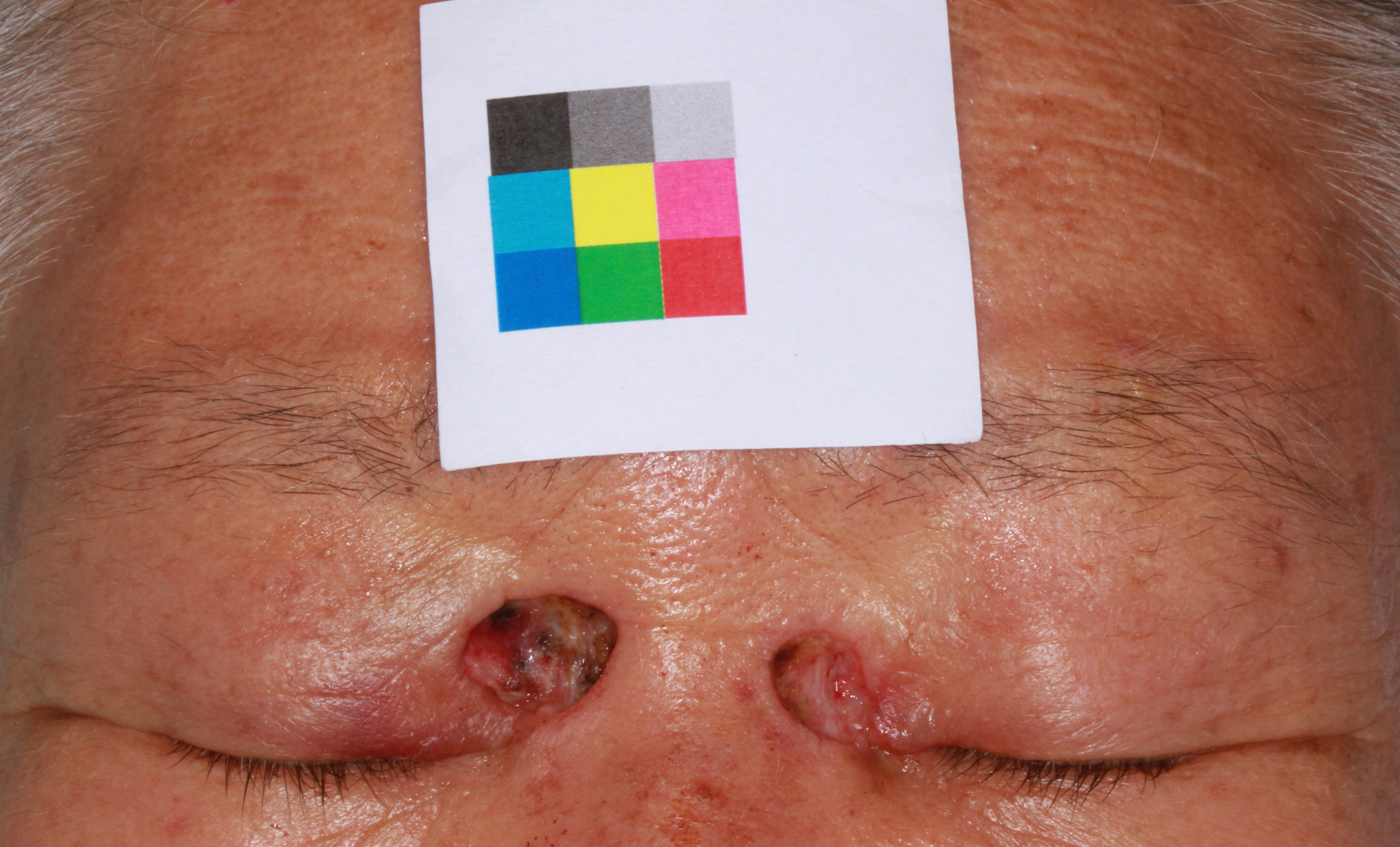

Supplement: S20 File — (ZIP) [file pone.0163092.s020.zip › 0915.jpg]

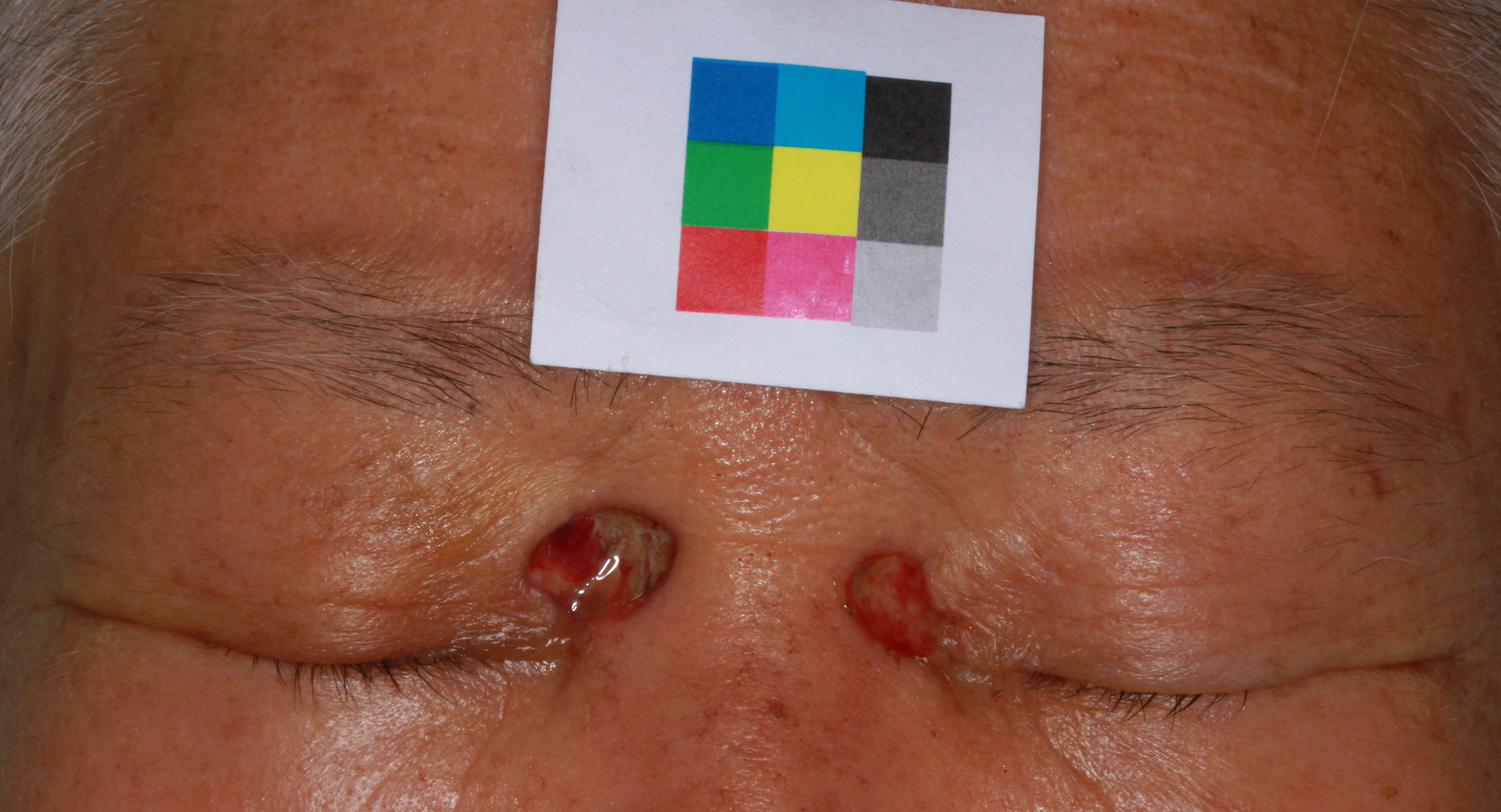

Supplement: S20 File — (ZIP) [file pone.0163092.s020.zip › 0917.jpg]

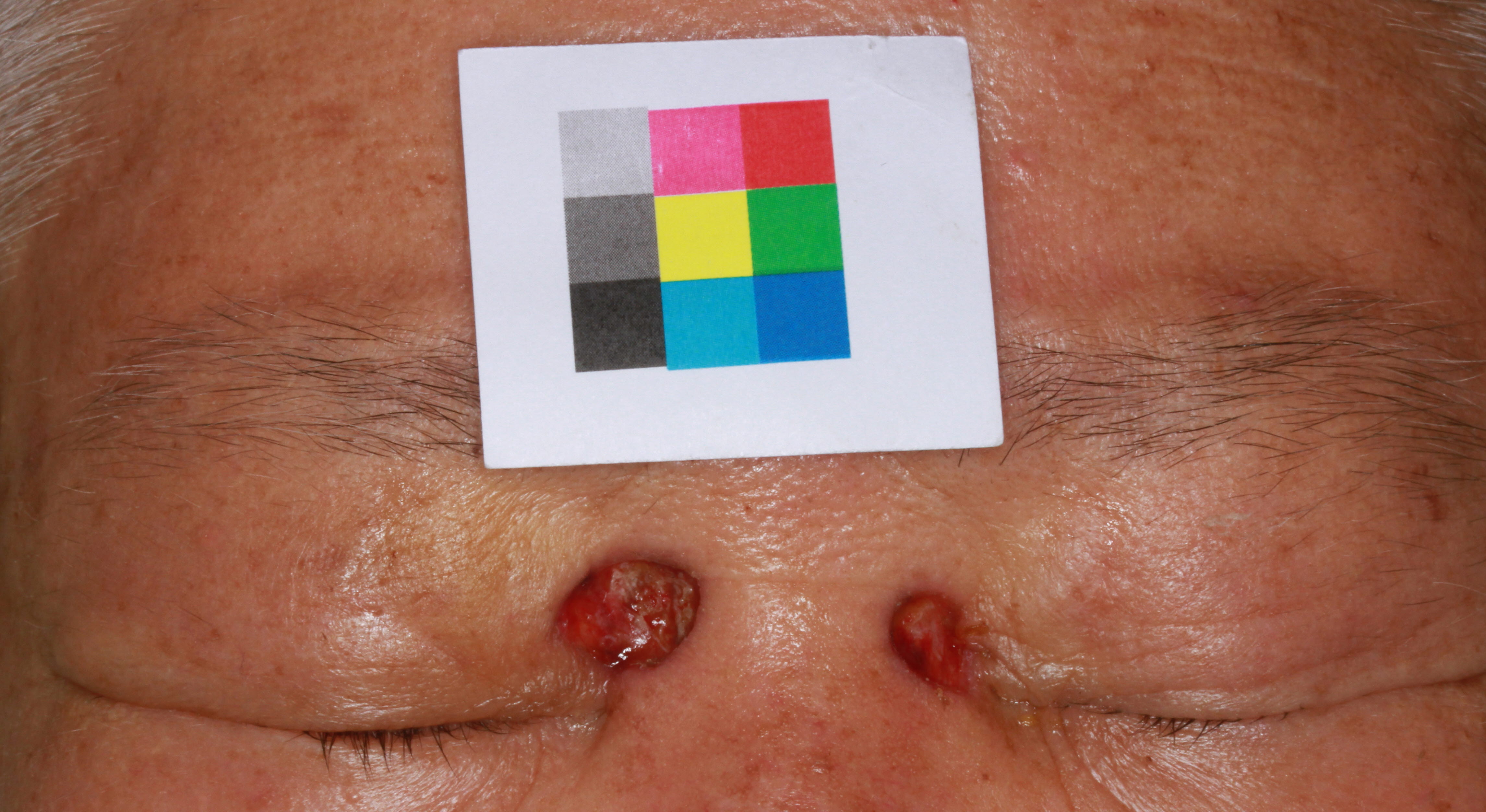

Supplement: S20 File — (ZIP) [file pone.0163092.s020.zip › 0921.jpg]

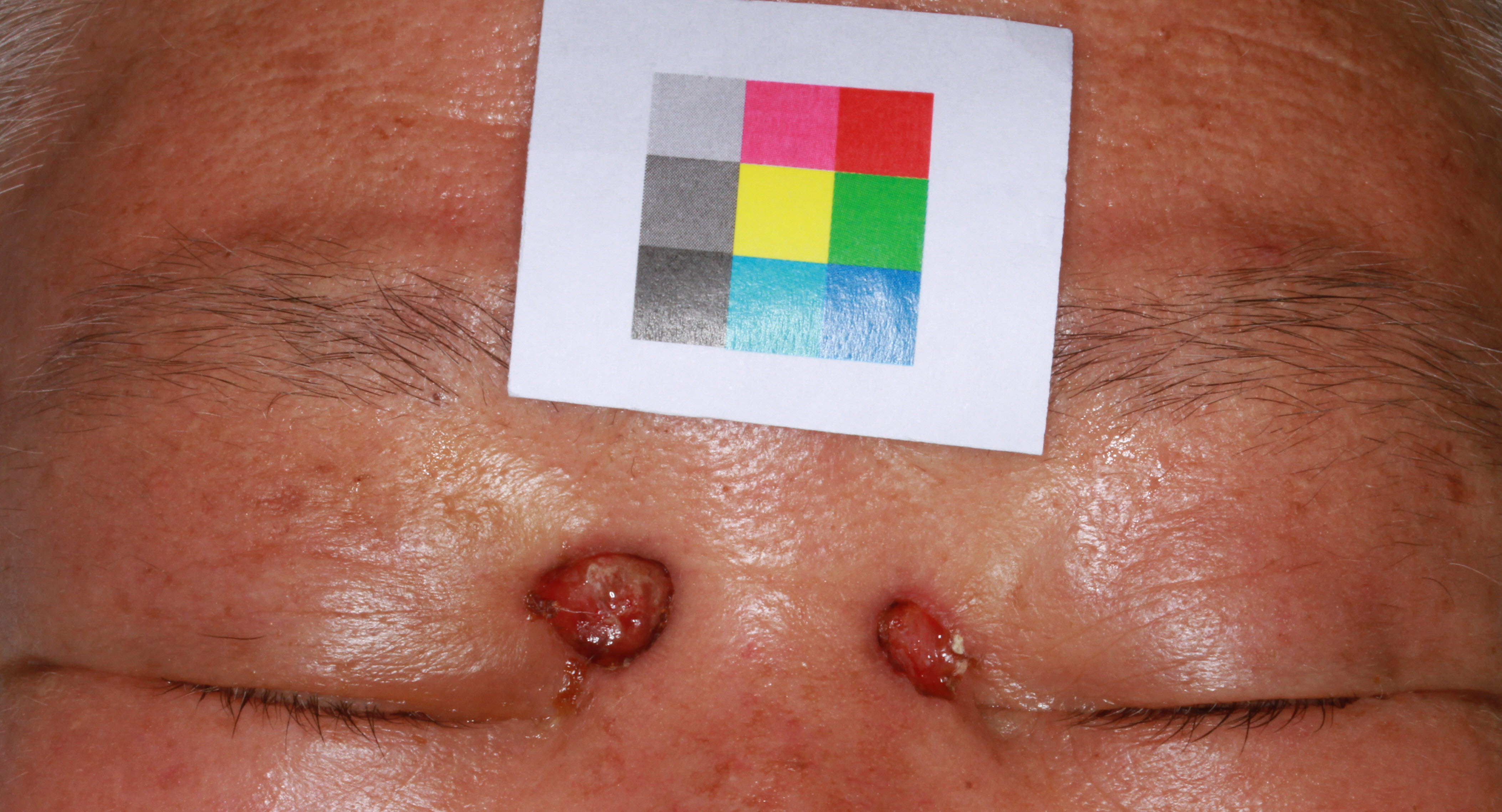

Supplement: S20 File — (ZIP) [file pone.0163092.s020.zip › 0923.jpg]

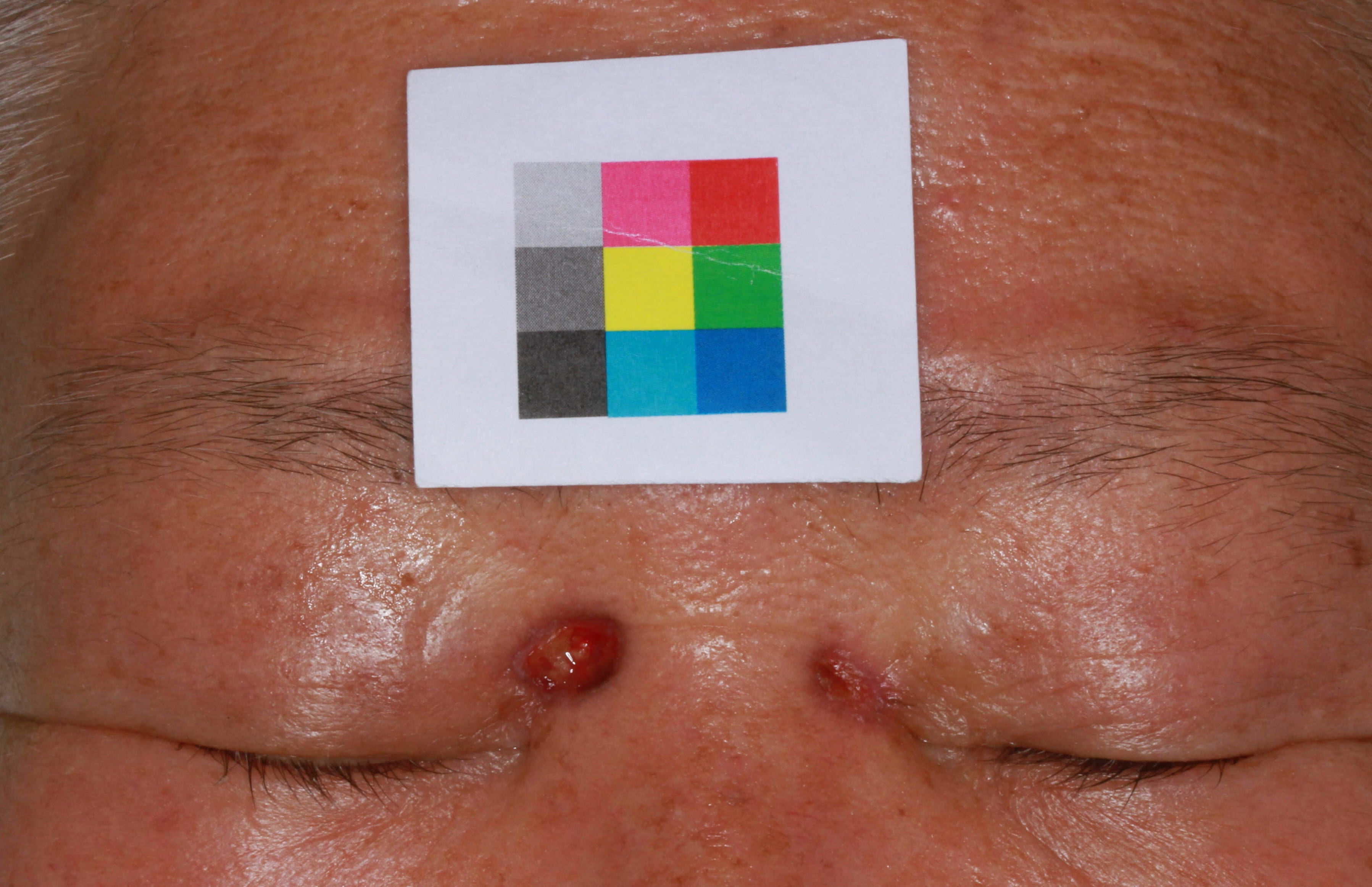

Supplement: S20 File — (ZIP) [file pone.0163092.s020.zip › 0930.jpg]

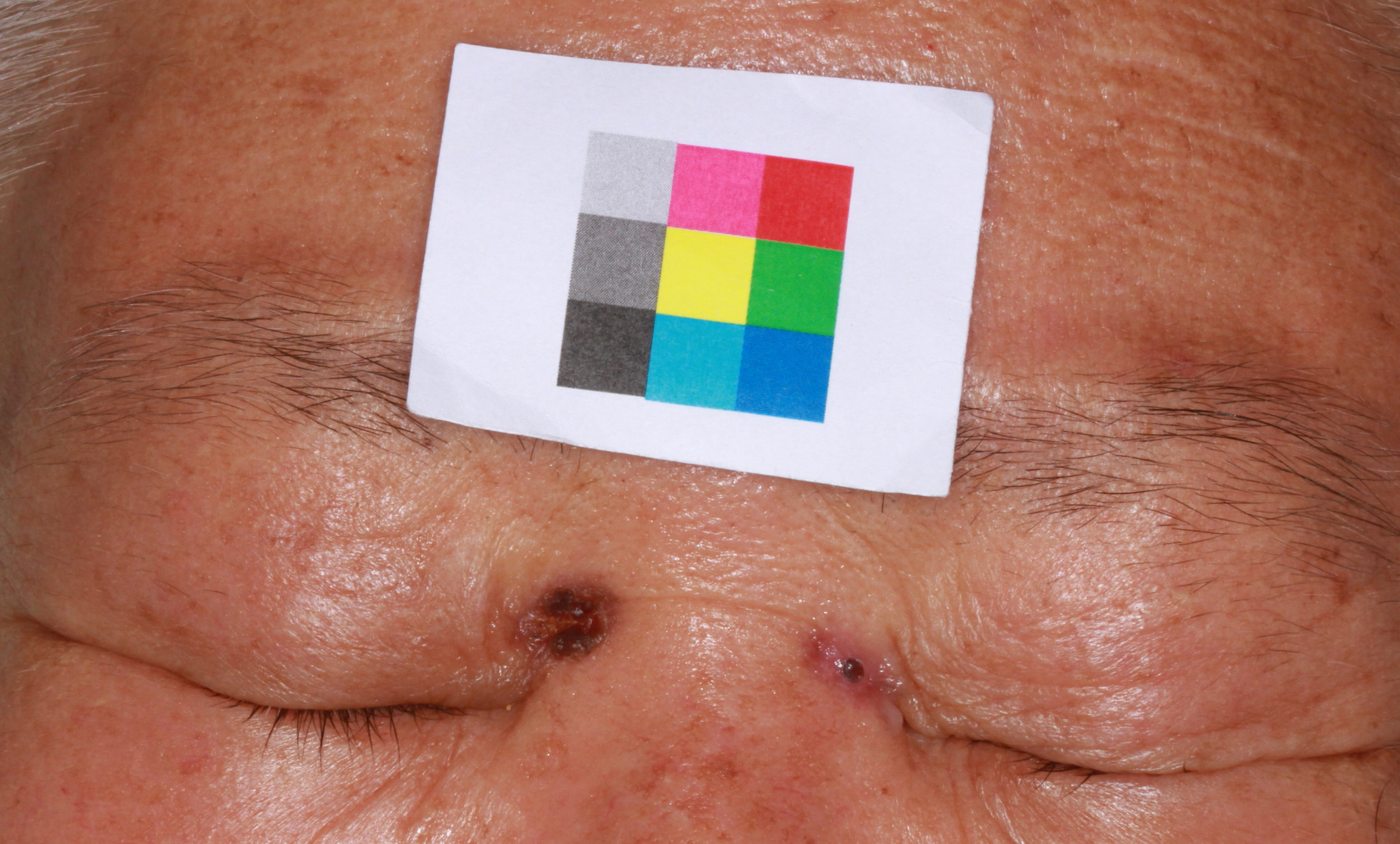

Supplement: S20 File — (ZIP) [file pone.0163092.s020.zip › 1006.jpg]

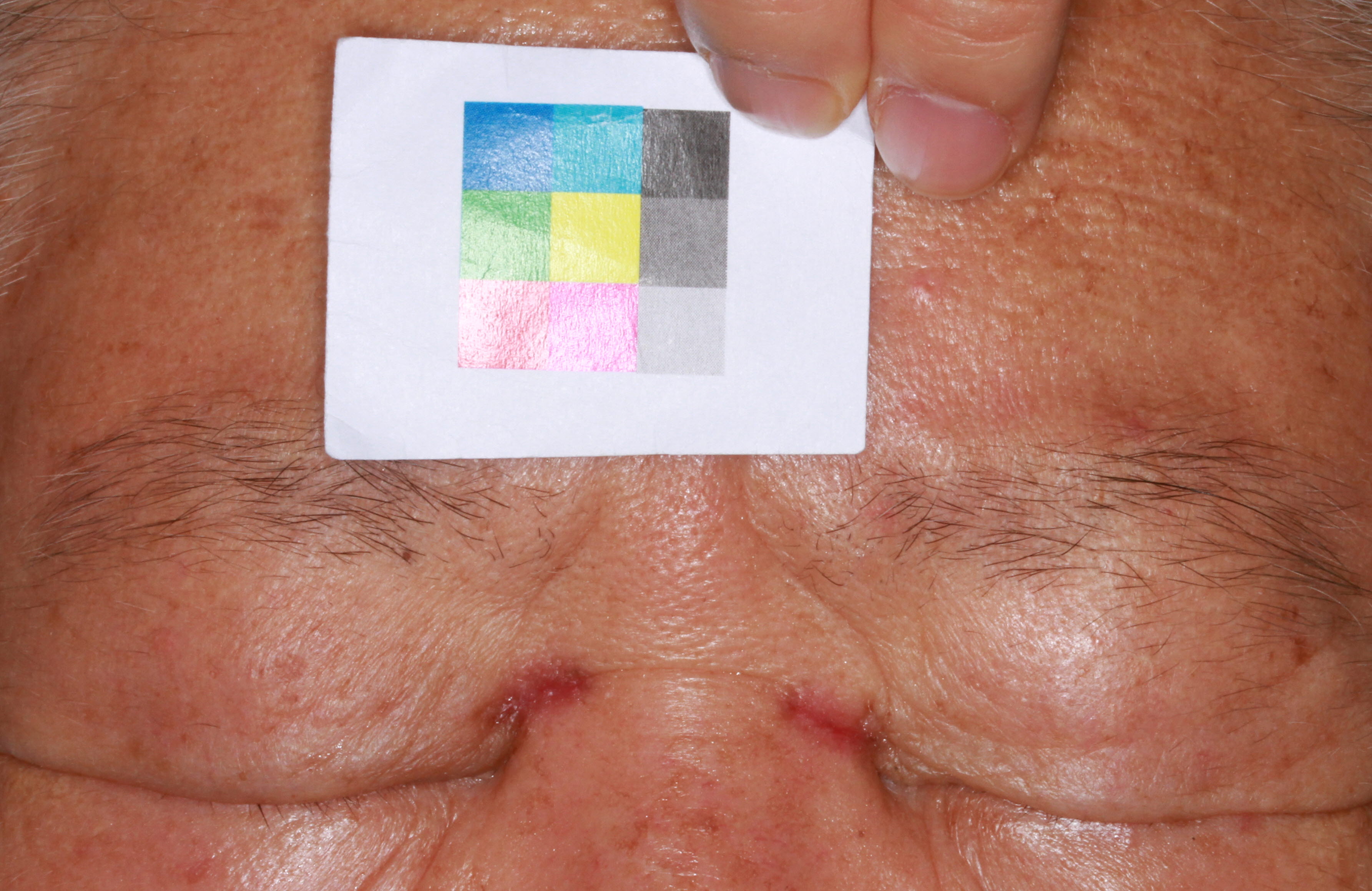

Supplement: S20 File — (ZIP) [file pone.0163092.s020.zip › 1015.jpg]

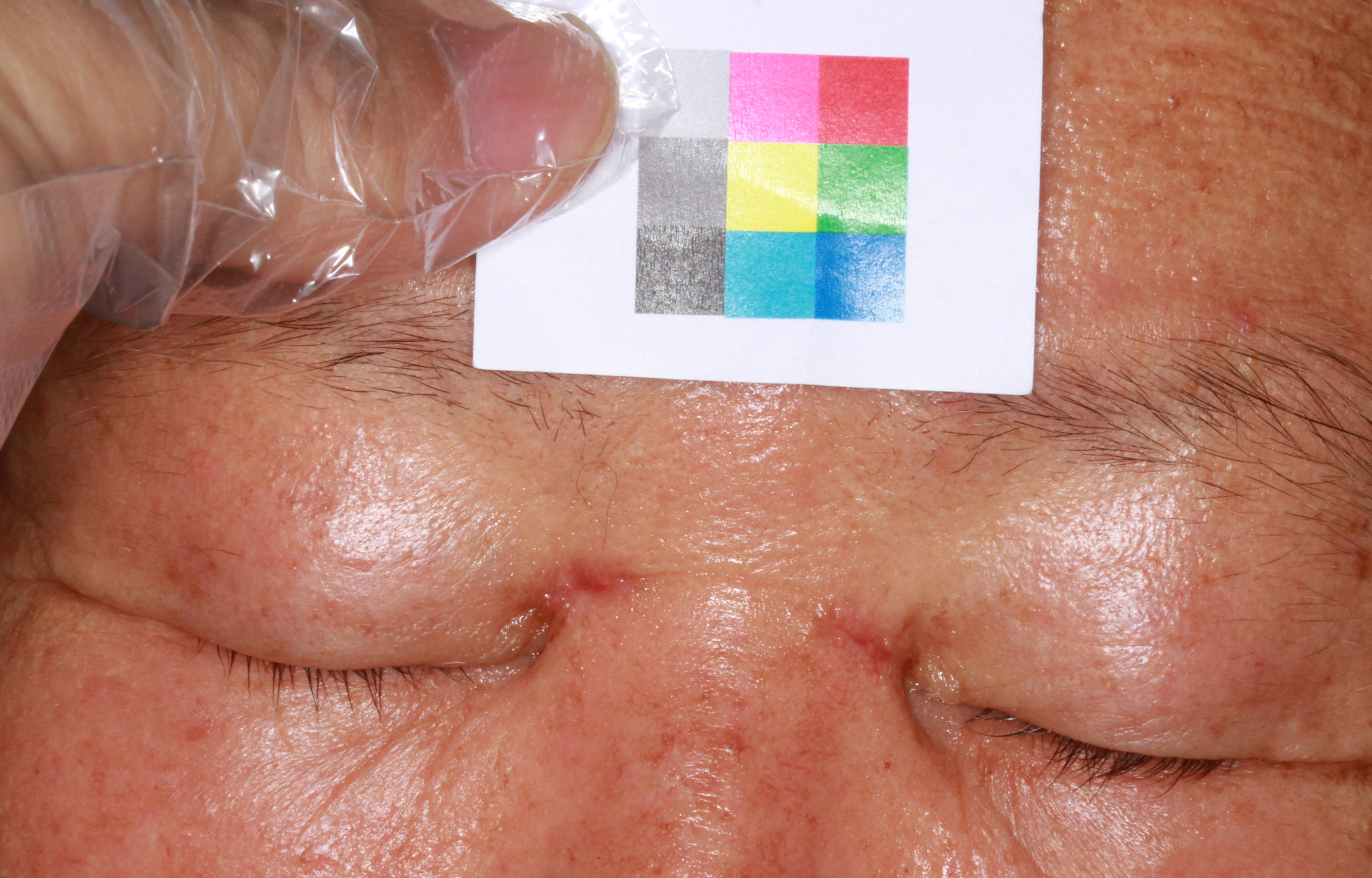

Supplement: S20 File — (ZIP) [file pone.0163092.s020.zip › 1111.jpg]

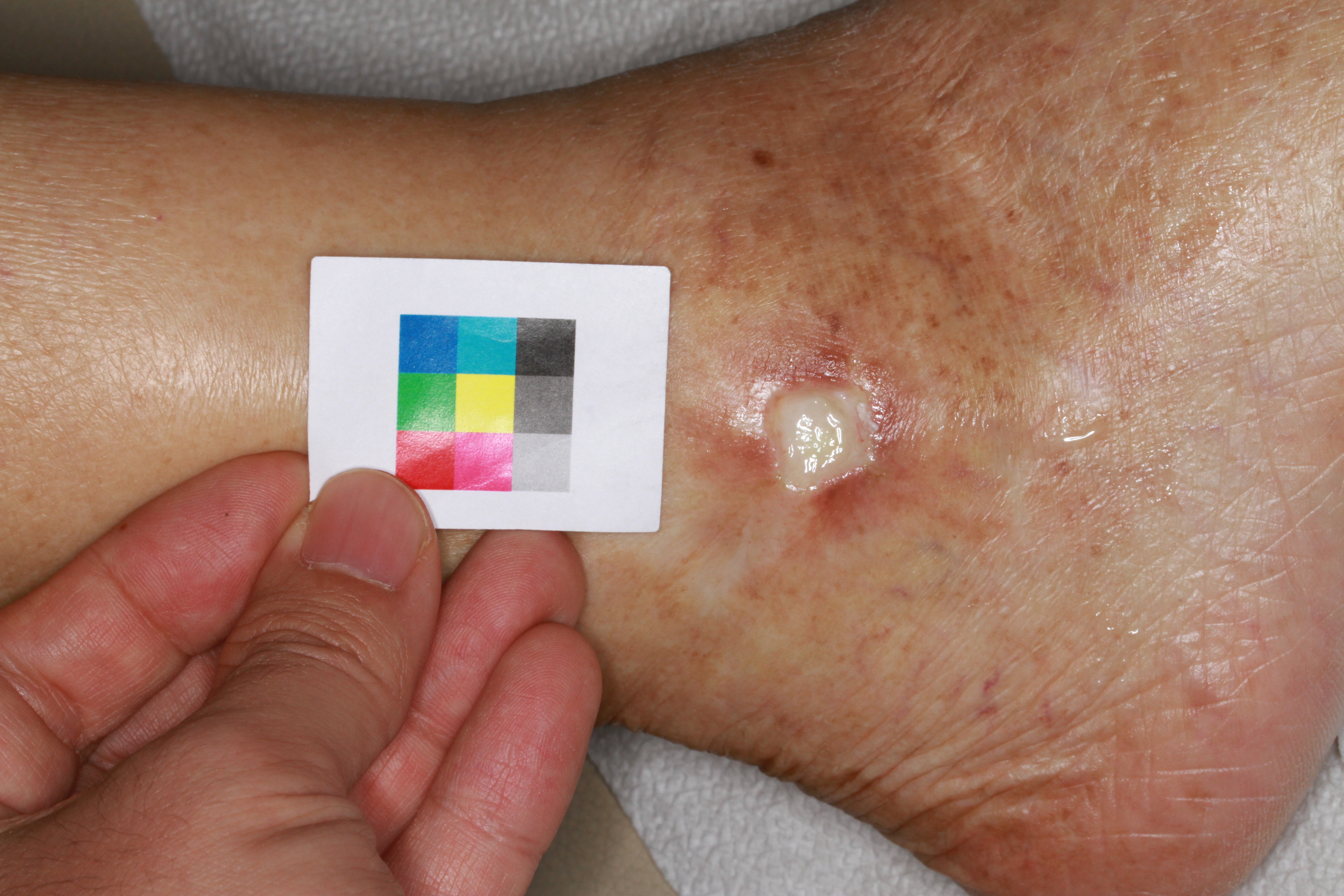

Supplement: S21 File — (ZIP) [file pone.0163092.s021.zip › 0205.JPG]

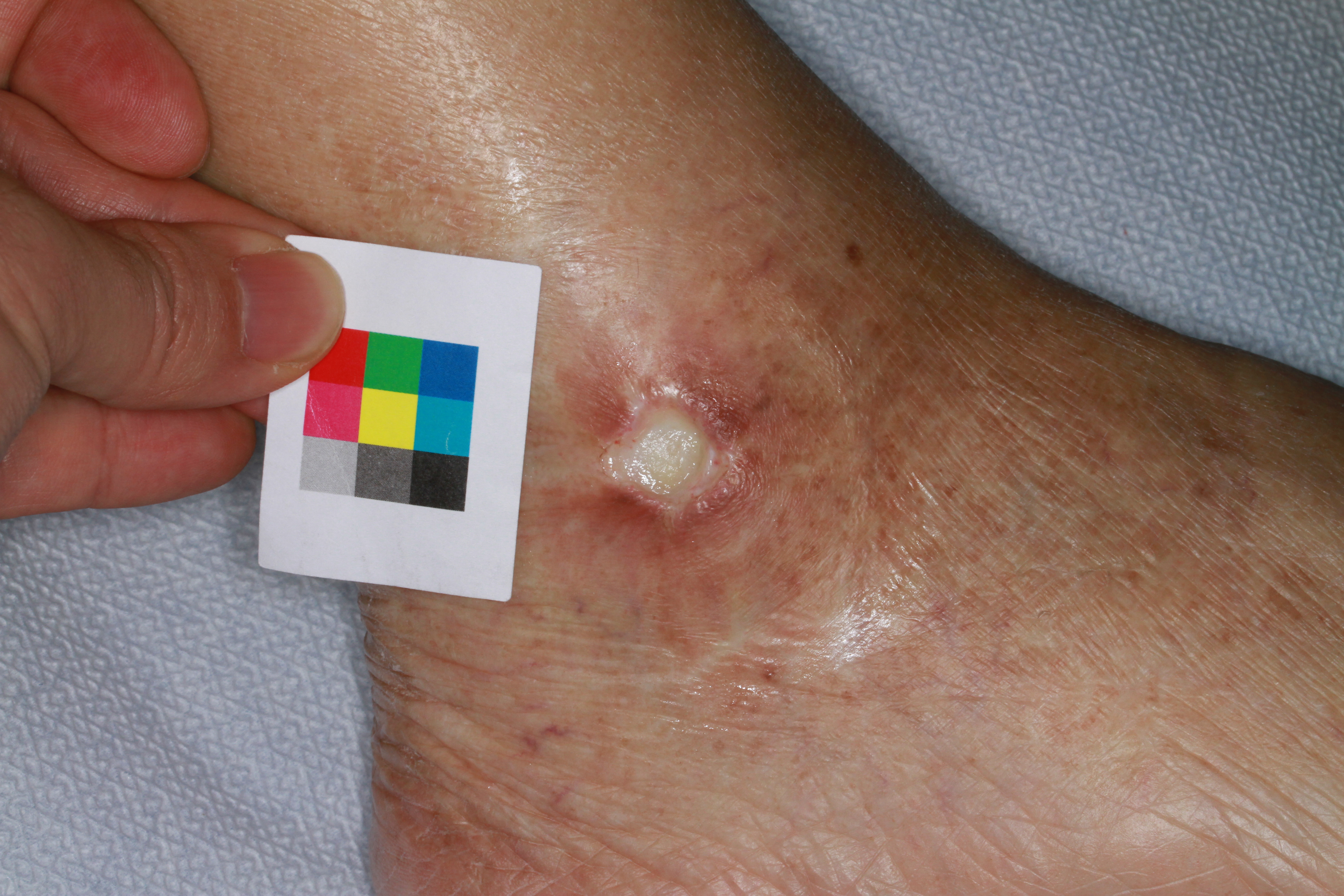

Supplement: S21 File — (ZIP) [file pone.0163092.s021.zip › 0208.JPG]

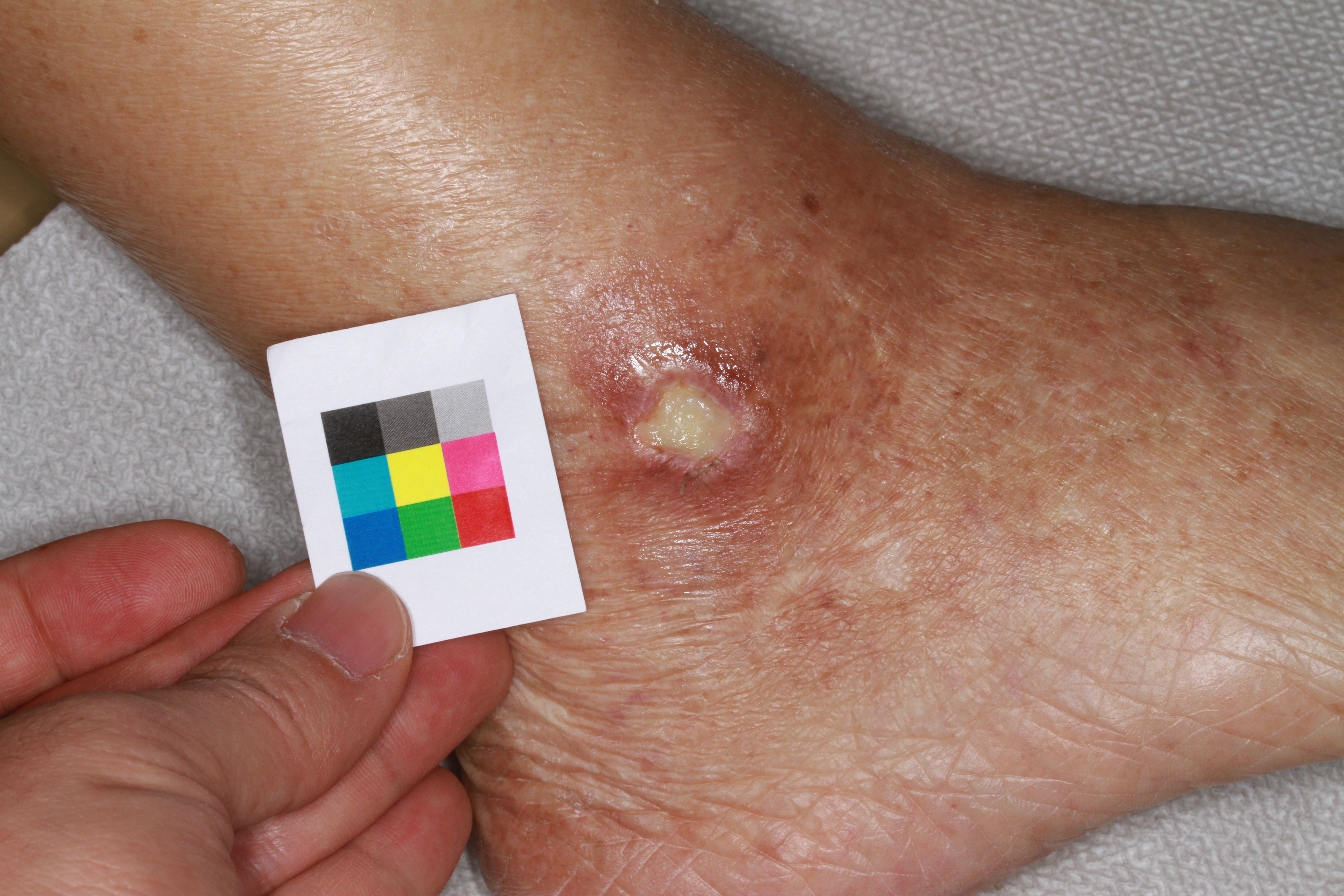

Supplement: S21 File — (ZIP) [file pone.0163092.s021.zip › 0220.JPG]

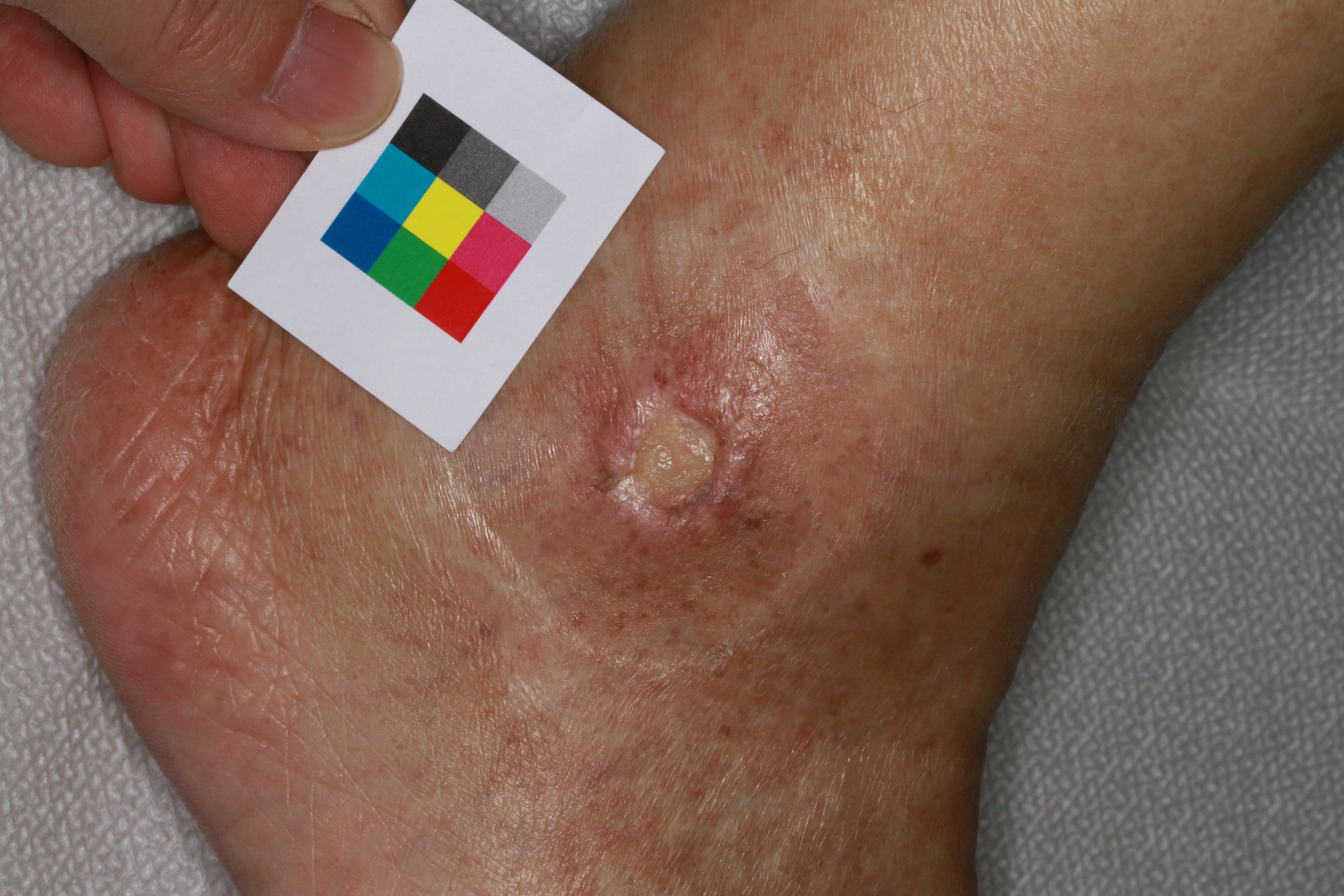

Supplement: S21 File — (ZIP) [file pone.0163092.s021.zip › 0321.JPG]

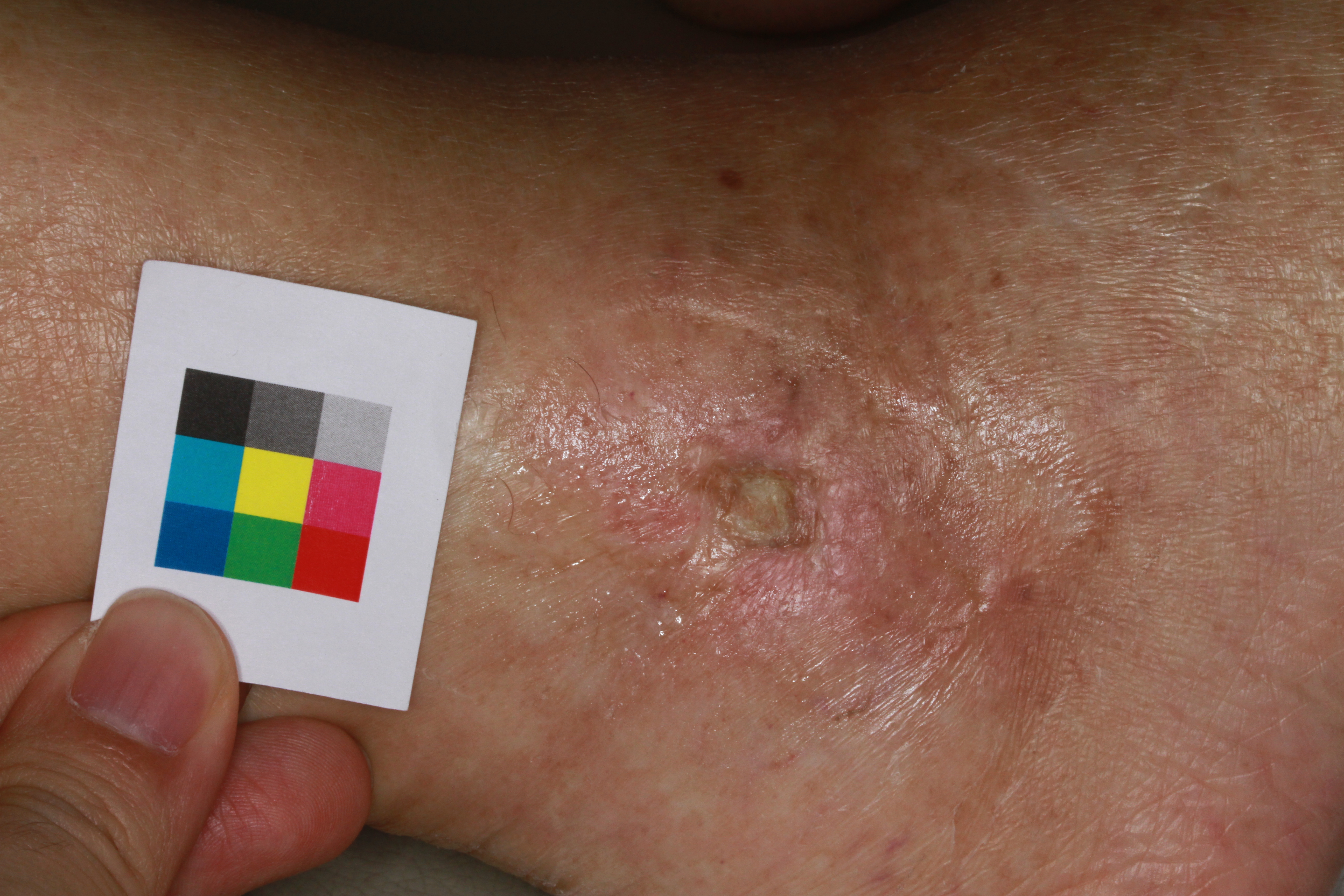

Supplement: S21 File — (ZIP) [file pone.0163092.s021.zip › 0523.JPG]

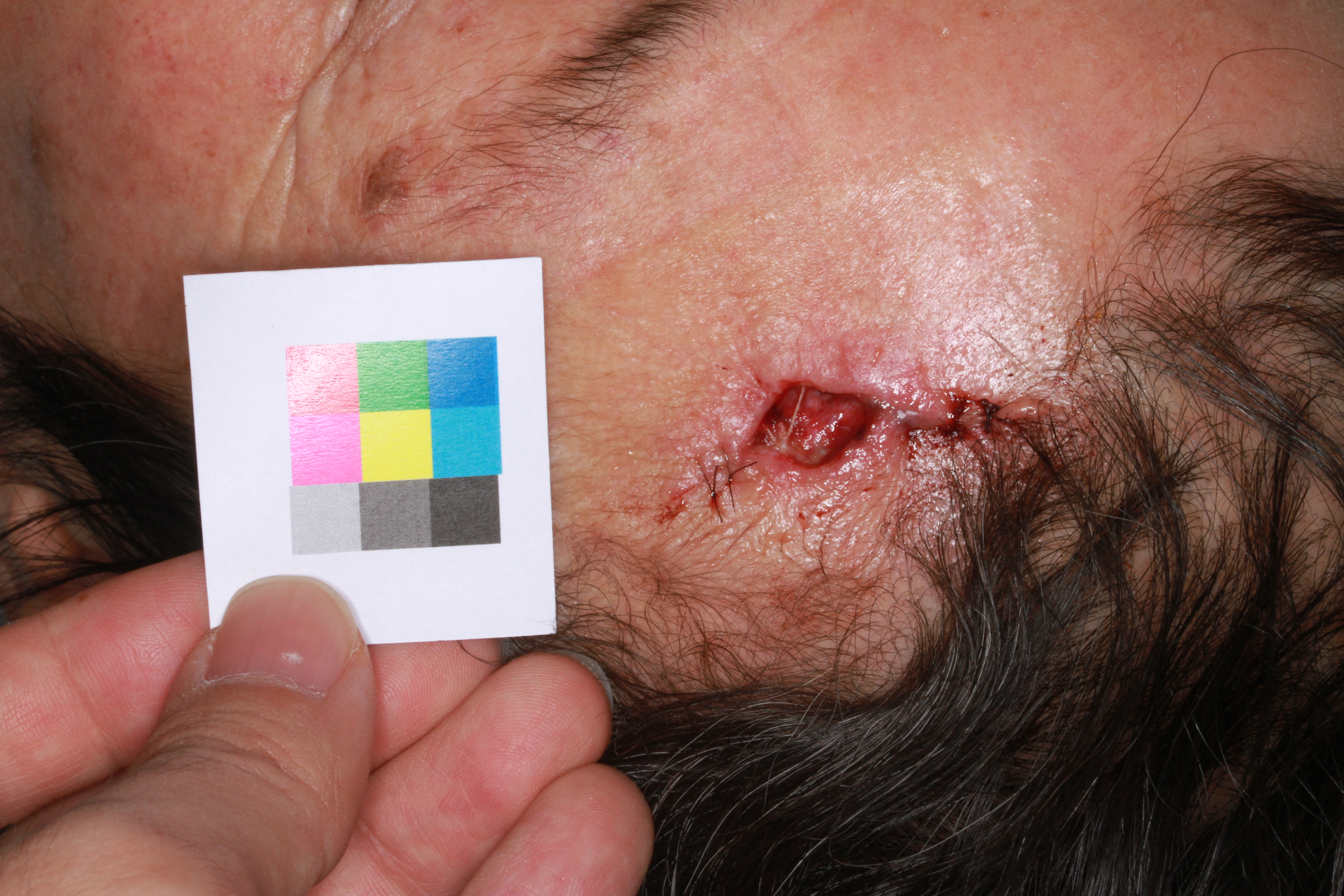

Supplement: S22 File — (ZIP) [file pone.0163092.s022.zip › 0716.JPG]

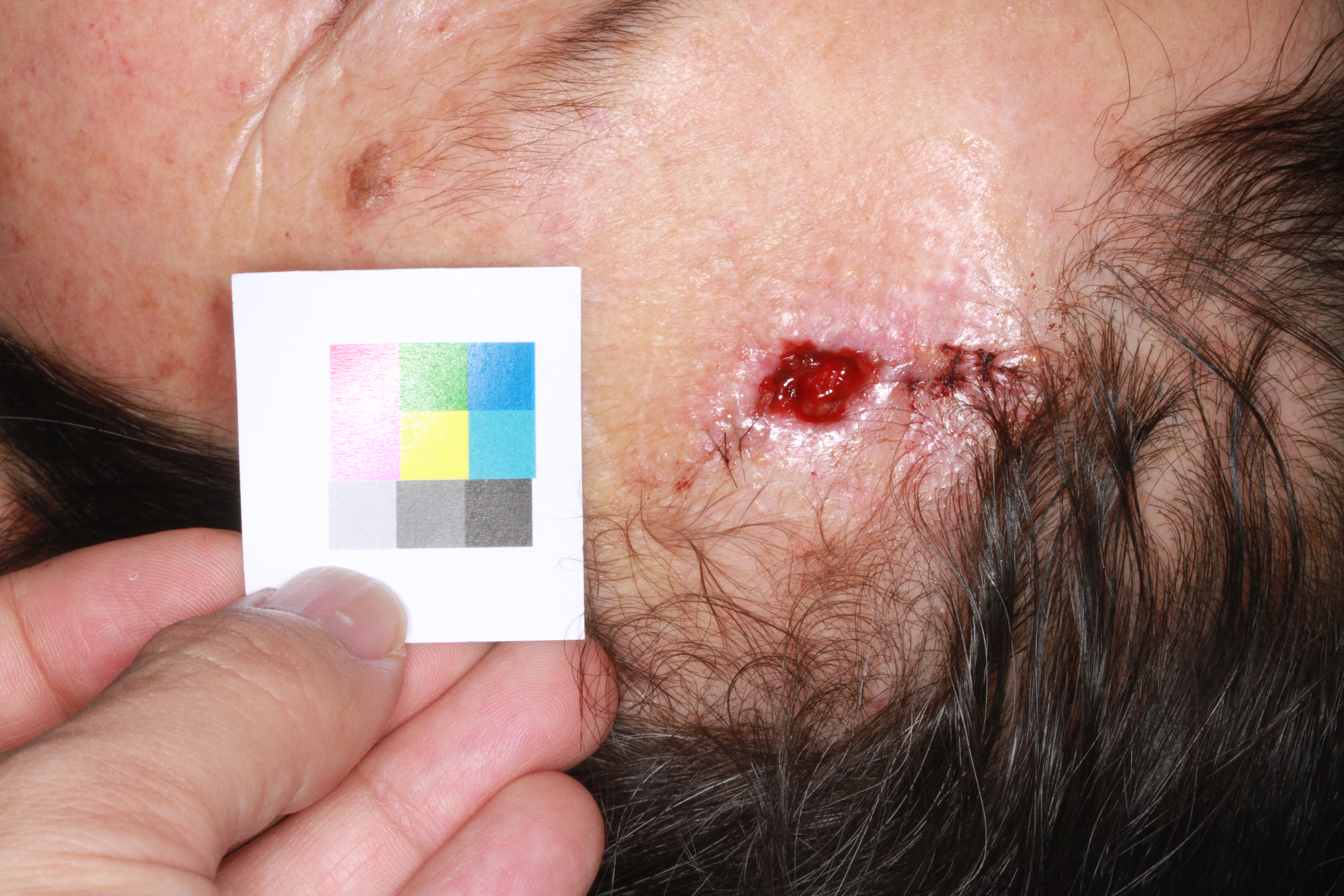

Supplement: S22 File — (ZIP) [file pone.0163092.s022.zip › 0718.JPG]

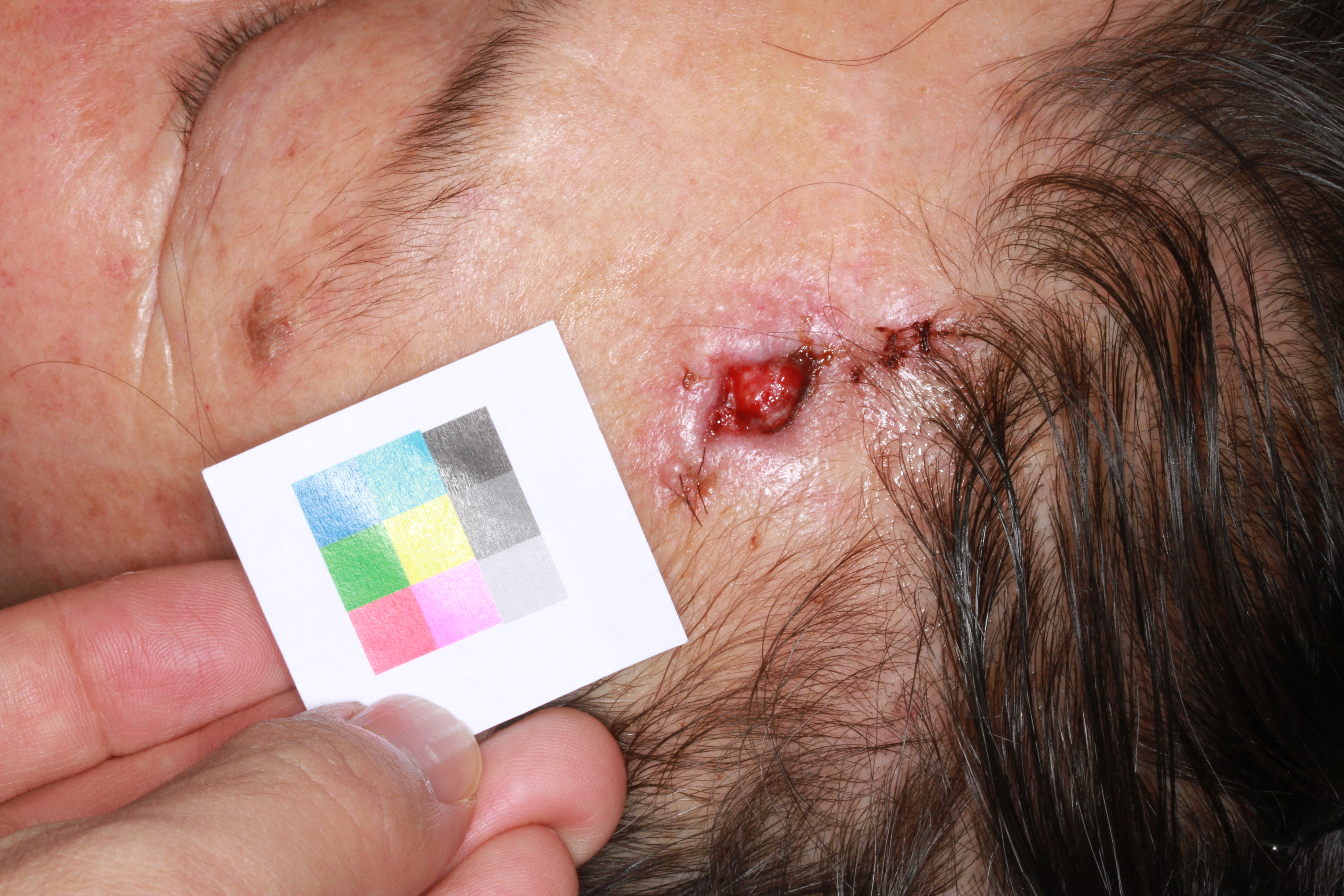

Supplement: S22 File — (ZIP) [file pone.0163092.s022.zip › 0722.JPG]

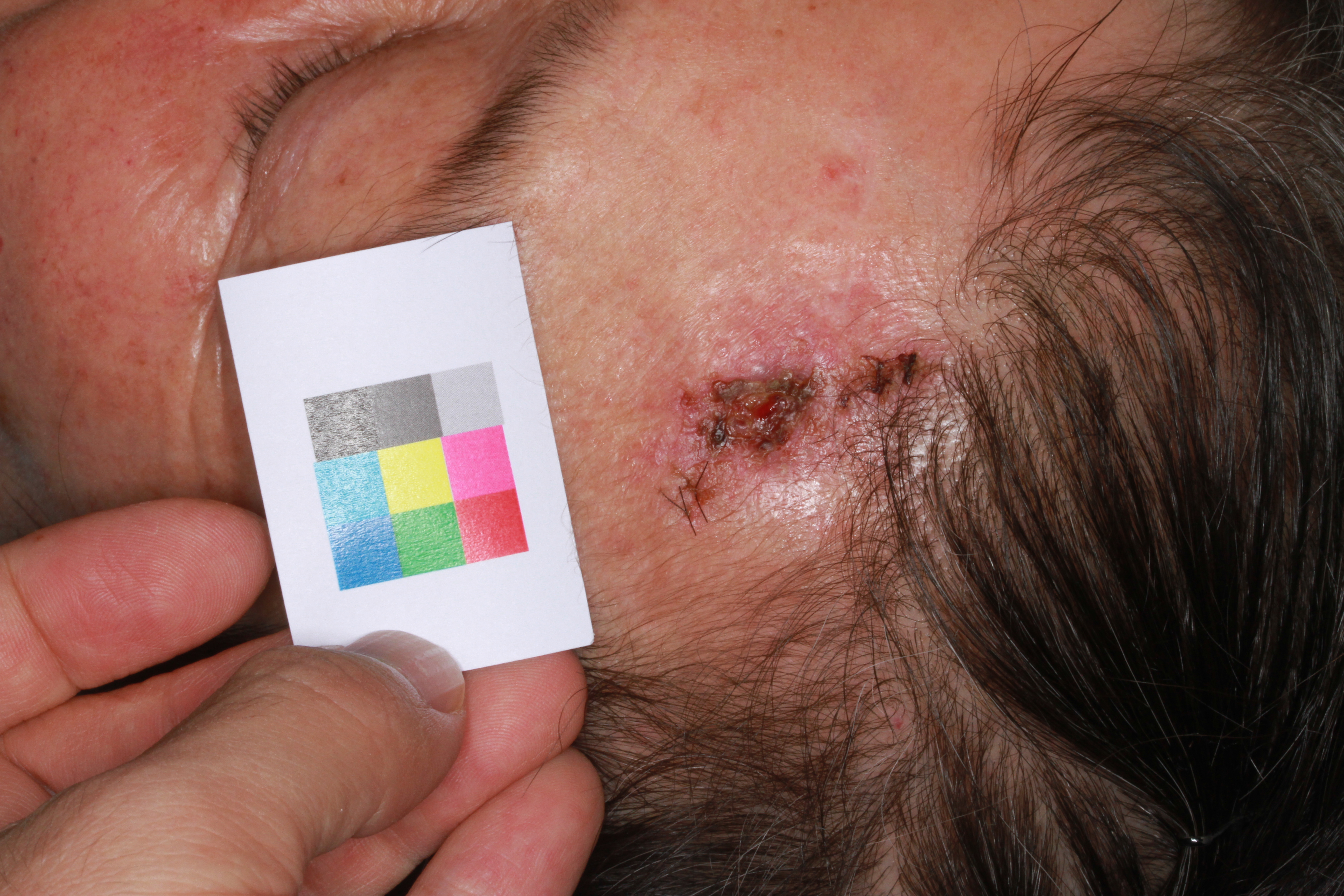

Supplement: S22 File — (ZIP) [file pone.0163092.s022.zip › 0729.JPG]

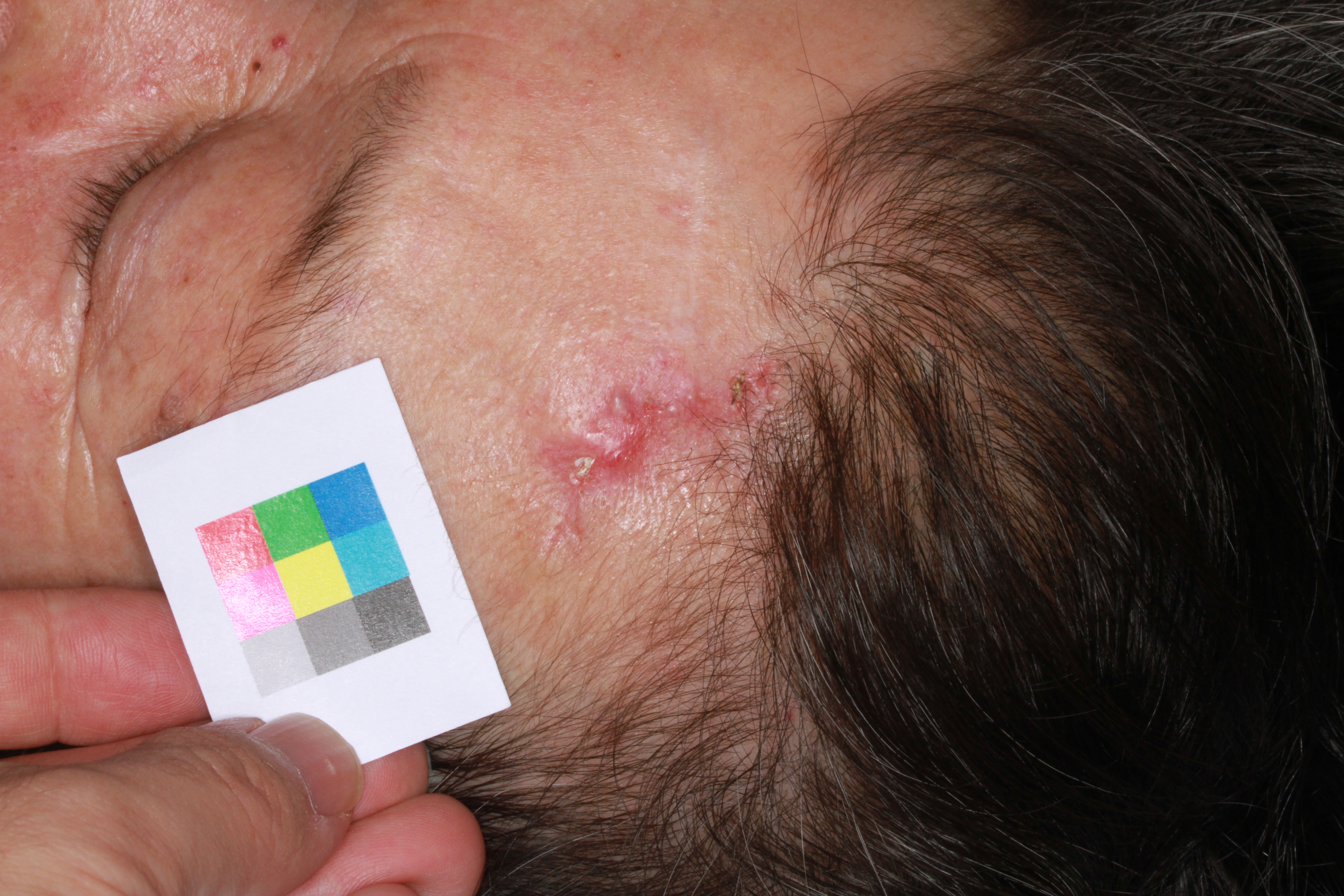

Supplement: S22 File — (ZIP) [file pone.0163092.s022.zip › 0819.JPG]

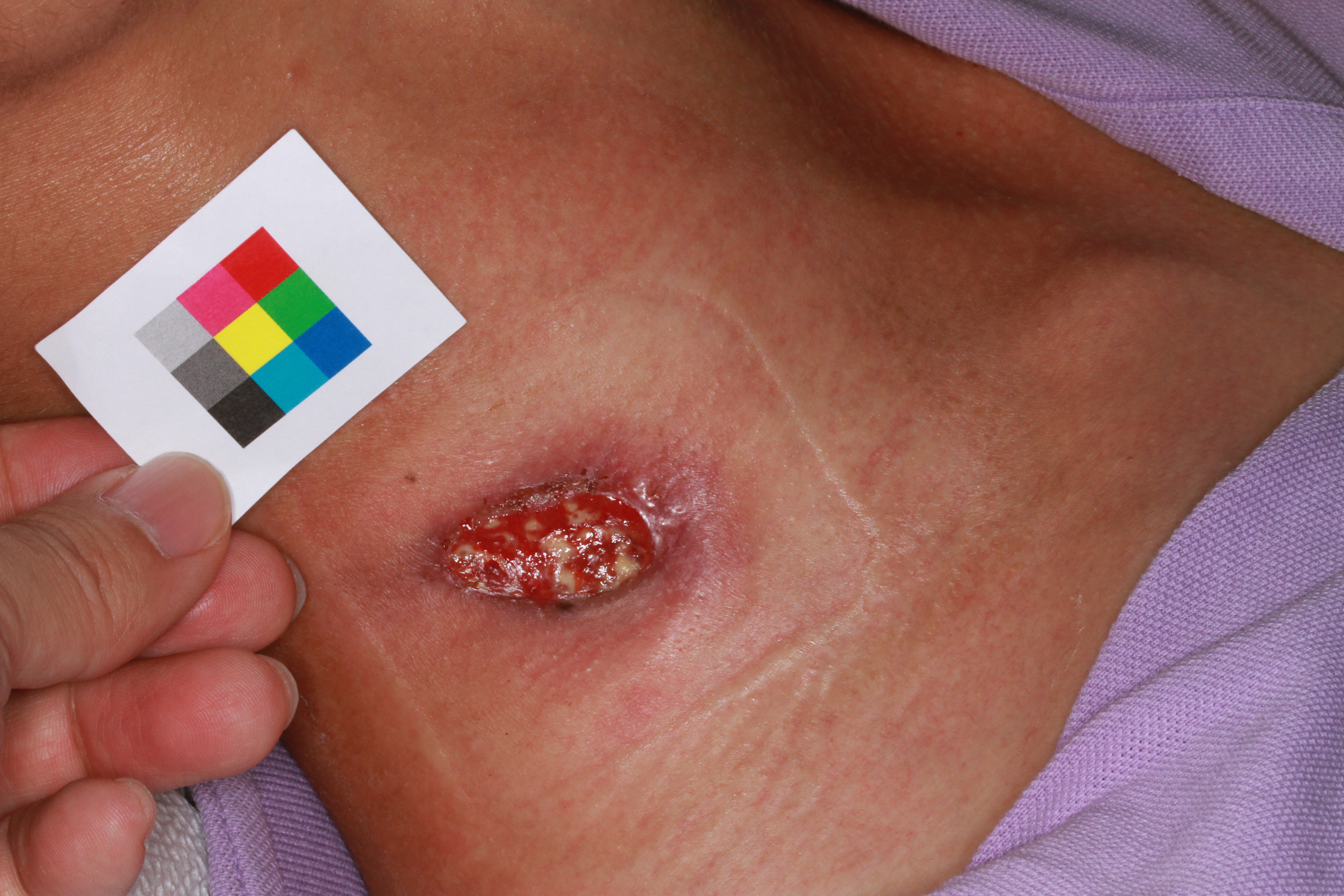

Supplement: S23 File — (ZIP) [file pone.0163092.s023.zip › 0620.JPG]

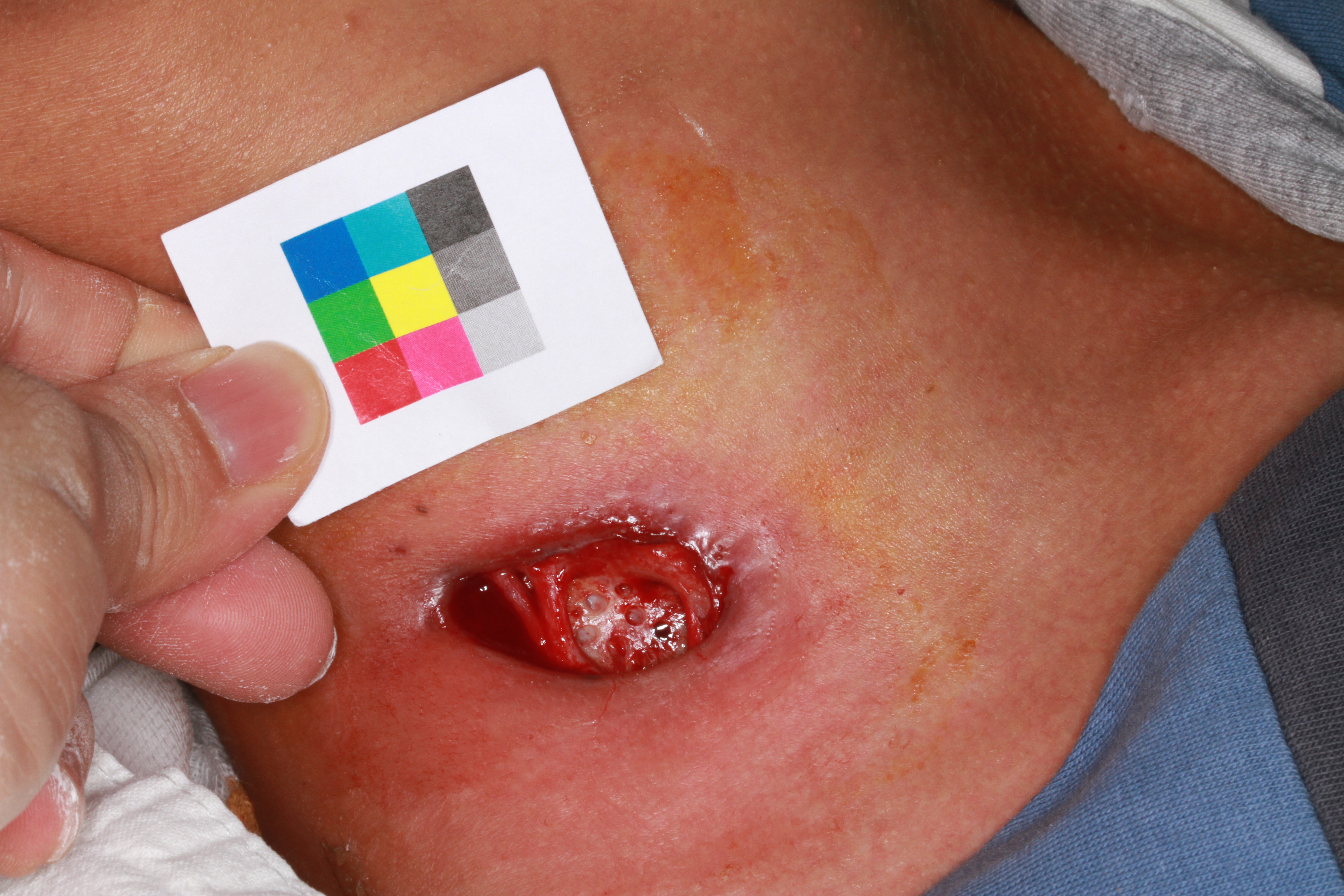

Supplement: S23 File — (ZIP) [file pone.0163092.s023.zip › 0624.JPG]

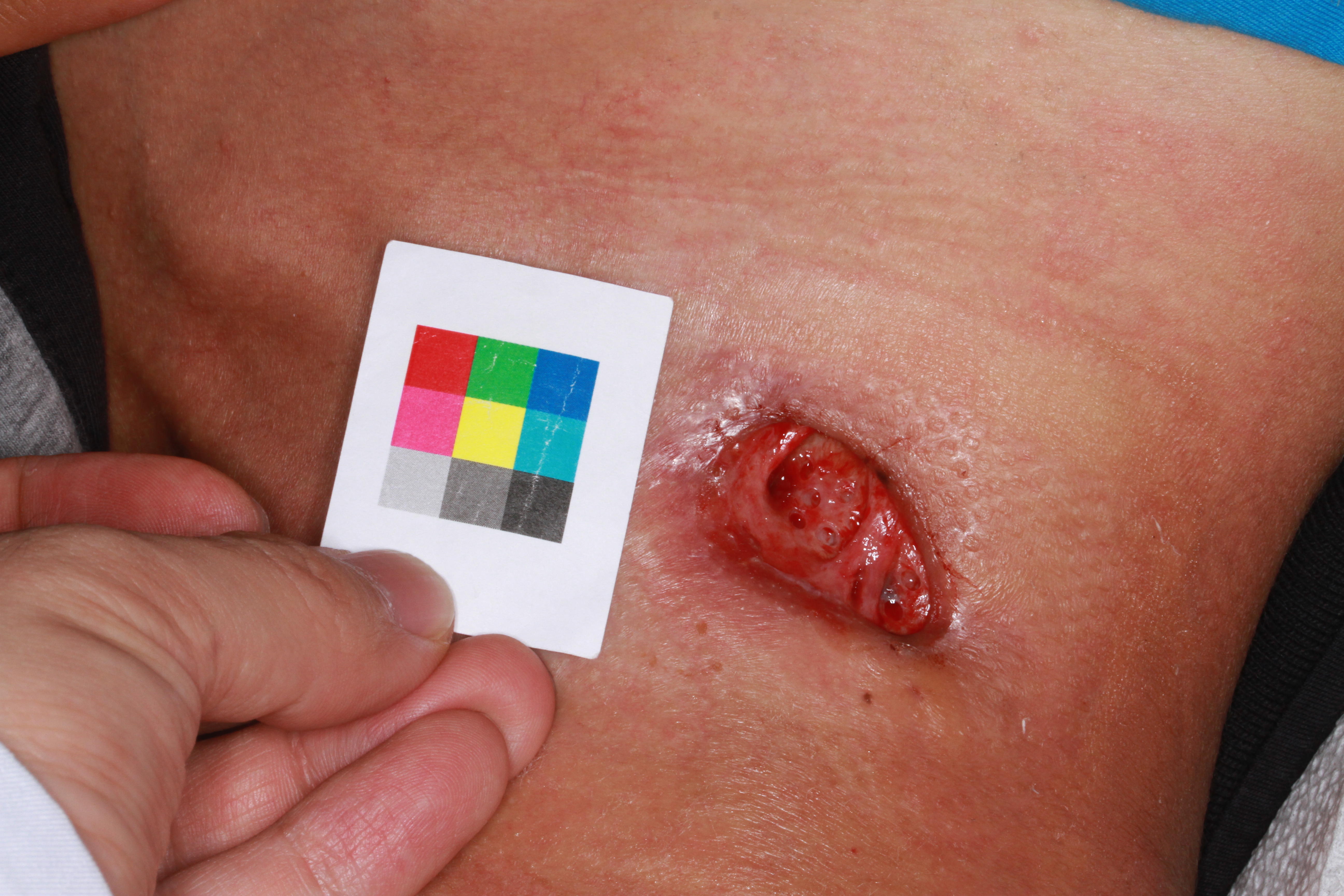

Supplement: S23 File — (ZIP) [file pone.0163092.s023.zip › 0625.JPG]

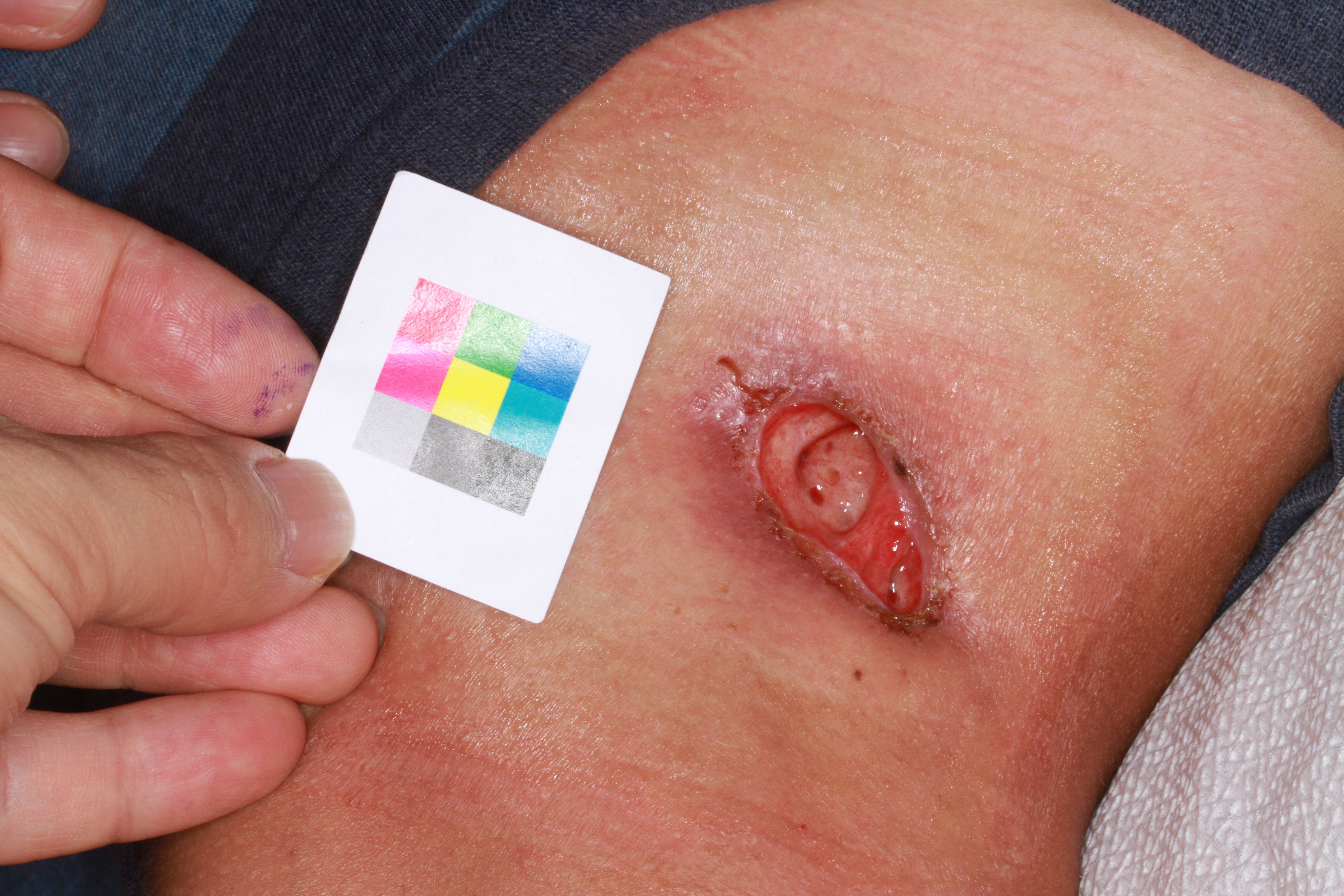

Supplement: S23 File — (ZIP) [file pone.0163092.s023.zip › 0701.JPG]

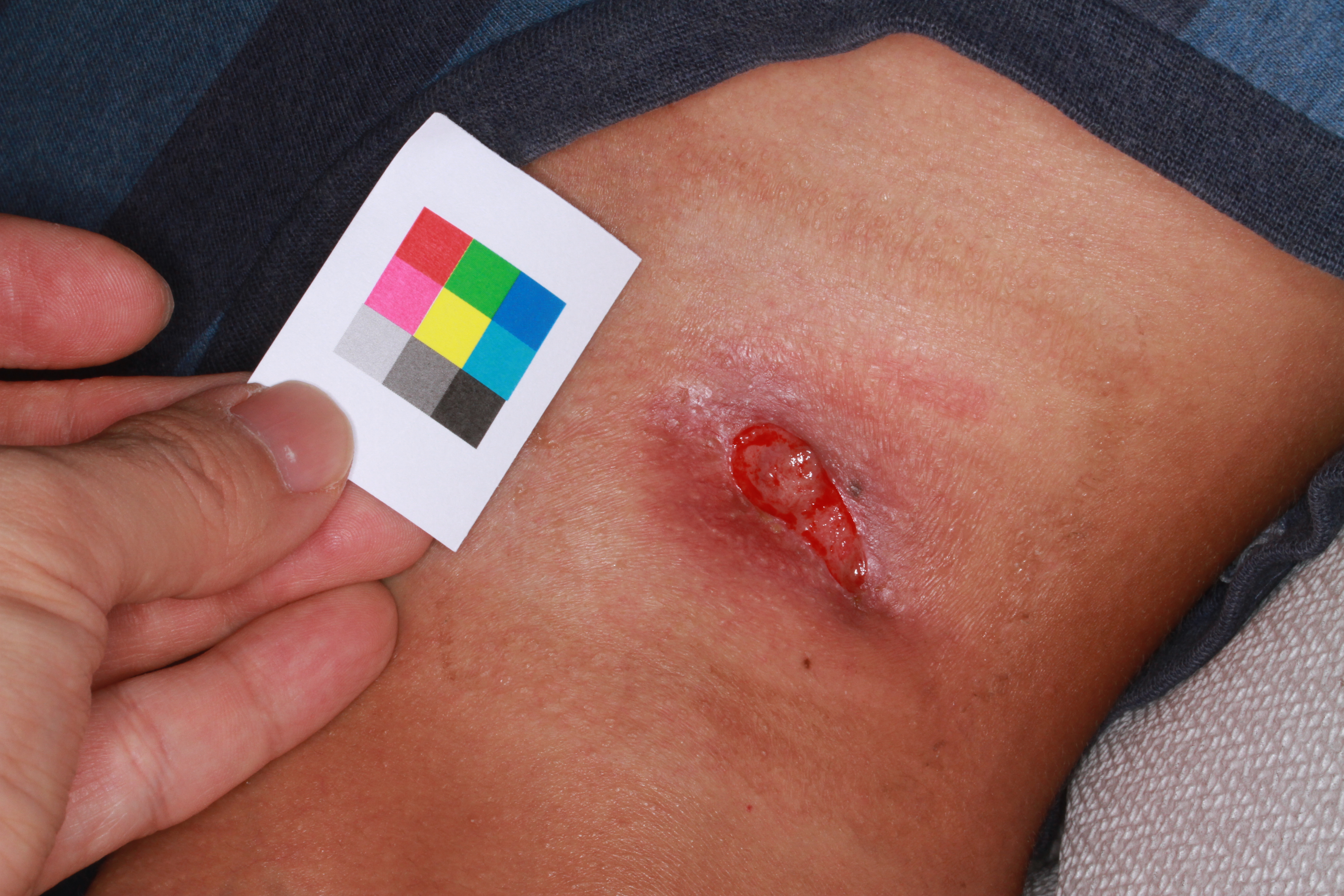

Supplement: S23 File — (ZIP) [file pone.0163092.s023.zip › 0709.JPG]

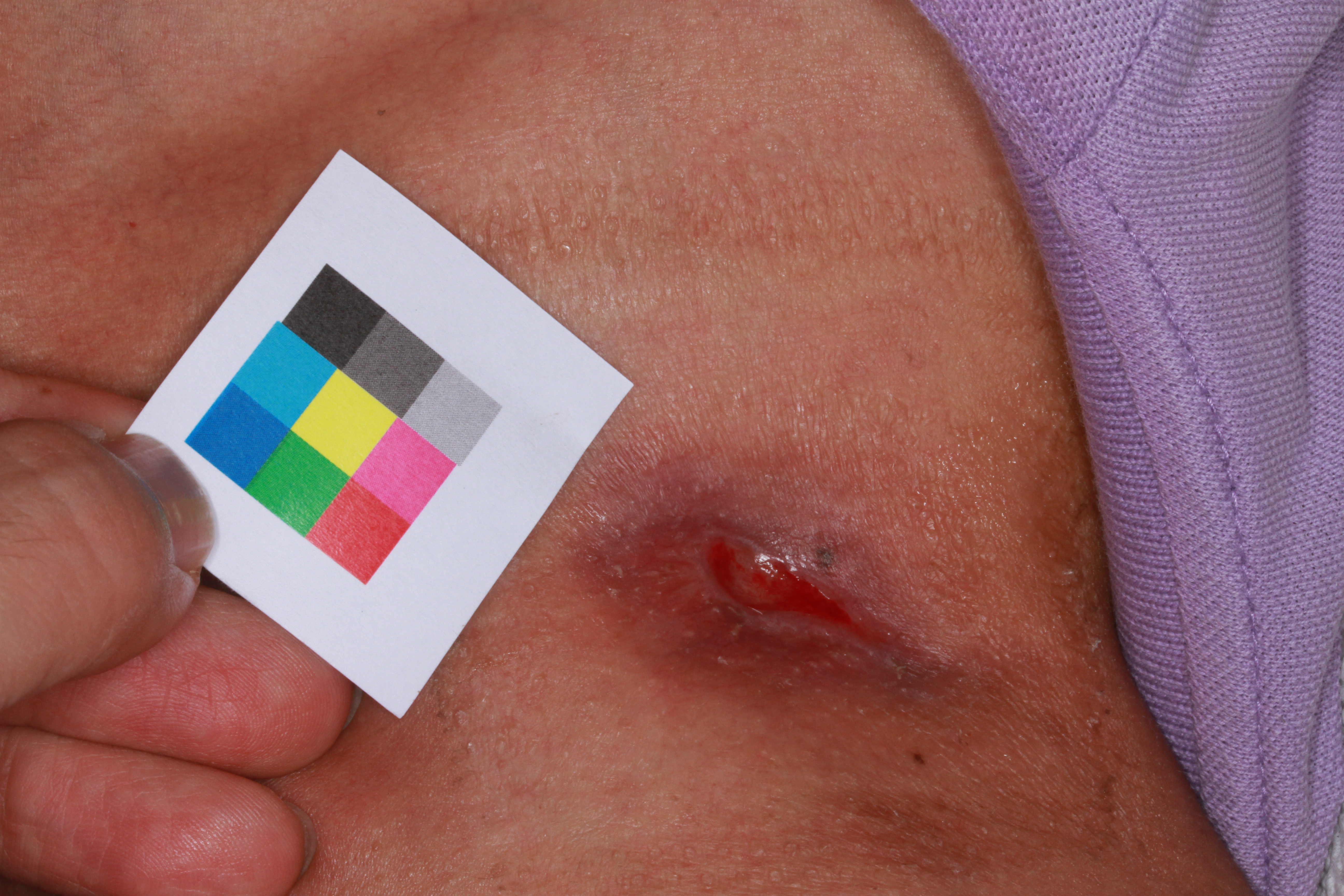

Supplement: S23 File — (ZIP) [file pone.0163092.s023.zip › 0715.JPG]

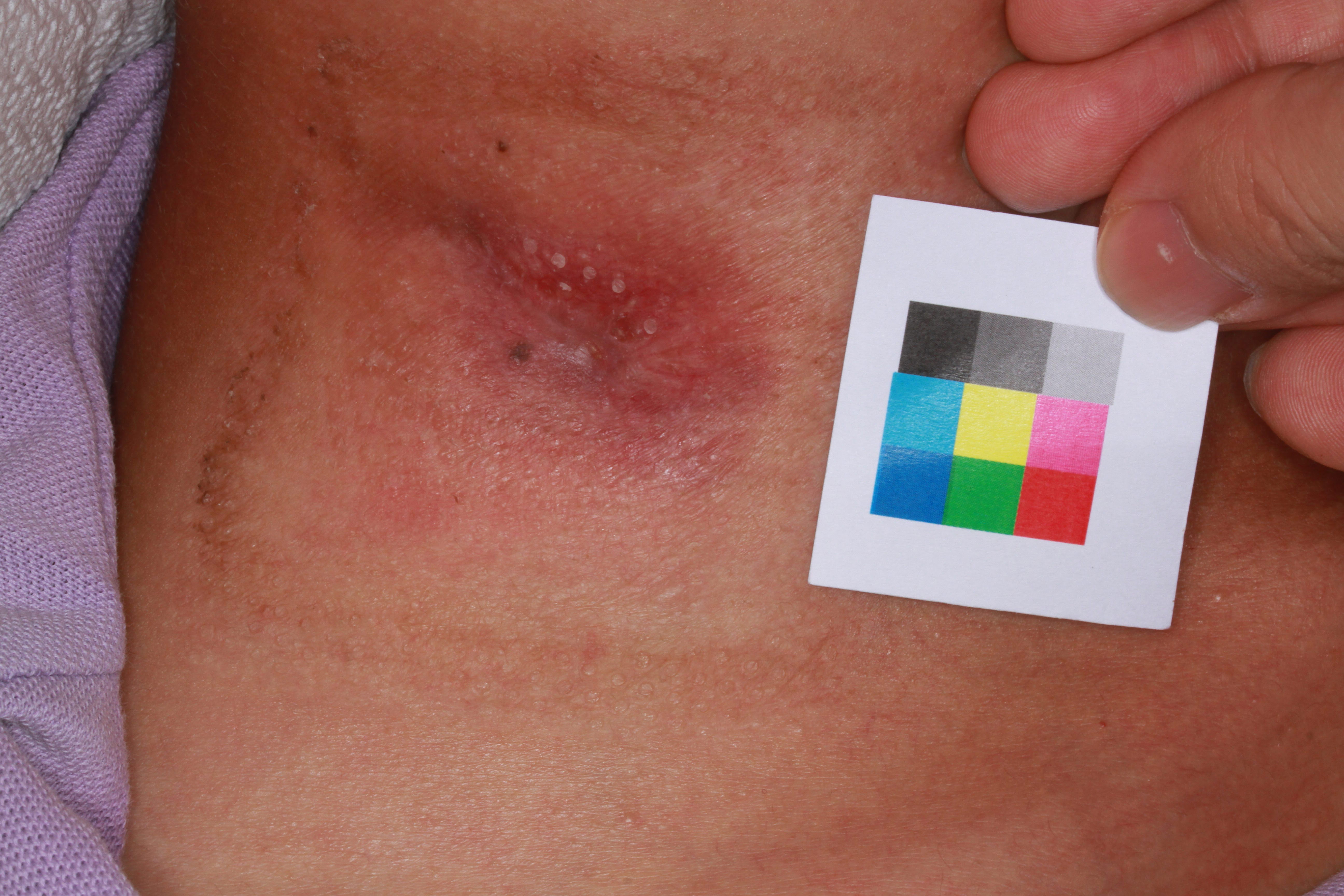

Supplement: S23 File — (ZIP) [file pone.0163092.s023.zip › 0722.JPG]

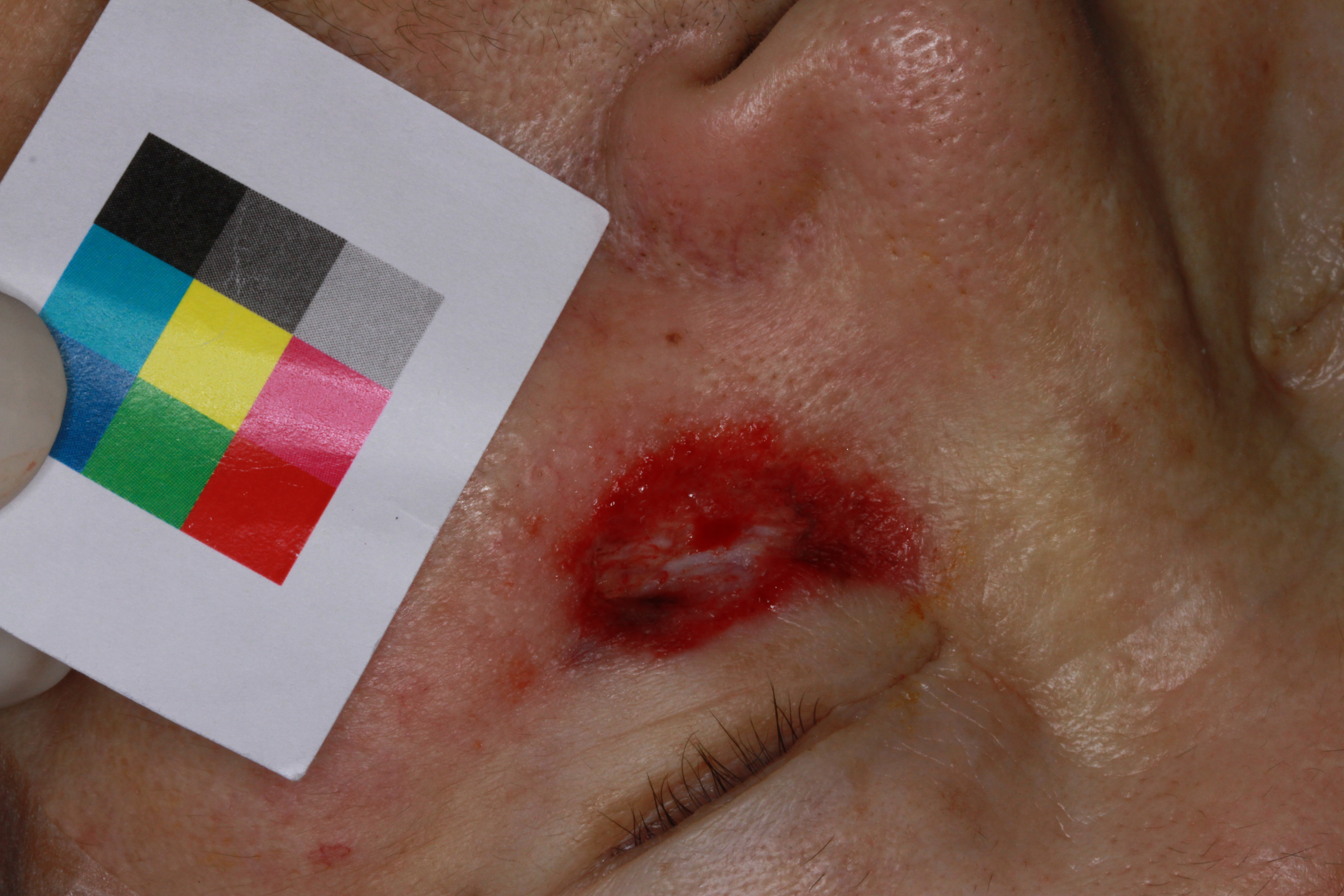

Supplement: S24 File — (ZIP) [file pone.0163092.s024.zip › 0624.JPG]

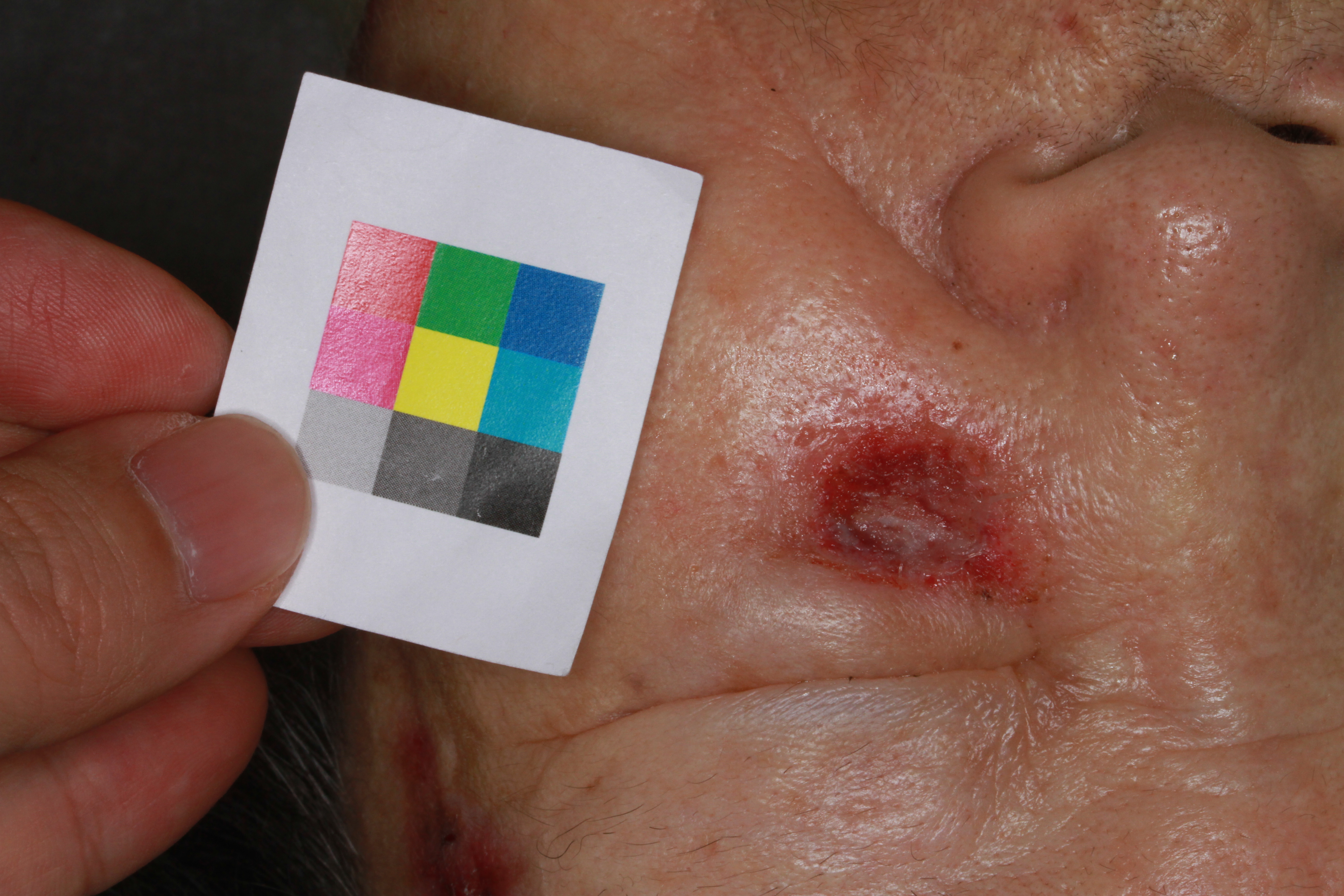

Supplement: S24 File — (ZIP) [file pone.0163092.s024.zip › 0625.JPG]

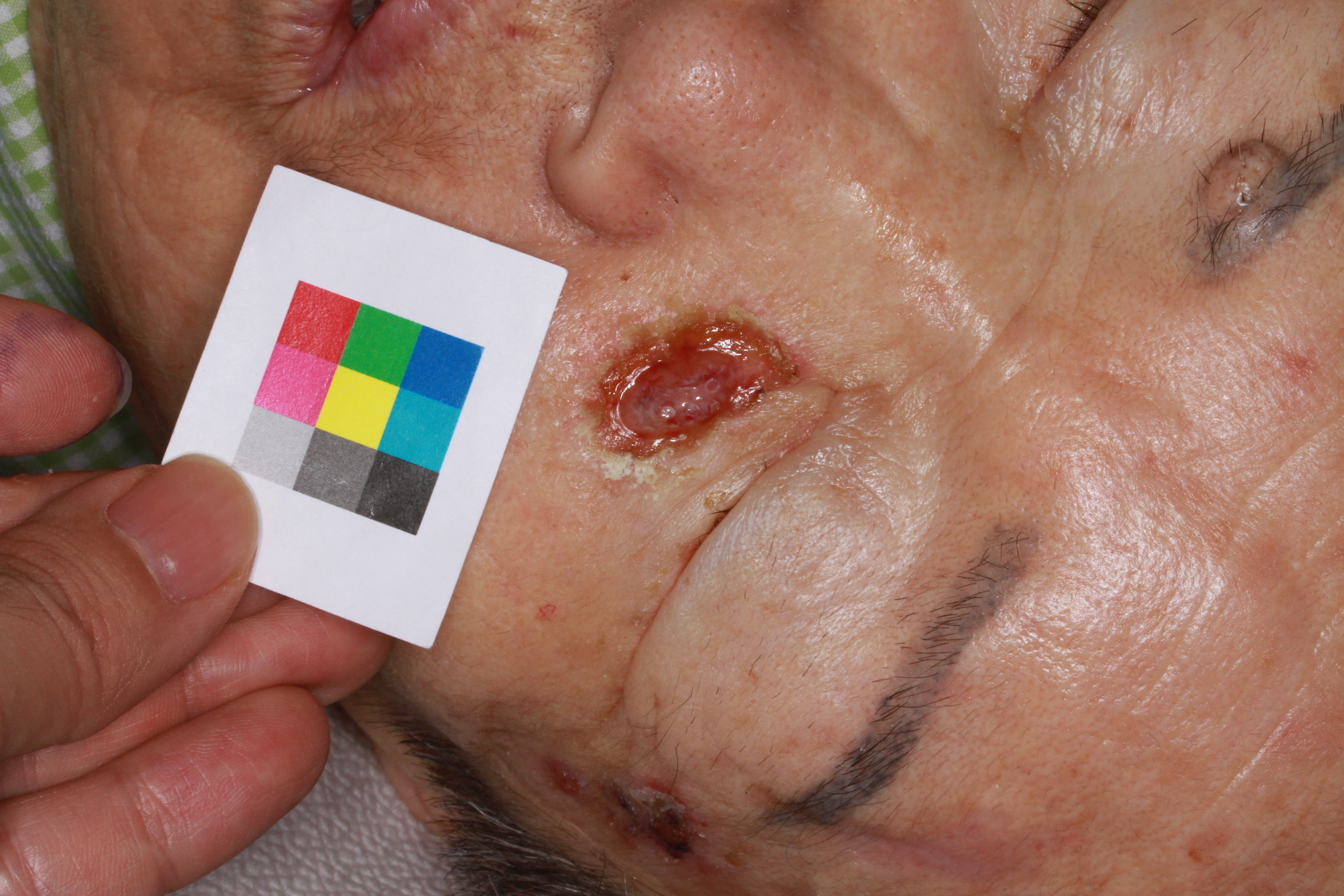

Supplement: S24 File — (ZIP) [file pone.0163092.s024.zip › 0701.JPG]

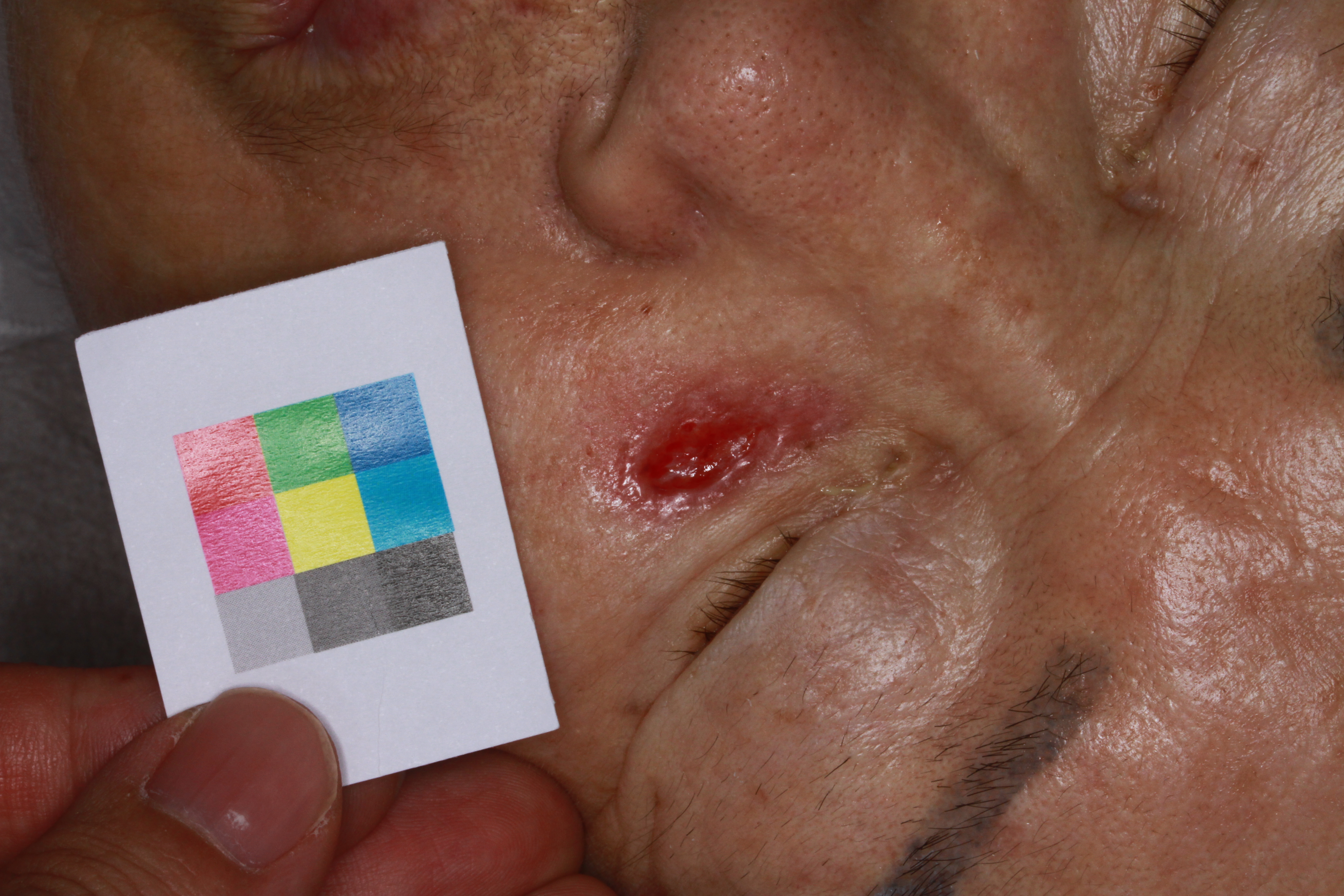

Supplement: S24 File — (ZIP) [file pone.0163092.s024.zip › 0709.JPG]

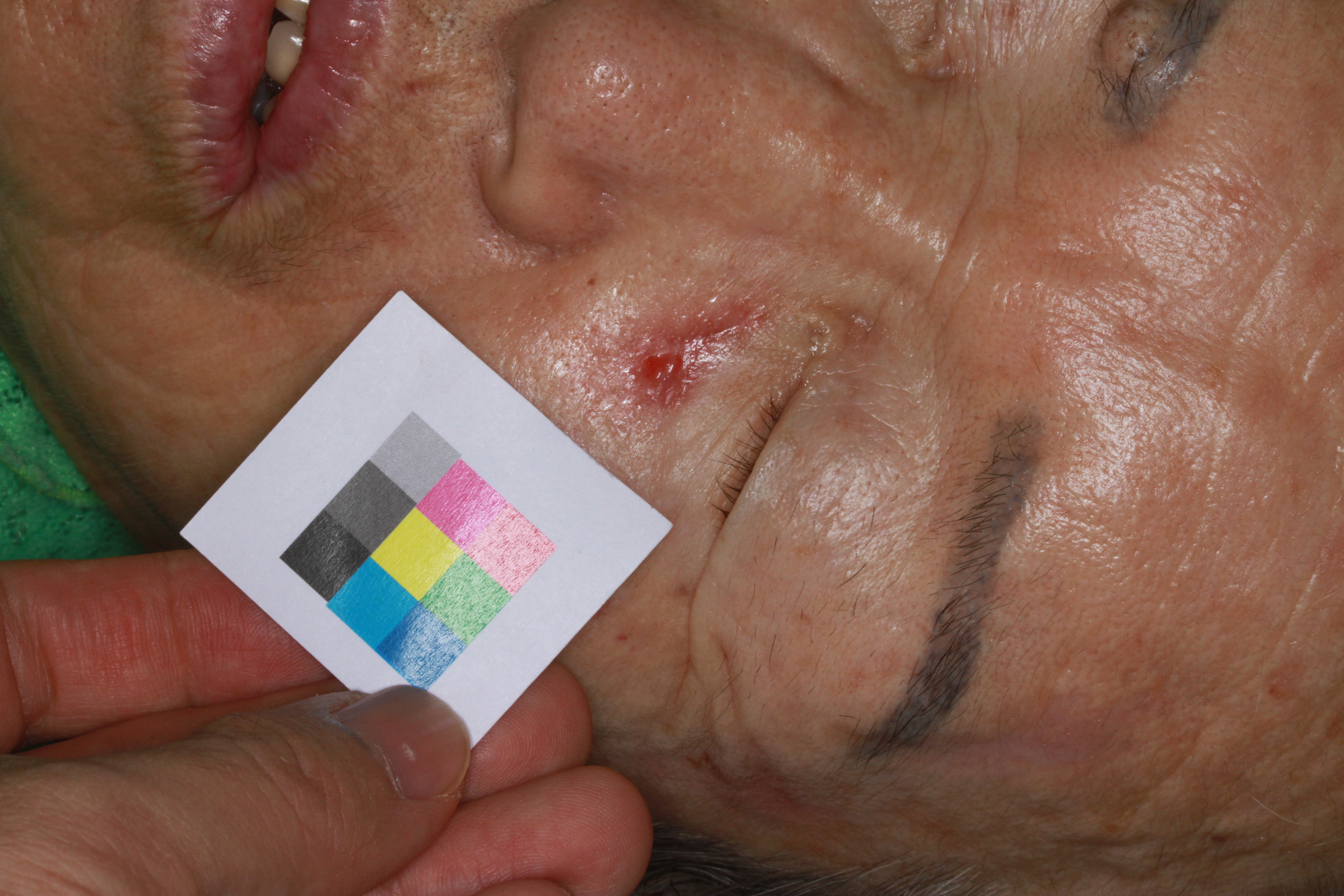

Supplement: S24 File — (ZIP) [file pone.0163092.s024.zip › 0722.JPG]

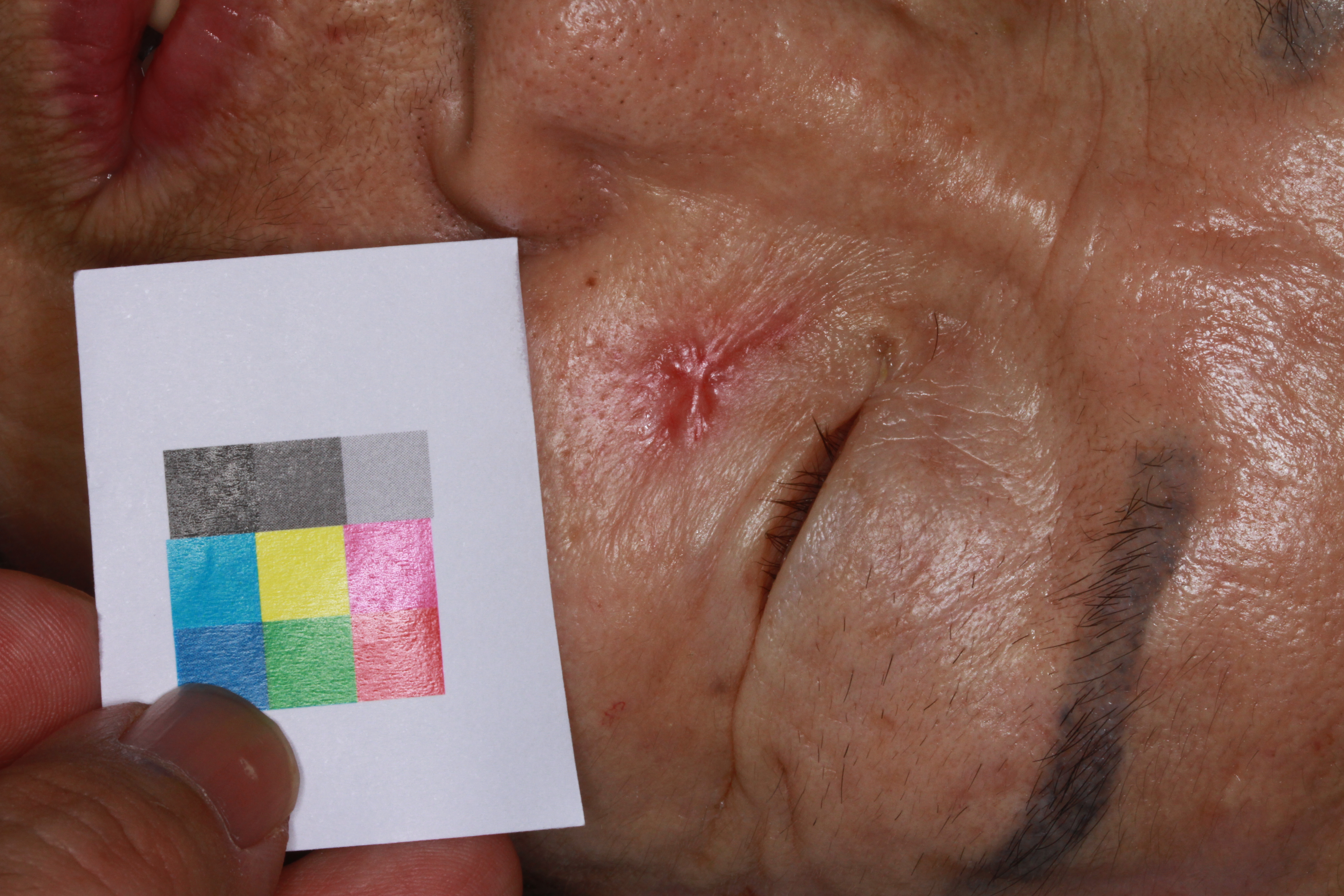

Supplement: S24 File — (ZIP) [file pone.0163092.s024.zip › 0819.JPG]

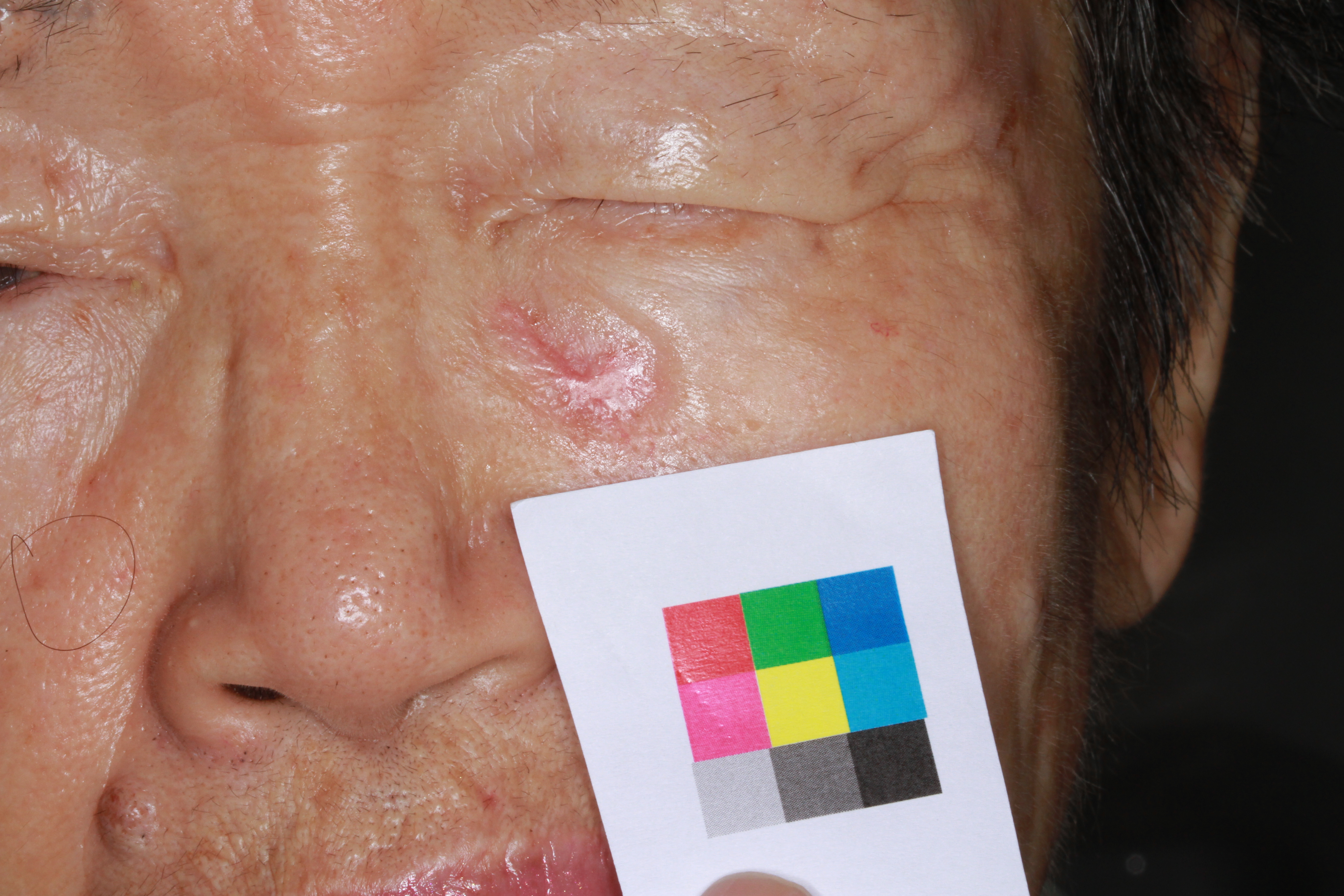

Supplement: S24 File — (ZIP) [file pone.0163092.s024.zip › 0903.JPG]

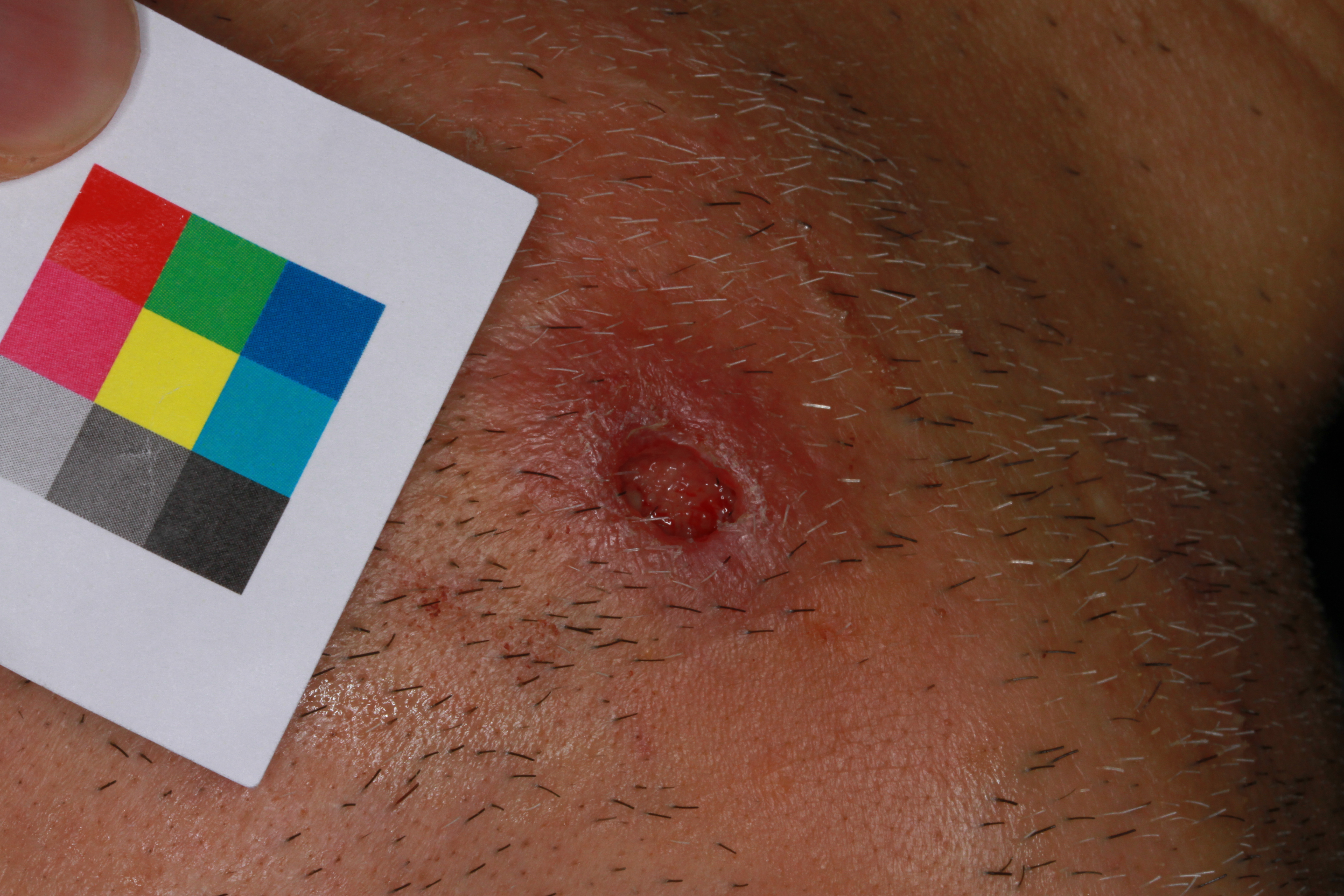

Supplement: S25 File — (ZIP) [file pone.0163092.s025.zip › 0623.JPG]

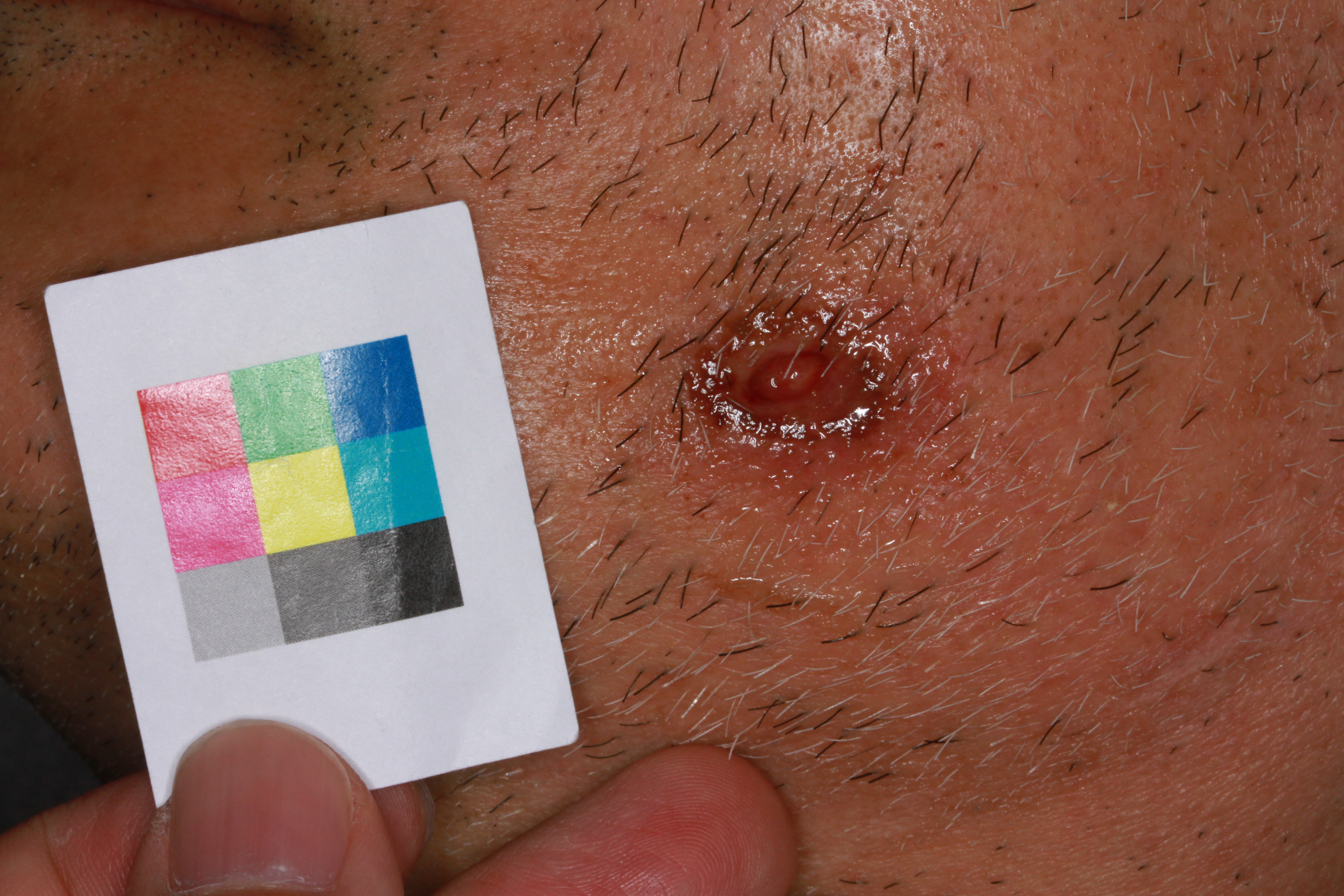

Supplement: S25 File — (ZIP) [file pone.0163092.s025.zip › 0625.JPG]

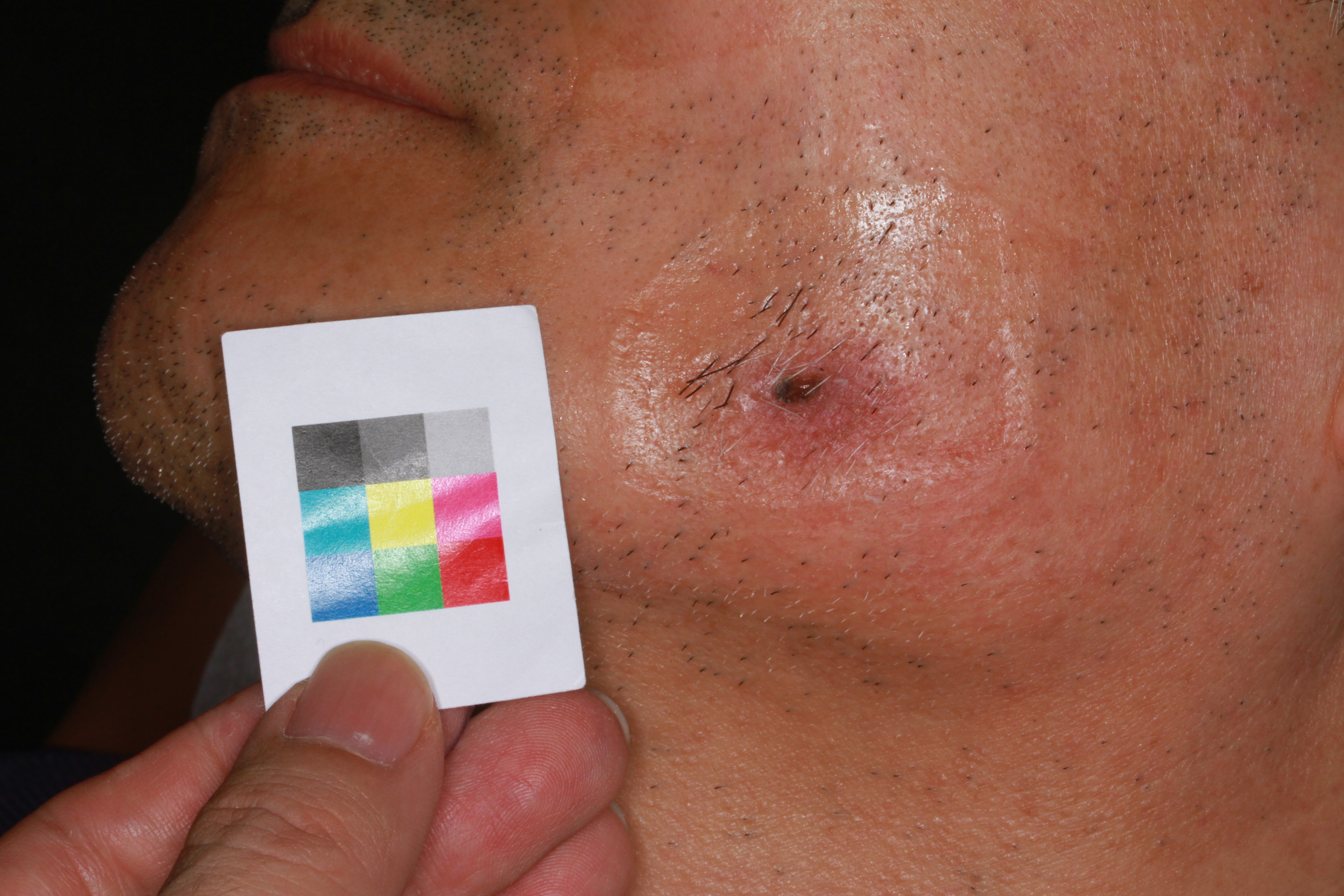

Supplement: S25 File — (ZIP) [file pone.0163092.s025.zip › 0701.JPG]

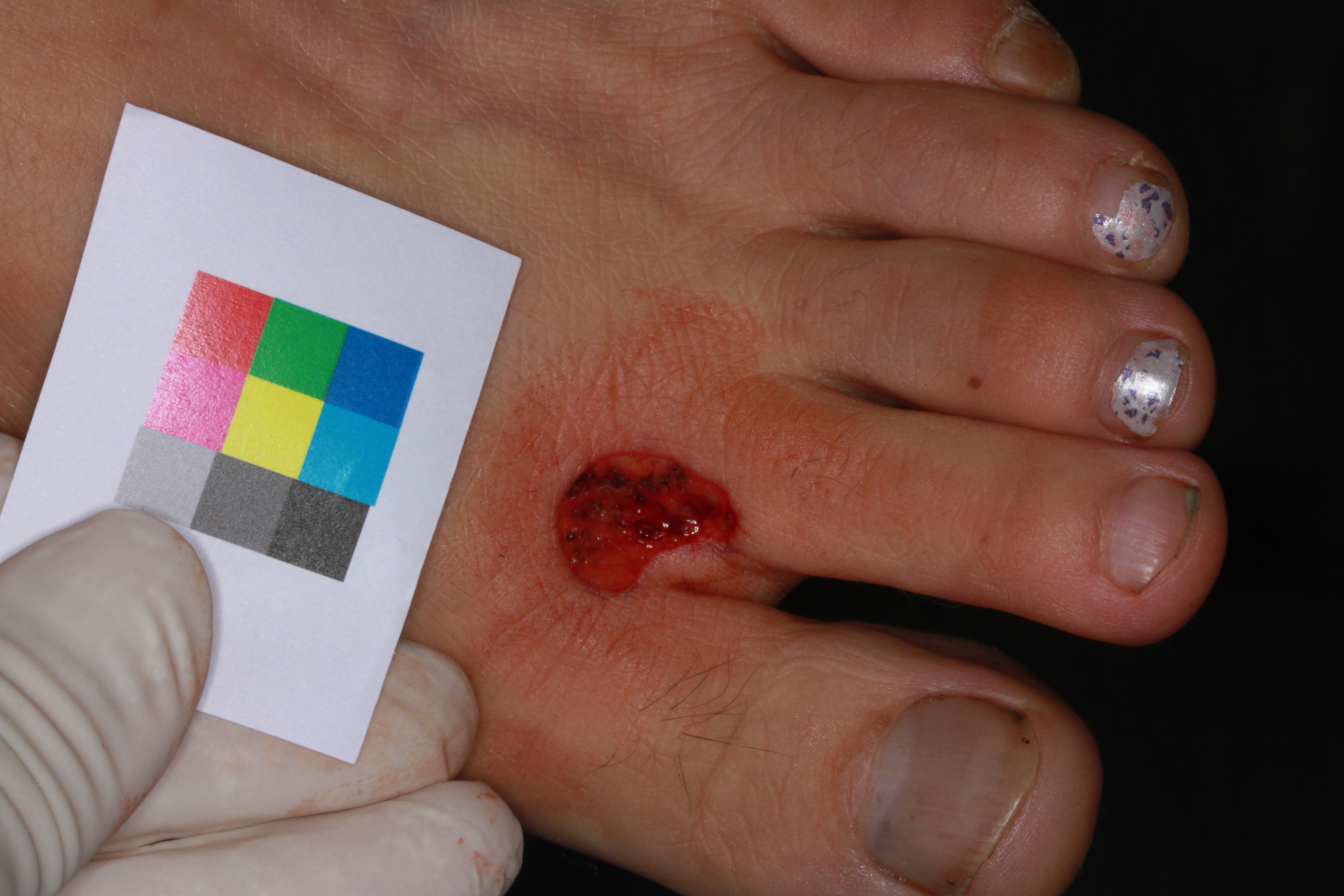

Supplement: S26 File — (ZIP) [file pone.0163092.s026.zip › 0812.JPG]

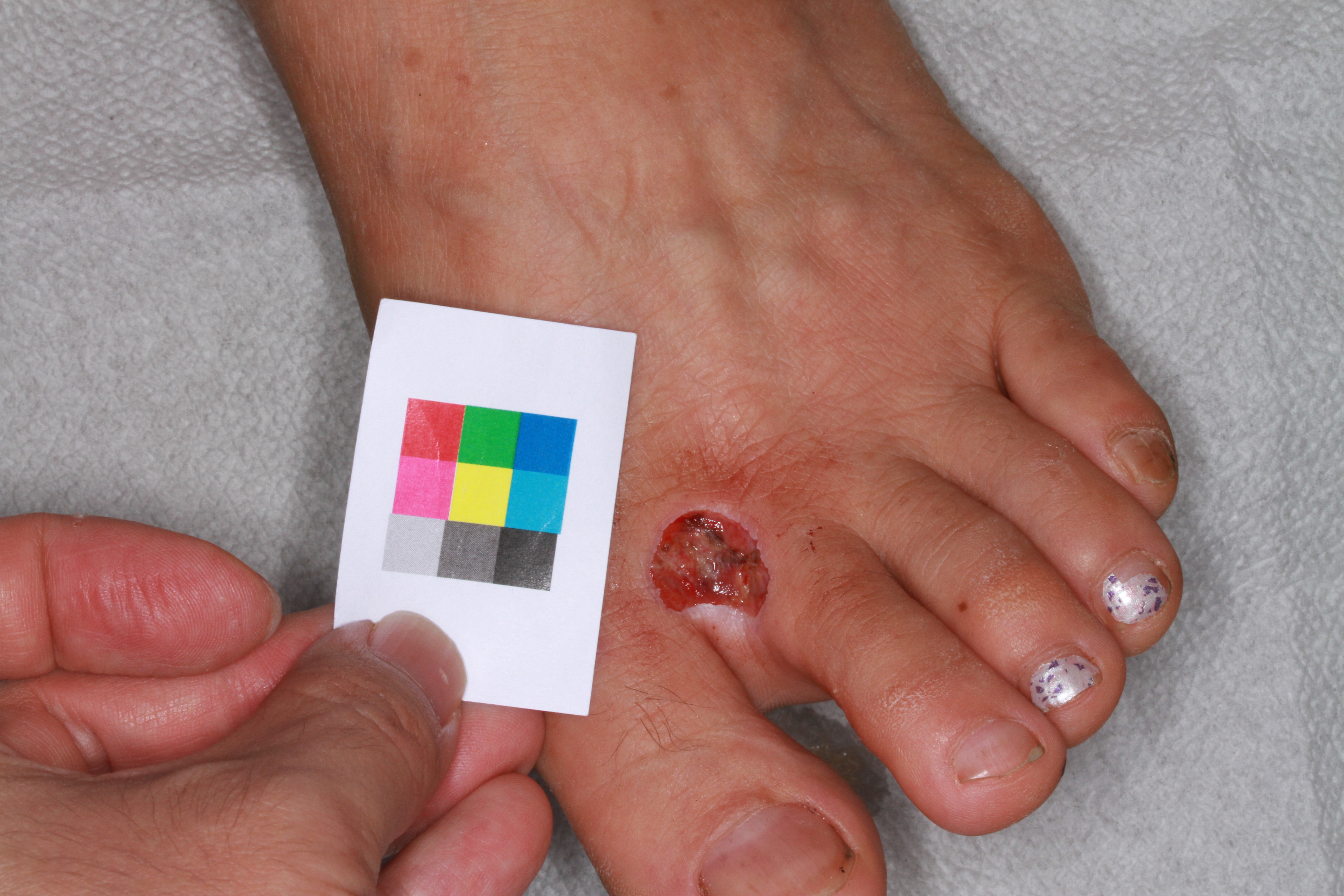

Supplement: S26 File — (ZIP) [file pone.0163092.s026.zip › 0813.JPG]

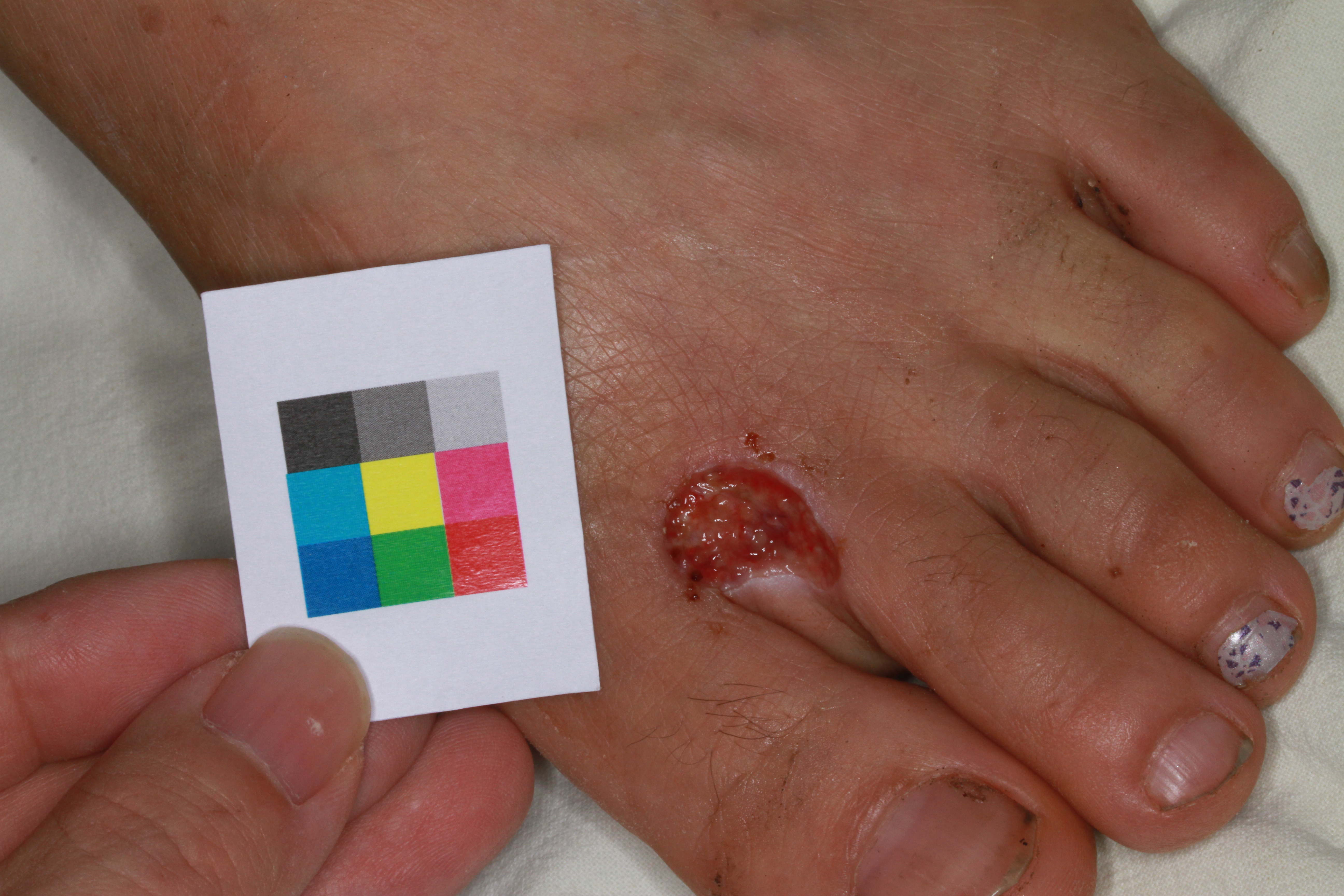

Supplement: S26 File — (ZIP) [file pone.0163092.s026.zip › 0819.JPG]

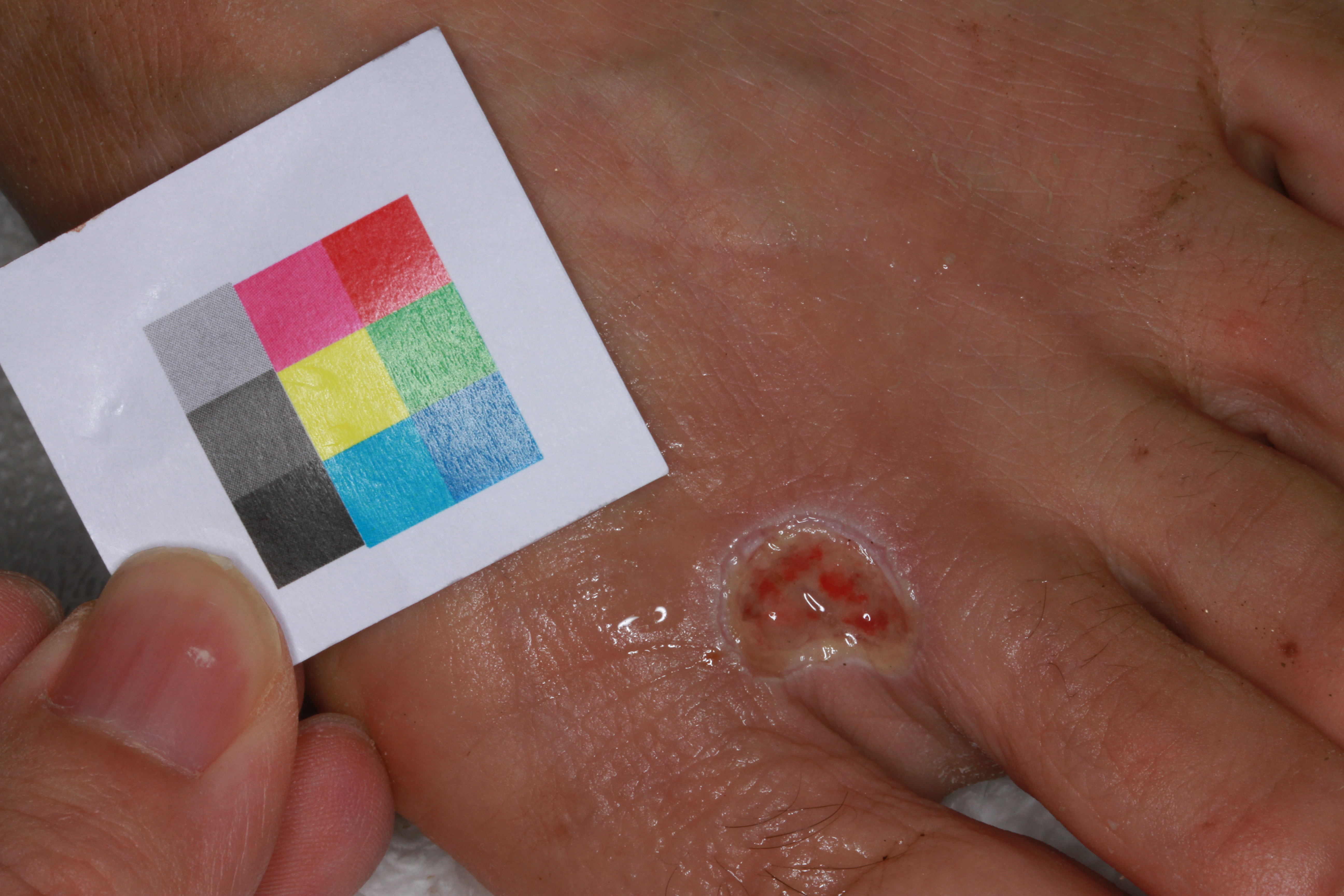

Supplement: S26 File — (ZIP) [file pone.0163092.s026.zip › 0826.JPG]

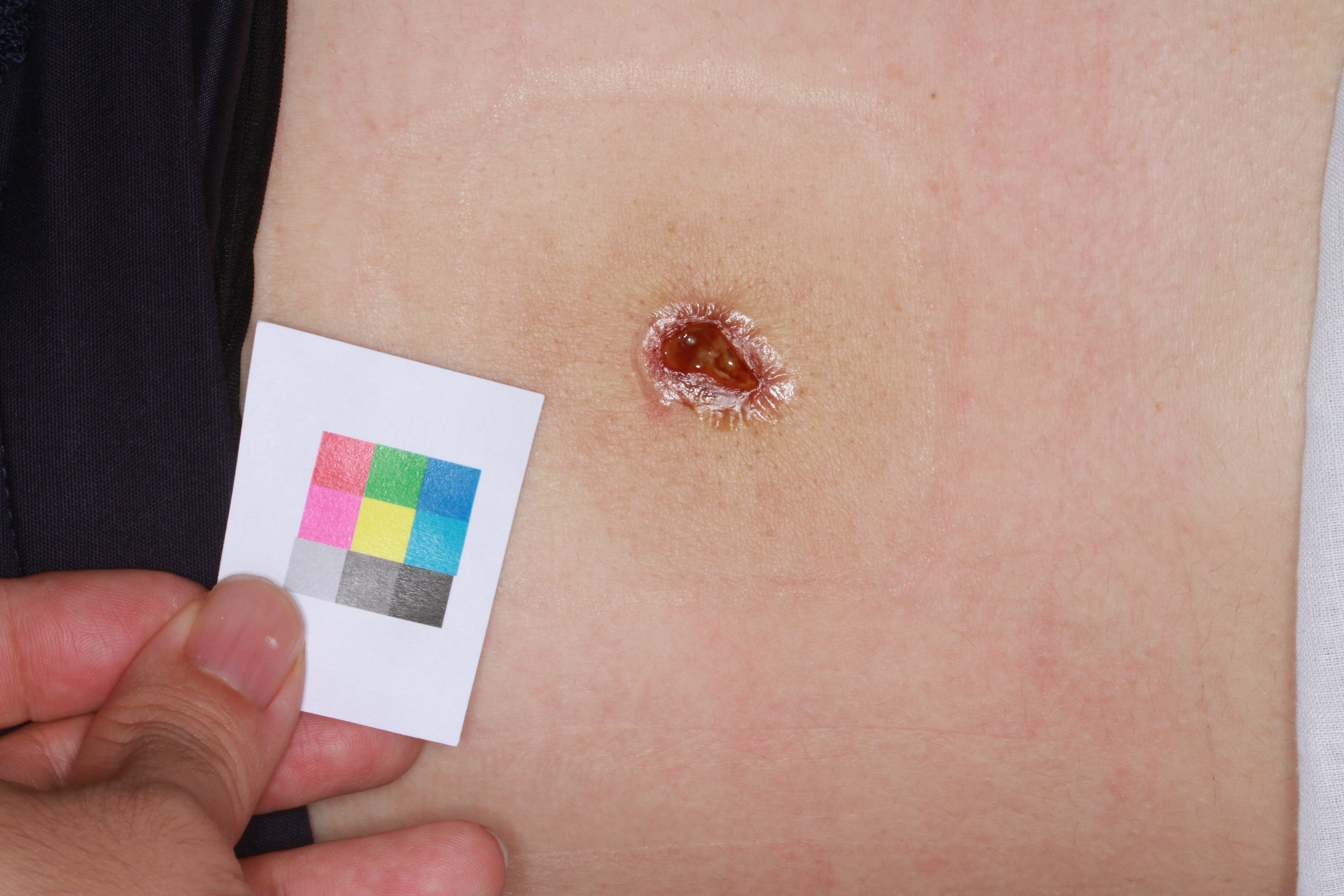

Supplement: S27 File — (ZIP) [file pone.0163092.s027.zip › 0827.JPG]

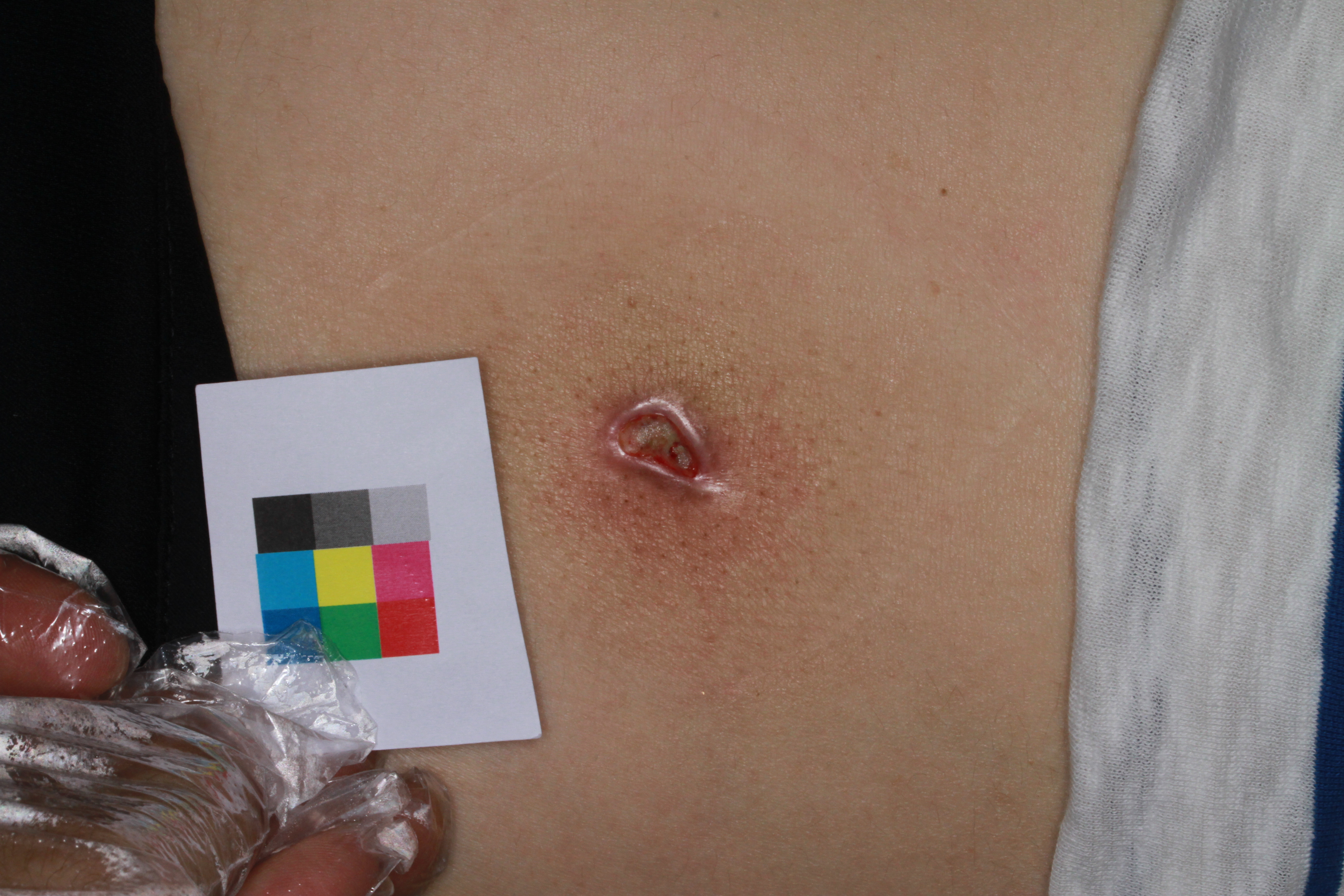

Supplement: S27 File — (ZIP) [file pone.0163092.s027.zip › 0902.JPG]

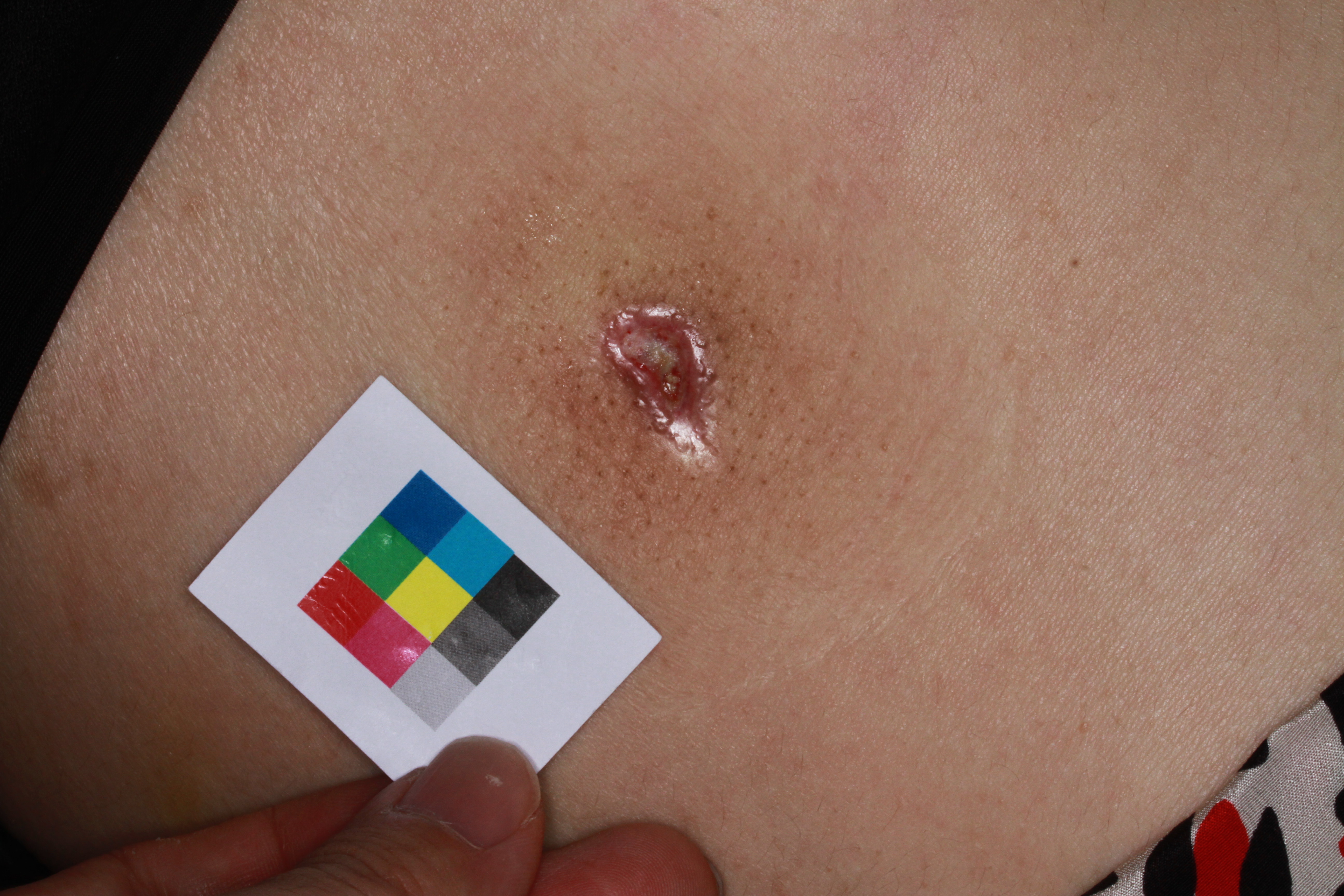

Supplement: S27 File — (ZIP) [file pone.0163092.s027.zip › 0905.JPG]

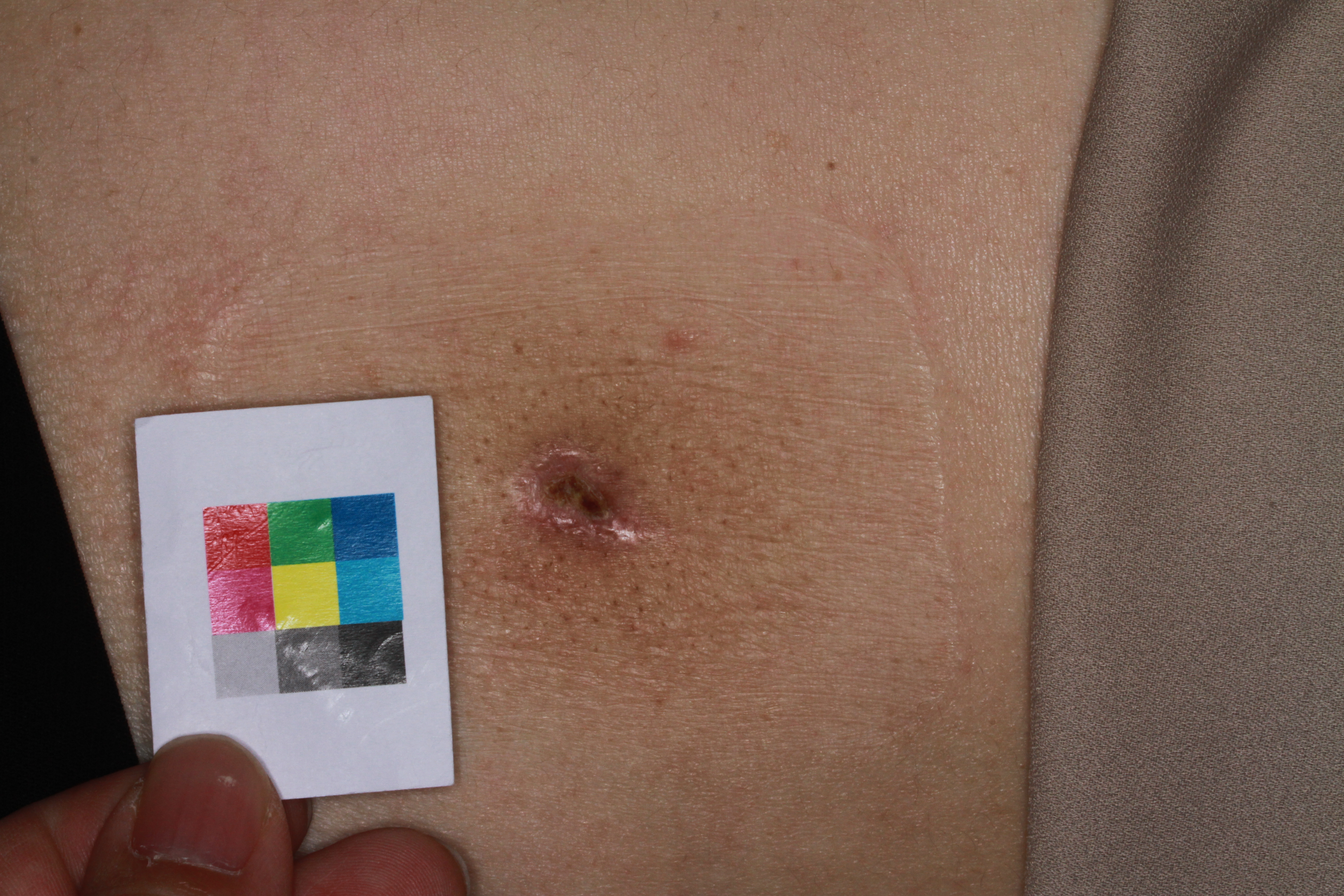

Supplement: S27 File — (ZIP) [file pone.0163092.s027.zip › 0916.JPG]

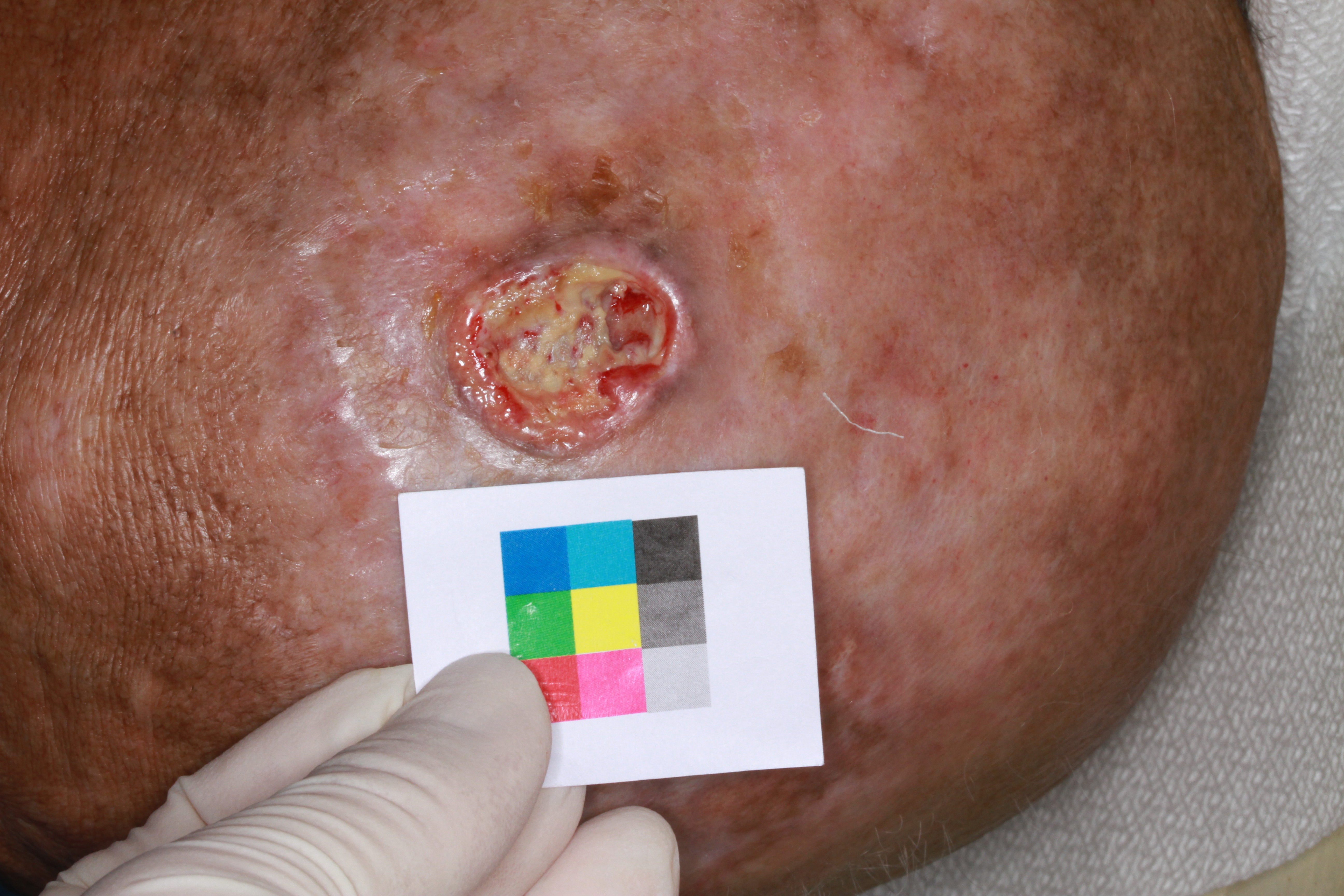

Supplement: S28 File — (ZIP) [file pone.0163092.s028.zip › 0905.JPG]

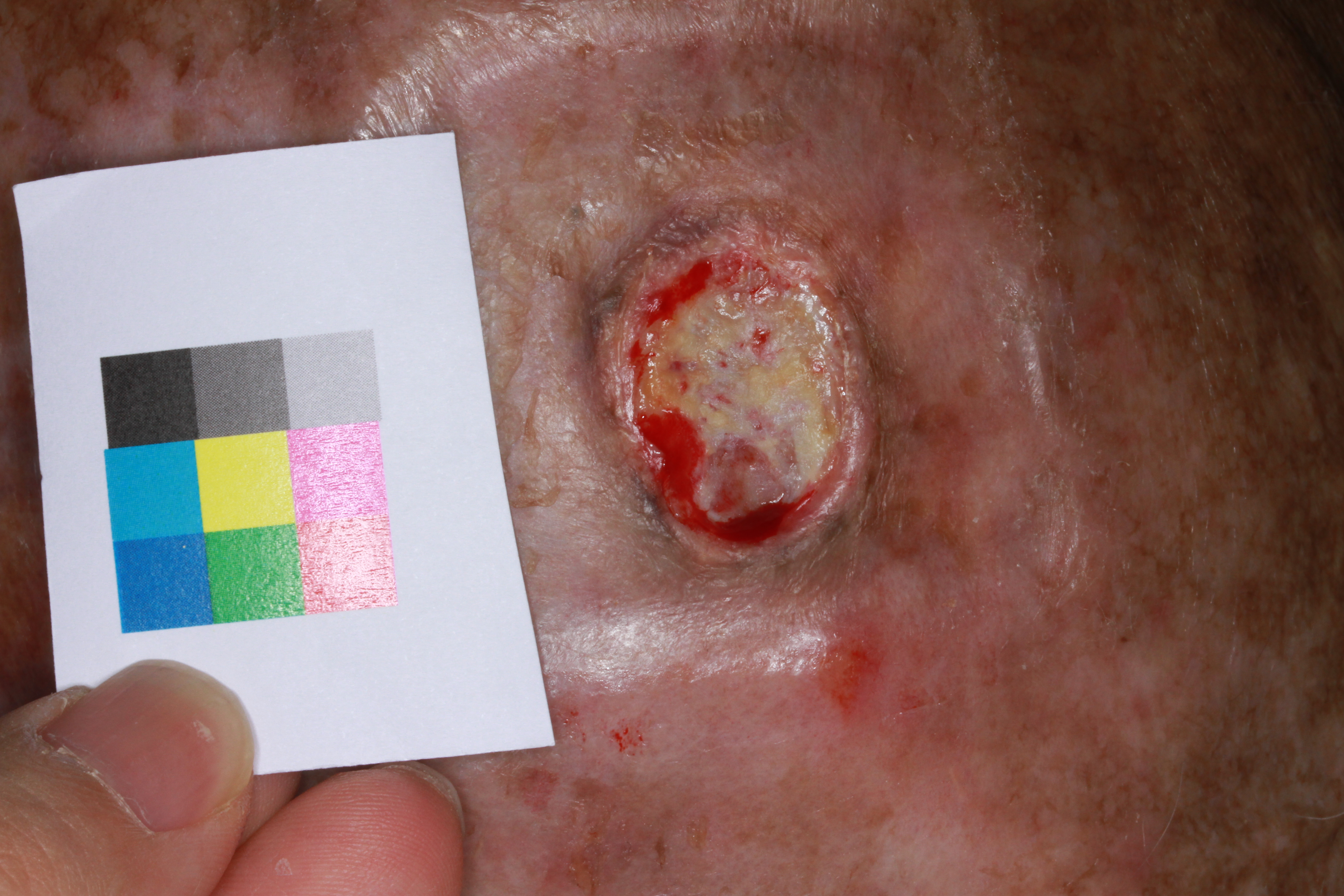

Supplement: S28 File — (ZIP) [file pone.0163092.s028.zip › 0916.JPG]

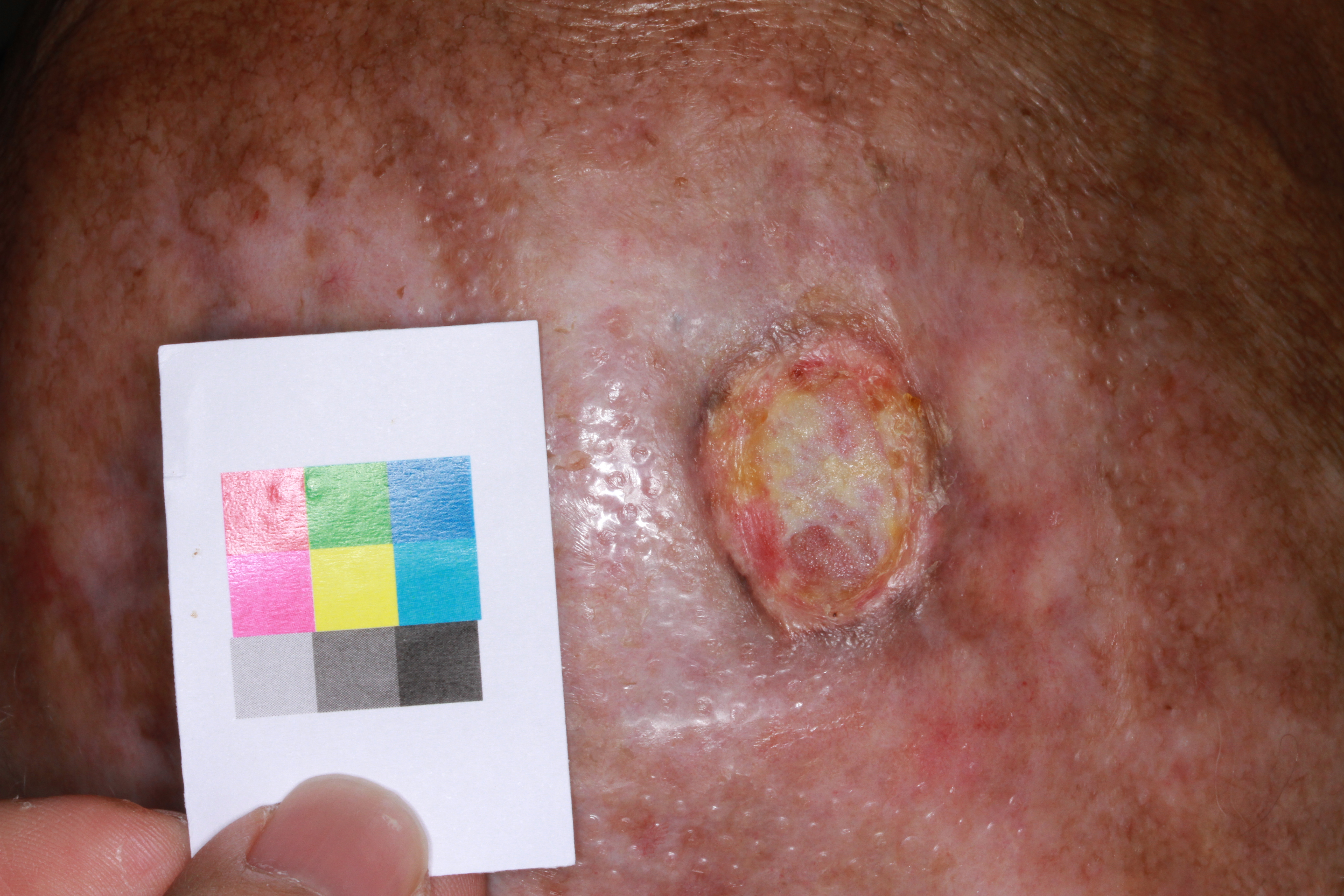

Supplement: S28 File — (ZIP) [file pone.0163092.s028.zip › 0930.JPG]

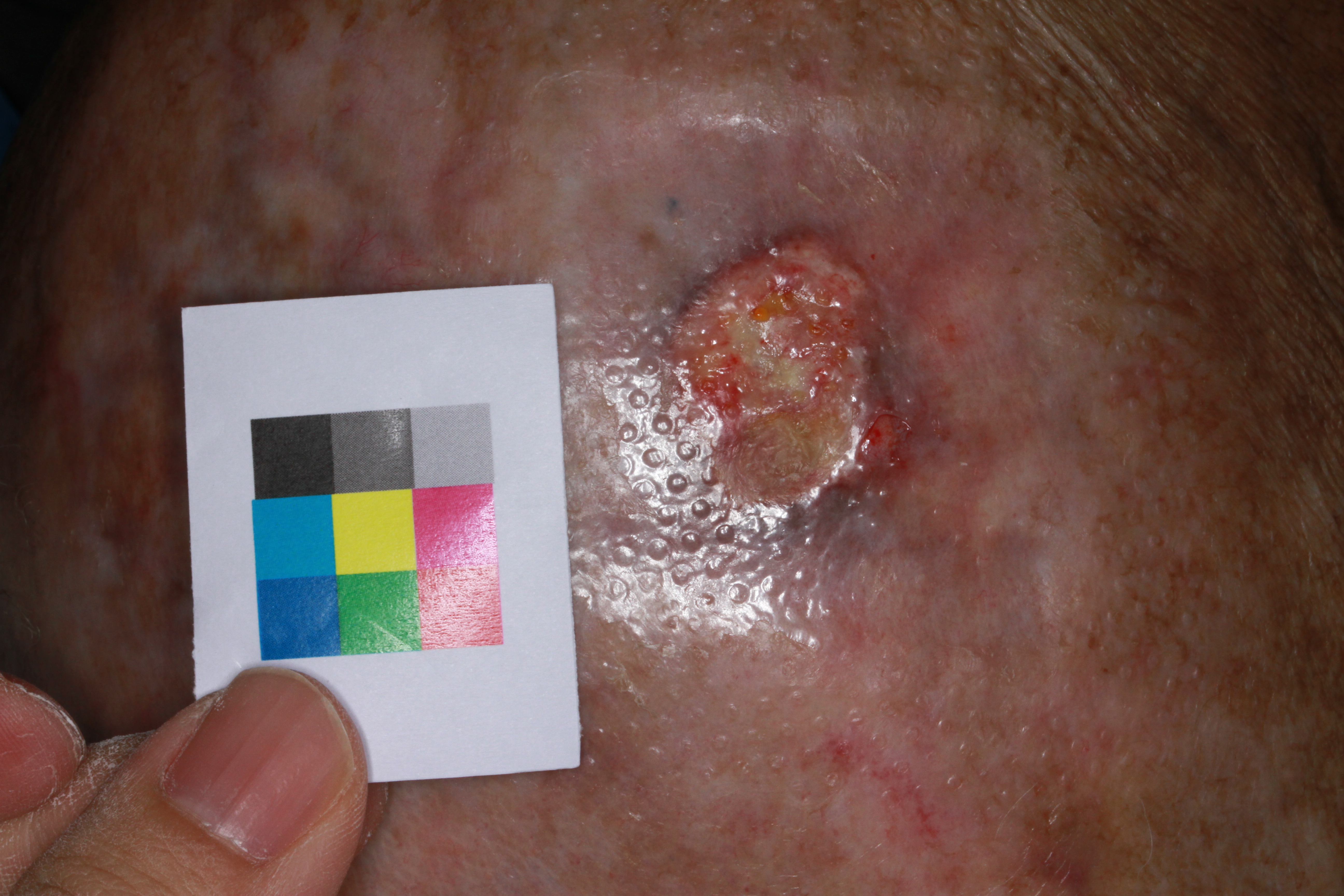

Supplement: S28 File — (ZIP) [file pone.0163092.s028.zip › 1028.JPG]

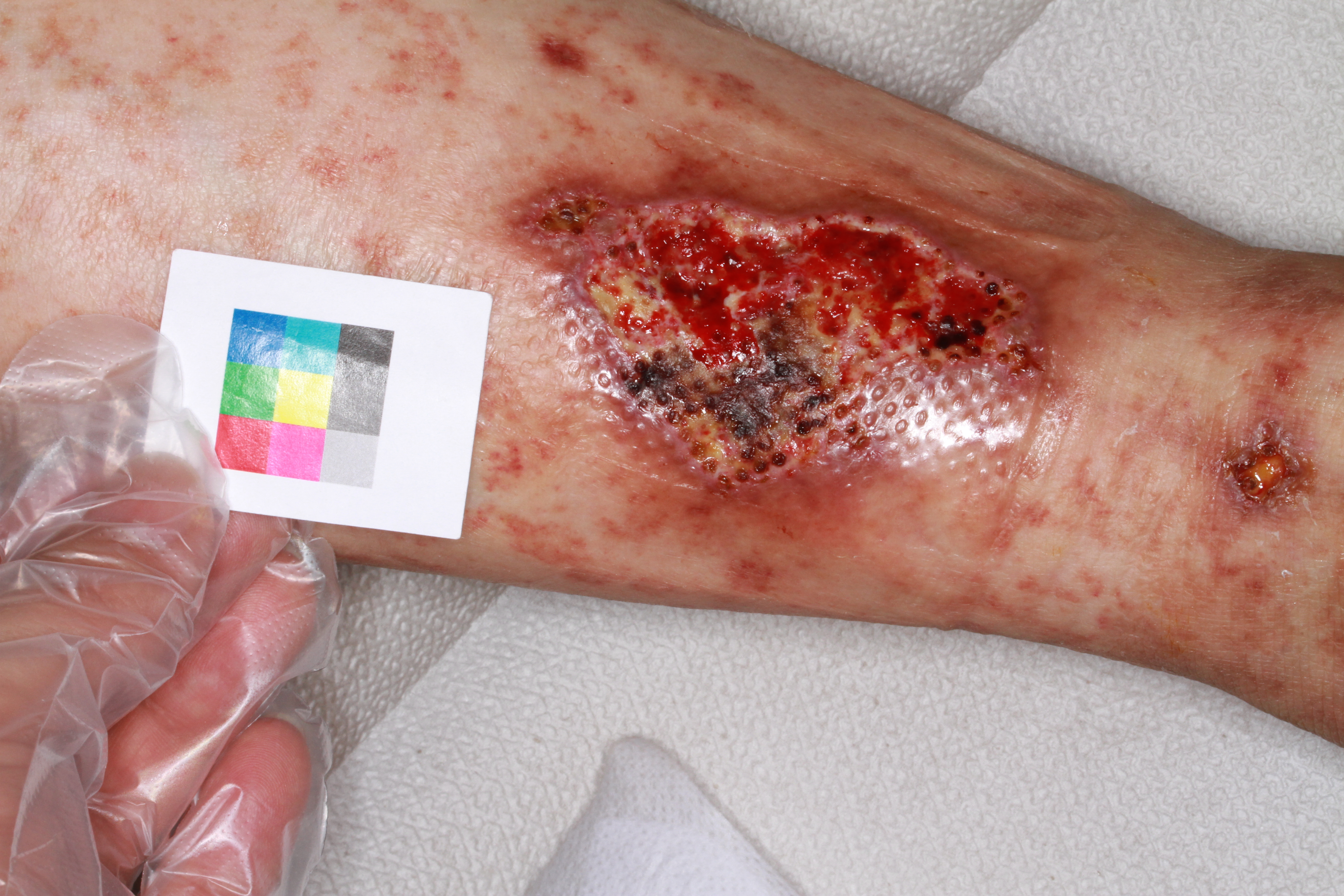

Supplement: S29 File — (ZIP) [file pone.0163092.s029.zip › 0701.JPG]

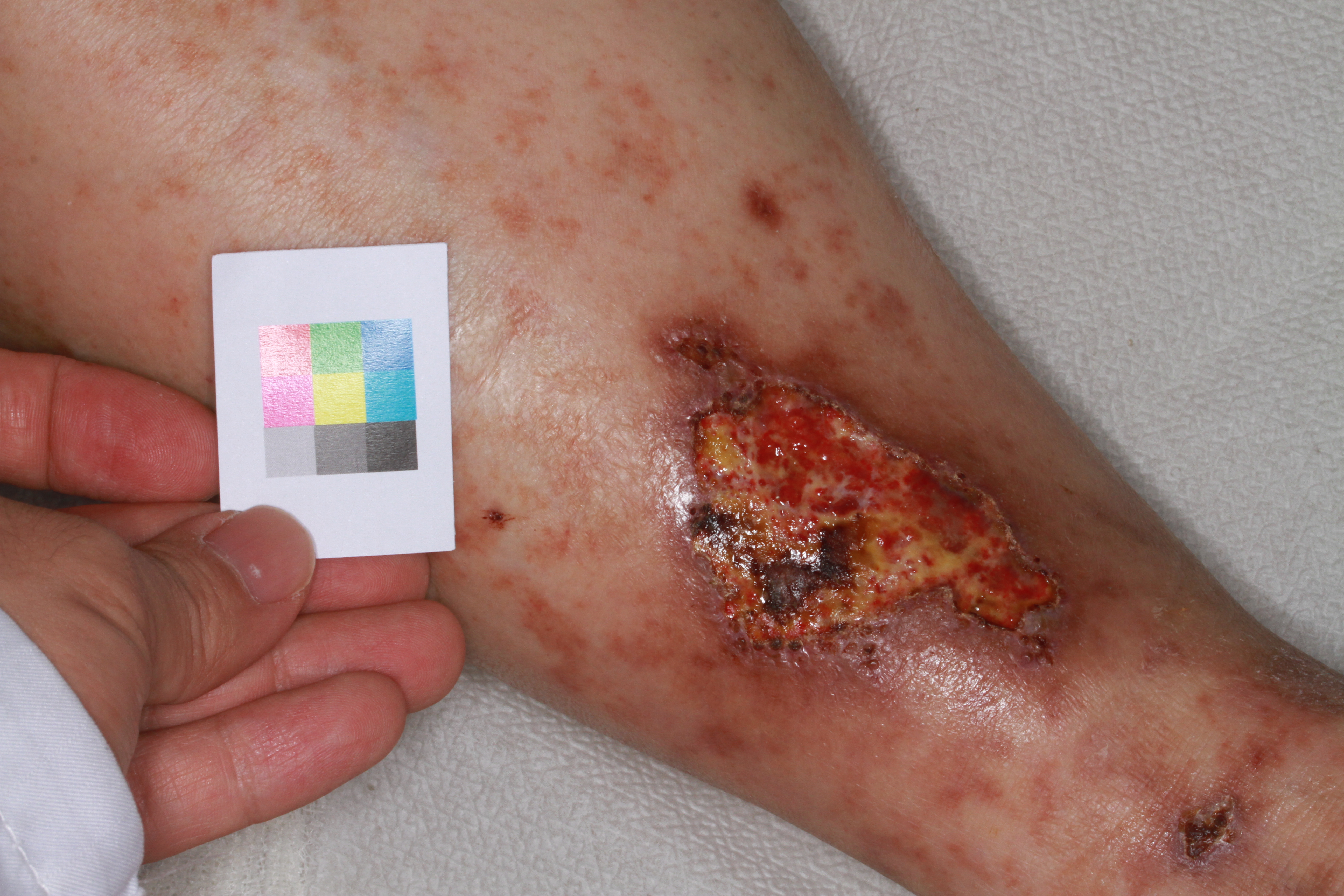

Supplement: S29 File — (ZIP) [file pone.0163092.s029.zip › 0709.JPG]

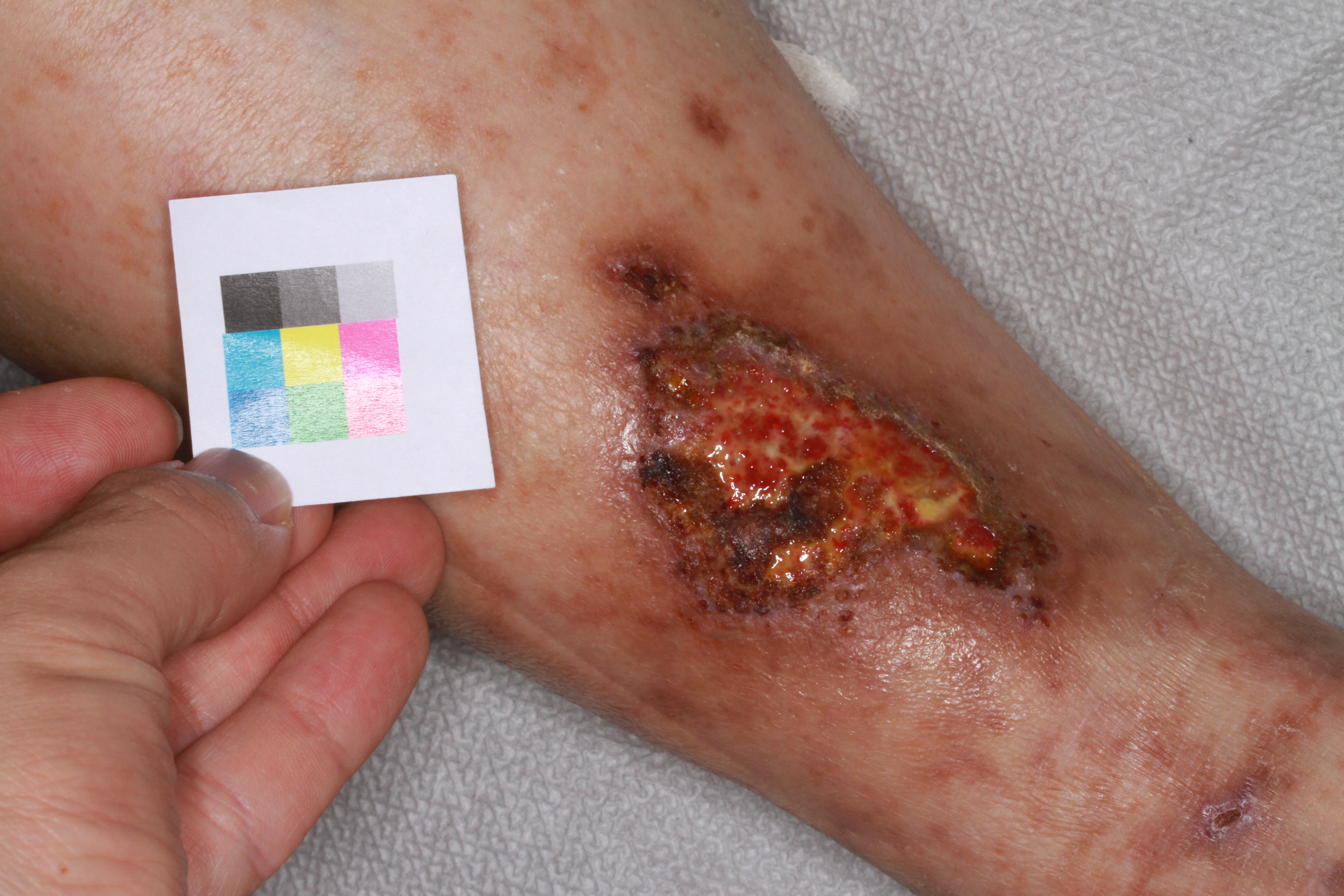

Supplement: S29 File — (ZIP) [file pone.0163092.s029.zip › 0723.JPG]

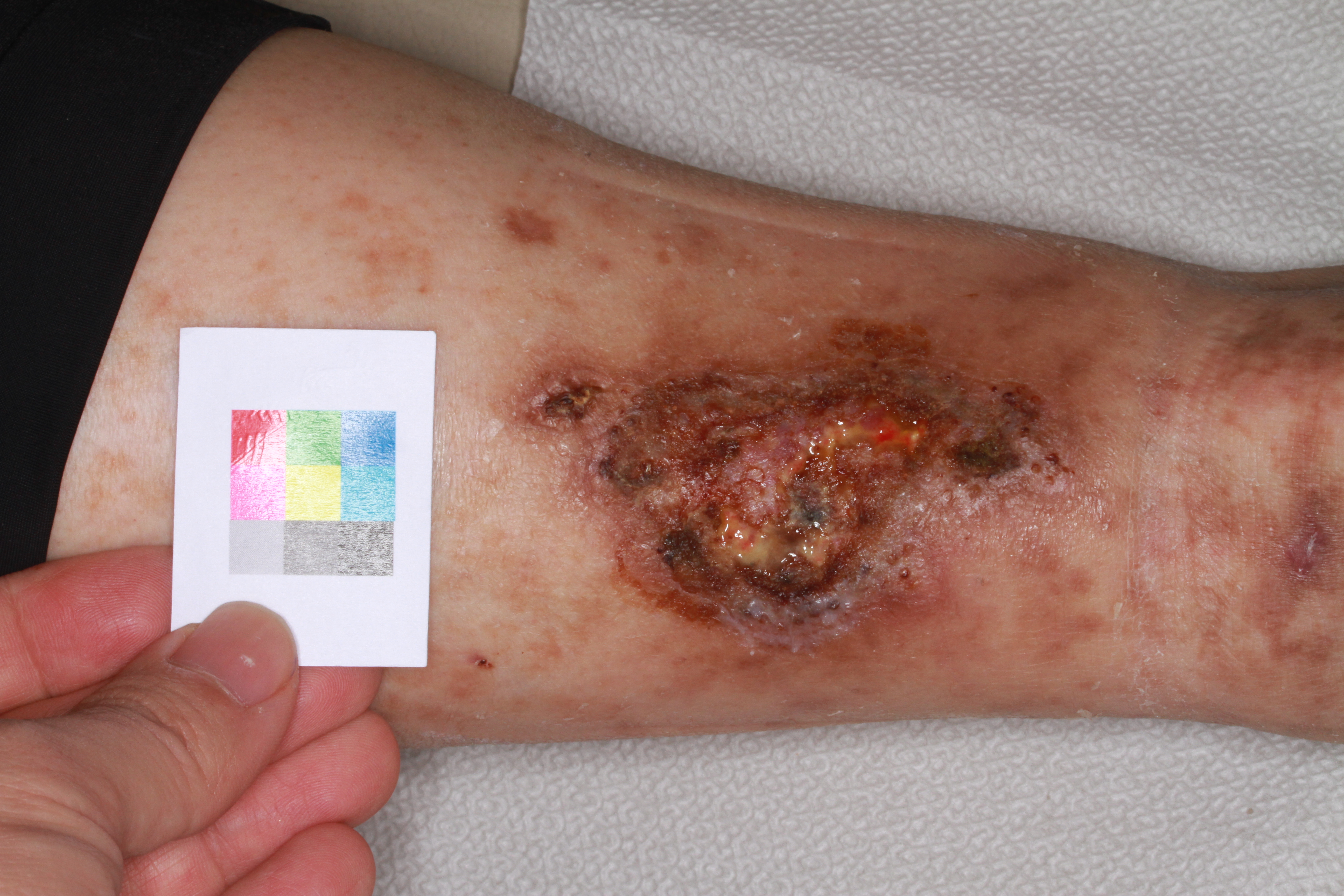

Supplement: S29 File — (ZIP) [file pone.0163092.s029.zip › 0806.JPG]

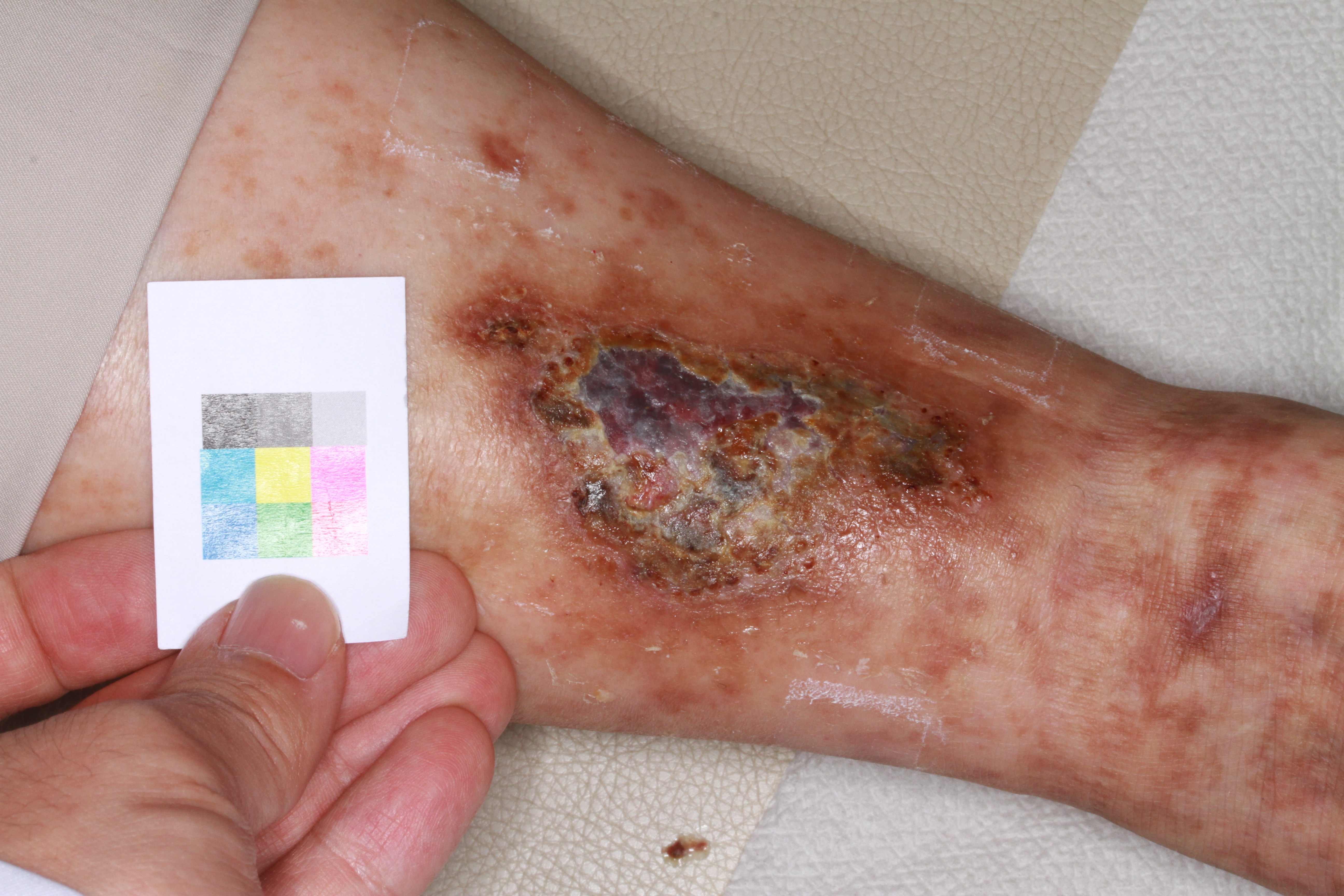

Supplement: S29 File — (ZIP) [file pone.0163092.s029.zip › 0820.JPG]

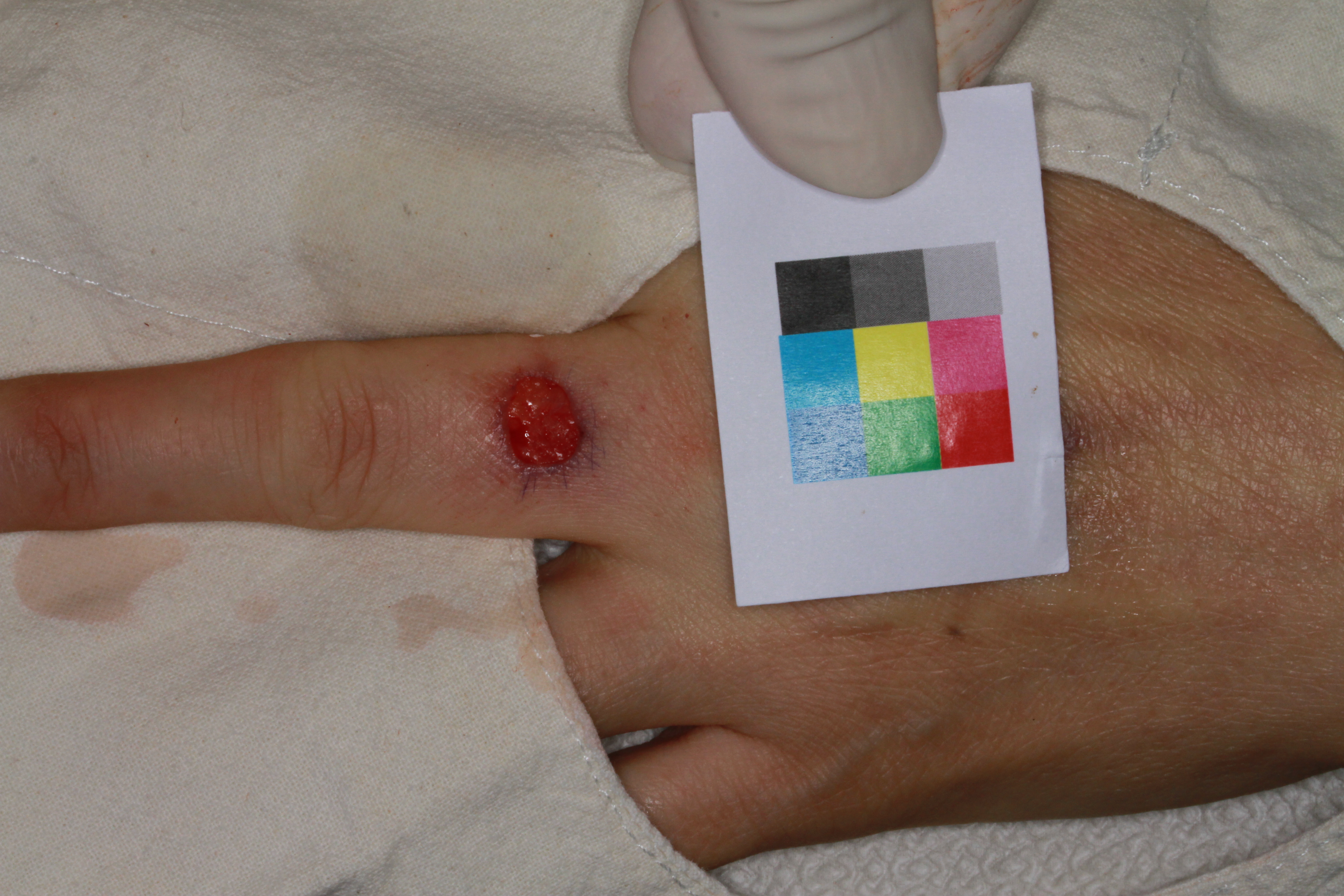

Supplement: S30 File — (ZIP) [file pone.0163092.s030.zip › 41014.JPG]

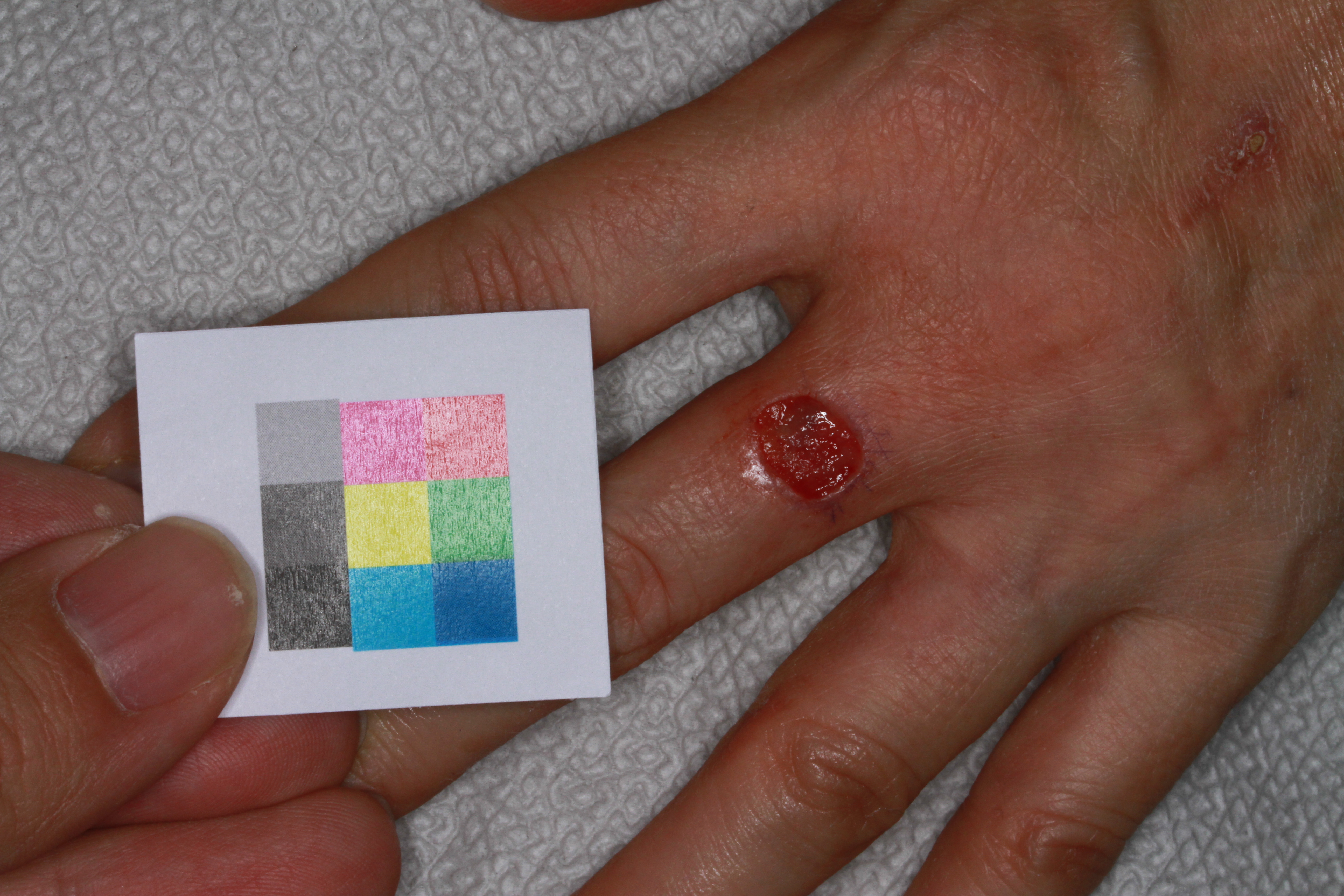

Supplement: S30 File — (ZIP) [file pone.0163092.s030.zip › 41015.JPG]

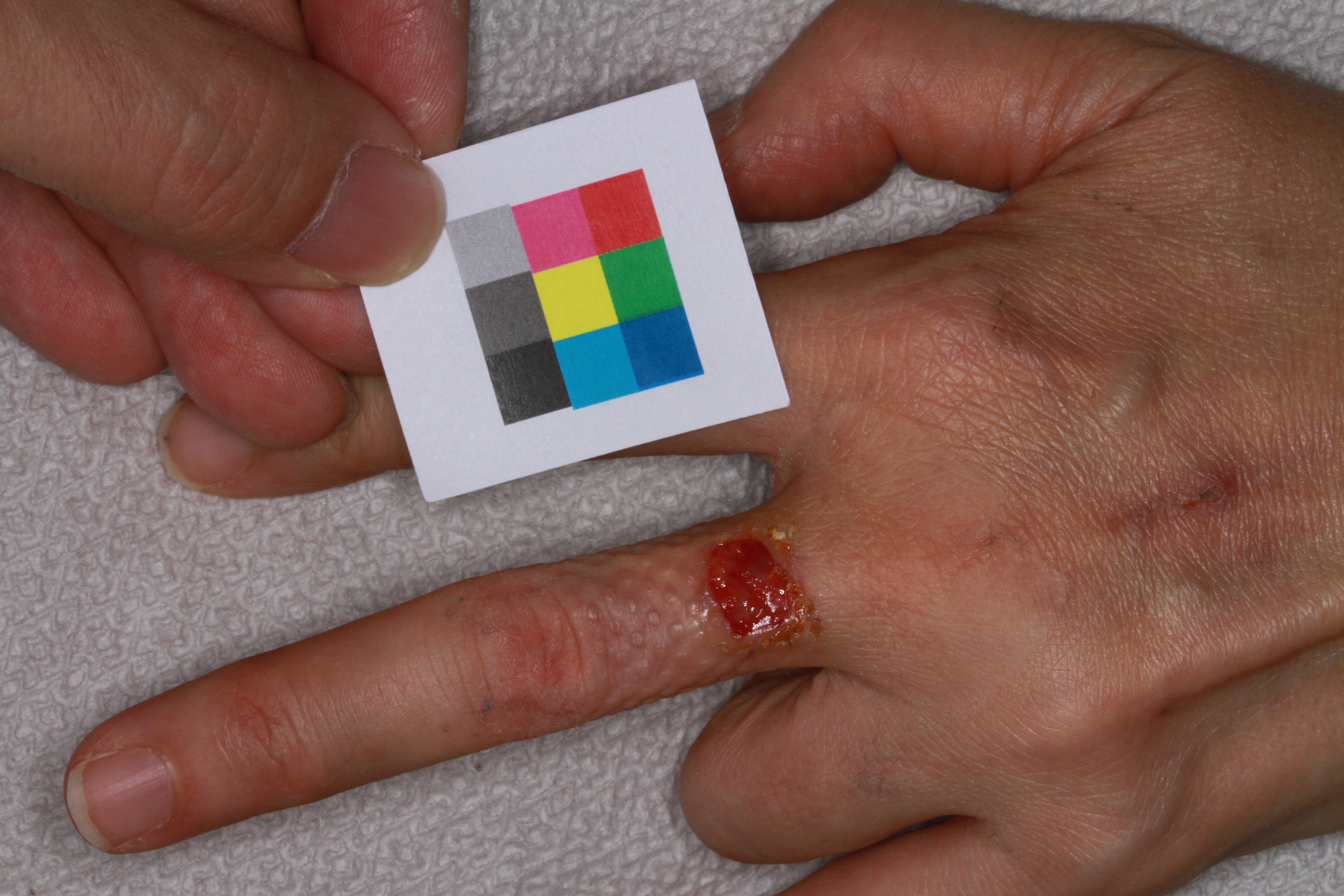

Supplement: S30 File — (ZIP) [file pone.0163092.s030.zip › 41017.JPG]

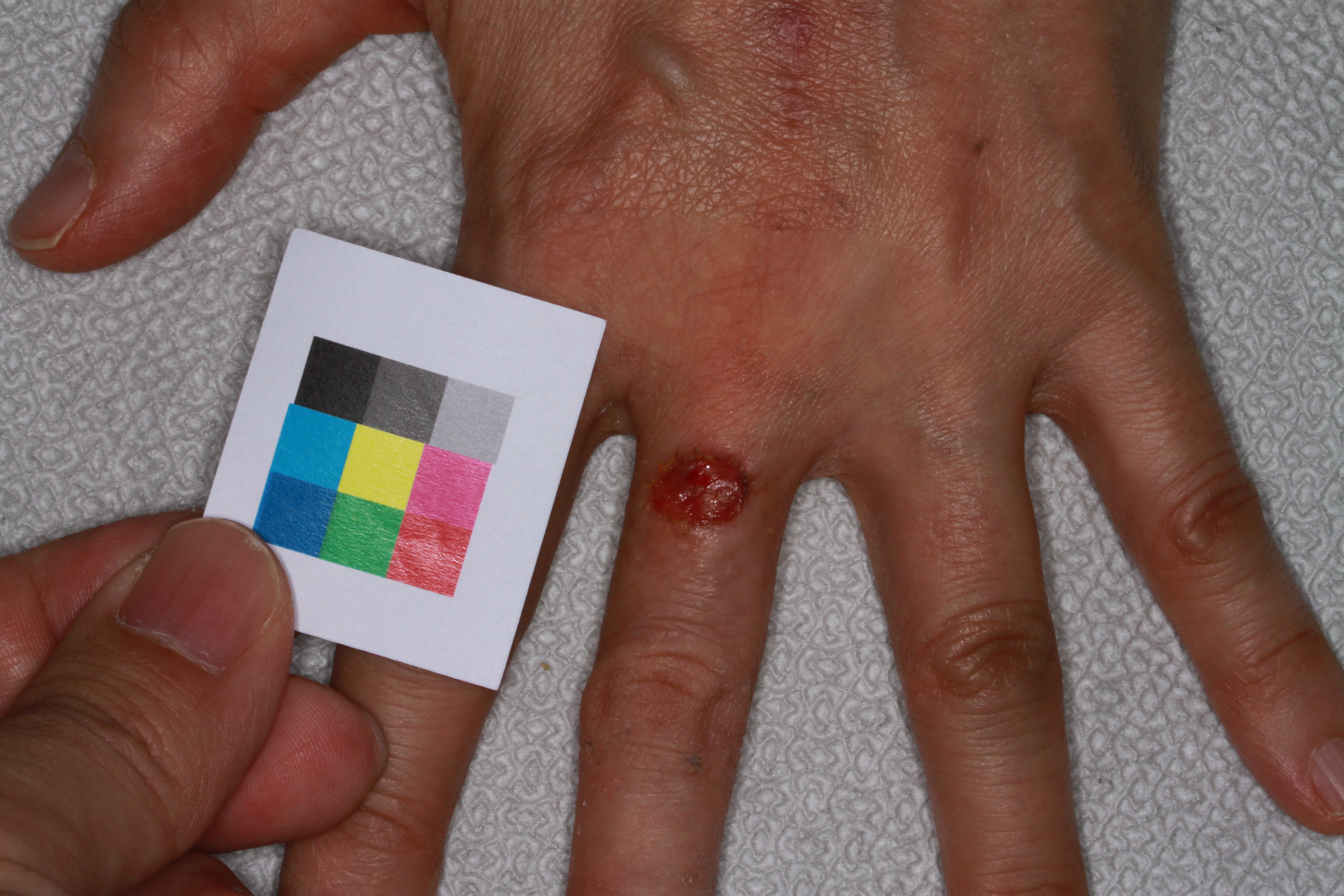

Supplement: S30 File — (ZIP) [file pone.0163092.s030.zip › 41020.JPG]

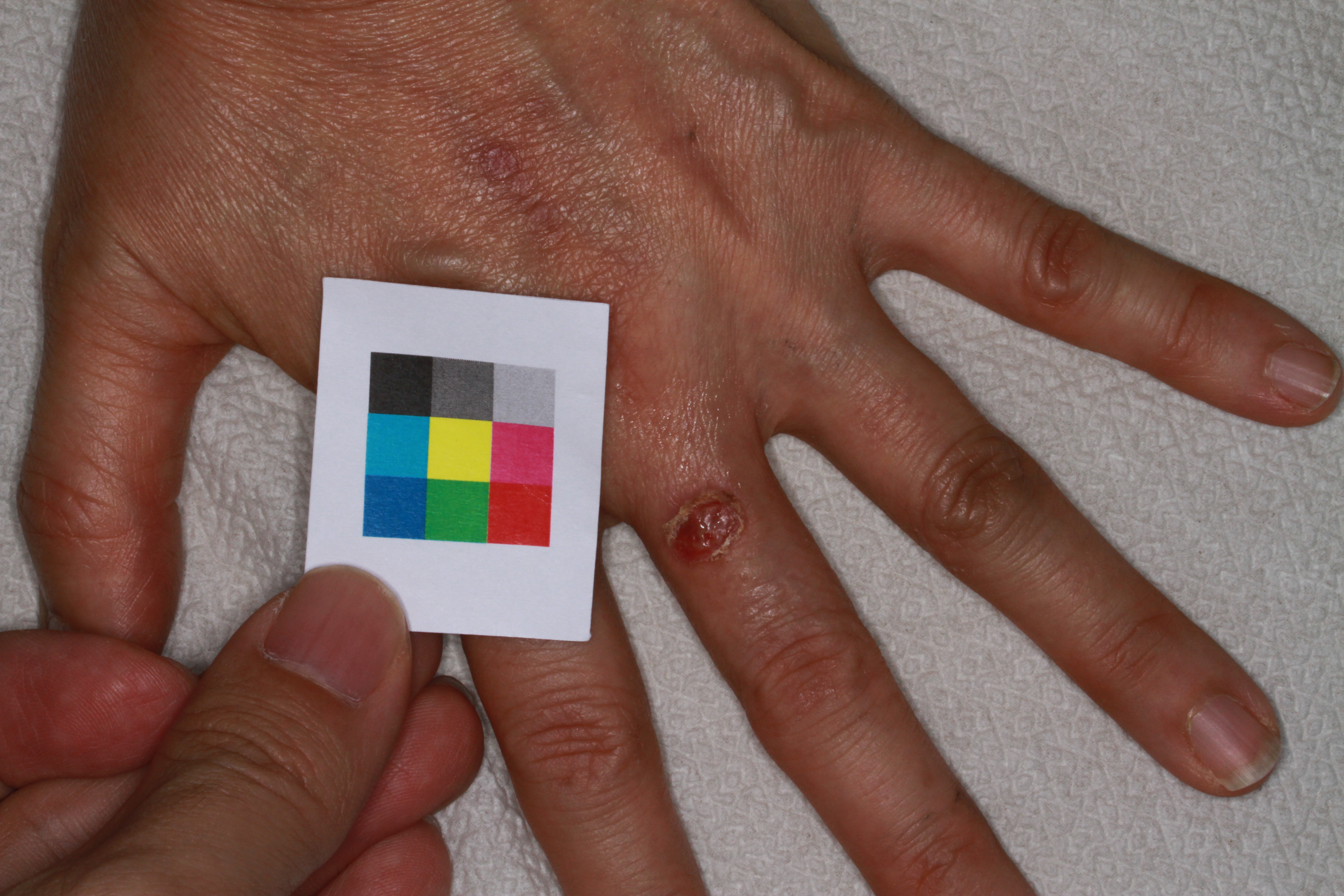

Supplement: S30 File — (ZIP) [file pone.0163092.s030.zip › 41024.JPG]

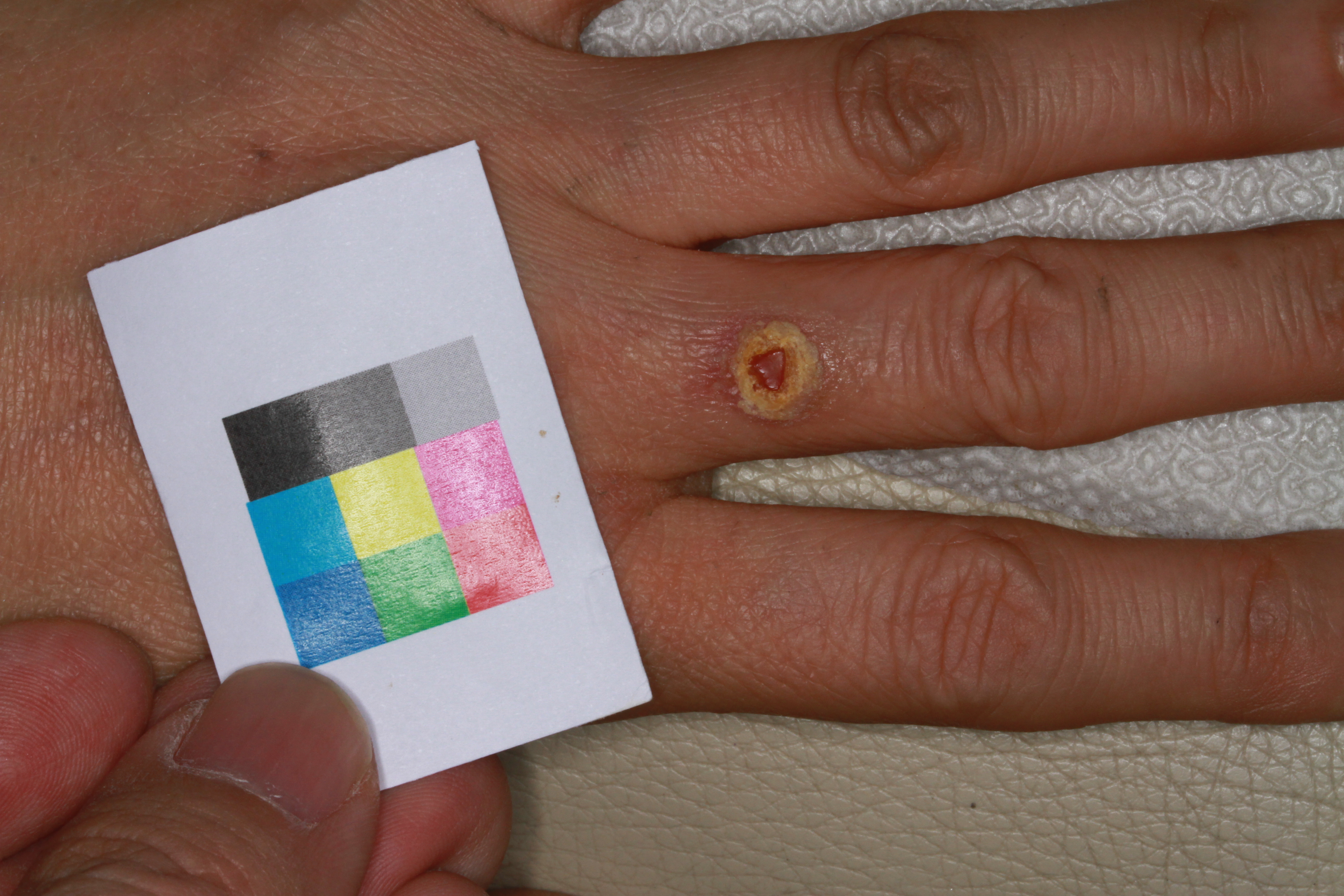

Supplement: S30 File — (ZIP) [file pone.0163092.s030.zip › 41031.JPG]
