# Supplementary figures and images for: Sequential Change of Wound Calculated by Image Analysis Using a Color Patch Method during a Secondary Intention Healing (part 3 of 3)
Source: PLoS One. 2016 Sep 20;11(9):e0163092. doi: 10.1371/journal.pone.0163092 (PMC5029888; doi:10.1371/journal.pone.0163092)

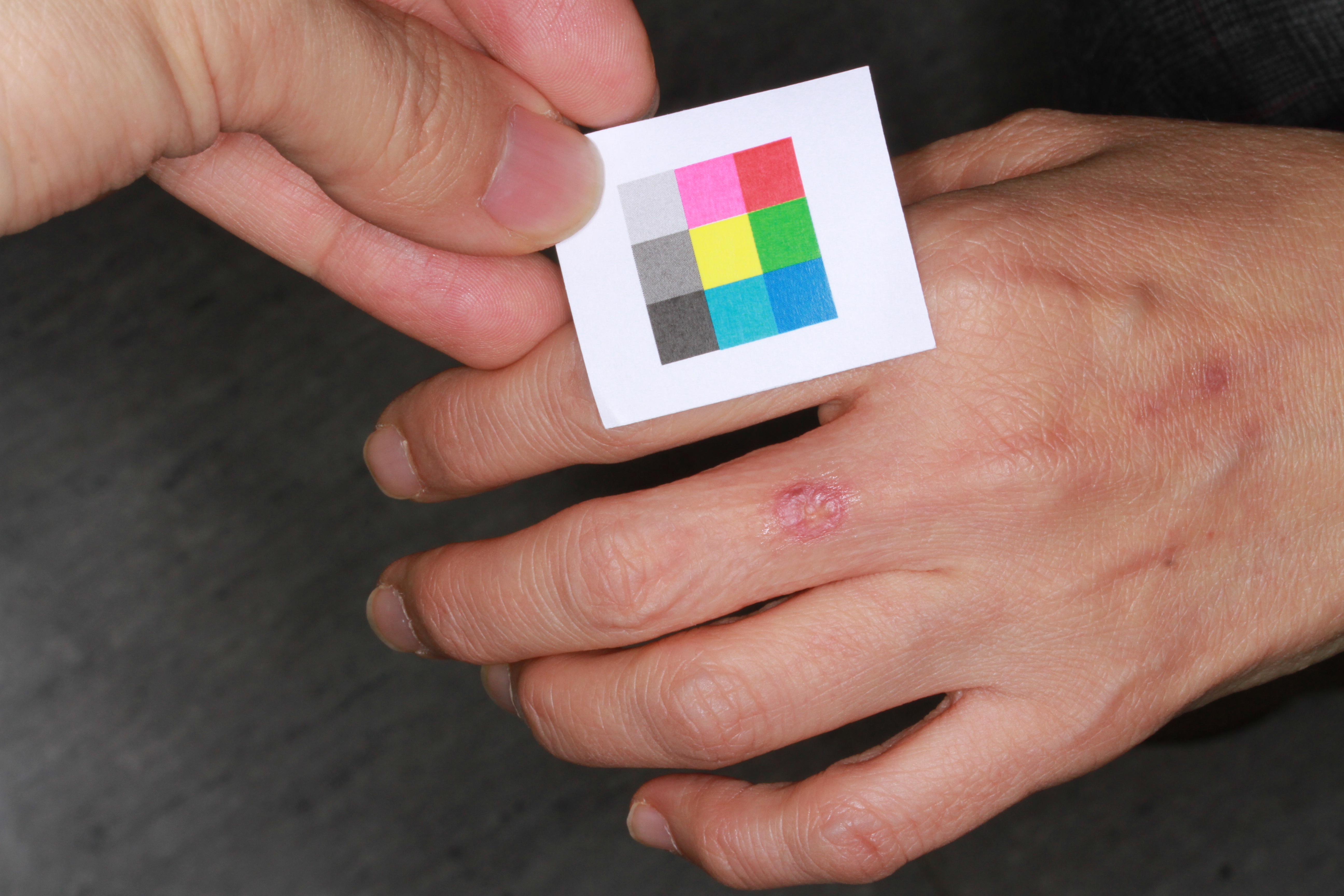

Supplement: S30 File — (ZIP) [file pone.0163092.s030.zip › 41117.JPG]
